# Cytonuclear Interactions and Subgenome Dominance Shape the Evolution of Organelle-Targeted Genes in the *Brassica* Triangle of U

**DOI:** 10.1093/molbev/msae043

**Published:** 2024-02-23

**Authors:** Shenglong Kan, Xuezhu Liao, Lan Lan, Jiali Kong, Jie Wang, Liyun Nie, Jun Zou, Hong An, Zhiqiang Wu

**Affiliations:** Marine College, Shandong University, Weihai 264209, China; Shenzhen Branch, Guangdong Laboratory of Lingnan Modern Agriculture, Key Laboratory of Synthetic Biology, Laboratory of the Ministry of Agriculture and Rural Affairs, Agricultural Genomics Institute at Shenzhen, Chinese Academy of Agricultural Sciences, Shenzhen 518120, China; Shenzhen Branch, Guangdong Laboratory of Lingnan Modern Agriculture, Key Laboratory of Synthetic Biology, Laboratory of the Ministry of Agriculture and Rural Affairs, Agricultural Genomics Institute at Shenzhen, Chinese Academy of Agricultural Sciences, Shenzhen 518120, China; Shenzhen Branch, Guangdong Laboratory of Lingnan Modern Agriculture, Key Laboratory of Synthetic Biology, Laboratory of the Ministry of Agriculture and Rural Affairs, Agricultural Genomics Institute at Shenzhen, Chinese Academy of Agricultural Sciences, Shenzhen 518120, China; College of Science, Health, Engineering and Education, Murdoch University, Murdoch, 6150 Western Australia, Australia; Shenzhen Branch, Guangdong Laboratory of Lingnan Modern Agriculture, Key Laboratory of Synthetic Biology, Laboratory of the Ministry of Agriculture and Rural Affairs, Agricultural Genomics Institute at Shenzhen, Chinese Academy of Agricultural Sciences, Shenzhen 518120, China; State Key Laboratory of Crop Stress Adaptation and Improvement, School of Life Sciences, Henan University, Kaifeng 475004, China; Shenzhen Branch, Guangdong Laboratory of Lingnan Modern Agriculture, Key Laboratory of Synthetic Biology, Laboratory of the Ministry of Agriculture and Rural Affairs, Agricultural Genomics Institute at Shenzhen, Chinese Academy of Agricultural Sciences, Shenzhen 518120, China; College of Science, Health, Engineering and Education, Murdoch University, Murdoch, 6150 Western Australia, Australia; Shenzhen Branch, Guangdong Laboratory of Lingnan Modern Agriculture, Key Laboratory of Synthetic Biology, Laboratory of the Ministry of Agriculture and Rural Affairs, Agricultural Genomics Institute at Shenzhen, Chinese Academy of Agricultural Sciences, Shenzhen 518120, China; National Key Laboratory of Crop Genetic Improvement, College of Plant Science and Technology, Huazhong Agricultural University, Wuhan 430070, China; Bioinformatics and Analytics Core, University of Missouri, Columbia, MO, USA; Shenzhen Branch, Guangdong Laboratory of Lingnan Modern Agriculture, Key Laboratory of Synthetic Biology, Laboratory of the Ministry of Agriculture and Rural Affairs, Agricultural Genomics Institute at Shenzhen, Chinese Academy of Agricultural Sciences, Shenzhen 518120, China

**Keywords:** allopolyploidy, cytonuclear interactions, mitochondria, plastid, subgenome dominance, *Brassica*

## Abstract

The interaction and coevolution between nuclear and cytoplasmic genomes are one of the fundamental hallmarks of eukaryotic genome evolution and, 2 billion yr later, are still major contributors to the formation of new species. Although many studies have investigated the role of cytonuclear interactions following allopolyploidization, the relative magnitude of the effect of subgenome dominance versus cytonuclear interaction on genome evolution remains unclear. The *Brassica* triangle of U features 3 diploid species that together have formed 3 separate allotetraploid species on similar evolutionary timescales, providing an ideal system for understanding the contribution of the cytoplasmic donor to hybrid polyploid. Here, we investigated the evolutionary pattern of organelle-targeted genes in *Brassica carinata* (BBCC) and 2 varieties of *Brassica juncea* (AABB) at the whole-genome level, with particular focus on cytonuclear enzyme complexes. We found partial evidence that plastid-targeted genes experience selection to match plastid genomes, but no obvious corresponding signal in mitochondria-targeted genes from these 2 separately formed allopolyploids. Interestingly, selection acting on plastid genomes always reduced the retention rate of plastid-targeted genes encoded by the B subgenome, regardless of whether the *Brassica nigra* (BB) subgenome was contributed by the paternal or maternal progenitor. More broadly, this study illustrates the distinct selective pressures experienced by plastid- and mitochondria-targeted genes, despite a shared pattern of inheritance and natural history. Our study also highlights an important role for subgenome dominance in allopolyploid genome evolution, even in genes whose function depends on separately inherited molecules.

## Introduction

Cytonuclear interactions refer to the stable interactive network and complexes resulting from cooperating nuclear and cytoplasmic gene products, which evolved over the course of eukaryotic history (Rand et al. 2004; Sloan et al. 2018). These interactions are essential in maintaining the fundamental physiological functions of eukaryotes and contribute to the evolution and speciation of organisms (Sackton et al. 2003; Chase 2007; Burton et al. 2013; Wolff et al. 2014; Sharbrough et al. 2017; Sloan et al. 2017; Zupok et al. 2021). The typical plant plastid genomes contain just 100 to 120 genes while plant mitochondrial genomes contain 50 to 60 genes in land plants (Tonti-Filippini et al. 2017; Kan et al. 2021; Wang et al. 2024), but the plastid and mitochondria are responsible for the lion's share of the cell's energy budget, requiring more than 2,000 and 3,000 proteins, respectively, in the process (Millar et al. 2005; Wijk and Baginsky 2011; Lee and Millar 2016). The vast majority of these organellar proteins and protein complexes are encoded by nuclear genes (organelle-targeted genes) or are jointly encoded by nuclear and organelle genomes, and the interactions between organelle-targeted genes and organelle genes are a source of both adaptive and nonadaptive effects on organisms, even reproductive isolation (Meiklejohn et al. 2013; Roux et al. 2016; Sloan et al. 2017, 2022; Postel and Touzet 2020). For instance, cytonuclear interactions in mitochondrial oxidative phosphorylation (OXPHOS) complex I between mitochondrial (e.g. *nad2* and *nad6*) and nuclear genes caused embryonic lethality in swordtail fish (Moran et al. 2024), whereas cytonuclear interactions in mitochondrial complex I and complex V between mitochondrial and nuclear genes were correlated with local adaptation in warblers (Wang et al. 2021). Furthermore, a large number of studies had shown that interactions between the pentatricopeptide repeat (PPR) gene family and mitochondrial genomes drove the evolution of cytoplasmic male sterility and the restoration of fertility in *Brassica*, *Citrus*, *Oryza*, *Raphanus*, and *Sorghum* (Chen and Liu 2014; Wanga et al. 2021; Wang et al. 2022). However, cytonuclear interaction is more crucial in allopolyploidization, which is one of the most important mechanisms of speciation. Approximately 13% of angiosperms are allopolyploid (Wood et al. 2009; Barker et al. 2016), including many important commercial crops (e.g. wheat, soybean, and cotton). This is because the diverged nuclear genomes from both parents are reintegrated into 1 nucleus through biparental inheritance, while the organelle genomes from only a single parent (usually maternal) are retained through uniparental inheritance during hybridization (Barker et al. 2016; Steige and Slotte 2016; Camus et al. 2022). By undergoing this process, the original cytonuclear balance is broken in hybrids, but how the organelle-targeted genes encoded by different nuclear subgenomes respond to this change remains an open and important question in evolutionary biology.

Previous work on cytonuclear interactions had mainly focused on ribulose-1,5-bisphosphate carboxylase/oxygenase (RuBisCo), the most abundant protein complex in leaf tissue, which was jointly encoded by the plastid gene (*rbcL*) and the nuclear gene family (*rbcS*). Consistent with the expectation that organelle-targeted genes in different subgenomes were under different selective pressures from organelle genomes, the maternally derived *rbcS* homoeologs were preferentially expressed in some ancient allopolyploids (e.g. *Arabidopsis*, *Arachis*, *Gossypium*, and *Nicotiana*; Gong et al. 2012, 2014). Conversely, this pattern was not found in young allopolyploid (*Tragopogon*) and newly synthetic allopolyploid (*Oryza* and *Cucumis*; Sehrish et al. 2015; Wang et al. 2017; Zhai et al. 2019). Therefore, a long period of adaptive coevolution may be necessary to fix the sites of cytonuclear interaction following hybridization. Further analysis of different functional domains of small subunit revealed that maternal to paternal gene conversion occurred only in the transit peptides and the C-terminal *β* strands in the *Arachnids*, *Arabidopsis*, *Gossypium*, and *Nicotiana* (Gong et al. 2014). Cytonuclear interactions have been shown to affect gene conversion in different domains, leading to the question of whether similar rules apply to other complexes and whether they are related to their structure. With the advancement of high-quality genome resources, Sharbrough et al. (2022) investigated the evolutionary pattern of all organelle-targeted genes in 6 independently formed allopolyploid angiosperms (*Brachypodium*, *Chenopodium*, *Coffea*, *Gossypium*, *Nicotiana*, and *Triticum*) and found that the evolutionary rate of organelle-targeted genes encoded by different subgenomes was different, but the organelle-targeted genes encoded by the maternal subgenome were not retained at higher rates or evolved more slowly than paternally derived gene copies. These results suggest that the evolution of organelle-targeted genes in allopolyploids may be influenced by multiple factors. Similarly, subgenome dominance, including differences in expression levels and evolutionary rates across subgenomes in allopolyploids, is also influenced by intrinsic features of the different subgenomes (e.g. transposable elements, epigenetic modifications, and cytonuclear incompatibility; Alger and Edger 2020; Roy 2021). Furthermore, previous studies on cytonuclear interactions always selected 1 or a few unrelated allopolyploids, making it impossible to distinguish whether those differences in gene retention rate, expression level, and evolutionary rate among subgenomes were caused by cytonuclear interactions or the intrinsic features of subgenomes. Perhaps the hybrids from the reciprocal cross are the ideal materials to explore this problem, but previous studies have also shown that organelle-targeted genes responding to cytoplasmic changes require long time periods to accumulate compensatory changes (Sehrish et al. 2015; Wang et al. 2017; Zhai et al. 2019). Therefore, it is necessary to select a group of allopolyploid species that have differentiated for a long time and shared parents.

**Fig. 1.**
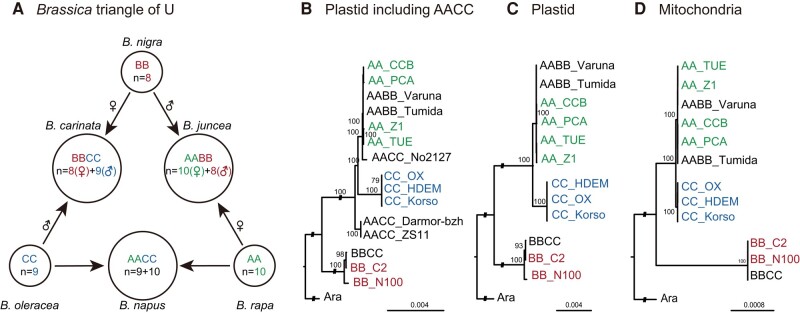
Phylogenies of the 6 species in the *Brassica* triangle of U based on protein-coding genes of organelle genomes. a) Relationships of the 6 species in the *Brassica* triangle of U. “♂” indicates the paternal progenitor, while “♀” indicates the maternal progenitor. b) The plastid phylogeny including all 6 species in the *Brassica* triangle of U. c) The plastid phylogeny of species in the *Brassica* triangle of U excluding *B. napus* (AACC). d) The mitochondrial phylogeny of species in the *Brassica* triangle of U excluding *B. napus* (AACC). The number around the nodes represents the bootstrap value greater than 75.

The U triangle in *Brassica*, containing 3 diploids and 3 allopolyploids famous for their morphological diversity and economic significance (Fig. 1a; Nagaharu 1935), is an ideal model to study polyploid evolution because the 3 diploid parents have given rise to distinct and reciprocally formed allopolyploids. Phylogenomic analysis indicated that *Brassica nigra* (BB) diverged with the common ancestor of *Brassica rapa* (AA) and *Brassica oleracea* (CC) around 6 million yr ago (mya), followed by divergence of *B. rapa* (AA) and *B. oleracea* (CC) around 3 mya (Yang et al. 2016; Song et al. 2021). The 3 allopolyploids formed from these 3 diploids via hybridization 8,000 to 70,000 yr ago: *Brassica napus* (AACC), *Brassica juncea* (AABB), and *Brassica carinata* (BBCC; Song et al. 2021; Hu et al. 2022; Yim et al. 2022). Both *B. juncea* (AABB) and *B. carinata* (BBCC) appeared to have formed in a single hybridization event, with *B. nigra* (BB) acting as the paternal progenitor of *B. juncea* (AABB) and the maternal progenitor of *B. carinata* (BBCC). However, the maternal progenitor of *B. napus* (AACC) remains unclear (An et al. 2019; Xue et al. 2020; Liu et al. 2022). Gong et al. (2014) also investigated the evolutionary pattern of *rbcS* in *B. napus* (AACC), and they observed differential expression between homoeologs. Despite the presence of nonsynonymous variants between *B. rapa* (AA) and *B. oleracea* (CC) in both the plastid and nuclear genomes, there did not appear to be maternally biased expression of nuclear genes that encode plastid protein complexes in *B. napus* (AACC; Ferreira de Carvalho et al. 2019). *Brassica napus* (AACC) is, however, the youngest allotetraploid in the triangle of U, and it has complex origin, so straightforward predictions of cytonuclear coevolution may not be entirely appropriate.

In this study, we investigated the patterns of cytonuclear interactions by comparing gene retention rate, homoeolog expression bias, and gene conversion/homoeologous exchange at the whole-genome level and in the core cytonuclear enzyme complexes in the *Brassica* triangle of U. We selected RuBisCo, the most well-studied complex on cytonuclear interactions, to represent the plastid core complexes, and mitochondrial complex III, a relatively simple complex encoded by mitochondrial and nuclear genomes, as the representative of the mitochondrial core complexes. As part of this analysis, we compared evolutionary patterns of organelle-targeted genes when *B. nigra* (BB) acted as the paternal progenitor in *B. juncea* (AABB) versus when *B. nigra* (BB) acted as the maternal progenitor in *B. carinata* (BBCC). We found that plastid-targeted genes experience selection to match plastid genomes, but there is no obvious corresponding signal in mitochondria-targeted genes from these 2 separately formed allopolyploids. Notably, selection from plastid genomes always reduced the retention rate of plastid-targeted genes encoded by the B subgenome, regardless of whether *B. nigra* (BB) was contributed from the paternal or maternal progenitor in *B. juncea* (AABB) and *B. carinata* (BBCC). Additionally, the nuclear-encoded subunits of mitochondrial complex III, which are localized to the inner mitochondrial membrane, have undergone exceptionally few gene conversion events compared to subunits localized to the mitochondrial matrix, suggesting that the structure of complexes affects the strength of cytonuclear interaction. Together, our study highlights an important role for subgenome dominance in allopolyploid genome evolution, even in genes that depend on position in the structure of multisubunit complexes.

## Results

### Phylogenetic Analysis of the *Brassica* Triangle of U Based on Organelle Genomes

To confirm the cytoplasmic donor of the selected allopolyploids, the plastid and mitochondrial protein-coding genes were used to infer maximum likelihood phylogenetic trees. In a concatenation of plastid-encoded protein-coding genes, *B. nigra* (BB) and *B. rapa* (AA) + *B. oleracea* (CC) formed 2 well-supported clades (Fig. 1b). *Brassica nigra* (BB) and *B. carinata* (BBCC) appeared to form a monophyletic clade, supporting the hypothesis that *B. nigra* (BB) was the cytoplasmic donor (maternal) of *B. carinata* (BBCC). *Brassica rapa* (AA) and *B. juncea* (AABB) also formed a monophyletic clade, supporting *B. rapa* (AA) as the cytoplasmic donor (maternal) of *B. juncea* (AABB). In contrast, the clade contained *B. napus* cultivar ‘Darmor-*bzh*’ (AACC_Darmor-bzh) and *B. napus* cultivar ‘ZS11’ (AACC_ZS11), which was sister to *B. rapa* (AA), *B. oleracea* (CC), *B. juncea* (AABB), and *B. napus* cultivar ‘No2127’ (AACC_No1127), indicating that the cytoplasmic donor of *B. napus* cultivar ‘Darmor-*bzh*’ (AACC_Darmor-bzh) and *B. napus* cultivar ‘ZS11’ (AACC_ZS11) may remain ambiguous. When *B. napus* (AACC) was removed from the analysis, the plastid and mitochondrial phylogenetic relationships were clustered into 3 100% supported monophyletic groups, including *B. nigra* (BB) + *B. carinata* (BBCC), *B. oleracea* (CC) and *B. rapa* (AA) + *B. juncea* (AABB), which strongly supported *B. nigra* (BB) as the cytoplasmic donor (maternal) of *B. carinata* (BBCC) and *B. rapa* (AA) as the cytoplasmic donor (maternal) of *B. juncea* (AABB; Fig. 1c and d).

We also identified the variants in protein-coding genes of organelle genomes between allopolyploids and their parents. All of the plastid and mitochondrial protein-coding genes of the 2 varieties of *B. juncea* (AABB) were identical to those of *B. rapa* (AA), including 486 synonymous sites and 358 nonsynonymous sites in the plastid and 13 synonymous sites and 30 nonsynonymous sites in the mitochondria, which was also strongly supported by the fact that *B. rapa* (AA) was the cytoplasmic donor (maternal) of *B. juncea* (AABB; Table 1). Similarly, the majority of variants present in the organelle genomes of *B. carinata* (BBCC) were identical to *B. nigra* (BB), including 503 synonymous sites, 382 nonsynonymous sites, and 14 private variants in the plastid, as well as 12 synonymous sites and 31 nonsynonymous sites in the mitochondria, which supported *B. nigra* (BB) as the cytoplasmic donor (maternal) of *B. carinata* (BBCC). However, nearly half of the variants were private sites in *B. napus* cultivar ‘Darmor-*bzh*’ (63), *B. napus* cultivar ‘ZS11’ (63), and *B. napus* cultivar ‘No2127’ (AACC_No1127; 66); 66 sites were identical to *B. oleracea* (CC; including 33 synonymous sites and 33 nonsynonymous sites); and only 5 sites were identical to *B. rapa* (AA; including 2 synonymous sites and 3 nonsynonymous sites). These results also supported that *B. napus* (AACC) has a complex history.

**Table 1:** Variants in organelle genomes of the 6 species in the *Brassica* triangle of U

	Type	AABB_tumida		AABB_varuna		BBCC		AACC_Darmor-bzh		AACC_ZS11		AACC_No2127	
		pt	mt	pt	mt	pt	mt	pt	mt	pt	mt	pt	mt
A-like	Synonymous	486	13	486	13	0	0	2	0	2	0	2	1
	Nonsynonymous	358	30	358	30	0	0	3	1	3	1	3	1
B-like	Synonymous	0	0	0	0	497	12	0	0	0	0	0	0
	Nonsynonymous	0	0	0	0	374	31	0	0	0	0	0	0
C-like	Synonymous	0	0	0	0	0	0	33	1	33	1	33	0
	Nonsynonymous	0	0	0	0	0	0	33	0	33	0	33	0
Private		0	0	0	0	14	0	63	0	63	0	66	0
Total	Synonymous	486	13	486	13	503	12	71	1	71	1	57	1
	Nonsynonymous	358	30	358	30	382	31	63	1	63	1	66	1
	Sum	844	43	844	43	885	43	134	2	134	2	134	2

A-like indicates that the nucleotide type of variants in alloploids is identical to *B. rapa* (AA); B-like indicates that the nucleotide type of variants in alloploids is identical to *B. nigra* (BB); and C-like indicates that the nucleotide type of variants in alloploids is identical to *B. oleracea* (CC). The pt indicates the number of variants in plastid protein-coding genes, while mt indicates the number of variants in mitochondrial protein-coding genes.

### Gene Retention Bias across Functional Categories in Allopolyploids

**Fig. 2.**
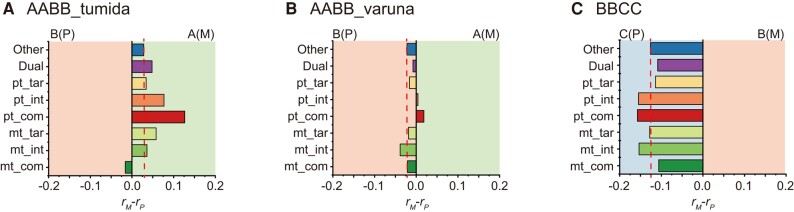
Gene classification and retention biased across the subgenomes in 3 allotetraploids. a) *B. juncea* var. *tumida* (AABB_tumida). b) *B. juncea* var. varuna (AABB_varuna). c) *B. carinata* (BBCC). “P” and “M” in brackets indicate paternal and maternal subgenomes in allotetraploids, respectively. The gene retention bias was calculated by the proportion of maternal subgenome minus that of paternal subgenome (*r*_M_-*r*_P_). The dot line indicates the retention ratio of nonorganelle-targeted genes in each allotetraploid. “Other” indicated nontargeted genes; “Dual” indicated dual-targeted genes; “pt_tar” and “mt_tar” indicated plastid- and mitochondria-targeted without interacting genes; “pt_int” and “mt_int” indicated plastid- and mitochondria-interacting without complex genes; and “pt_com” and “mt_com” indicated genes encoded plastid and mitochondria enzyme complexes.

According to whether the products encoded by nuclear genes transported to organelles, they are categorized into nontargeted genes (“Other”) and organelle-targeted genes. The latter can be further subdivided into 7 subcategories. Nuclear genes, whose products jointly encode chimeric enzyme complexes with organelle genes, fall into 2 subcategories: plastid (“pt_com”) and mitochondria enzyme complexes (“mt_com”). These genes directly interact with organelles, with the strongest interactive strength. Nuclear gene products that participate in organelle genome replication, transcription, and translation but are not incorporated into chimeric enzyme complexes are classified as plastid- (“pt_int”) and mitochondria-interacting without complex (“mt_int”) genes, with intermediate interactive strength. Nuclear gene products transported to the organelles but not directly interacting with organelle gene products are referred to as plastid- (“pt_tar”) and mitochondria-targeted without interacting (“mt_tar”) genes. Thus, they have the weakest interactive strength. The last subcategory involves gene products transported into both plastids and mitochondria, referred to as dual-targeted genes (“Dual”). The number of genes encoded by each subgenome in allopolyploids was smaller than that in corresponding diploid parents across all functional categories, and the number of genes encoded by different subgenomes in the same allopolyploids was different (supplementary tables S1 and S2 and fig. S1, Supplementary Material online). Therefore, we took nontargeted genes as controls to evaluate whether cytonuclear interaction affected the gene retention rate across functional categories. In *B. juncea* var. *tumida* (AABB_tumida), all functional categories, with the exception of mitochondria enzyme complexes (mt_com), tended to retain maternal copy (A subgenome). With the enhancement of cytonuclear interactions, plastid-targeted genes were more preferred to preserve maternal copy, while mt_tar and mt_int also tended to maintain more maternal copy (Fig. 2a; supplementary fig. S1, Supplementary Material online). The nontargeted genes encoded by the paternal subgenome (B) were more likely to be retained than those in the maternal subgenome of *B. juncea* var. *varuna* (AABB_varuna). With the enhancement of cytonuclear interactions, the retention bias of plastid-targeted genes transformed from the paternal subgenome to the maternal subgenome (Fig. 2b; supplementary table S1 and fig. S1, Supplementary Material online). In contrast, all functional categories were biased to retain the copy encoded by the paternal subgenome (C subgenome) in *B. carinata* (BBCC; Fig. 2c; supplementary table S2 and fig. S1, Supplementary Material online). Unfortunately, no significant differences in retention bias were observed between nontargeted genes and organelle-targeted genes (supplementary table S3, Supplementary Material online).

### Divergence of Organelle-Targeted Genes across Subgenomes in Allopolyploids

**Fig. 3.**
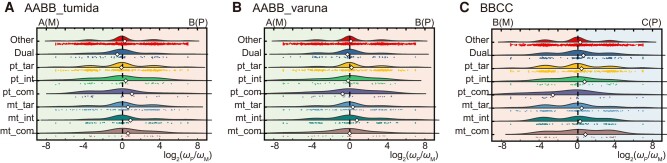
Genome-wide bias in *ω* (*d_N_*/*d_S_*) between the paternal and maternal subgenomes in allotetraploid species. a) *B. juncea* var. *tumida* (AABB_tumida). b) *B. juncea* var. *varuna* (AABB_varuna). c) *B. carinata* (BBCC). “P” and “M” in brackets indicate paternal and maternal subgenomes in allotetraploids, respectively. Diamond indicates the log_2_-transformed ratio of evolutionary rate between subgenomes of each functional category concatenated matrix. “Other” indicated nontargeted genes; “Dual” indicated dual-targeted genes; “pt_tar” and “mt_tar” indicated plastid- and mitochondria-targeted noninteracting genes, “pt_int” and “mt_int” indicated plastid- and mitochondria-interacting noncomplex genes; and “pt_com” and “mt_com” indicated genes encoded plastid and mitochondria enzyme complexes.

We calculated the evolutionary rate (i.e. *d_N_*/*d_S_*) of the single-gene matrix and the concatenated matrix of each functional category encoded by different subgenomes. Firstly, we found that the evolutionary rate of the concatenated matrix of all functional categories except mt_int was lower than that of nontargeted genes in all 3 selected allopolyploids (supplementary table S4, Supplementary Material online). In particular, the evolutionary rates of pt_com and mt_com were the lowest. These results suggested that the evolution of organelle-targeted genes may be constrained, and pt_com and mt_com may be subject to the strongest selection due to direct interaction with products encoded by organelle genomes, or that they were really important complexes. Furthermore, to detect whether organelle-targeted genes encoded by different subgenomes were affected by cytonuclear interaction, we also compared the differences in evolutionary rate between the paternal and maternal subgenomes across different functional categories in concatenated matrixes and single-gene matrixes (Fig. 3; supplementary table S4, Supplementary Material online). The log_2_-transformed ratio between *ω*_P_ and *ω*_M_ of the concatenated matrix of nontargeted genes was close to 0, while the distribution of the log_2_-transformed ratio between *ω*_P_ and *ω*_M_ of the single nontargeted gene matrix was also close to 0, indicating that the divergence and the number of divergent gene between paternal and maternal subgenomes were similar. In *B. juncea* var. *tumida* (AABB_tumida), the log_2_-transformed ratio between *ω*_P_ and *ω*_M_ of pt_tar was close to 0, the log_2_-transformed ratio between *ω*_P_ and *ω*_M_ of pt_int and pt_com was slightly larger than that of nontargeted genes, and the log_2_-transformed ratio of pt_com was larger than that of pt_int (Fig. 3a). The log_2_-transformed ratios between *ω*_P_ and *ω*_M_ of mt_tar, mt_int, and mt_com were larger than those of nontargeted genes. The pattern of plastid-targeted genes of *B. juncea* var. *varuna* (AABB_varuna) was contrary to that of *B. juncea* var. *tumida* (AABB_tumida), but the pattern of mitochondria-targeted genes was similar to *B. juncea* var. *tumida* (AABB_tumida; Fig. 3b). Only pt_tar of *B. juncea* var. *varuna* (AABB_varuna) exhibited a significant difference in *ω* between nontargeted genes and organelle-targeted genes (supplementary table S5, Supplementary Material online). Compared with *B. juncea* (AABB), the log_2_-transformed ratio between *ω*_P_ and *ω*_M_ of pt_tar, mt_tar, and mt_int was around the value of nontargeted genes in *B. carinata* (BBCC), but the log_2_-transformed ratio of pt_com was much smaller than that of nontargeted genes (Fig. 3c). The log_2_-transformed ratio of mt_com was larger than that of nontargeted genes, indicating the evolutionary rate of plastid–complex genes encoded by the maternal genome was increased, while that of mitochondria–complex genes encoded by the paternal subgenome was increased.

To detect whether cytonuclear interactions contribute to fixing the sequences of paternal homoeologs converted to maternal homoeologs by gene conversion, we inferred the gene conversion based on the principle that the similarity between orthologous genes without gene conversion was larger than that of homologous genes according to the previous study (Wei et al. 2021). We detected 66, 6, and 84 gene conversion events in *B. juncea* var. *tumida* (AABB_tumida), *B. juncea* var. *varuna* (AABB_varuna), and *B. carinata* (BBCC), respectively (Table 2). In *B. juncea* var. *tumida* (AABB_tumida), the majority of the gene conversion events occurred in nontargeted genes (74%). Among these, the substitution of sequence from maternal homoeologs to paternal homoeologs [A(M)-to-B(P)] accounted for 18, while the reciprocal substitutions [B(P)-to-A(M)] accounted for 31, resulting in an approximate ratio of 58%. Only pt_tar, pt_com, and mt_int occurred gene conversions with the ratio between A(M)-to-B(P) and B(P)-to-A(M) being 80%, 100%, and 50%, respectively. Notably, the sequences of maternal homoeologs were more likely to be fixed than that of nontargeted genes (Table 2; supplementary table S6, Supplementary Material online). In *B. juncea* var. *varuna* (AABB_varuna), there were only 2 gene conversions that occurred in nontargeted genes (33%), and they were A(M)-to-B(P) conversions. In contrast, only 1 gene conversion occurred in dual-targeted genes and it was B(P)-to-A(M). Additionally, 2 gene conversions occurred in pt_tar, including 1 A(M)-to-B(P) and 1 B(P)-to-A(M) conversion, whereas a single gene conversion occurred in mt_com, characterized by an A(M)-to-B(P) conversion (Table 2; supplementary table S7, Supplementary Material online). Similar to the pattern observed in *B. juncea* var. *tumida* (AABB_tumida), the majority of gene conversions in *B. carinata* (BBCC) also occurred in nontargeted genes (83%). Among these, the replacement of paternal homoeologs by maternal homoeologs [B(M)-to-C(P)] was 32, and the reciprocal replacement [C(P)-to-B(M)] was 38, resulting in an approximate ratio of 84%. Notably, gene conversions were limited to dual, pt_tar, and mt_tar, with B(M)-to-C(P) to C(P)-to-B(M) ratios of 0%, 33%, and 600%, respectively. Collectively, organelle-targeted genes retained more B(M)-to-C(P) conversions (175%; Table 2; supplementary table S8, Supplementary Material online). As the region affected by gene conversion was relatively small, it may not be enough to cause similarity differences that can be detected, so this method may underestimate the frequency of gene conversion events.

**Table 2:** Number of gene conversions between the paternal and maternal homoeologs in all allotetraploid species at genome-wide level

	AABB_tumida		AABB_varuna		BBCC	
Functional category	A(M)-to-B(P)	B(P)-to-A(M)	A(M)-to-B(P)	B(P)-to-A(M)	B(M)-to-C(P)	C(P)-to-B(M)
Other	18	31	2	0	32	38
Dual-targeted (dual)	0	3	0	1	0	3
Plastid-targeted genes	5	6	1	1	1	3
Plastid-targeted without interacting (pt_tar)	4	5	1	1	1	3
Plastid-interacted without complexes (pt_int)	0	0	0	0	0	0
Plastid enzyme complexes (pt_com)	1	1	0	0	0	0
Mitochondria-targeted genes	1	2	1	0	6	1
Mitochondria-targeted without interacting (mt_tar)	0	0	0	0	6	1
Mitochondria-interacted without complexes (mt_int)	1	2	0	0	0	0
Mitochondria enzyme complexes (mt_com)	0	0	1	0	0	0
Total	24	42	4	2	39	45

A(M)-to-B(P) indicates the sequence of B was replaced by A, while B(P)-to-A(M) indicates the sequence of A was replaced by B. B(M)-to-C(P) indicates the sequence of C was replaced by B, while C(P)-to-B(M) indicates the sequence of B was replaced by C.

### Homoeolog Expression Bias of Organelle-Targeted Genes in Allopolyploids

**Fig. 4.**
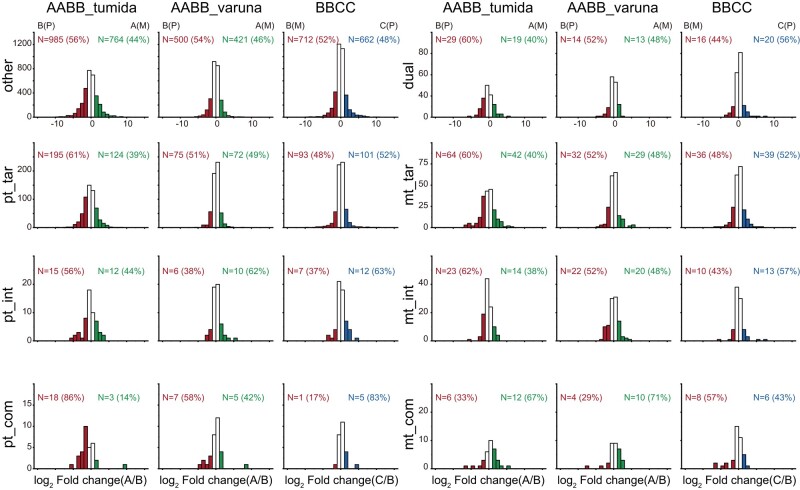
Genome-wide expression divergence between the paternal and maternal subgenomes in allotetraploid species. P and M in brackets indicate paternal and maternal subgenomes in allotetraploids, respectively. The *x* axis represents the log_2_-transformed ratio of expression difference between subgenomes. “Other” indicated nontargeted genes; “Dual” indicated dual-targeted genes; “pt_tar” and “mt_tar” indicated plastid- and mitochondria-targeted noninteracting genes; “pt_int” and “mt_int” indicated plastid- and mitochondria-interacting noncomplex genes; and “pt_com” and “mt_com” indicated genes encoded plastid and mitochondria enzyme complexes.

To investigate the effect of cytonuclear interaction on the expression of homoeologs, we downloaded serval RNA-seq data from all 3 allopolyploids, including 3 replicates of mixed RNA-seq from *B. juncea* var. *tumida* (AABB_tumida), 3 replicates of several tissues from *B. carinata* (BBCC), and 1 from *B. juncea* var. *varuna* (AABB_varuna). Thus, we could compare expression differences between subgenomes at a transcriptome-wide level among different tissues. Among the 3 allopolyploids, the number of the highly expressed nontargeted genes encoded by the B subgenome was larger than the other subgenome (Fig. 4). There were more highly expressed plastid-targeted genes encoded by the paternal subgenome in *B. juncea* var. *tumida* (AABB_tumida), and the proportion of highly expressed genes encoded by the paternal genome of pt_tar and pt_com was larger than that of nontargeted genes. However, only pt_com exhibited a significant difference between nontargeted genes and organelle-targeted genes (supplementary table S9, Supplementary Material online). We also observed that the highly expressed paternal genes of pt_tar and pt_com were more than maternal homoeologs in *B. juncea* var. *varuna* (AABB_varuna). Although the number of highly expressed nontargeted genes encoded by the B subgenome in *B. carinata* (BBCC) was higher than that of the C subgenome, the number of highly expressed plastid-targeted genes encoded by the C subgenome was more, and this preference was strengthened as the intensity of cytonuclear interaction increased. The biased pattern between paternal and maternal subgenomes of differentially expressed genes of dual, mt_tar, and mt_int was similar to that of nontargeted genes in 3 allopolyploids, but the number of mt_com with highly expressed genes encoded by the maternal subgenome was significantly higher than that encoded by the paternal subgenome.

We analyzed the RNA-seq data of 6 tissues (including root, stem, leaf, flower, silique, and seed) with 3 replicates of *B. carinata* (BBCC) to detect whether cytonuclear interactions affect homoeolog expression bias across different tissues. We found that the proportion of highly expressed nontargeted genes encoded by the maternal subgenome (B) was larger than that of the paternal subgenome (C), except stem and seed (supplementary fig. S2, Supplementary Material online). In contrast, the paternal subgenome encoded more highly expressed plastid-targeted genes than the maternal subgenome in leaf, flower, silique, and seed, and this preference was reinforced in leaf, flower, and silique with the intension of cytonuclear interactions. In most tissues, there were more highly expressed mt_tar and mt_int genes encoded by the paternal subgenome than those of the maternal subgenome, but more highly expressed mt_com genes were encoded by the maternal subgenome, except in the root.

### Evolution of Organelle and Nuclear Genes Encoded RuBisCo in Allopolyploids

**Fig. 5.**
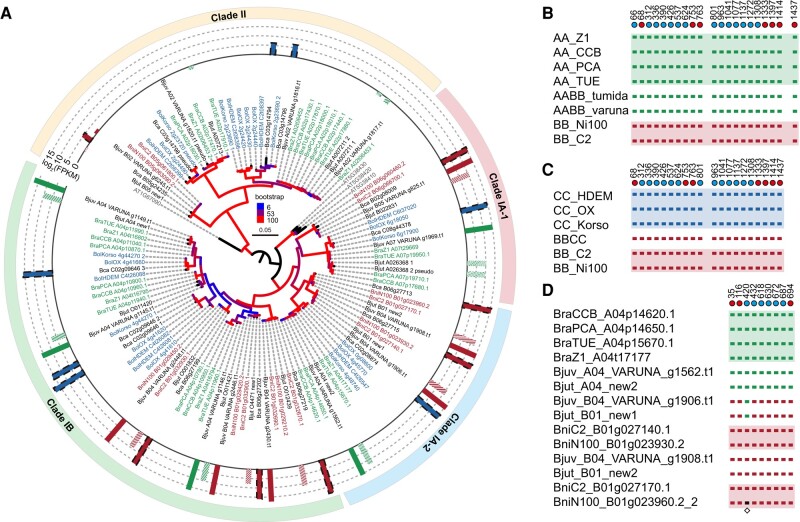
Maximum likelihood phylogeny and variants of genes encoded RuBisCo in studied genomes/subgenomes. a) Maximum likelihood phylogeny and expression level of *rbcS* gene family. The species names in green indicate *B. rapa* (AA), red indicates *B. nigra* (BB), and blue indicates *B. oleracea* (CC). The bar chart shows the expression abundance of genes encoded by different subgenomes in allopolyploids, with the A subgenome in green, the B subgenome in red, the C subgenome in blue, *B. juncea* var. *tumida* (AABB_tumida) with slash lines, and *B. carinata* (BBCC) with the dot sides. b and c) Variants of *rbcL* in allopolyploids and their parents. d) Variants and gene conversion of *rbcS* in allopolyploids and their parents. The green shade indicates *B. rapa* (AA), red indicates *B. nigra* (BB), and blue indicates *B oleracea* (CC). The red dot indicates a nonsynonymous genomic-specific site, while the blue dot indicates synonymous genomic-specific sites. The hollow diamond indicates the gene conversion from paternal sequence to maternal homoeologs.

RuBisCo catalyzed the first major carbon fixation in the Calvin cycle during photosynthesis, is the key enzyme that determines the rate of carbon assimilation in photosynthesis, and is also the most studied complex in cytonuclear interactions. It consisted of 8 large subunits encoded by the plastid gene (*rbcL*) and 8 small subunits encoded by a nuclear gene family (*rbcS*; Pottier et al. 2018). There were 21 (including 14 synonymous sites and 7 nonsynonymous sites) variants between *B. rapa* (AA) and *B. nigra* (BB) and 20 (including 13 synonymous sites and 7 nonsynonymous sites) variants between *B. nigra* (BB) and *B. oleracea* (CC) in *rbcL* (Table 3 and Fig. 5b and c; supplementary fig. S3, Supplementary Material online). Notably, all variants were completely identical to maternal progenitor in allopolyploids. However, there were no variants distributed in the active sites or the binding site between LSU and SSU. The *rbcS* gene family contained 4 genes in *Arabidopsis*, including 1 gene distributed on chromosome (Chr) 1 and 3 genes distributed on Chr5. In contrast, the *rbcS* gene family consisted of 8 members in diploid species of *Brassica*, except for *B. oleracea* var. *capitata* (CC_OX), which had 2 additional members, and *B. oleracea* var. *botrytis* (CC_Korso) and *B. nigra* var. *N100* (BB), which had 1 less member (supplementary table S10 and fig. S4a, Supplementary Material online). The B subgenomes of *B. juncea* var. *varuna* (AABB) and *B. carinata* (BBCC) contained 7 members of *rbcS*, whereas in *B. juncea* var. *tumida* (AABB), 1 member was lost. However, the number of *rbcS* in the A and C subgenomes remained the same as diploids, except for the A subgenome of *B. juncea* var. *tumida* (AABB_tumida), which had 1 additional member compared with diploids. The members of *rbcS* were distributed on Chr2, Chr4, and Chr7 in the A genomes/subgenomes; Chr1, Chr5, and Chr6 in the B genomes/subgenomes; and Chr2, Chr4, and Chr6 in the C genomes/subgenomes in *Brassica* (supplementary fig. S5, Supplementary Material online).

**Table 3:** Variants and conversions in genes encoded RuBisCo complex in all allotetraploids and its diploid parents

			A-B	AABB_tumida				AABB_varuna				C-B	BBCC			
Gene	Subunit	Clade	genome-specific SNPs	A(M)-to-B(P)		B(P)-to-A(M)		A(M)-to-B(P)		B(P)-to-A(M)		genome-specific SNPs	B(M)-to-C(P)		C(P)-to-B(M)	
				S	N	S	N	S	N	S	N		S	N	S	N
*rbcL*	LSU		21	NA	NA	NA	NA	NA	NA	NA	NA	20	NA	NA	NA	NA
*rbcS*	SSU	IA-1	39	0	0	0	0	0	0	0	0	42	0	0	0	0
		IA-2	8	0	0	0	0	0	0	0	0	6	0	0	0	0
		IB	NA	NA	NA	NA	NA	NA	NA	NA	NA	NA	NA	NA	NA	NA
		II	25	1	0	0	0	0	0	0	0	25	0	0	0	0
Total			72	1	0	0	0	0	0	0	0	73	0	0	0	0

A(M)-to-B(P) indicates the sequence of B was replaced by A, while B(P)-to-A(M) indicates the sequence of A was replaced by B. B(M)-to-C(P) indicates the sequence of C was replaced by B, while C(P)-to-B(M) indicates the sequence of B was replaced by C.

S, synonymous substitution; N, nonsynonymous substitution; NA, not available.

We also reconstructed the phylogeny of the *rbcS* gene family, and it could be divided into 2 subfamilies (clade I and clade II; Fig. 5a). The clade I contained 3 members of *Arabidopsis* that formed a monophyly, whereas the members of *Brassica* could be further divided into 2 subclades (clade IA and clade IB). The relationship between members of clade IA-1 was consistent with the species tree, and the members encoded by B subgenomes underwent tandem duplication after B diverged with A and C in clade IA-2. Clade IB consisted of the adjacent genes distributed in Chr4 in A genomes/subgenomes, Chr1 in B genomes/subgenomes, and Chr4 in C subgenomes/genomes, but the cluster was not consistent with the species tree. The clade II contained only 1 member of *Arabidopsis*, and the members of the A and C genomes/subgenomes underwent a duplication event before the divergence of A and C. Therefore, we identified genome-specific single nucleotide polymorphisms (SNPs) for each clade, excluding clade IB, to detect gene conversions occurring between subgenomes. There were 39, 8, and 25 genome-specific sites between A and B genomes/subgenomes and 42, 6, and 25 genome-specific sites between B and C genomes/subgenomes in clade IA-1, clade IA-2, and clade II, respectively (Table 3; supplementary fig. S6, Supplementary Material online). Notably, there was only 1 synonymous conversion that occurred between the A and B subgenomes in clade II from *B. juncea* var. *tumida* (AABB_tumida), which converted paternal homoeolog to maternal homoeolog (Fig. 5d).

We quantified the expression abundance of all members of *rbcS* in allopolyploids to infer whether cytonuclear interactions affected the homoeolog expression bias. The expression levels of the homoeologs encoded by different subgenomes in clade I were similar; the expression abundance of each copy of duplicated genes encoded by B subgenomes in clade IA-2 was consistent with that of homoeologs encoded by the other subgenome, and even the expression levels of newly originated pseudogenes in the A subgenome of *B. juncea* var. *tumida* (AABB_tumida) were also similar to that of the B homoeolog and the original copy in the A subgenome (Fig. 5a). In clade II, the expression abundance of homoeologs of *B. juncea* (AABB) was at a very low level, and the expression abundance of homoeologs encoded by the 2 subgenomes of *B. carinata* (BBCC) was similar, but much lower than that of clade I.

### Evolution of Organelle and Nuclear Genes Encoded Mitochondrial Complex III in Allopolyploids

**Fig. 6.**
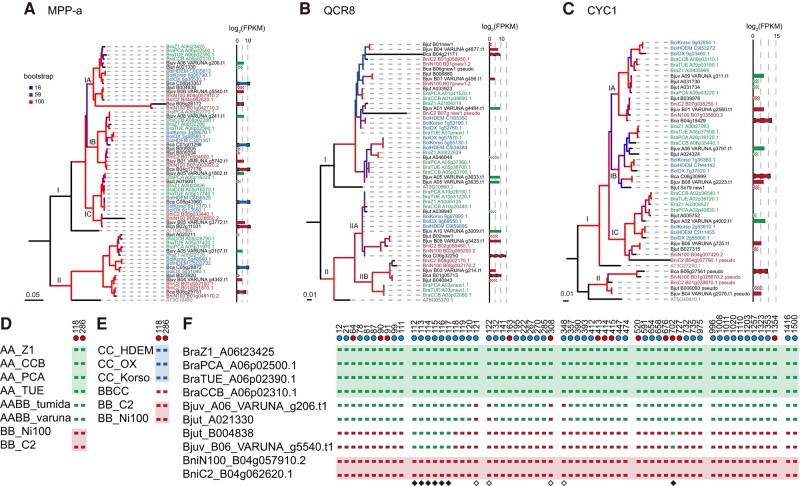
Maximum likelihood phylogenies and variants of genes encoded mitochondrial complex III in studied genomes/subgenomes. a) Maximum likelihood phylogeny of the gene family encoded by the C-terminal subunit (MPP-α). b) Maximum likelihood phylogeny of the gene family encoded by the transmembrane helices (QCR8). c) Maximum likelihood phylogeny of the gene family by encoded the N-terminal subunit (CYC1). The species names in green indicate *B. rapa* (AA), red indicates *B. nigra* (BB), and blue indicates *B oleracea* (CC). The bar chart shows the expression abundance of genes encoded by different subgenomes in allopolyploids, with the A subgenome in green, the B subgenome in red, the C subgenome in blue, *B. juncea* var. *tumida* (AABB_tumida) with slash lines, and *B. carinata* (BBCC) with the dot sides. d and e) Variants of *cob* in allopolyploids and their parents. f) Variants and gene conversion of clade IA-1 of the gene family encoded by the C-terminal subunit (MPP-α) in *B. juncea* (AABB) and its parents. The green shade indicates *B. rapa* (AA), red indicates *B. nigra* (BB), and blue indicates *B oleracea* (CC). The red dot indicates a nonsynonymous genomic-specific site, while the blue dot indicates synonymous genomic-specific sites. The solid diamond indicates the gene conversion from maternal sequence to paternal homoeologs, and the hollow diamond indicates the gene conversion in the opposite direction.

Mitochondrial complex III (cytochrome bc1 complex or ubiquinol: cytochrome c oxidoreductase) transfers electrons from ubiquinone to cytochrome c. It is one of the critical complexes in respiration and consists of 1 mitochondrial gene (*cob*) and 10 nuclear gene families in *Arabidopsis*. Based on the crystal structure, the complex III contained 13 transmembrane helices, including 8 from COB (*cob*) and 1 from CYC1, UCR1, QCR8, QCR9, and QCR10. The MPP domain was consisted of 2 MPP-ɑ/β heterodimers, which extended into the matrix, and the C-terminus of CYC1. The entire QCR6 and UCR1 extended into the intermembrane space in *Vigna radiata* (Maldonado et al. 2021), which provided an opportunity to compare the impact of cytonuclear interactions on subunits at different spatial positions within complex III. The sequence of *cob* in *B. rapa* (AA) and *B. oleracea* (CC) was identical, but there were only 2 variants in *cob* between *B. nigra* (BB) and *B. rapa* (AA) and *B. oleracea* (CC), both of which were nonsynonymous (Table 4 and Fig. 6d and e; supplementary fig. S7, Supplementary Material online). Notably, the variants in allopolyploids were completely identical to those of the maternal parent.

**Table 4:** Variants and conversions in genes encoded mitochondrial complex III in all allotetraploids

		A-B	AABB_tumida				AABB_varuna				C-B	BBCC			
Gene	Subunit	genome-specific SNPs	A(M)-to-B(P)		B(P)-to-A(M)		A(M)-to-B(P)		B(P)-to-A(M)		genome-specific SNPs	B(M)-to-C(P)		C(P)-to-B(M)	
			S	N	S	N	S	N	S	N		S	N	S	N
*Cob*		2	NA	NA	NA	NA	NA	NA	NA	NA	2	NA	NA	NA	NA
*At1g51980_At3g16480*	MPP-α	229	7	2	4	1	7	3	3	2	252	8	9	0	1
*At3g02090*	MPP-β	194	6	0	2	2	6	0	1	0	215	8	2	5	0
*At4g32470_At5g25450*	QCR7	35	0	0	0	0	0	0	0	0	40	0	0	1	0
*At2g40765*	QCR10	NA	NA	NA	NA	NA	NA	NA	NA	NA	NA	NA	NA	NA	NA
*At3g52730*	QCR9	8	0	0	0	0	0	0	0	0	9	0	0	0	0
*At3g10860_At5g05370*	QCR8	19	1	1	0	0	6	4	1	0	8	1	0	2	0
*At5g13430_At5g13440*	UCR1	203	21	4	1	1	25	3	5	1	259	27	0	27	2
*At1g15120_At2g01090*	QCR6	29	4	0	0	0	3	0	0	0	34	0	0	3	1
*At3g27240_At5g40810*	CYC1	45	1	0	0	0	1	0	1	0	28	0	0	2	0
Total		764	40	7	7	4	48	10	11	3	847	44	11	38	4

QCR8, QCR9, and QCR10 are located in the inner membrane. A(M)-to-B(P) indicates the sequence of B was replaced by A, while B(P)-to-A(M) indicates the sequence of A was replaced by B. B(M)-to-C(P) indicates the sequence of C was replaced by B, while C(P)-to-B(M) indicates the sequence of B was replaced by C.

S, synonymous substitution; N, nonsynonymous substitution; NA, not available.

There were 0 to 10 members of each gene family that encoded complex III in the studied subgenomes/genomes of *Brassica*; most gene families expanded after diverging with *Arabidopsis*, except for QCR10 (supplementary table S11, Supplementary Material online). Those genes were distributed on multiple chromosomes in allopolyploids corresponding to those in diploid genomes (supplementary fig. S8, Supplementary Material online). Correspondingly, the phylogenetic tree of each gene family could be divided into several clades, most of which matched the species tree (Fig. 6a to c; supplementary fig. S9, Supplementary Material online), so we identified gene conversion events based on genome/subgenome specific variants in those clades. There are 8 to 229 genome-specific sites between A and B genomes/subgenomes and 8 to 259 genome-specific sites between B and C genomes/subgenomes (Table 4; supplementary table S12, Supplementary Material online). In total, we identified 47 maternally biased gene conversions (40 synonymous conversions and 7 nonsynonymous conversions) and 11 (7 synonymous conversions and 4 nonsynonymous conversions) in the opposite direction in *B. juncea* var. *tumida* (AABB_tumida); 58 maternally biased gene conversions (48 synonymous conversions and 10 nonsynonymous conversions) and 14 (11 synonymous conversions and 3 nonsynonymous conversions) in the opposite direction in *B. juncea* var. *varuna* (AABB_varuna); and 55 maternally biased gene conversions (44 synonymous conversions and 11 nonsynonymous conversions) and 42 (38 synonymous conversions and 4 nonsynonymous conversions) in the opposite direction in *B. carinata* (BBCC; Table 4 and Fig. 6f; supplementary table S12 and figs. S10 to S17, Supplementary Material online). Notably, both the number of genome-specific sites and gene conversions of the subunits located in the mitochondrial inner membrane (QCR8, QCR9, and QCR10) were less than those located in the mitochondrial matrix. The expression levels of homoeologs were close, and the differences in expression levels between different subgenomes are often caused by the differences in the number of members (Fig. 6a to c; supplementary fig. S9a to i, Supplementary Material online).

## Discussion

The 3 allotetraploids in the U triangle of *Brassica* originated from the hybridization between 2 of the 3 diploids for a long time period, especially *B. nigra* (BB), which acts as the paternal progenitor of *B. juncea* (AABB) and maternal progenitor of *B. carinata* (BBCC; Nagaharu 1935; Song et al. 2021), and their relationships were also confirmed based on organelle genomes in this study. As cytonuclear interactions cannot be observed in recently formed and synthetic allopolyploids, the U triangle of *Brassica* is a more suitable system to explore the relative magnitude of the effect of cytonuclear interactions and the subgenome dominance evolution of organelle-targeted genes. We investigated the retention preference, evolutionary rates, homoeologous exchanges, and homoeolog expression bias of organelle-targeted genes (including plastid- and mitochondria-targeted genes) and nontargeted genes at the genome-wide level and in typical complexes in 3 allopolyploids, following 3 primary conclusions. Firstly, our prominent finding was that selection from plastid genomes always reduced the retention rate of plastid-targeted genes encoded by the B subgenome, regardless of whether the *B. nigra* (BB) subgenome was contributed to the paternal or maternal progenitor, suggesting that cytonuclear interactions and subgenome dominance co-shaped the evolutionary fate of organelle-targeted genes. Secondly, although plastids and mitochondria are natural controls in plants, which are uniparental inheritance, multicopy, and have a slower evolutionary rate than that of the nuclear genome, we found partial evidence that plastid-targeted genes experience selection to match plastid genomes, but no obvious corresponding signal in mitochondria-targeted genes in these 2 separately formed allopolyploids. Thirdly, we also found that the subunits of mitochondrial complex III located in the inner mitochondrial membrane had undergone exceptionally few gene conversions compared to those located in the mitochondrial matrix, indicating the structure of the complex affected the strength of cytonuclear interactions.

### Evolutionary Trajectories of Organelle-Targeted Genes Corresponding to B Subgenome as Paternal and Maternal Parent in Allopolyploids

The most prominent result among our data was that the B subgenome contained fewer plastid-targeted genes with the interactive strength between plastid and nuclear genes in 3 alloploids. Consistently, the B subgenome also encoded fewer *rbcS* genes than that of the other subgenome. Those results were partially consistent with what was expected. Theoretically, as organellar genomes are usually inherited maternally, the subgenomes derived from the maternal progenitor are more suitable and more likely to be retained and functional in hybrids (Sharbrough et al. 2017; Camus et al. 2022). Our data confirmed that the retention bias of plastid-targeted genes in 2 varieties of *B. juncea* (AABB) was consistent with expectation, and this expectation was also observed in *Arabidopsis*, *Arachis*, *Gossypium*, and *Nicotiana* (Gong et al. 2012, 2014). However, the paternal-derived plastid-targeted genes in *B. carinata* (BBCC) had a greater tendency to retain, which was opposite to the expectation, suggesting that there were some potential factors that influenced the intersubgenome retention bias except cytonuclear interactions. The formation of this phenomenon may be similar to that of subgenome dominance in allopolyploids, where the gene retention rate and/or expression level of 1 subgenome were higher than those of the other subgenome (Cheng et al. 2018; Alger and Edger 2020; Roy 2021). Subgenome dominance was influenced by repetitive elements, small RNAs, epigenetic modifications, and cytonuclear incompatibility and had been observed in many allopolyploids, from resynthesized neoallopolyploids in *Mimulus* to ancient allopolyploids in *Arabidopsis*, *Zea*, and *Brassica* (Thomas et al. 2006; Schnable et al. 2011; Cheng et al. 2016; Edger et al. 2017; Kang et al. 2019). Previous studies had shown that the B subgenome contained more transposons, repetitive sequences, etc. (Kang et al. 2021; Yim et al. 2022), which may be one of the reasons why genes tended to be lost. Furthermore, it had been observed that organelle-targeted genes usually maintained multiple copies in young polyploids but reverted to single copy in ancient polyploids (Li et al. 2016). Given that, the allopolyploids in *Brassica* originated approximately 8,000 yr ago and were relatively young, suggesting the organelle-targeted genes in *Brassica* were ongoing to return to single copy. Consequently, the formation time of allopolyploids served as another reason. An alternative explanation for no statistically significant differences or only weak significance in retention rate between subgenomes could be attributed to cytonuclear interactions impacting only a restricted subset of genes, while the signals might have been diluted in genome-wide analysis (Sloan et al. 2024). Additionally, allopolyploid genome assembly posed challenges in subgenome phasing (Jia et al. 2022; Sun et al. 2022; Wang et al. 2023), potentially leading to errors in the identification of organelle-targeted genes encoded by distinct subgenomes. Although our genome-wide analysis could alleviate assembly and annotation errors, future advancements in sequencing and assembly technology will facilitate more accurate investigations into retention preferences among subgenomes.

We also examined the preference of gene conversion, revealing that *B. juncea* var. *tumida* (AABB_tumida) and *B. carinata* (BBCC) harbored more paternal homoeologous sequence in maternal homoeologs (P-to-M) than reciprocal conversion (M-to-P) at the whole-genome level. This preference was more obvious in plastid-targeted genes in *B. carinata* (BBCC). There was only 1 conversion site in the *rbcS* of *B. juncea* var. *tumida* (AABB_tumida), whereas there were more genome-specific sites transitioning from paternal type to maternal type in mitochondrial complex III of *B. juncea* (AABB) and *B. carinata* (BBCC). The conversion pattern of mitochondrial complex III was consistent with previous studies on RuBisCo in other plant lineages, which supported that selection from organelle genomes contributes to fixing the maternal region in the paternal homoeologous genes (Gong et al. 2012, 2014; Li et al. 2019). Alternately, as the method based on the sequence substitution rate was referred to as the principle of the previous studies, it was not sensitive enough to detect small gene conversion events at the whole-genome level, resulting in underestimating the frequency of gene conversion and misleading the bias of directions of gene conversion (Zhuang et al. 2019; Wei et al. 2021). There is still a need to improve identification accuracy, like gene families, at the whole-genome level.

Expression bias is another dimension of subgenome divergence (especially for organelle-targeted homoeologs) in allopolyploids. There were more highly expressed nontargeted genes encoded by the B subgenome in *B. juncea* (AABB) and *B. carinata* (BBCC), consistented with the previous studies (Yang et al. 2016; Song et al. 2021; Yim et al. 2022). The maternal (A) subgenome of *B. juncea* (AABB) contained more highly expressed plastid-targeted genes, which was consistent with the observations in *Arabidopsis*, *Arachis*, *Nicotiana*, and *Gossypium* (Gong et al. 2012, 2014). In contrast, the *B. juncea* var. *tumida* (AABB_tumida) and nontargeted genes and *B. carinata* (BBCC) exhibited reciprocal expression preferences. One possible explanation was insufficient time after polyploid speciation, a recently formed allopolyploid (*Tragopogon miscellus*) and 2 synthetic allopolyploids (*Oryza* and *Cucumis*), in which both parental *rbcS* copies were expressed in most populations and there was a different expression bias in different populations (Sehrish et al. 2015; Wang et al. 2017; Zhai et al. 2019). Since nontargeted genes also shown the expression bias of the paternal subgenome, the expression bias of organelle-targeted genes may be the same as that of nontargeted genes, and both were affected by subgenome characteristics, which was another possible explanation of expression bias (Yang et al. 2016; Alger and Edger 2020). Additionally, the impact of cytonuclear interactions on expression may vary across cell and developmental stages; however, mixed RNA-seq data could obscure the signal (Zhang et al. 2023; Sloan et al. 2024). Those results indicate that subgenome dominance and cytonuclear interactions jointly play roles in the evolution of organelle-targeted genes.

### Different Selective Pressures between Plastid and Mitochondria-Targeted Genes in Allopolyploids

Both plastids and mitochondria in plants are usually uniparental inheritance, multicopy, and have a slower evolution rate than that of the nuclear genome, which are the natural controls for investigating the pattern of cytonuclear interactions. However, the interactions between mitochondrion and nuclear genomes in plants are often neglected. In this study, we found that the plastid-targeted genes had reduced the retention rate of the B subgenome, while mitochondria-targeted genes were randomly retained across categories in the 3 allopolyploids. The B subgenome contained fewer members of the *rbcS* gene family than the other subgenome in 3 allopolyploids, but the B subgenome contained more members of gene families encoding complex III. Accordingly, the paternal subgenomes of *B. juncea* var. *tumida* (AABB_tumida) and *B. carinata* (BBCC) encoded more highly expressed plastid-targeted genes, but the expression pattern of mitochondria-targeted genes was more complex. The main possible explanation for this difference between mitochondria- and plastid-targeted genes was that the evolutionary rate of plastid genomes was higher than that of mitochondrial genome in land plants (Wolfe et al. 1987; Drouin et al. 2008). Correspondingly, we also found that the number of variants in plastid protein-coding genes was significantly higher than that in mitochondrial protein-coding genes between cytoplasmic donors, resulting in the selective pressure on organelle-targeted genes from plastid genomes being stronger than that from mitochondrial genomes. The other dimension of the main possible explanation was that both plastids and mitochondrial genomes undergo heteroplasmic sorting to fix variations among multiple copies within cells, and plastids had a faster sorting rate than that of mitochondrial genomes, often within a single generation (Broz et al. 2022). Therefore, the fixation of nuclear genome variations may compensate for plastid variations rapidly (Osada and Akashi 2012; Havird et al. 2015; Hill 2020; Zhang et al. 2022). Furthermore, we assumed that the interactive strength between organelle-targeted genes and organelles depended on whether they coencode complexes. However, certain studies had revealed that cytonuclear interactions, particularly those involving PPR genes—genes targeted to organelles and participating in the posttranscriptional modification of organelle genes—were more intense than those involving organelle ribosomes (Forsythe et al. 2020). Consequently, the retention bias of mitochondria-targeted genes appeared to be random in these 3 allopolyploids.

There was only 1 synonymous gene conversion that occurred in *rbcS* in *B. juncea* var. *tumida* (AABB_tumida), but there were 58 to 97 gene conversions in nuclear gene families encoded in mitochondrial complex III. In addition to the fact that the evolutionary and heteroplasmic sorting rate of plastids is higher than that of mitochondria, concerted evolution is also one of the reasons why gene conversion sites are less fixed in *rbcS*, because some members are located in adjacent regions of the chromosome (Meagher et al. 1989; Gong et al. 2014). Notably, the most gene conversions occurred in the subunits of complex III located in the matrix compared with the subunits located in the inner membrane. The subunits located in the inner membrane played an important role in the fixation of the complex and the electronic channel (Maldonado et al. 2021), so constraints on those subunits were stronger than those located in the matrix, resulting in fewer variants and gene conversion sites. Because there are many differences in physiology, evolution, and genetics between plastids and mitochondria, the factors that cause the differences between plastid and mitochondrial-targeted genes need to be studied in more groups.

## Materials and Methods

### Data Resource

Due to the 6 species in the triangle of U that are important vegetables and oil crops, all except *B. carinata* (BBCC), many chromosome-level genomes and related data have been published (Wu et al. 2022). To cover the genetic diversity of different species as much as possible, we selected 4 varieties for *B. rapa* (AA), 2 for *B. nigra* (BB), and 3 for *B. oleracea* (CC), respectively (Perumal et al. 2020; Cai et al. 2021; Guo et al. 2021). A vegetable-use variety *B. juncea* var. *tumida* and an oil-bearing variety *B. juncea* var. *varuna* were selected to represent *B. juncea* (AABB) allopolyploids (Yang et al. 2016; Paritosh et al. 2020), while the winter-type *B. napus* cultivar ‘Darmor-*bzh*’, semiwinter-type *B. napus* cultivar ‘ZS11’, and spring-type *B. napus* cultivar ‘No2127’ were used to represent *B. napus* (AACC) allopolyploids (Sun et al. 2017; Rousseau-Gueutin et al. 2020; Song et al. 2020). Only an individual of *B. carinata* was selected (Song et al. 2021). We selected the model plant *Arabidopsis thaliana* as an outgroup because it was the related genus of *Brassica* and the complete gene function annotation (CyMIRA; Forsythe et al. 2019). The information on assemblies, annotations, and sequencing data used in this study was provided in supplementary table S13, Supplementary Material online.

### Assembly, Annotation, and Analysis of Organelle Genomes

The resequencing data of each individual were downloaded from NCBI (https://www.ncbi.nlm.nih.gov/). FastQC v0.11.9 (https://www.bioinformatics.babraham.ac.uk/projects/fastqc/) and Trimmomatic v0.39 were applied to quality control (Bolger et al. 2014). Then, GetOrganelle v.1.7.5 was used to assemble plastid and mitochondrial genomes, and the mitochondrial assembly graphs of each individual were simplified to a single-circle molecule by Bandage v 0.8.1 (Wick et al. 2015; Jin et al. 2020). The PGA and BLASTn v2.11.0+ were employed to annotate plastid and mitochondrial genomes, respectively (Camacho et al. 2009; Qu et al. 2019). Both of them were checked manually by BioEdit v7.2.5 (Hall 1999).

To further verify the cytoplasmic donor of the allopolyploids, we applied IQ-TREE v2.1.4 to reconstruct the phylogenetic relationship of 6 species in the triangle of U and outgroup based on the protein-coding genes encoded by the plastid and mitochondrial genomes, respectively (Minh et al. 2020). Furthermore, we also counted the number of variants in organelle protein-coding genes in allotetraploids and their parents. Those results were consistent with the results of previous studies showing that the cytoplasmic donor of *B. napus* (AACC) remains unclear (An et al. 2019), so we excluded *B. napus* (AACC) in the following analysis.

### Inference of Orthologous Groups in Each Allopolyploid and Its Parents

We used both phylogenetic and syntenic methods to infer orthologous groups (OGs) as described by Sharbrough et al. (2022). In the phylogenetic method, we used OrthoFinder v2.3.8 to infer the OGs (referring to “primary OGs”) from the whole proteome (the longest transcript) of each allopolyploid and its diploid parents (Emms and Kelly 2019). We aligned the protein sequences of each OG by MAFFT v7.487, and the corresponding DNA sequences were aligned according to the aligned protein matrix by PAL2NAL v13 (Suyama et al. 2006; Nakamura et al. 2018). The uncertain regions of each OG were assigned confidence scores and filtered by the ZORRO and Perl scripts (Wu et al. 2012; Ran et al. 2018). Then, we employed IQ-TREE v2.1.4 to reconstruct the maximum likelihood tree, and subsequently, we applied PhyloTreePruner v. 1.0 to exclude paralogs (Kocot et al. 2013; Minh et al. 2020). Finally, we obtain all monophyletic OGs (which refer to “high-quality OGs”), which contain 1 sequence from each diploid parent, 2 from each subgenome in allopolyploid, and 1 from the outgroup. For the syntenic method, we inferred the genome-wide syntenic orthologs by pSONIC (Conover et al. 2021). Notably, as multiple round genome duplications and rapid genomic reshuffling in *Brassica* (Cheng et al. 2012; Hao et al. 2021), the OGs inferred by the syntenic method (e.g. 4,662 in *B. carinata* [BBCC]) was much fewer than that inferred by the phylogenetic method (e.g. 6,167 in *B. carinata* [BBCC]). Moreover, only a small subset of OGs overlapped (e.g. 1,862 in *B. carinata* [BBCC]) between those 2 methods. Therefore, we only took the high-quality OGs inferred from the phylogenetic method in the following analysis.

### Classification of Nuclear Genes Based on CyMIRA

As *Arabidopsis* and *Brassica* are closely related genera, we classified primary OGs and high-quality OGs according to CyMIRA, respectively (Forsythe et al. 2019). According to subcellular localization and direct molecular interaction with cytoplasmic and nuclear gene products, CyMIRA is divided into 11 categories and 27 subcategories. Among them, “CyMIRA targeting” and “CyMIRA Interaction” described subcellular localization and interacting organelles, respectively, whereas “CyMIRA Interaction Category” and “CyMIRA Interaction Subcategory” described the functional classification and complexes of direct cytonuclear interactions, respectively. According to “CyMIRA targeting,” nuclear genes were classified into 2 categories: nontargeted genes (“Other”) and organelle-targeted genes, depending on whether their products transported to organelles or not. According to “CyMIRA targeting,” “CyMIRA Interaction Category,” and “CyMIRA Interaction Subcategory,” the latter can be subdivided into 7 subcategories. The nuclear gene products that are incorporated into jointly encoded chimeric enzyme complexes including plastid (“pt_com”) and mitochondria enzyme complexes (“mt_com”; e.g. RuBisCo, photosystems I and II, OXPHOS complexes, ATPase, and organelle ribosomes). The nuclear gene products that participate in organelle genome replication, transcription, and translation (e.g. PPR genes), but are not incorporated into chimeric enzyme complexes, were designated as plastid- (“pt_int”) and mitochondria-interacting without complex (“mt_int”) genes. The nuclear gene products that are transported to the organelles but do not directly interact with the organelle gene products were termed plastid- (“pt_tar”) and mitochondria-targeted without interacting (“mt_tar”) genes. The last subcategory involves gene products transported into both plastids and mitochondria, referred to as dual-targeted genes (“Dual”). The number of genes in each functional category was counted, including all diploids, allopolyploids, and outgroup.

### Evolutionary Rate of Organelle-Targeted Genes

We calculated the evolutionary rate of each high-quality OG and concatenated the matrix of each functional category. For each OG, we retired DNA sequences and protein sequences according to the gene ID inferred by PhyloTreePruner, and the matrixes were aligned and filtered following the pipeline in the above section. We applied codeml within PAML v4.9j to estimate the synonymous rate (*d_S_*) in model 1 (NSsites = 0; Yang 2007). And we excluded poorly aligned OGs whose *d_S_* was larger than the cutoff values, which are depicted by red dot lines in supplementary fig. S18, Supplementary Material online. Then, we estimated *d_N_*, *d_S_*, and *ω* for each OG and concatenated the matrix of each functional category using model 1 and parameters according to Sharbrough et al. (2022).

### Detection of Gene Conversion

The gene conversion events between 2 subgenomes in each allopolyploid were detected according to the previous studies, which were based on the principle that the sequence similarity between orthologous genes is higher than that of homoeologs in the same OG (supplementary fig. S19a, Supplementary Material online; Song et al. 2021; Wei et al. 2021). For example, we divided the high-quality OGs into the following 4 types, as defined in *B. carinata* (BBCC).

1. “No conversion”: the similarity between the 2 group orthologous genes (BcaB and BniB, BcaC and BolC) is greater than that between the 2 group homoeologs (BcaB and BcaC, BcaC and BniB; supplementary fig. S19a, Supplementary Material online).
2. “C-to-B”: the similarity between the homoeologs (BcaB and BcaC) is greater than that between the 2 groups of orthologous genes, and the similarity between the orthologous genes (BcaB and BniB) is lower than that between the orthologous genes (BcaC and BolC; supplementary fig. S19b, Supplementary Material online).
3. “B-to-C”: the similarity between the homoeologs (BcaB and BcaC) is greater than that between the 2 groups of orthologous genes, and the similarity between the orthologous genes (BcaB and BniB) is greater than that between the orthologous genes (BcaC and BolC; supplementary fig. S19c, Supplementary Material online).
4. “Reciprocal or unknown”: the similarity of the 2 groups of homoeologs is greater than that between the 2 groups of orthologous genes (supplementary fig. S19d, Supplementary Material online).

### Expression Analyses in Allopolyploids

The RNA-seq data of each individual were downloaded from NCBI (https://www.ncbi.nlm.nih.gov/), and the raw data were filtered by FastQC v0.11.9 and Trimmomatic v0.39 (Bolger et al. 2014). Then, the clean reads of each allopolyploid were mapped to the corresponding genome by HISAT2 v2.2.1 (Kim et al. 2015), and the fragments per kilobase of transcript sequence per million base pairs (FPKM) were calculated by StringTie v2.2.1 (Pertea et al. 2016). The differentially expressed genes were conducted by DESeq2, and the | log_2_(fold change) | ≥ 1 was used as the threshold for screening differentially expressed genes (DEGs) (Love et al. 2014; Song et al. 2021).

### Analysis of Plastid RuBisCo and Mitochondrial Complex III Complexes

We selected the most typical plastid complex RuBisCo and the mitochondrial complex III to dissect the evolutionary pattern of organelle-targeted gene families in allopolyploids. To retrieve all genes encoded by these 2 complexes, the nuclear genes of *Arabidopsis* encoded by these 2 complexes were used as queries to tBLASTn against both protein and genome databases of all individuals used in this study (identity > 90%, *E* < 1E−5; Camacho et al. 2009). The BLAST hits were further filtered manually based on phylogenetic analysis (Zhang et al. 2020). The multiple sequence alignment of each gene family was generated by MAFFT v7.487 and PAL2NAL v13 (Suyama et al. 2006; Nakamura et al. 2018). The uncertain regions were filtered by ZORRO and Perl scripts (Wu et al. 2012; Ran et al. 2018). Then, the phylogenetic trees were reconstructed by IQ-TREE v2.1.4 under the GTR + I model (Minh et al. 2020). The gene conversion events were detected according to Gong et al. (2014), which were based on variants in alignments. The expression analysis was the same as in the above section.

## Supplementary Material

Supplementary material is available at *Molecular Biology and Evolution* online.

## Supplementary Material

msae043_Supplementary_Data

## Data Availability

NCBI accession numbers of genomic resources used in this study are provided in supplementary table S13, Supplementary Material online.
