# Supplementary material for: Cytonuclear Interactions and Subgenome Dominance Shape the Evolution of Organelle-Targeted Genes in the Brassica Triangle of U
Source: Mol Biol Evol. 2024 Feb 23;41(3):msae043. doi: 10.1093/molbev/msae043 (PMC10919925; doi:10.1093/molbev/msae043)
Supplement: msae043_Supplementary_Data [file msae043_supplementary_data.zip › Supplementary Figure S4.pdf]

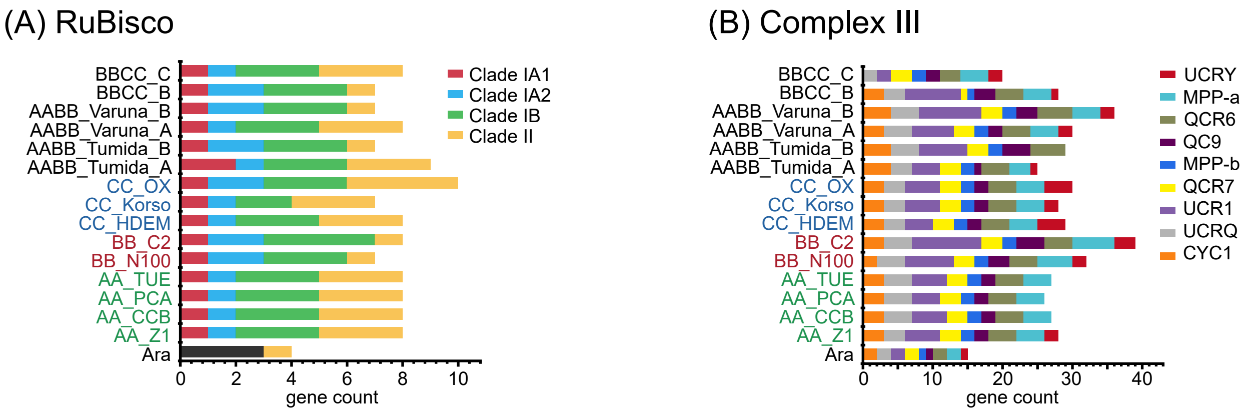

Supplementary Fig S4. Variations of nuclear genes number encoded Rubisco and Complex III complexes in studied genomes/subgenomes.
