# Supplementary material for: Cytonuclear Interactions and Subgenome Dominance Shape the Evolution of Organelle-Targeted Genes in the Brassica Triangle of U
Source: Mol Biol Evol. 2024 Feb 23;41(3):msae043. doi: 10.1093/molbev/msae043 (PMC10919925; doi:10.1093/molbev/msae043)
Supplement: msae043_Supplementary_Data [file msae043_supplementary_data.zip › Supplementary Figure S6.pdf]

(A) Clade IA-1 AABB

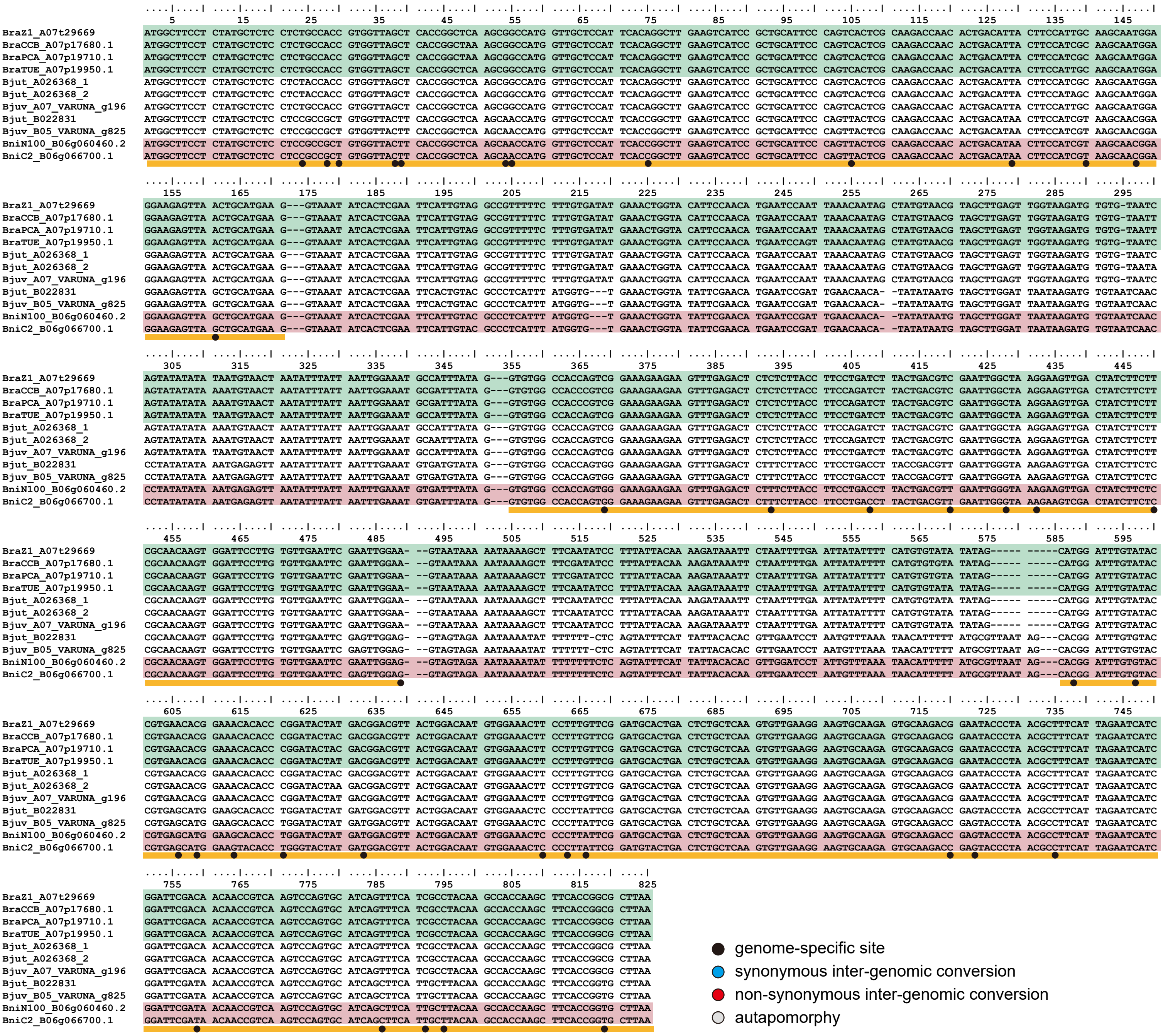

(B) Clade IA-1 BBCC

```
                         5         15         25         35         45         55         65         75         85         95        105        115        125        135        145
BolHDEM_C6t37020     ATGGCTTCCT CTATGCTCTC CTCTGCCACC GTGGTTAGCT CACCGGCTCA AGCGGCCATG GTTGCTCCAT TCACAGGCTT GAAGTCATCC GCTGCATTCC CAGTCACTCG CAAGACCGAC ACTGACATTA CTTCCATCGC AAGCAATGGA
BolOX_6g18050        ATGGCTTCCT CTATGCTCTC CTCTGCCACC GTGGTTAGCT CACCGGCTCA AGCGGCCATG GTTGCTCCAT TCACAGGCTT GAAGTCATCC GCTGCATTCC CAGTCACTCG CAAGACCGAC ACTGACATTA CTTCCATCGC AAGCAATGGA
BolKorso_6g17900     ATGGCTTCCT CTATGCTCTC CTCTGCCACC GTGGTTAGCT CACCGGCTCA AGCGGCCATG GTTGCTCCAT TCACAGGCTT GAAGTCATCC GCTGCATTCC CAGTCACTCG CAAGACCGAC ACTGACATTA CTTCCATGGC AAGCAATGGA
Bca_C08g44378        ATGGCTTCCT CTATGCTCTC CTCTGCCACC GTGGTTAGCT CACCGGCTCA AGCGGCCATG GTTGCTCCAT TCACAGGCTT GAAGTCATCC GCTGCATTCC CAGTCACTCG CAAGACCGAC ACTGACATTA CTTCCATGGC AAGCAATGGA
Bca_B02g08009        ATGGCTTCCT CTATGCTCTC CTCCGCCGCT GTGGTTACTT CACCGGCTCA AGCAACCATG GTTGCTCCAT TCACCGGCTT GAAGTCATCC GCTGCATTCC CAGTTACTCG CAAGACCAAC ACTGACATAA CTTCCATCGT AAGCAACGGA
BniN100_B06g060460.2 ATGGCTTCCT CTATGCTCTC CTCCGCCGCT GTGGTTACTT CACCGGCTCA AGCAACCATG GTTGCTCCAT TCACCGGCTT GAAGTCATCC GCTGCATTCC CAGTTACTCG CAAGACCAAC ACTGACATAA CTTCCATCGT AAGCAACGGA
BniC2_B06g066700.1   ATGGCTTCCT CTATGCTCTC CTCCGCCGCT GTGGTTACTT CACCGGCTCA AGCAACCATG GTTGCTCCAT TCACCGGCTT GAAGTCATCC GCTGCATTCC CAGTTACTCG CAAGACCAAC ACTGACATAA CTTCCATCGT AAGCAACGGA

                       155        165        175        185        195        205        215        225        235        245        255        265        275        285        295
BolHDEM_C6t37020     GGAAGAGTTA ACTGCATGAA G---GTAAAT ATCACTCGAA TCCATTGTAG GCCGTTTTTC TTTGTGATAT GAAACTGGTA CATTCCAACA TGAATCCAAT TAAACAATAG CTATGTAACG TAGCTTGAGT TGGTAAGATG TGTGTAATCA
BolOX_6g18050        GGAAGAGTTA ACTGCATGAA G---GTAAAT ATCACTCGAA TCCATTGTAG GCCGTTTTTC TTTGTGATAT GAAACTGGTA CATTCCAACA TGAATCCAAT TAAACAATAG CTATGTAACG TAGCTTGAGT TGGTAAGATG TGTGTAATCA
BolKorso_6g17900     GGAAGAGTTA ACTGCATGAA G---GTAAAT ATCACTCGAA TCCATTGTAG GCCGTTTTTC TTTGTGATAT GAAACTGGTA CATTCCAACA TGAATCCAAT TAAACAATAG CTATGTAACG TAGCTTGAGT TGGTAAGATG TGTGTAATCA
Bca_C08g44378        GGAAGAGTTA ACTGCATGAA G---GTAAAT ATCACTCGAA TCCATTGTAG GCCGTTTTTC TTTGTGATAT GAAACTGGTA CATTCCAACA TGAATCCAAT TAAACAATAG CTATGTAACG TAGCTTGAGT TGGTAAGATG TGTGTAATCA
Bca_B02g08009        GGAAGAGTTA GCTGCATGAA G---GTAAAT ATCACTCGAA TTCATTGTAC GCCCTCATTT ATGGTGTGAA ACTGGTATAT TCGAACATGA AT---CCGAT TGAACAACAT ATATAATGTA GCTTGGATTA ATAAGACGTG TAATCAACCC
BniN100_B06g060460.2 GGAAGAGTTA GCTGCATGAA G---GTAAAT ATCACTCGAA TTCATTGTAC GCCCTCATTT ATGGTGTGAA ACTGGTATAT TCGAACATGA AT---CCGAT TGAACAACAT ATATAATGTA GCTTGGATTA ATAAGATGTG TAATCAACCC
BniC2_B06g066700.1   GGAAGAGTTA GCTGCATGAA G---GTAAAT ATCACTCGAA TTCATTGTAC GCCCTCATTT ATGGTGTGAA ACTGGTATAT TCGAACATGA AT---CCGAT TGAACAACAT ATATAATGTA GCTTGGATTA ATAAGATGTG TAATCAACCC

                       305        315        325        335        345        355        365        375        385        395        405        415        425        435        445
BolHDEM_C6t37020     GTATATATAA AATGTAACTA ATAT-----A ATTGGAAATG TGATTTATAG ----GTGTGG CCACCAGTCG GGAAGAAGAA GTTTGAGACT CTCTCTTACC TTCCTGATCT TACTGACGTC GAATTGTCTA AGGAAGTTGA CTATCTTCTT
BolOX_6g18050        GTATATATAA AATGTAACTA ATAT-----A ATTGGAAATG TGATTTATAG ----GTGTGG CCACCAGTCG GGAAGAAGAA GTTTGAGACT CTCTCTTACC TTCCTGATCT TACTGACGTC GAATTGTCTA AGGAAGTTGA CTATCTTCTT
BolKorso_6g17900     GTATGTATAA AATGTAACTA ATATTTATTA ATTGGAAATG TGATTTATAG ----GTGTGG CCACCAGTCG GGAAGAAGAA GTTTGAGACT CTCTCTTACC TTCCTGATCT TACTGACGTC GAATTGTCTA AGGAAGTTGA CTATCTTCTT
Bca_C08g44378        GTATGTATAA AATGTAACTA ATATTTATTA ATTGGAAATG TGATTTATAG ----GTGTGG CCACCAGTCG GGAAGAAGAA GTTTGAGACT CTCTCTTACC TTCCTGATCT TACTGACGTC GAATTGTCTA AGGAAGTTGA CTATCTTCTT
Bca_B02g08009        -TATATATAA ATGAGAGTTA ATATTTATTA ATTTGAAATG TGATTTATAG ----GTGTGG CCACCAGTGG GAAAGAAGAA GTTTGAGACT CTTTCTTACC TTCCTGACCT TACCGACGTT GAATTGGGTA AAGAAGTTGA CTATCTTCTC
BniN100_B06g060460.2 -TATATATAA ATGAGAGTTA ATATTTATTA ATTTGAAATG TGATTTATAG ----GTGTGG CCACCAGTGG GAAAGAAGAA GTTTGAGACT CTTTCTTACC TTCCTGACCT TACTGACGTT GAATTGGGTA AAGAAGTTGA CTATCTTCTC
BniC2_B06g066700.1   -TATATATAA ATGAGAGTTA ATATTTATTA ATTTGAAATG TGATTTATAG ----GTGTGG CCACCAGTGG GAAAGAAGAA GTTTGAGACT CTTTCTTACC TTCCTGACCT TACTGACGTT GAATTGGGTA AAGAAGTCGA CTATCTTCTC

                       455        465        475        485        495        505        515        525        535        545        555        565        575        585        595
BolHDEM_C6t37020     CGCAACAAGT GGATTCCTTG TGTTGAATTC GAATTGGAA- --GTAATAAA AATAAAAGCT TTCAATATCC TTTATTACAA AAGATAAATT CTAATTTTGA ATTATATTTT CATGTGTGTA TAG------- -----CATGG ATTTGTATAC
BolOX_6g18050        CGCAACAAGT GGATTCCTTG TGTTGAATTC GAATTGGAA- --GTAATAAA AATAAAAGCT TTCAATATCC TTTATTACAA AAGATAAATT CTAATTTTGA ATTATATTTT CATGTGTGTA TAG------- -----CATGG ATTTGTATAC
BolKorso_6g17900     CGCAACAAGT GGATTCCTTG TGTTGAATTC GAATTGGAA- --GTAATAAA AATAAAAGCT TTCAATATCC TTTATTACGA AAGATAAATT CTAATTTTGA ATTATTTTTT CATGTGTGTA TAG------- -----CATGG ATTTGTATAC
Bca_C08g44378        CGCAACAAGT GGATTCCTTG TGTTGAATTC GAATTGGAA- --GTAATAAA AATAAAAGCT TTCAATATCC TTTATTACGA AAGATAAATT CTAATTTTGA ATTATATTTT CATGTGTGTA TAG------- -----CATGG ATTTGTATAC
Bca_B02g08009        CGGAACAAGT GGATTCCTTG TGTTGAATTC GAGTTGGAG- --GTAGTAGA AATAAAATAT TTTTTTCTCA GTATTTCATT ATTACACAAG TTGGATCCTA ATGTTTAAAT AACATTTTTA TGCGTTAATA G----CACGG ATTTGTGTAC
BniN100_B06g060460.2 CGCAACAAGT GGATTCCTTG TGTTGAATTC GAATTGGAG- --GTAGTAGA AATAAAATAT TTTTTTTCTC AGTATTTCAT TATTACACAC GTTGGATCCT ATTGTTTAAA TAACATTTTT ATGCGTTAAT AG---CACGG ATTTGTGTAC
BniC2_B06g066700.1   CGCAACAAGT GGATTCCTTG TGTTGAATTC GAGTTGGAG- --GTAGTAGA AATAAAATAT TTTTTTTCTC AGTATTTCAT TATTACACAA GTTGAATCCT AATGTTTAAA TAACATTTTT ATGCGTTAAT AG---CACGG ATTTGTGTAC

                       605        615        625        635        645        655        665        675        685        695        705        715        725        735        745
BolHDEM_C6t37020     CGTGAACACG GAAACACACC CGGATACTAT GACGGACGTT ACTGGACAAT GTGGAAACTT CCTTTGTTCG GATGCACTGA CTCTGCTCAA GTGTTGAAGG AAGTGCAAGA GTGCAAGAAG GAATACCCTA ACGCTTTCAT TAGAATCATC
BolOX_6g18050        CGTGAACACG GAAACACACC CGGATACTAT GACGGACGTT ACTGGACAAT GTGGAAACTT CCTTTGTTCG GATGCACTGA CTCTGCTCAA GTGTTGAAGG AAGTGCAAGA GTGCAAGAAG GAATACCCTA ACGCTTTCAT TAGAATCATC
BolKorso_6g17900     CGTGAACACG GAAACACACC CGGATACTAT GACGGACGTT ACTGGACAAT GTGGAAACTT CCTTTGTTCG GATGCACTGA CTCTGCTCAA GTGTTGAAGG AAGTGCAAGA GTGCAAGAAG GAATATCCTA ACGCTTTCAT TAGAATCATC
Bca_C08g44378        CGTGAACACG GAAACACACC CGGATACTAT GACGGACGTT ACTGGACAAT GTGGAAACTT CCTTTGTTCG GATGCACTGA CTCTGCTCAA GTATTGAAGG AAGTGCAAGA GTGCAAGAAG GAATATCCTA ACGCTTTCAT TAGAATCATC
Bca_B02g08009        CGTGAGCATG GAAGCACACC TGGATACTAT GATGGACGTT ACTGGACAAT GTGGAAACTC CCCTTATTCG GATGCACTGA CTCTGCTCAA GTGTTGAAGG AAGTGCAAGA GTGCAAGACC GAGTACCCTA ACGCCTTCAT TAGAATCATC
BniN100_B06g060460.2 CGTGAGCATG GAAGCACACC TGGATACTAT GATGGACGTT ACTGGACAAT GTGGAAACTC CCCTTATTCG GATGCACTGA CTCTGCTCAA GTGTTGAAGG AAGTGCAAGA GTGCAAGACC GAGTACCCTA ACGCCTTCAT TAGAATCATC
BniC2_B06g066700.1   CGTGAGCATG GAAGTACACC TGGGTACTAT GATGGACGTT ACTGGACAAT GTGGAAACTC CCCTTATTCG GATGTACTGA CTCTGCTCAA GTGTTGAAGG AAGTGCAAGA GTGCAAGACC GAGTACCCTA ACGCCTTCAT TAGAATCATC

                       755        765        775        785        795        805        815        825
BolHDEM_C6t37020     GGATTCGACA ACAACCGTCA AGTCCAGTGC ATCAGTTTCA TCGCCTACAA GCCACCAAGC TTCACCGGTG CTTAA
BolOX_6g18050        GGATTCGACA ACAACCGTCA AGTCCAGTGC ATCAGTTTCA TCGCCTACAA GCCACCAAGC TTCACCGGTG CTTAA
BolKorso_6g17900     GGATTCGACA ACAACCGTCA AGTCCAGTGC ATCAGTTTCA TCGCCTACAA GCCACCAAGC TTCACCGGTG CTTAA
Bca_C08g44378        GGATTCGACA ACAACCGTCA AGTCCAGTGC ATCAGTTTCA TCGCCTACAA GCCACCAAGC TTCACCGGTG CTTAA
Bca_B02g08009        GGATTCGATA ACAACCGTCA AGTCCAGTGC ATCAGCTTCA TTGCTTACAA GCCACCAAGC TTCACCGGTG CTTAA
BniN100_B06g060460.2 GGATTCGATA ACAACCGTCA AGTCCAGTGC ATCAGCTTCA TTGCTTACAA GCCACCAAGC TTCACCGGTG CTTAA
BniC2_B06g066700.1   GGATTCGATA ACAACCGTCA AGTCCAGTGC ATCAGCTTCA TTGCTTACAA GCCACCAAGC TTCACCGGTG CTTAA
```

(C) Clade IA-2 AABB

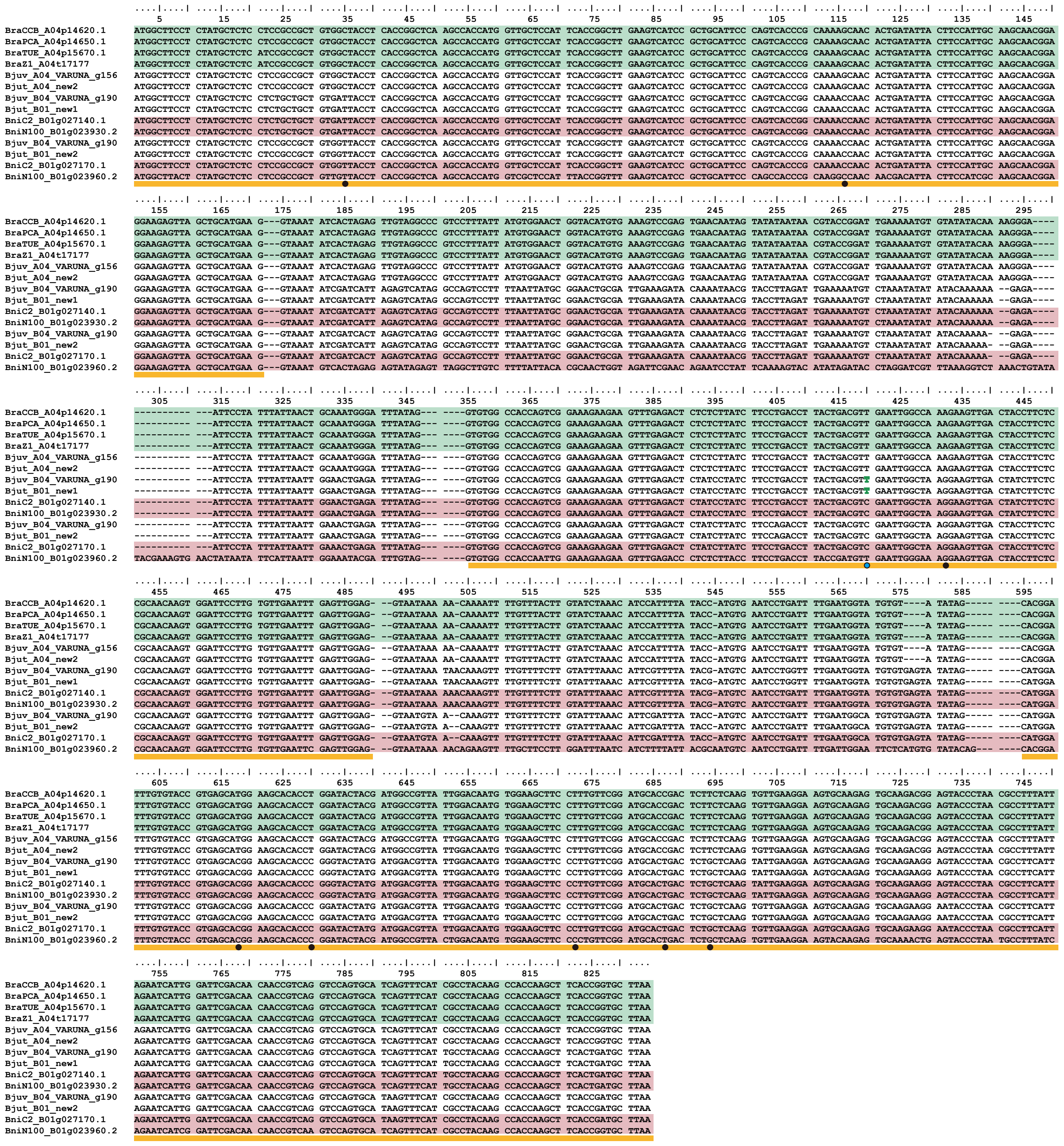

(D) Clade IA-2 BBCC

```
                         5          15         25         35         45         55         65         75         85         95         105        115        125        135        145
BolKorso_4g48740         ATGGCTTCCT CTATGCTCTC CTCCGCCGCT GTGGCTACCT CACCGGCTCA AGCCACCATG GTTGCTCCAT TCACTGGCTT GAAGTCATCC GCTGCATTCC CAGTCACCCG CAAAAGCAAC ACTGATATTA CTTCCATTGC AAGCAACGGA
BolHDEM_C4t26547         ATGGCTTCCT CTATGCTCTC CTCCGCCGCT GTGGCTACCT CACCGGCTCA AGCCACCATG GTTGCTCCAT TCACTGGCTT GAAGTCATCC GCTGCATTCC CAGTCACCCG CAAAAGCAAC ACTGATATTA CTTCCATTGC AAGCAACGGA
BolOX_4g45770            ATGGCTTCCT CTATGCTCTC CTCCGCCGCT GTGGCTACCT CACCGGCTCA AGCCACCATG GTTGCTCCAT TCACCGGCTT GAAGTCATCC GCTGCATTCC CAGTCACCCG CAAAAGCAAC ACTGATATTA CTTCCATTGC AAACAACGGA
BolOX_0g08600            ATGGCTTCCT CTATGCTCTC CTCCGCCGCT GTGGCTACCT CACCGGCTCA AGCCACCATG GTTGCTCCAT TCACCGGCTT GAAGTCATCC GCTGCATTCC CAGTCACCCG CAAAAGCAAC ACTGATATTA CTTCCATTGC AAACAACGGA
Bca_C02g08874            ATGGCTTCCT CTATGCTCTC CTCCGCCGCT GTGGCTACCT CACCGGCTCA AGCCACCATG GTTGCTCCAT TCACCGGCTT GAAGTCATCC GCTGCATTCC CAGTCACCCG CAAAAGCAAC ACTGATATTA CTTCCATTGC AAACAACGGA
Bca_B06g27715            ATGGCTTCCT CTATGCTCTC CTCTGCTGCT GTGATTACCT CACCGGCTCA AGCCACCATG GTTGCTCCAT TCACCGGCTT GAAGTCATCC GCTGCATTCC CAGTCACCCG CAAAACCAAC ACTGATATTA CTTCCATTGC AAGCAACGGA
BniC2_B01g027140.1       ATGGCTTCCT CTATGCTCTC CTCTGCTGCT GTGATTACCT CACCGGCTCA AGCCACCATG GTTGCTCCAT TCACCGGCTT GAAGTCATCC GCTGCATTCC CAGTCACCGG CAAAACCAAC ACTGATATTA CTTCCATTGC AAGCAACGGA
BniN100_B01g023930.2     ATGGCTTCCT CTATGCTCTC CTCTGCTGCT GTGATTACCT CACCGGCTCA AGCCACCATG GTTGCTCCAT TCACCGGCTT GAAGTCATCC GCTGCATTCC CAGTCACCGG CAAAACCAAC ACTGATATTA CTTCCATTGC AAGCAACGGA
Bca_B06g27713            ATGGCTTCCT CTATGCTCTC CTCCGCCGCT GTGGTTACCT CACCGGCTCA AGCCACCATG GTTGCTCCAT TCACCGGCTT GAAGTCATCC GCTGCATTCC CAGTCACCCG CAAAACCAAC ACTGATATTA CTTCCATTGC AAGCAACGGA
BniC2_B01g027170.1       ATGGCTTCCT CTATGCTCTC CTCCGCCGCT GTGGTTACCT CACCGGCTCA AGCCACCATG GTTGCTCCAT TCACCGGCTT GAAGTCATCC GCTGCATTCC CAGTCACCCG CAAAACCAAC ACTGATATTA CTTCCATTGC AAGCAACGGA
BniN100_B01g023960.2     ATGGCTTACT CTATGCTCTC CTCCGCCGCT GTTGTTACCT CACCGGCTCA AGCCACCATG GTCGCTCCAT TTACCGGTTT GAAGTCATCC GCTGCATTCC CAGCCACCCG CAAGGCCAAC AACGACATTA CTTCCATCGC AAGCAACGGA

                         155        165        175        185        195        205        215        225        235        245        255        265        275        285        295
BolKorso_4g48740         GGAAGAGTTA GCTGCATGAA G---GTAAAT ATCACTAGAG TTGTAGGCCC GTCCTTTATT ATGTGGAACT GGTACATGTG AAAATCCGAG TGAACAATAG TATATAATAA CGTACCGGAT TGAAAAA--- ---------- ---TGTGTAT
BolHDEM_C4t26547         GGAAGAGTTA GCTGCATGAA G---GTAAAT ATCACTAGAG TTGTAGGCCC GTCCTTTATT ATGTGGAACT GGTACATGTG AAAATCCGAG TGAACAATAG TATATAATAA CGTACCGGAT TGAAAAA--- ---------- ---TGTGTAT
BolOX_4g45770            GGAAGAGTTA GCTGCATGAA G---GTAAAT ATCACTAGAG TTGTAGGCCC GTCCTTTATT ATGTGGAACT GGTACATGTG AAAATCCGAG TGAACAATAG TATATAATAA CGTACCGGAT TGAAAAA--- ---------- ---TGTGTAT
BolOX_0g08600            GGAAGAGTTA GCTGCATGAA G---GTAAAT ATCACTAGAG TTGTAGGCCC GTCCTTTATT ATGTGGAACT GGTACATGTG AAAATCCGAG TGAACAATAG TATATAATAA CGTACCGGAT TGAAAAA--- ---------- ---TGTGTAT
Bca_C02g08874            GGAAGAGTTA GCTGCATGAA G---GTAAAT ATCACTAGAG TTGTAGGCCC GTCCTTTATT ATGTGGAACT GGTACATGTG AAAATCCGAG TGAACAATAG TATATAATAA CGTACCGGAT TGAAAAA--- ---------- ---TGTGTAT
Bca_B06g27715            GGAAGAGTTA GCTGCATGAA G---GTAAAT ATCGATCACT TGAGTCATAG GCCAGTCCTT TTAATTATGC GGAACTGCGA TTGAAAGATA CAAAATAACG TACCTTAGAT TGAAAAATGT CTAAA----- ---------- ---TATATAT
BniC2_B01g027140.1       GGAAGAGTTA GCTGCATGAA G---GTAAAT ATCGATCATT AGAGTCATAG GCCAGTCCTT TTAATTATGC GGAACTGCGA TTGAAAGATA CAAAATAACG TACCTTAGAT TGAAAAATGT CTAAA----- ---------- ---TATATAT
BniN100_B01g023930.2     GGAAGAGTTA GCTGCATGAA G---GTAAAT ATCGATCATT AGAGTCATAG GCCAGTCCTT TTAATTATGC GGAACTGCGA TTGAAAGATA CAAAATAACG TACCTTAGAT TGAAAAATGT CTAAA----- ---------- ---TATATAT
Bca_B06g27713            GGAAGAGTTA GCTGCATGAA G---GTAAAT ATCGATCACT TGAGTCATAG GCCAGTCCTT TTAATTATGC GGAACTGCGA TTGAAAGATA CAAAATAACG TACCTTAGAT TGAAAAATGT CTAAA----- ---------- ---TATATAT
BniC2_B01g027170.1       GGAAGAGTTA GCTGCATGAA G---GTAAAT ATCGATCACT AGAGTCATAG GCCAGTCCTT TTAATTATGC GGAACTGCGA TTGAAAGATA CAAAATAACG TACCTTAGAT TGAAAAATGT CTAAA----- ---------- ---TATATAT
BniN100_B01g023960.2     GGAAGAGTTA GCTGCATGAA G---GTAAAT GTCACTAGAG AGTATAGAGT TAGGCTTGTC TTTTATTACA CGCAACTGGT AGATTCGAAC AGAATCCTAT TCAAAAGTAC ATATAGATAC CTAGGATCGT TTAAAGGTCT AAACTGTATA

                         305        315        325        335        345        355        365        375        385        395        405        415        425        435        445
BolKorso_4g48740         ATACAAAAGG GAA-TTCCTA TTTATTAACT GCAAAATGGG ATTTATAG-- ----GTGTGG CCACCAGTCG GAAAGAAAAA GTTTGAGACT CTCTCTTATC TTCCTGACCT TACTGACGTT GAATTGGCCA AAGAAGTTGA CTACCTTCTC
BolHDEM_C4t26547         ATACAAAAGG GAA-TTCCTA TTTATTAACT GCAAAATGGG ATTTATAG-- ----GTGTGG CCACCAGTCG GAAAGAAAAA GTTTGAGACT CTCTCTTATC TTCCTGACCT TACTGACGTT GAATTGGCCA AAGAAGTTGA CTACCTTCTC
BolOX_4g45770            ATACAAAAGG GAA-TTCCTA TTTATTAACT GCAAA-TGGG ATTTATAG-- ----GTGTGG CCACCAGTTG GAAAGAAGAA GTTTGAGACT CTCTCTTATC TTCCTGACCT TACTGACGTT GAATTGGCCA AAGAAGTTGA CTACCTTCTC
BolOX_0g08600            ATACAAAAGG GAA-TTCCTA TTTATTAACT GCAAA-TGGG ATTTATAG-- ----GTGTGG CCACCAGTTG GAAAGAAGAA GTTTGAGACT CTCTCTTATC TTCCTGACCT TACTGACGTT GAATTGGCCA AAGAAGTTGA CTACCTTCTC
Bca_C02g08874            ATACAAAAGG GAA-TTCCTA TTTATTAACT GCAAA-TGGG ATTTATAG-- ----GTGTGG CCACCAGTTG GAAAGAAGAA GTTTGAGACT CTCTCTTATC TTCCTGACCT TACTGACGTT GAATTGGCCA AAGAAGTTGA CTACCTTCTC
Bca_B06g27715            ACAAAAA-GA GAA-TTCCTA TTTATTAATT GGAAC-TGAG ATTTATAG-- ----GTGTGG CCACCAGTCG GAAAGAAGAA GTTTGAGACT CTATCCTATC TTCCTGACCT TACTGACGTC GAATTGGCTA AGGAAGTTGA CTATCTTCTC
BniC2_B01g027140.1       ACAAAAAAGA GAA-TTCCTA TTTATTAATT GGAAC-TGAG ATTTATAG-- ----GTGTGG CCACCAGTCG GAAAGAAGAA GTTTGAGACT CTATCCTATC TTCCTGACCT TACTGACGTC GAATTGGCTA AGGAAGTTGA CTATCTTCTC
BniN100_B01g023930.2     ACAAAAAAGA GAA-TTCCTA TTTATTAATT GGAAC-TGAG ATTTATAG-- ----GTGTGG CCACCAGTCG GAAAGAAGAA GTTTGAGACT CTATCCTATC TTCCTGACCT TACTGACGTC GAATTGGCTA AGGAAGTTGA CTATCTTCTC
Bca_B06g27713            ACAAAAA-GA GAATTCCTAT TT-ATTAATT G-AAACTGAG ATTTATAG-- ----GTGTGG CCACCAGTCG GAAAGAAGAA GTTTGAGACT CTATCTTATC TTCCTGACCT TACTGACGTC GAATTGGCTA AGGAAGTTGA CTACCTTCTC
BniC2_B01g027170.1       ACAAAAA-GA GAA-TTCCTA TTTATTAATT GAAAC-TGAG ATTTATAG-- ----GTGTGG CCACCAGTCG GAAAGAAGAA GTTTGAGACT CTATCTTATC TTCCTGACCT TACTGACGTC GAATTGGCTA AGGAAGTTGA CTACCTTCTC
BniN100_B01g023960.2     TACGAAA-GT GAACTATAAT ATTCATTAAT TGGAAATACG ATTTGTAG-- ----GTGTGG CCACCAATTG GAAAGAAGAA GTTTGAGACC CTCTCTTACC TTCCTGACCT TACCGATGTT GAATTGGGAA AGGAAGTTGA CTACCTTCTC

                         455        465        475        485        495        505        515        525        535        545        555        565        575        585        595
BolKorso_4g48740         CGCAACAAGT GGATTCCTTG TGTTGAATTT GAGTTGGAG- --GTAATAAA AA-CAAAATT TTGTTTACTT GTATCTAAAC ATCCATTTAA TACCA-TGTG AATCCTGATT TTGAATGGTA TGTGT----A TATAG----- -CACGGATTT
BolHDEM_C4t26547         CGCAACAAGT GGATTCCTTG TGTTGAATTT GAGTTGGAG- --GTAATAAA AA-CAAAATT TTGTTTACTT GTATCTAAAC ATCCATTTAA TACCA-TGTG AATCCTGATT TTGAATGGTA TGTGT----A TATAG----- -CACGGATTT
BolOX_4g45770            CGCAACAAGT GGATTCCTTG TGTTGAATTT GAGTTGGAG- --GTAATAAA AA-CAAAATT TTGTTTACTT GTATCTAAAC ATCCATTTTA TACCA-TGTG AATCATGATT TTGAATGGTA TGTCT----A TATAG----- -CACGGATTT
BolOX_0g08600            CGCAACAAGT GGATTCCTTG TGTTGAATTT GAGTTGGAG- --GTAATAAA AA-CAAAATT TTGTTTACTT GTATCTAAAC ATCCATTTTA TACCA-TGTG AATCATGATT TTGAATGGTA TGTCT----A TATAG----- -CACGGATTT
Bca_C02g08874            CGCAACAAGT GGATTCCTTG TGTTGAATTT GAGTTGGAG- --GTAATAAA AA-CAAAATT TTGTTTACTT GTATCTAAAC ATCCATTTTA TACCA-TGTG AATCATGATT TTGAATGGTA TGTCT----A TATAG----- -CACGGATTT
Bca_B06g27715            CGCAACAAGT GGATTCCTTG TGTTGAATTT GAATTGGAG- --GTAATAAA TAACAAAGTT TTGTTTTCTT GTATTTAAAC ATTCGTTTTA TACGA-TGTC AATCCTGATT TTGAATGGTA TGTGTGAGTA TATAG----- -CATGGATTT
BniC2_B01g027140.1       CGCAACAAGT GGATTCCTTG TGTTGAATTT GAATTGGAG- --GTAATAAA AAACAAAGTT TTGTTTTCTT GTATTTAAAC ATTCGTTTTA TACGA-TGTC AATCCTGATT TTGAATGGTA TGTGTGAGTA TATAG----- -CATGGATTT
BniN100_B01g023930.2     CGCAACAAGT GGATTCCTTG TGTTGAATTT GAATTGGAG- --GTAATAAA AAACAAAGTT TTGTTTTCTT GTATTTAAAC ATTCGTTTTA TACGA-TGTC AATCCTGATT TTGAATGGTA TGTGTGAGTA TATAG----- -CATGGATTT
Bca_B06g27713            CGCAACAAAT GGATTCCTTG TGTTGAATTT GAGTTGGAG- --GTAATGTA ACAAA--GTT TTGTTTTCTT GTATTTAAAC ATTCGATTTA TACCA-TGTC AATCCTGATT TTGAATGGCA TGTGTGAGTA TATAG----- -CATGGATTT
BniC2_B01g027170.1       CGCAACAAGT GGATTCCTTG TGTTGAATTT GAGTTGGAG- --GTAATGTA ACAAA--GTT TTGTTTTCTT GTATTTAAAC ATTCGATTTA TACCA-TGTC AATCCTGATT TTGAATGGCA TGTGTGAGTA TATAG----- -CATGGATTT
BniN100_B01g023960.2     CGCAACAAGT GGATTCCTTG TGTTGAATTC GAGTTGGAG- --GTAATAAA AACAGAAGTT TTGCTTCCTT GGATTTAATC ATCTTTTATT ACGCAATGTC AATCCTGATT TTGATTGGAA TTCTCATGTG TATACAG--- -CACGGATTT

                         605        615        625        635        645        655        665        675        685        695        705        715        725        735        745
BolKorso_4g48740         GTGTACCGTG AGCATGGAAG CACACCTGGA TACTACGATG GCCGTTATTG GACAATGTGG AAGCTTCCTT TGTTCGGATG CACTGACTCT GCTCAAGTGT TGAAGGAAGT GCAAGAGTGC AAGACGGAGT ACCCTAACGC CTTTATTAGA
BolHDEM_C4t26547         GTGTACCGTG AGCATGGAAG CACACCTGGA TACTACGATG GCCGTTATTG GACAATGTGG AAGCTTCCTT TGTTCGGATG CACTGACTCT GCTCAAGTGT TGAAGGAAGT GCAAGAGTGC AAGACGGAGT ACCCTAACGC CTTTATTAGA
BolOX_4g45770            GTGTACCGTG AGCATGGAAG CACACCTGGA TACTACGATG GCCGTTATTG GACAATGTGG AAGCTTCCTT TGTTTGGATG CACTGACTCT GCTCAAGTGT TGAAGGAAGT GCAAGAGTGC AAGACGGAGT ACCCTAACGC CTTTATTAGA
BolOX_0g08600            GTGTACCGTG AGCATGGAAG CACACCTGGA TACTACGATG GCCGTTATTG GACAATGTGG AAGCTTCCTT TGTTTGGATG CACTGACTCT GCTCAAGTGT TGAAGGAAGT GCAAGAGTGC AAGACGGAGT ACCCTAACGC CTTTATTAGA
Bca_C02g08874            GTGTACCGTG AGCATGGAAG CACACCTGGA TACTACGATG GCCGTTATTG GACAATGTGG AAGCTTCCTT TGTTTGGATG CACTGACTCT GCTCAAGTGT TGAAGGAAGT GCAAGAGTGC AAGACGGAGT ACCCTAACGC CTTTATTAGA
Bca_B06g27715            GTGTACCGTG AGCACGGAAG CACACCCGGG TACTATGATG GACGTTATTG GACAATGTGG AAGCTTCCCT TGTTCGGATG CACTGACTCT GCTCAAGTAT TGAAGGAAGT GCAAGAGTGC AAGAAGGAGT ACCCTAACGC CTTCATTAGA
BniC2_B01g027140.1       GTGTACCGTG AGCACGGAAG CACACCCGGG TACTATGATG GACGTTATTG GACAATGTGG AAGCTTCCCT TGTTCGGATG CACTGACTCT GCTCAAGTAT TGAAGGAAGT GCAAGAGTGC AAGAAGGAGT ACCCTAACGC CTTCATTAGA
BniN100_B01g023930.2     GTGTACCGTG AGCACGGAAG CACACCCGGG TACTATGATG GACGTTATTG GACAATGTGG AAGCTTCCCT TGTTCGGATG CACTGACTCT GCTCAAGTAT TGAAGGAAGT GCAAGAGTGC AAGAAGGAGT ACCCTAACGC CTTCATTAGA
Bca_B06g27713            GTGTACCGTG AGCACGGAAG CACACCCGGA TACTATGATG GACGTTATTG GACAATGTGG AAGCTTCCCT TGTTCGGATG CACTGACTCT GCTCAAGTGT TGAAGGAAGT GCAAGAGTGC AAGAAGGAAT ACCCTAACGC CTTCATTAGA
BniC2_B01g027170.1       GTGTACCGTG AGCACGGAAG CACACCCGGA TACTATGATG GACGTTATTG GACAATGTGG AAGCTTCCCT TGTTCGGATG CACTGACTCT GCTCAAGTGT TGAAGGAAGT GCAAGAGTGC AAGAAGGAAT ACCCTAACGC CTTCATTAGA
BniN100_B01g023960.2     GTCTACCGTG AGCACGGAAG CACACCCGGA TACTACGATG GCCGTTACTG GACAATGTGG AAGCTTCCCC TGTTCGGATG CACTGACTCT GCTCAAGTGT TGAAGGAAGT ACAAGAGTGC AAAACTGAGT ACCCTAATGC CTTTATCAGA

                         755        765        775        785        795        805        815        825
BolKorso_4g48740         ATCATTGGAT TCGACAACAA CCGTCAGGTC CAGTGCATCA GTTTCATCGC CTACAAGCCA CCAAGCTTCA CCGGTGCTTA A
BolHDEM_C4t26547         ATCATTGGAT TCGACAACAA CCGTCAGGTC CAGTGCATCA GTTTCATCGC CTACAAGCCA CCAAGCTTCA CCGGTGCTTA A
BolOX_4g45770            ATCATTGGAT TCGACAACAA CCGTCAGGTC CAGTGCATCA GTTTCATCGC CTACAAGCCA CCAAGCTTCA CCGGTGCTTA A
BolOX_0g08600            ATCATTGGAT TCGACAACAA CCGTCAGGTC CAGTGCATCA GTTTCATCGC CTACAAGCCA CCAAGCTTCA CCGGTGCTTA A
Bca_C02g08874            ATCATTGGAT TCGACAACAA CCGTCAGGTC CAGTGCATCA GTTTCATCGC CTACAAGCCA CCAAGCTTCA CCGGTGCTTA A
Bca_B06g27715            ATCATTGGAT TCGACAACAA CCGTCAGGTC CAGTGCATCA GTTTCATTGC CTACAAGCCA CCAAGCTTCA CTGATGCTTA A
BniC2_B01g027140.1       ATCATTGGAT TCGACAACAA CCGTCAGGTC CAGTGCATCA GTTTCATTGC CTACAAGCCA CCAAGCTTCA CTGATGCTTA A
BniN100_B01g023930.2     ATCATTGGAT TCGACAACAA CCGTCAGGTC CAGTGCATCA GTTTCATTGC CTACAAGCCA CCAAGCTTCA CTGATGCTTA A
Bca_B06g27713            ATCATTGGAT TCGACAACAA CCGTCAGGTC CAGTGCATAA GTTTCATCGC CTACAAGCCA CCAAGCTTCA CCGATGCTTA A
BniC2_B01g027170.1       ATCATTGGAT TCGACAACAA CCGTCAGGTC CAGTGCATAA GTTTCATCGC CTACAAGCCA CCAAGCTTCA CCGATGCTTA A
BniN100_B01g023960.2     ATCATCGGAT TCGACAACAA CCGTCAAGTC CAGTGCATCA GTTTCATCGC CTACAAGCCA CCAAGCTTCA CCGGTGCTTA A
```

(E) Clade IB AABB

```
                          5         15         25         35         45         55         65         75         85         95        105        115        125        135        145
BraTUE_A04p11990.1    ATGGCTTACT CTATGCTCTC CTCCGCCGCT GTTGTTACCT CCCCGGCTCA AGCCACCATG GTTGCTCCAT TCACCGGCTT GAAGTCTTCC TCTGCATTCC CAGTCACCCG CAAGGCCAAC AACGACATTA CTTCCATCGT TAGCAACGGA
BraZ1_A04t16802       ATGGCTTACT CTATGCTCTC CTCCGCCGCT GTTGTTACCT CCCCGGCTCA AGCCACCATG GTCGCTCCAT TCACCGGCTT GAAGTCTTCC TCTGCATTCC CAGTCACCCG CAAGGCCAAC AACGACATTA CCTCCATCGT TAGCAACGGA
BraCCB_A04p11040.1    ATGGCTTACT CTATGCTCTC CTCCGCCGCT GTTGTTACCT CCCCGGCTCA AGCCACCATG GTCGCTCCAT TCACCGGCTT GAAGTCTTCC TCTGCATTCC CAGTCACCCG CAAGGCCAAC AACGACATTA CCTCCATCGT TAGCAACGGA
BraPCA_A04p10870.1    ATGGCTTACT CTATGCTCTC CTCCGCCGCT GTTGTTACCT CCCCGGCTCA AGCCACCATG GTCGCTCCAT TCACCGGCTT GAAGTCTTCC TCTGCATTCC CAGTCACCCG CAAGGCCAAC AACGACATTA CCTCCATCGT TAGCAACGGA
Bjuv_A04_VARUNA_g114  ATGGCTTACT CTATGCTCTC CTCCGCCGCT GTTGTTACCT CCCCGGCTCA AGCCACCATG GTTGCTCCAT TCACCGGCTT GAAGTCTTCC TCTGCATTCC CAGTCACCCG CAAGGCCAAC AACGACATTA CTTCCATCGT TAGCAACGGA
Bjut_A04_new1         ATGGCTTACT CTATGCTCTC CTCCGCCGCT GTTGTTACCT CCCCGGCTCA AGCCACCATG GTTGCTCCAT TCACCGGCTT GAAGTCTTCC TCTGCATTCC CAGTCACCCG CAAGGCCAAC AACGACATTA CTTCCATCGT TAGCAACGGA
BraPCA_A04p10800.1    ATGGCTTCCT CTATGCTCTC CTCCGCAGCT GTGGTTACGT CACCGGCTCA AGCCACCATG GTCGCTCCAT TCACCGGCTT GAAGTCTTCC TCTGCATTCC CAGTCACCCG CAAGGCCAAC AATGACATTA CCTCCATCGT TAGCAACGGA
BraCCB_A04p10960.1    ATGGCTTCCT CTATGCTCTC CTCCGCAGCT GTGGTTACGT CACCGGCTCA AGCCACCATG GTCGCTCCAT TCACCGGCTT GAAGTCTTCC TCTGCATTCC CAGTCACCCG CAAGGCCAAC AATGACATTA CCTCCATCGT TAGCAACGGA
BraZ1_A04t16795       ATGGCTTCCT CTATGCTCTC CTCCGCAGCT GTGGTTACGT CACCGGCTCA AGCCACCATG GTCGCTCCAT TCACCGGCTT GAAGTCTTCC TCTGCATTCC CAGTCACCCG CAAGGCCAAC AATGACATTA CCTCCATCGT TAGCAACGGA
BraTUE_A04p11940.1    ATGGCTTCCT CTATGCTCTC CTCCGCTGCT GTGGTTACCT CACCGGCTCA AGCCACCATG GTCGCTCCAT TCACCGGCTT GAAGTCTTCC TCTGCATTCC CAGTCACCCG CAAGGCCAAC AAAGACATTA CCTCCATCGT TAGCAACGGA
Bjut_O011420          ATGGCTTCCT CTATGCTCTC CTCCGCTGCT GTGGTTACCT CACCGGCTCA AGCCACCATG GTCGCTCCAT TCACCGGCTT GAAGTCTTCC TCTGCATTCC CAGTCACCCG CAAGGCCAAC AAAGACATTA CATCCATCGT TAGCAACGGA
Bjuv_A04_VARUNA_g114  ATGGCTTCCT CTATGCTCTC CTCCGCTGCT GTGGTTACCT CACCGGCTCA AGCCACCATG GTCGCTCCAT TCACCGGCTT GAAGTCTTCC TCTGCATTCC CAGTCACCCG CAAGGCCAAC AATGACATTA CCTCCATCGT TAGCAACGGA
BniN100_B01g029450.2  ATGGCGTCCT CTATGCTCTC CTCCGCCGCT GTGGTTACCT CCCCGGCTCA AGCCACCATG GTCGCTCCAT TCACCGGCTT GAAGTCTTCC TCTGCATTCC CAGTCACCCG CAAGGCCAAC AGTGACATTA CTTCCATCAC TAGCAACGGA
BniC2_B01g032930.1    ATGGCGTCCT CTATGCTCTC CTCCGCCGCT GTGGTTACCT CCCCGGCTCA AGCCACCATG GTCGCTCCAT TCACCGGCTT GAAGTCTTCC TCTGCATTCC CAGTCACCCG CAAGGCCAAC AGTGACATTA CTTCCATCAC TAGCAACGGA
Bjuv_B04_VARUNA_g244  ATGGCGTCCT CTATGCTCTC CTCCGCCGCT GTGGTTACCT CCCCGGCTCA AGCCACCATG GTCGCTCCAT TCACCGGCTT GAAGTCTTCC TCTGCATTCC CAGTCACCCG CAAGGCCAAC AGTGACATTA CTTCCATCAC TAGCAACGGA
Bjut_O001832          ATGGCGTCCT CTATGCTCTC CTCCGCCGCT GTGGTTACCT CCCCGGCTCA AGCCACCATG GTCGCTCCAT TCACCGGCTT GAAGTCTTCC TCTGCATTCC CAGTCACCCG CAAGGCCAAC AGTGACATTA CTTCCATCAC TAGCAACGGA
BraPCA_A04p10780.1    ATGGCTTCCT CTATGCTTTC CTCCGCTGCT GTGGTTACCT CCCCGGCTCA AGCCACCATG GTCGCTCCAT TCACCGGCTT GAAGTCATCC GCTGCATTCC CAGTCACCCG CAAAGCAAAC AACGACATTA CTTCCATTGC AAGCAACGGA
BraCCB_A04p10950.1    ATGGCTTCCT CTATGCTTTC CTCCGCTGCT GTGGTTACCT CCCCGGCTCA AGCCACCATG GTCGCTCCAT TCACCGGCTT GAAGTCATCC GCTGCATTCC CAGTCACCCG CAAAGCAAAC AACGACATTA CTTCCATTGC AAGCAACGGA
BraZ1_A04t16794       ATGGCTTCCT CTATGCTTTC CTCCGCTGCT GTGGTTACCT CCCCGGCTCA AGCCACCATG GTCGCTCCAT TCACCGGCTT GAAGTCATCC GCTGCATTCC CAGTCACCCG CAAAGCAAAC AACGACATTA CTTCCATTGC AAGCAACGGA
BraTUE_A04p11960.1    ATGGCTTCCT CTATGCTTTC CTCCGCTGCT GTGGTTACCT CCCCGGCTCA AGCCACCATG GTCGCTCCAT TCACCGGCTT GAAGTCATCC GCTGCATTCC CAGTCACCCG CAAAGCAAAC AACGACATTA CTTCCATTGC AAGCAACGGA
Bjut_O011421          ATGGCTTCCT CTATGCTTTC CTCCGCTGCT GTGGTTACCT CCCCGGCTCA AGCCACCATG GTCGCTCCAT TCACCGGCTT GAAGTCATCC GCTGCATTCC CAGTCACCCG CAAAGCAAAC AACGACATTA CTTCCATTGC AAGCAACGGA
Bjuv_A04_VARUNA_g114  ATGGCTTCCT CTATGCTTTC CTCCGCTGCT GTGGTTACCT CCCCGGCTCA AGCCACCATG GTCGCTCCAT TCACCGGCTT GAAGTCATCC GCTGCATTCC CAGTCACCCG CAAAGCAAAC AACGACATTA CTTCCATTGC AAGCAACGGA
BniN100_B01g029420.2  ATGGCTTCCT CTATGCTCTC CTCCGCCGCT GTGGTTACCT CACCGGCTCA AGCCACCATG GTCGCTCCAT TCACCGGCTT GAAGTCATCC GCTGCATTCC CAGTCACCCG CAAAGCAAAC AACGACATTA CTTCCATCGC AAGCAACGGA
BniC2_B01g032900.1    ATGGCTTCCT CTATGCTCTC CTCCGCCGCT GTGGTTACCT CACCGGCTCA AGCCACCATG GTCGCTCCAT TCACCGGCTT GAAGTCATCC GCTGCATTCC CAGTCACCCG CAACGCAAAC AACGACATTA CTTCCATCGC AAGCAACGGA
Bjut_O4471_new1       ATGGCTTCCT CTATGCTCTC CTCCGCCGCT GTGGTTACCT CACCGGCTCA AGCCACCATG GTCGCTCCAT TCACCGGCTT GAAGTCATCC GCTGCATTCC CAGTCACCCG CAAAGCAAAC AACGACATTA CTTCCATCGC AAGCAACGGA
Bjuv_B04_VARUNA_g244  ATGGCTTCCT CTATGCTCTC CTCCGCCGCT GTGGTTACCT CACCGGCTCA AGCCACCATG GTCGCTCCAT TCACCGGCTT GAAGTCATCC GCTGCATTCC CAGTCACCCG CAAAGCAAAC AACGACATTA CTTCCATCGC AAGCAACGGA
BniC2_B01g032690.1    ATGGCTTACT CTATGCTCTC CTCCGCCGCT GTTGTTACCT CACCGGCTCA AGCCACCATG GTCGCTCCAT TTACCGGTTT GAAGTCATCC GCTGCATTCC CAGCCACCCG CAAGGCCAAC AACGACATTA CTTCCATCGC AAGCAACGGA
BniN100_B01g029210.2  ATGGCTTACT CTATGCTCTC CTCCGCCGCT GTTGTTACCT CACCGGCTCA AGCCACCATG GTCGCTCCAT TTACCGGTTT GAAGTCATCC GCTGCATTCC CAGCCACCCG CAAGGCCAAC AACGACATTA CTTCCATCGC AAGCAACGGA
BniC2_B01g032670.1    ATGGCTTACT CTATGCTCTC CTCCGCCGCT GTTGTTACCT CACCGGCTCA AGCCACCATG GTCGCTCCAT TCACCGGTTT GAAGTCATCC GCTGCATTCC CAGCCACCCG CAAGGCCAAC AACGACATTA CTTCCATCGC AAGCAACGGA
Bjuv_B04_VARUNA_g243  ATGGCTTACT CTATGCTCTC CTCCGCCGCT GTTGTTACCT CACCGGCTCA AGCCACCATG GTCGCTCCAT TTACCGGTTT GAAGTCATCC GCTGCATTCC CAGCCACCCG CAAGGCCAAC AACGACATTA CTTCCATCGC AAGCAACGGA
Bjut_O012439          ATGGCTTACT CTATGCTCTC CTCCGCCGCT GTTGTTACCT CACCGGCTCA AGCCACCATG GTCGCTCCAT TTACCGGTTT GAAGTCATCC GCTGCATTCC CAGCCACCCG CAAGGCCAAC AACGACATTA CTTCCATCGC AAGCAACGGA

                        155        165        175        185        195        205        215        225        235        245        255        265        275        285        295
BraTUE_A04p11990.1    GGAAGAGTTA GCTGCATGAA G---GTAAAT GTCACTAGAG AGTCTAGAGC TAGGCTTGTC TTTTATTACA CGCAACTGGT AGATTCGAAC AGAATCCTAT TCAAAAGTAC ATATATAGAT ACCTAGAATC GTTAAACAGT ATATACAAAA
BraZ1_A04t16802       GGAAGAGTTA GCTGCATGAA G---GTAAAT GTCACTAGAG AGTCTAGAGC TAGGCTTGTC TTTTATTACA CGCAACTGGT AGATTCGAAC AGAATCCTAT TCAAAAGTGA TACCTAGAAC CGTTAAAAGG TCTAAACAGT ATATACAAAA
BraCCB_A04p11040.1    GGAAGAGTTA GCTGCATGAA G---GTAAAT GTCACTAGAG AGTCTAGAGC TAGGCTTGTC TTTTATTACA CGCAACTGGT AGATTCGAAC AGAATCCTAT TCAAAAGTGA TACCTAGAAC CGTTAAAAGG TCTAAACAGT ATATACAAAA
BraPCA_A04p10870.1    GGAAGAGTTA GCTGCATGAA G---GTAAAT GTCACTAGAG AGTCTAGAGC TAGGCTTGTC TTTTATTACA CGCAACTGGT AGATTCGAAC AGAATCCTAT TCAAAAGTGA TACCTAGAAC CGTTAAAAGG TCTAAACAGT ATATACAAAA
Bjuv_A04_VARUNA_g114  GGAAGAGTTA GCTGCATGAA G---GTAAAT GTCACTAGAG AGTCTAGAGC TAGGCTTGTC TTTTATTACA CGCAACTGGT AGATTCGAAC AGAATCCTAT TCAAAAGTAC ATATATAGAT ACCTAGAATC GTTAAACAGT ATATACAAAA
Bjut_A04_new1         GGAAGAGTTA GCTGCATGAA G---GTAAAT GTCACTAGAG AGTCTAGAGC TAGGCTTGTC TTTTATTACA CGCAACTGGT AGATTCGAAC AGAATCCTAT TCAAAAGTAC ATATATAGAT ACCTAGAATC GTTAAACAGT ATATACAAAA
BraPCA_A04p10800.1    GGAAGAGTTA GCTGCATGAA G---GTAAAT GTCACTAGAG AGTCTAGAGC TAGGCTTGTC TTTTATTACA CGCAACTGGT AGATTCGAAC AGAATCCTAT TCAAAAGTAC ATTTAGATAC CTAGAATCGT TAAACAGTAT ATACAAAAGT
BraCCB_A04p10960.1    GGAAGAGTTA GCTGCATGAA G---GTAAAT GTCACTAGAG AGTCTAGAGC TAGGCTTGTC TTTTATTACA CGCAACTGGT AGATTCGAAC AGAATCCTAT TCAAAAGTAC ATTTAGATAC CTAGAATCGT TAAACAGTAT ATACAAAAGT
BraZ1_A04t16795       GGAAGAGTTA GCTGCATGAA G---GTAAAT GTCACTAGAG AGTCTAGAGC TAGGCTTGTC TTTTATTACA CGCAACTGGT AGATTCGAAC AGAATCCTAT TCAAAAGTAC ATTTAGATAC CTAGAATCGT TAAACAGTAT ATACAAAAGT
BraTUE_A04p11940.1    GGAAGAGTTA GCTGCATGAA G---GTAAAT GTCACTAGAG AGTCTAGAGC TAGGCTTGTC TTTTATTACA CGCAACTGGT AGATTCGAAC AGAATCCTAT TCAAAAGTAC ATATAGAGAT ACCTAGAATC GTTAAACAGT ATATACAAAA
Bjut_O011420          GGAAGAGTTA GCTGCATGAA G---GTAAAT GTCACTAGAG AGTCTAGAGC TAGGCTTGTC TTTTATTACA CGCAACTGGT AGATTCGAAC AGAATCCTAT TCAAAAGTAC ATATAGAGAT ACCTAGAATC GTTAAACAGT ATATACAAAA
Bjuv_A04_VARUNA_g114  GGAAGAGTTA GCTGCATGAA G---GTAAAT GTCACTAGAG AGTCTAGAGC TAGGCTTGTC TTTTATTACA CGCAACTGGT AGATTCGAAC AGAATCCTAT TCAAAAGTAC ATATAGAGAT ACCTAGAATC GTTAAACAGT ATATACAAAA
BniN100_B01g029450.2  GGAAAAGTTA GCTGCATGAA G---GTAAAT TTCACTAGAA TAATTGTAGG CATGTCTTTT ATTACATGCA ACTTGTAAAT TTGAACATGA ATCCTATTCA AAAATGTATA TAACATATCT AGGAATTAAA AAAAAAAGTG TAAACAATAT
BniC2_B01g032930.1    GGAAAAGTTA GCTGCATGAA G---GTAAAT TTCACTAGAA TAATTGTAGG CATGTCTTTT ATTACATGCA ACTTGTAAAT TTGAACATGA ATCCTATTCA AAAATGTATA TAACATATCT AGGAATTAAA AAAAAAAGTG TAAACAATAT
Bjuv_B04_VARUNA_g244  GGAAAAGTTA GCTGCATGAA G---GTAAAT TTCACTAGAA TAATTGTAGG CATGTCTTTT ATTACATGCA ACTTGTAAAT TTGAACATGA ATCCTATTCA AAAATGTATA TAACATATCT AGGAATTAAA AAAAAAAGTG TAAACAATAT
Bjut_O001832          GGAAAAGTTA GCTGCATGAA G---GTAAAT TTCACTAGAA TAATTGTAGG CATGTCTTTT ATTACATGCA ACTTGTAAAT TTGAACATGA ATCCTATTCA AAAATGTATA TAACATATCT AGGAATTAAA AAAAAAAGTG TAAACAATAT
BraPCA_A04p10780.1    GGAAGAGTTA GCTGCATGAA G---GTAAAT ATCTCTAGAA TAATTGCAGG CCTGTCCTTT ATTACACGTA ACTGGTGCAT TCGAACATGA ATCCCATTGA AAAGTGTATA TAACGTACCT AGGATCAATA AATGTCCAAA ACAATATATA
BraCCB_A04p10950.1    GGAAGAGTTA GCTGCATGAA G---GTAAAT ATCTCTAGAA TAATTGCAGG CCTGTCCTTT ATTACACGTA ACTGGTGCAT TCGAACATGA ATCCCATTGA AAAGTGTATA TAACGTACCT AGGATCAATA AATGTCCAAA ACAATATATA
BraZ1_A04t16794       GGAAGAGTTA GCTGCATGAA G---GTAAAT ATCTCTAGAA TAATTGCAGG CCTGTCCTTT ATTACACGTA ACTGGTGCAT TCGAACATGA ATCCCATTGA AAAGTGTATA TAACGTACCT AGGATCAATA AATGTCCAAA ACAATATATA
BraTUE_A04p11960.1    GGAAGAGTTA GCTGCATGAA G---GTAAAT ATCTCTAGAA TAATTGCAGG CCTGTCCTTT ATAACACGTA ACTGGTGCAT TCGAACATGA ATCCCATTGA AAAGTGTATA TAACGTACCT AGGATCAATA AATGTCCAAA ACAATATATA
Bjut_O011421          GGAAGAGTTA GCTGCATGAA G---GTAAAT ATCTCTAGAA TAATTGCAGG CCTGTCCTTT ATTACACGTA ACTGGTGCAT TCGAACATGA ATCCCATTGA AAAGTGTATA TAACGTACCT AGGATCAATT AATGTCCAAA ACAATATATA
Bjuv_A04_VARUNA_g114  GGAAGAGTTA GCTGCATGAA G---GTAAAT ATCTCTAGAA TAATTGCAGG CCTGTCCTTT ATTACACGTA ACTGGTGCAT TCGAACATGA ATCCCATTGA AAAGTGTATA TAACGTACCT AGGATCAATT AATGTCCAAA ACAATATATA
BniN100_B01g029420.2  GGAAGAGTTA GCTGCATGAA G---GTAAAT GTCACCAGAT TAATTGTAGG CCAGTCCTTC ATTTCACGCA ACTGGTGCAT TTGAACATGA ATCCTATTCA AAAAATGTAT ATAACATAGC TAGGATCAAT AAATGTCCAA AAAATATATA
BniC2_B01g032900.1    GGAAGAGTTA GCTGCATGAA G---GTAAAT GTCACCAGAT TAATTGTAGG CCAGTCCTTC ATTTCACGCA ACTGGTGCAT TTGAACATGA ATCCTATTCA AAAAATGTAT ATAACATAGC TAGGATCAAT AAATGTCCAA AAAATATATA
Bjut_O4471_new1       GGAAGAGTTA GCTGCATGAA G---GTAAAT GTCACCAGAT TAATTGTAGG CCAGTCCTTC ATTTCACGCA ACTGGTGCAT TTGAACATGA ATCCTATTCA AAAAATGTAT ATAACATAGC TAGGATCAAT AAATGTCCAA AAAATATATA
Bjuv_B04_VARUNA_g244  GGAAGAGTTA GCTGCATGAA G---GTAAAT GTCACCAGAT TAATTGTAGG CCAGTCCTTC ATTTCACGCA ACTGGTGCAT TTGAACATGA ATCCTATTCA AAAAATGTAT ATAACATAGC TAGGATCAAT AAATGTCCAA AAAATATATA
BniC2_B01g032690.1    GGAAGAGTTA GCTGCATGAA G---GTAAAT GTCACTAGAG AGTATAGAGT TAGGCTTGTC TTTTATTACA CGCAACTGGT AGATTCGAAC AGAATCCTAT TCAAAAGTAC ATATAGATAC CTAGGATCGT TTAAAGGTCT AAACTGTATA
BniN100_B01g029210.2  GGAAGAGTTA GCTGCATGAA G---GTAAAT GTCACTAGAG AGTATAGAGT TAGGCTTGTC TTTTATTACA CGCAACTGGT AGATTCGAAC AGAATCCTAT TCAAAAGTAC ATATAGATAC CTAGGATCGT TTAAAGGTCT AAACTGTATA
BniC2_B01g032670.1    GGAAGAGTTA GCTGCATGAA G---GTAAAT GTCACTAGAG AGTATAGAGT CAGGCTTGTC TTTTATTACA CGCAACTGGT AGATTCGAAC AGAATCCTAT TCAAAAGTAC ATATAGATAC CTATGATCGT TTAAAGGTCT AAACTGTATA
Bjuv_B04_VARUNA_g243  GGAAGAGTTA GCTGCATGAA G---GTAAAT GTCACTAGAG AGTATAGAAT TAGGCTTGTC TTTTATTACA CGCAACTGGT AGATTCGAAC AGAATCCTAT TCAAAAGTAC ATATAGATAC CTAGGATCGT TTAAAGGTCT AAACTGTATA
Bjut_O012439          GGAAGAGTTA GCTGCATGAA G---GTAAAT GTCACTAGAG AGTATAGAAT TAGGCTTGTC TTTTATTACA CGCAACTGGT AGATTCGAAC AGAATCCTAT TCAAAAGTAC ATATAGATAC CTAGGATCGT TTAAAGGTCT AAACTGTATA

                        305        315        325        335        345        355        365        375        385        395        405        415        425        435        445
BraTUE_A04p11990.1    GTGAACTAAT ATTCATTAAT TGGAAATACG TTTCGTAG-- ---------- -GTGTGGCCA CCAGTTGGAA AGAAGAAGTT TGAGACCCTC TCTTACCTTC CTGACCTTAC CGAAGTTGAA TTGGGTAAGG AAGTCGACTA CCTTCTCCGC
BraZ1_A04t16802       GTGAACTAAT ATTCATTAAT TGGAAATACG ATTTGTAG-- ---------- -GTGTGGCCA CCAGTTGGAA AGAAGAAGTT TGAGACCCTC TCTTACCTTC CTGACCTTAC CGAAGTTGAA TTGGGTAAGG AAGTTGACTA CCTTCTCCGC
BraCCB_A04p11040.1    GTGAACTAAT ATTCATTAAT TGGAAATACG ATTTGTAG-- ---------- -GTGTGGCCA CCAGTTGGAA AGAAGAAGTT TGAGACCCTC TCTTACCTTC CTGACCTTAC CGAAGTTGAA TTGGGTAAGG AAGTTGACTA CCTTCTCCGC
BraPCA_A04p10870.1    GTGAACTAAT ATTCATTAAT TGGAAATACG ATTTGTAG-- ---------- -GTGTGGCCA CCAGTTGGAA AGAAGAAGTT TGAGACCCTC TCTTACCTTC CTGACCTTAC CGAAGTTGAA TTGGGTAAGG AAGTTGACTA CCTTCTCCGC
Bjuv_A04_VARUNA_g114  GTGAACTAAT ATTCATTAAT TGGAAATACG TTTCGTAG-- ---------- -GTGTGGCCA CCAGTTGGAA AGAAGAAGTT TGAGACCCTC TCTTACCTTC CTGACCTTAC CGAAGTTGAA TTGGGTAAGG AAGTCGACTA CCTTCTCCGC
Bjut_A04_new1         GTGAACTAAT ATTCATTAAT TGGAAATACG TTTCGTAG-- ---------- -GTGTGGCCA CCAGTTGGAA AGAAGAAGTT TGAGACCCTC TCTTACCTTC CTGACCTTAC CGAAGTTGAA TTGGGTAAGG AAGTAGACTA CCTTCTCCGC
BraPCA_A04p10800.1    GAACTAATAT TCATTAATTG GAAATACGTT TCGTAG---- ---------- -GTGTGGCCA CCAGTTGGAA AGAAGAAGTT TGAGACCCTC TCTTACCTTC CTGACCTTAC CGAAGTTGAA TTGGGTAAGG AAGTCGACTA CCTTCTCCGC
BraCCB_A04p10960.1    GAACTAATAT TCATTAATTG GAAATACGTT TCGTAG---- ---------- -GTGTGGCCA CCAGTTGGAA AGAAGAAGTT TGAGACCCTC TCTTACCTTC CTGACCTTAC CGAAGTTGAA TTGGGTAAGG AAGTCGACTA CCTTCTCCGC
BraZ1_A04t16795       GAACTAATAT TCATTAATTG GAAATACGTT TCGTAG---- ---------- -GTGTGGCCA CCAGTTGGAA AGAAGAAGTT TGAGACCCTC TCTTACCTTC CTGACCTTAC CGAAGTTGAA TTGGGTAAGG AAGTCGACTA CCTTCTCCGC
BraTUE_A04p11940.1    GTGAACTAAT ATTCATTAAT TGGAAATACG TTTCGTAG-- ---------- -GTGTGGCCA CCAGTTGGAA AGAAGAAGTT TGAGACCCTC TCTTACCTTC CTGACCTTAC CGAAGTTGAA TTGGGTAAGG AAGTTGACTA CCTTCTCCGC
Bjut_O011420          GTGAACTAAT ATTCATTAAT TGGAAATACG TTTCGTAG-- ---------- -GTGTGGCCA CCAGTTGGAA AGAAGAAGTT TGAGACCCTC TCTTACCTTC CTGACCTTAC CGAAGTTGAA TTGGGTAAGG AAGTTGACTA CCTTCTCCGC
Bjuv_A04_VARUNA_g114  GTGAACTAAT ATTCATTAAT TGGAAATACG TTTCGTAG-- ---------- -GTGTGGCCA CCAGTTGGAA AGAAGAAGTT TGAGACCCTC TCTTACCTTC CTGACCTTAC CGAAGTTGAA TTGGGTAAGG AAGTTGACTA CCTTCTCCGC
BniN100_B01g029450.2  ATACAAAAGT GAATTAATAT TCATGAAATG GAAATACGAT TTGTAG---- -GTGTGGCCA CCAGTTGGAA AGAAGAAGTT TGAGACCCTC TCTTACCTTC CTGACCTTAC CGATGTTGAA TTGGGTAAGG AAGTTGACTA CCTTCTCCGC
BniC2_B01g032930.1    ATACAAAAGT GAATTAATAT TCATGAAATG GAAATACGAT TTGTAG---- -GTGTGGCCA CCAGTTGGAA AGAAGAAGTT TGAGACCCTC TCTTACCTTC CTGACCTTAC CGATGTTGAA TTGGGTAAGG AAGTTGACTA CCTTCTCCGC
Bjuv_B04_VARUNA_g244  ATACAAAAGT GAATTAATAT TCATGAAATG GAAATACGAT TTGTAG---- -GTGTGGCCA CCAGTTGGAA AGAAGAAGTT TGAGACCCTC TCTTACCTTC CTGACCTTAC CGATGTTGAA TTGGGTAAGG AAGTTGACTA CCTTCTCCGC
Bjut_O001832          ATACAAAAGT GAATTAATAT TCATGAAATG GAAATACGAT TTGTAG---- -GTGTGGCCA CCAGTTGGAA AGAAGAAGTT TGAGACCCTC TCTTACCTTC CTGACCTTAC CGATGTTGAA TTGGGTAAGG AAGTTGACTA CCTTCTCCGC
BraPCA_A04p10780.1    CAAACGTGAC TTAGTATTCA CCAACTGGAA ATGTGATTTA ATTTGTAG-- -GTGTGGCCA CCAGTTGGAA AGAAGAAGTT TGAGACCCTC TCTTACCTTC CTGACCTTAC CGAAGTTGAA TTGGGTAAGG AAGTCGACTA CCTTCTCCGC
BraCCB_A04p10950.1    CAAACGTGAC TTAGTATTCA CCAACTGGAA ATGTGATTTA ATTTGTAG-- -GTGTGGCCA CCAGTTGGAA AGAAGAAGTT TGAGACCCTC TCTTACCTTC CTGACCTTAC CGAAGTTGAA TTGGGTAAGG AAGTCGACTA CCTTCTCCGC
BraZ1_A04t16794       CAAACGTGAC TTAGTATTCA CCAACTGGAA ATGTGATTTA ATTTGTAG-- -GTGTGGCCA CCAGTTGGAA AGAAGAAGTT TGAGACCCTC TCTTACCTTC CTGACCTTAC CGAAGTTGAA TTGGGTAAGG AAGTCGACTA CCTTCTCCGC
BraTUE_A04p11960.1    CAAACGTGAC TTAGTATTCA CCAACTCGAA ATGTGATTTA ATTTGTAG-- -GTGTGGCCA CCAGTTGGAA AGAAGAAGTT TGAGACCCTC TCTTACCTTC CTGACCTTAC CGAAGTTGAA TTGGGTAAGG AAGTCGACTA CCTTCTCCGC
Bjut_O011421          CAAACGTGAC TTAGTATTCA CCAACTGGAA ATGTGATTTC ATTTGTAG-- -GTGTGGCCA CCAGTTGGAA AGAAGAAGTT TGAGACCCTC TCTTACCTTC CTGACCTTAC CGAAGTTGAA TTGGGTAAGG AAGTCGACTA CCTTCTCCGC
Bjuv_A04_VARUNA_g114  CAAACGTGAC TTAGTATTCA CCAACTGGAA ATGTGATTTC ATTTGTAG-- -GTGTGGCCA CCAGTTGGAA AGAAGAAGTT TGAGACCCTC TCTTACCTTC CTGACCTTAC CGAAGTTGAA TTGGGTAAGG AAGTCGACTA CCTTCTCCGC
BniN100_B01g029420.2  CAAAGGTCAA TTAGTATTCA CGAACTGGAA ATGCAATTTA TAG------- -GTATGGCCA CCAGTTGGAA AGAAGAAGTT TGAGACTCTC TCTTACCTTC CTGACCTTAC CGATGTTGAA TTGGGTAAGG AAGTTGACTA CCTTCTCCGC
BniC2_B01g032900.1    CAAAGGTCAA TTAGTATTCA CGAACTGGAA ATGCAATTTA TAG------- -GTATGGCCA CCAGTTGGAA AGAAGAAGTT TGAGACTCTC TCTTACCTTC CTGACCTTAC CGATGTTGAA TTGGGTAAGG AAGTTGACTA CCTTCTCCGC
Bjut_O4471_new1       CAAAGGTCAA TTAGTATTCA CGAACTGGAA ATGCAATTTA TAG------- -GTATGGCCA CCAGTTGGAA AGAAGAAGTT TGAGACTCTC TCTTACCTTC CTGACCTTAC CGATGTTGAA TTGGGTAAGG AAGTTGACTA CCTTCTCCGC
Bjuv_B04_VARUNA_g244  CAAAGGTCAA TTAGTATTCA CGAACTGGAA ATGCAATTTA TAG------- -GTATGGCCA CCAGTTGGAA AGAAGAAGTT TGAGACTCTC TCTTACCTTC CTGACCTTAC CGATGTTGAA TTGGGTAAGG AAGTTGACTA CCTTCTCCGC
BniC2_B01g032690.1    TACGAAAGTG AACTATAATA TTCATTAATT GGAAATACGA TTTGTAG--- -GTGTGGCCA CCAATTGGAA AGAAGAAGTT TGAGACCCTC TCTTACCTTC CTGACCTTAC CGATGTTGAA TTGGGAAAGG AAGTTGACTA CCTTCTCCGC
BniN100_B01g029210.2  TACGAAAGTG AACTATAATA TTCATTAATT GGAAATACGA TTTGTAG--- -GTGTGGCCA CCAATTGGAA AGAAGAAGTT TGAGACCCTC TCTTACCTTC CTGACCTTAC CGATGTTGAA TTGGGAAAGG AAGTTGACTA CCTTCTCCGC
BniC2_B01g032670.1    TACGAAAGTG AACTATAATA TTCATTAATT GGAAATACGA TTTGTAG--- -GTGTGGCCA CCAATTGGAA AGAAGAAGTT TGAGACCCTC TCTTACCTTC CTGACCTTAC CGATGTTGAA TTGGGAAAGG AAGTTGACTA CCTTCTCCGC
Bjuv_B04_VARUNA_g243  TACGAAAGTG AACTATAATA TTCATTAATT GGAAATACGA TTTGTAG--- -GTGTGGCCA CCAATTGGAA AGAAGAAGTT TGAGACCCTC TCTTACCTTC CTGACCTTAC CGATGTTGAA TTGGGAAAGG AAGTTGACTA CCTTCTCCGC
Bjut_O012439          TACGAAAGTG AACTATAATA TTCATTAATT GGAAATACGA TTTGTAG--- -GTGTGGCCA CCAATTGGAA AGAAGAAGTT TGAGACCCTC TCTTACCTTC CTGACCTTAC CGATGTTGAA TTGGGAAAGG AAGTTGACTA CCTTCTCCGC
```

```
                        455        465        475        485        495        505        515        525        535        545        555        565        575        585        595
BraTUE_A04p11990.1      AACAAGTGGA TTCCTTGTGT TGAATTCGAG TTGGAG---G TAATAAAAAC AGAAGTTTTG TTTTCTTGGA TCTAAACATC TTTTATCACA TAATCTCAAT CCTGATTTTG ATTGGAATTC TCATGCGTGT GTAG----CA CGGGTTTGTT
BraZ1_A04t16802         AACAAGTGGA TTCCTTGTGT TGAATTCGAG TTGGAG---G TAATAAAAAT AGAACTTTTG TTTTCTTGGA TCTAAACATC TTTTATCACA TAATCTTAAT CCTGATTTTG ATTGGAATTC TCATGCGTGT GTAG----CA CGGATTTGTT
BraCCB_A04p11040.1      AACAAGTGGA TTCCTTGTGT TGAATTCGAG TTGGAG---G TAATAAAAAT AGAACTTTTG TTTTCTTGGA TCTAAACATC TTTTATCACA TAATCTTAAT CCTGATTTTG ATTGGAATTC TCATGCGTGT GTAG----CA CGGATTTGTT
BraPCA_A04p10870.1      AACAAGTGGA TTCCTTGTGT TGAATTCGAG TTGGAG---G TAATAAAAAT AGAACTTTTG TTTTCTTGGA TCTAAACATC TTTTATCACA TAATCTTAAT CCTGATTTTG ATTGGAATTC TCATGCGTGT GTAG----CA CGGATTTGTT
Bjuv_A04_VARUNA_g114    AACAAGTGGA TTCCTTGTGT TGAATTCGAG TTGGAG---G TAATAAAAAT AGAAGTTTCT TTTTTGGATC TAAACATCTT TTATCACATA ATCTCAATCC TGATTTTGAT TGGAATTCTC ACGTGTGTGT AG------CA CGGGTTTGTT
Bjut_A04_new1           AACAAGTGGA TTCCTTGTGT TGAATTCGAG TTGGAG---G TAATAAAAAT AGAAGTTTCT TTTTTGGATC TAAACATCTT TTATCACATA ATCTCAATCC TGATTTTGAT TGGAATTCTC ACGTGTGTGT AG------CA CGGGTTTGTT
BraPCA_A04p10800.1      AACAAGTGGA TTCCTTGTGT TGAATTCGAG TTGGAG---G TAATAAAAAC AGAAGTTTTG TTTTATGGAT CTAAACATCT TTTATCACAT AATCTCAATC CTGATTTTGA TTGGAATTCT CACGTGTGTG TAG-----CA CGGGTTTGTT
BraCCB_A04p10960.1      AACAAGTGGA TTCCTTGTGT TGAATTCGAG TTGGAG---G TAATAAAAAC AGAAGTTTTG TTTTATGGAT CTAAACATCT TTTATCACAT AATCTCAATC CTGATTTTGA TTGGAATTCT CACGTGTGTG TAG-----CA CGGGTTTGTT
BraZ1_A04t16795         AACAAGTGGA TTCCTTGTGT TGAATTCGAG TTGGAG---G TAATAAAAAC AGAAGTTTTG TTTTATGGAT CTAAACATCT TTTATCACAT AATCTCAATC CTGATTTTGA TTGGAATTCT CACGTGTGTG TAG-----CA CGGGTTTGTT
BraTUE_A04p11940.1      AACAAGTGGA TTCCTTGTGT TGAATTCGAG TTGGAG---G TAATAAAATA GAAGTTTTTT TTTGGATCTA AACATCTTTT ATCACATAAT CTCAATCCTG ATTTTGATTG GAATTCTCAT GCGTATGTAG --------CA CGGGTTTGTT
Bjut_O011420            AACAAGTGGA TTCCTTGTGT TGAATTCGAG TTGGAG---G TAATAAAATA GAAGTTTTTT TTGGATCTAA ACATCTTTTA TCACATAATC TCAATCCTGA TTTTGATTGG AATTCTCATG TGTGTGTAG- --------CA CGGATTTGTG
Bjuv_A04_VARUNA_g114    AACAAGTGGA TTCCTTGTGT TGAATTCGAG TTGGAG---G TAATAAAATA GAAGTTTTTT TTGGATCTAA ACATCTTTTA TCACATAATC TCAATCCTGA TTTTGATTGG AATTCTCATG TGTGTGTAG- --------CA CGGATTTGTG
BniN100_B01g029450.2    AACAAGTGGA TTCCTTGTGT TGAATTCGAG TTGGAG---G TAATAAAAAC AGAAGTTCTG TTTCCATGGA TCTAAACATC TTTTATGACA CAATGCCAAT CCTGATTTTG ATTGGAATTC ACATGTGTGT ATAG----CA CGGATTTGTG
BniC2_B01g032930.1      AACAAGTGGA TTCCTTGTGT TGAATTCGAG TTGGAG---G TAATAAAAAC AGAAGTTCTG TTTCCATGGA TCTAAACATC TTTTATGACA CAATGCCAAT CCTGATTTTG ATTGGAATTC ACATGTGTGT ATAG----CA CGGATTTGTG
Bjuv_B04_VARUNA_g244    AACAAGTGGA TTCCTTGTGT TGAATTCGAG TTGGAG---G TAATAAAAAC AGAAGTTCTG TTTCCATGGA TCTAAACATC TTTTATGACA CAATGCCAAT CCTGATTTTG ATTGGAATTC ACATGTGTGT ATAG----CA CGGATTTGTG
Bjut_O001832            AACAAGTGGA TTCCTTGTGT TGAATTCGAG TTGGAG---G TAATAAAAAC AGAAGTTTTG TTTCCTTGGA TTTAATCATC TTTTATTACT CAATGTCAAT CCTGATTTTG ATTGGAATTC ACATGTGTGT ATAG----CA CGGATTTGTG
BraPCA_A04p10780.1      AACAAGTGGA TTCCTTGTGT TGAATTCGAG TTGGAG---G TAATAAAAAC AGAAGTTTTG TTTTTTGGAT CTAAACATCT TTTATCACAT AATCTCAATC CTGATTTTGA TTGGAATTCT CACGTGTGTG TAG-----CA CGGGTTTGTT
BraCCB_A04p10950.1      AACAAGTGGA TTCCTTGTGT TGAATTCGAG TTGGAG---G TAATAAAAAC AGAAGTTTTG TTTTTTGGAT CTAAACATCT TTTATCACAT AATCTCAATC CTGATTTTGA TTGGAATTCT CACGTGTGTG TAG-----CA CGGGTTTGTT
BraZ1_A04t16794         AACAAGTGGA TTCCTTGTGT TGAATTCGAG TTGGAG---G TAATAAAAAC AGAAGTTTTG TTTTTTGGAT CTAAACATCT TTTATCACAT AATCTCAATC CTGATTTTGA TTGGAATTCT CACGTGTGTG TAG-----CA CGGGTTTGTT
BraTUE_A04p11960.1      AACAAGTGGA TTCCTTGTGT TGAATTCGAG TTGGAG---G TAATAAAACT AGAAGTTTTT TTTGGATCTA AACATCTTTT ATCACATAAT CTCAATCCTG ATTTTGATTG GAATTCTCAT GCGTGTGTAG --------CA CGGATTTGTG
Bjut_O011421            AACAAGTGGA TTCCTTGTGT TGAATTCGAG TTGGAG---G TAATAAAAAC AGAAGTTTTG TTTCCTTGGA TTTAATCATC TTTTATTACG CAATGTCAAT CCTGATTTTG ATTGGAATTG TCATGTGTGT ATAG----CA CGGATTTGTG
Bjuv_A04_VARUNA_g114    AACAAGTGGA TTCCTTGTGT TGAATTCGAG TTGGAG---G TAATAAAAAT AGAAGTTTTT TTTGGATCTA AACATCTTTT ATCACATAAT CTCAATCCTG ATTTTGATTG GAATTCTCAT GTGTGTGTAG --------CA CGGATTTGTG
BniN100_B01g029420.2    AACAAGTGGA TTCCTTGTGT TGAATTCGAG TTGGAG---G TAATAAAAAC AGAAGTTTTG CTTCCTTGGA TTTAATCATC TTCTATTACG CAATGTCAAT CCTGATTTTG ATTGGAATTG TCATGTGTAT ACAG----CA CGGATTTGTC
BniC2_B01g032900.1      AACAAGTGGA TTCCTTGTGT TGAATTCGAG TTGGAG---G TAATAAAAAC AGAAGTTTTG CTTCCTTGGA TTTAATCATC TTCTATTACG CAATGTCAAT CCTGATTTTG ATTGGAATTG TCATGTGTAT ACAG----CA CGGATTTGTC
Bjut_O4471_new1         AACAAGTGGA TTCCTTGTGT TGAATTCGAG TTGGAG---G TAATAAAAAC AGAAGTTTTG TTTCCTTGGA TTTAATCATC TTTTATTACT CAATGTCAAT CCTGATTTTG ATTGGAATTT TCATGTGTTT ATAG----CA CGGATTTGTT
Bjuv_B04_VARUNA_g244    AACAAGTGGA TTCCTTGTGT TGAATTCGAG TTGGAG---G TAATAAAAAC AGAAGTTTTG CTTCCTTGGA TTTAATCATC TTCTATTACG CAATGTCAAT CCTGATTTTG ATTGGAATTG TCATGTGTAT ACAG----CA CGGATTTGTC
BniC2_B01g032690.1      AACAAGTGGA TTCCTTGTGT TGAATTCGAG TTGGAG---G TAATAAAAAC AGAAGTTTTG CTTCCTTGGA TTTAATCATC TTTTATTACG CAATGTCAAT CCTGATTTTG ATTGGAATTC TCATGTGTAT ACAG----CA CGGATTTGTC
BniN100_B01g029210.2    AACAAGTGGA TTCCTTGTGT TGAATTCGAG TTGGAG---G TAATAAAAAC AGAAGTTTTG CTTCCTTGGA TTTAATCATC TTTTATTACG CAATGTCAAT CCTGATTTTG ATTGGAATTC TCATGTGTAT ACAG----CA CGGATTTGTC
BniC2_B01g032670.1      AACAAGTGGA TTCCTTGTGT TGAATTCGAG TTGGAG---G TAATAAAAAC AGAAGTTTTG CTTCCTTGGA TTTAATCATC TTTTATTACG CAATGTCAAT CCTGATTTTG ATTGGAATTC TCATGTGTAT ACAG----CA CGGATTTGTC
Bjuv_B04_VARUNA_g243    AACAAGTGGA TTCCTTGTGT TGAATTCGAG TTGGAG---G TAATAAAAAC AGAAGTTTTG CTTCCTTGGA TTTAATCATC TTTTATTACG CAATGTCAAT CCTGATTTTG ATTGGAATTC TCATGTGTAT ACAG----CA CGGATTTGTC
Bjut_O012439            AACAAGTGGA TTCCTTGTGT TGAATTCGAG TTGGAG---G TAATAAAAAC AGAAGTTTTG CTTCCTTGGA TTTAATCATC TTTTATTACG CAATGTCAAT CCTGATTTTG ATTGGAATTC TCATGTGTAT ACAG----CA CGGATTTGTC

                        605        615        625        635        645        655        665        675        685        695        705        715        725        735        745
BraTUE_A04p11990.1      TACCGTGAGC ACGGAAGCAC CCCCGGATAC TACGATGGAC GTTACTGGAC AATGTGGAAG CTTCCCTTGT TCGGATGCAC TGATTCTGCT CAAGTGTTGA AGGAAGTCCA AGAGTGCAAA ACGGAGTACC CTAACGCCTT CATCAGGATC
BraZ1_A04t16802         TACCGTGAGC ACGGAAGCAC CCCCGGATAC TACGATGGAC GTTACTGGAC AATGTGGAAG CTTCCCTTGT TCGGATGCAC CGACTCTGCT CAAGTGTTGA AGGAAGTCCA AGAGTGCAAA ACGGAGTACC CTAACGCCTT CATCAGGATC
BraCCB_A04p11040.1      TACCGTGAGC ACGGAAGCAC CCCCGGATAC TACGATGGAC GTTACTGGAC AATGTGGAAG CTTCCCTTGT TCGGATGCAC CGACTCTGCT CAAGTGTTGA AGGAAGTCCA AGAGTGCAAA ACGGAGTACC CTAACGCCTT CATCAGGATC
BraPCA_A04p10870.1      TACCGTGAGC ACGGAAGCAC CCCCGGATAC TACGATGGAC GTTACTGGAC AATGTGGAAG CTTCCCTTGT TCGGATGCAC CGACTCTGCT CAAGTGTTGA AGGAAGTCCA AGAGTGCAAA ACGGAGTACC CTAACGCCTT CATCAGGATC
Bjuv_A04_VARUNA_g114    TACCGTGAGC ATGGAAGCAC CCCCGGATAC TACGATGGAC GTTACTGGAC AATGTGGAAG CTTCCCTTGT TCGGATGCAC CGACTCTGCT CAAGTGTTGA AGGAAGTCCA AGAGTGCAAA ACGGAGTACC CTAACGCCTT CATCAGGATC
Bjut_A04_new1           TACCGTGAGC ATGGAAGCAC CCCCGGATAC TACGATGGAC GTTACTGGAC AATGTGGAAG CTTCCCTTGT TCGGATGCAC AGACTCTGCT CAAGTGTTGA AGGAAGTCCA AGAGTGCAAA ACGGAGTACC CTAACGCCTT CATCAGGATC
BraPCA_A04p10800.1      TACCGTGAGC ACGGAAGCAC CCCCGGATAC TACGATGGAC GTTACTGGAC AATGTGGAAG CTTCCCTTGT TCGGATGCAC TGACTCTGCT CAAGTGTTGA AGGAAGTCCA AGAGTGCAAA ACGGAGTACC CTAACGCCTT CATCAGGATC
BraCCB_A04p10960.1      TACCGTGAGC ACGGAAGCAC CCCCGGATAC TACGATGGAC GTTACTGGAC AATGTGGAAG CTTCCCTTGT TCGGATGCAC TGACTCTGCT CAAGTGTTGA AGGAAGTCCA AGAGTGCAAA ACGGAGTACC CTAACGCCTT CATCAGGATC
BraZ1_A04t16795         TACCGTGAGC ACGGAAGCAC CCCCGGATAC TACGATGGAC GTTACTGGAC AATGTGGAAG CTTCCCTTGT TCGGATGCAC TGACTCTGCT CAAGTGTTGA AGGAAGTCCA AGAGTGCAAA ACGGAGTACC CTAACGCCTT CATCAGGATC
BraTUE_A04p11940.1      TACCGTGAGC ACGGAAGCAC CCCCGGATAC TACGATGGCC GTTACTGGAC AATGTGGAAG CTTCCCTTGT TCGGATGCAC CGACTCTGCT CAAGTGTTGA AAGAAGTCCA AGAGTGCAAA ACGGAGTACC CTAACGCCTT CATCAGGATC
Bjut_O011420            TACCGTGAGC AAGGAAGCAC CCCCGGATAC TACGATGGCC GTTACTGGAC AATGTGGAAG CTTCCCTTGT TCGGATGCAC AGACTCTGCT CAAGTGTTGA AAGAAGTCCA AGAGTGCAAA ACGGAGTACC CTAACGCCTT CATCAGGATC
Bjuv_A04_VARUNA_g114    TACCGTGAGC ATGGAAGCAC CCCCGGATAC TACGATGGCC GTTACTGGAC AATGTGGAAG CTTCCCTTGT TCGGATGCAC CGACTCTGCT CAAGTGTTGA AAGAAGTCCA AGAGTGCAAA ACGGAGTACC CTAACGCCTT CATCAGGATC
BniN100_B01g029450.2    TACCGTGAGC ACGGAAGCAC ACCCGGATAC TACGATGGCC GTTACTGGAC AATGTGGAAG CTTCCCTTGT TCGGATGCAC TGACTCTGCT CAGGTGTTGA AGGAAGTCCA AGAGTGCAAA ACAGAGTACC CTAACGCCTT CATCAGAATC
BniC2_B01g032930.1      TACCGTGAGC ACGGAAGCAC ACCCGGATAC TACGATGGCC GTTACTGGAC AATGTGGAAG CTTCCCTTGT TCGGATGCAC TGACTCTGCT CAGGTGTTGA AGGAAGTCCA AGAGTGCAAA ACAGAGTACC CTAACGCCTT CATCAGAATC
Bjuv_B04_VARUNA_g244    TACCGTGAGC ACGGAAGCAC ACCCGGATAC TACGATGGCC GTTACTGGAC AATGTGGAAG CTTCCCTTGT TCGGATGCAC TGACTCTGCT CAGGTGTTGA AGGAAGTCCA AGAGTGCAAA ACAGAGTACC CTAACGCCTT CATCAGAATC
Bjut_O001832            TACCGTGAGC ACGGAAGCAC ACCCGGATAC TACGATGGCC GTTACTGGAC AATGTGGAAG CTTCCCTTGT TCGGATGCAC TGACTCTGCT CAGGTGTTGA AGGAAGTCCA AGAGTGCAAA ACAGAGTACC CTAACGCCTT CATCAGAATC
BraPCA_A04p10780.1      TACCGTGAGC ACGGAAGCAC CCCCGGATAC TACGATGGCC GTTACTGGAC AATGTGGAAG CTTCCCTTGT TCGGATGCAC TGACTCTGCT CAAGTGTTGA AGGAAGTCCA AGAATGCAAA ACGGAGTACC CTAACGCTTT CATCAGGATC
BraCCB_A04p10950.1      TACCGTGAGC ACGGAAGCAC CCCCGGATAC TACGATGGCC GTTACTGGAC AATGTGGAAG CTTCCCTTGT TCGGATGCAC TGACTCTGCT CAAGTGTTGA AGGAAGTCCA AGAATGCAAA ACGGAGTACC CTAACGCTTT CATCAGGATC
BraZ1_A04t16794         TACCGTGAGC ACGGAAGCAC CCCCGGATAC TACGATGGCC GTTACTGGAC AATGTGGAAG CTTCCCTTGT TCGGATGCAC TGACTCTGCT CAAGTGTTGA AGGAAGTCCA AGAATGCAAA ACGGAGTACC CTAACGCTTT CATCAGGATC
BraTUE_A04p11960.1      TACCGTGAGC ACGGAAGCAC CCCCGGATAC TACGATGGCC GTTACTGGAC AATGTGGAAG CTTCCCTTGT TCGGATGCAC TGACTCTGCT CAAGTGTTGA AGGAAGTCCA AGAGTGCAAA ACGGAGTACC CTAACGCCTT CATCAGGATC
Bjut_O011421            TACCGTGAGC ACGGAAGCAC ACCCGGATAC TACGATGGCC GTTACTGGAC AATGTGGAAG CTTCCCTTGT TCGGATGCAC TGACTCTGCT CAGGTGTTGA AGGAAGTCCA AGAGTGCAAA ACAGAGTACC CTAACGCCTT CATCAGAATC
Bjuv_A04_VARUNA_g114    TACCGTGAGC ACGGAAGCAC CCCCGGATAC TACGATGGCC GTTACTGGAC AATGTGGAAG CTTCCCTTGT TCGGATGCAC TGACTCTGCT CAAGTGTTGA AGGAAGTCCA AGAGTGCAAA ACGGAGTACC CTAACGCCTT CATCAGGATC
BniN100_B01g029420.2    TACCGTGAGC ACGGAAGCAC ACCCGGATAC TACGATGGCC GTTACTGGAC AATGTGGAAG CTTCCCTTGT TCGGATGCAC TGACTCTGCT CAGGTGTTGA AGGAAGTCCA AGAGTGCAAA ACAGAGTACC CTAACGCCTT CATCAGAATC
BniC2_B01g032900.1      TACCGTGAGC ACGGAAGCAC ACCCGGATAC TACGATGGCC GTTACTGGAC AATGTGGAAG CTTCCCTTGT TCGGATGCAC TGACTCTGCT CAGGTGTTGA AGGAAGTCCA AGAGTGCAAA ACAGAGTACC CTAACGCCTT CATCAGAATC
Bjut_O4471_new1         TACCGTGAGC ACGGAAGCAC ACCCGGATAC TACGATGGCC GTTACTGGAC AATGTGGAAG CTTCCCTTGT TCGGATGCAC TGACTCTGCT CAGGTGTTGA AGGAAGTCCA AGAGTGCAAA ACAGAGTACC CTAACGCCTT CATCAGAATC
Bjuv_B04_VARUNA_g244    TACCGTGAGC ACGGAAGCAC ACCCGGATAC TACGATGGCC GTTACTGGAC AATGTGGAAG CTTCCCTTGT TCGGATGCAC TGACTCTGCT CAGGTGTTGA AGGAAGTCCA AGAGTGCAAA ACAGAGTACC CTAACGCCTT CATCAGAATC
BniC2_B01g032690.1      TACCGTGAGC ACGGAAGCAC ACCCGGATAC TACGATGGCC GTTACTGGAC AATGTGGAAG CTTCCCCTGT TCGGATGCAC TGACTCTGCT CAAGTGTTGA AGGAAGTACA AGAGTGCAAA ACTGAGTACC CTAATGCCTT TATCAGAATC
BniN100_B01g029210.2    TACCGTGAGC ACGGAAGCAC ACCCGGATAC TACGATGGCC GTTACTGGAC AATGTGGAAG CTTCCCCTGT TCGGATGCAC TGACTCTGCT CAAGTGTTGA AGGAAGTACA AGAGTGCAAA ACTGAGTACC CTAATGCCTT TATCAGAATC
BniC2_B01g032670.1      TACCGTGAGC ACGGAAGCAC ACCCGGATAC TACGATGGCC GTTACTGGAC AATGTGGAAG CTTCCCCTGT TCGGATGCAC TGACTCTGCT CAAGTGTTGA AGGAAGTACA AGAGTGCAAA GCTGAGTACC CTAATGCCTT TATCAGAATC
Bjuv_B04_VARUNA_g243    TACCGTGAGC ACGGAAGCAC ACCCGGATAC TACGATGGCC GTTACTGGAC AATGTGGAAG CTTCCCCTGT TCGGATGCAC TGACTCTGCT CAAGTGTTGA AGGAAGTACA AGAGTGCAAA ACTGAGTACC CTAATGCCTT TATCAGAATC
Bjut_O012439            TACCGTGAGC ACGGAAGCAC ACCCGGATAC TACGATGGCC GTTACTGGAC AATGTGGAAG CTTCCCCTGT TCGGATGCAC TGACTCTGCT CAAGTGTTGA AGGAAGTACA AGAGTGCAAA ACTGAGTACC CTAATGCCTT TATCAGAATC

                        755        765        775        785        795        805        815        825
BraTUE_A04p11990.1      ATCGGATTCG ACAACAACCG TCAAGTCCAG TGCATCAGTT TCATCGCCTA CAAGCCACCA AGCTTCACCG GTGCTTAA
BraZ1_A04t16802         ATCGGATTCG ACAACAACCG TCAAGTCCAG TGCATCAGTT TCATCGCCTA CAAGCCACCA AGCTTCACCG GTGCTTAA
BraCCB_A04p11040.1      ATCGGATTCG ACAACAACCG TCAAGTCCAG TGCATCAGTT TCATCGCCTA CAAGCCACCA AGCTTCACCG GTGCTTAA
BraPCA_A04p10870.1      ATCGGATTCG ACAACAACCG TCAAGTCCAG TGCATCAGTT TCATCGCCTA CAAGCCACCA AGCTTCACCG GTGCTTAA
Bjuv_A04_VARUNA_g114    ATCGGATTCG ACAACAACCG TCAAGTCCAG TGCATCAGTT TCATCGCCTA CAAGCCACCA AGCTTCACCG GTGCTTAA
Bjut_A04_new1           ATCGGATTCG ACAACAACCG TCAAGTCCAG TGCATCAGTT TCATCGCCTA CAAGCCACCA AGCTTCACCG GTGCTTAA
BraPCA_A04p10800.1      ATCGGATTCG ACAACAACCG TCAAGTCCAG TGCATCAGTT TCATCGCCTA CAAACCACCA AGCTTCACCG GTGCTTAA
BraCCB_A04p10960.1      ATCGGATTCG ACAACAACCG TCAAGTCCAG TGCATCAGTT TCATCGCCTA CAAACCACCA AGCTTCACCG GTGCTTAA
BraZ1_A04t16795         ATCGGATTCG ACAACAACCG TCAAGTCCAG TGCATCAGTT TCATCGCCTA CAAACCACCA AGCTTCACCG GTGCTTAA
BraTUE_A04p11940.1      ATCGGATTCG ACAACAACCG TCAAGTCCAG TGCATCAGTT TCATCGCCTA CAAACCACCA AGCTTCACCG GTGCTTAA
Bjut_O011420            ATCGGATTCG ACAACAACCG TCAAGTCCAG TGCATCAGTT TCATCGCCTA CAAACCACCA AGCTTCACCG GTGCTTAA
Bjuv_A04_VARUNA_g114    ATCGGATTCG ACAACAACCG TCAAGTCCAG TGCATCAGTT TCATCGCCTA CAAACCACCA AGCTTCACCG GTGCTTAA
BniN100_B01g029450.2    ATCGGATTCG ACAACAACCG TCAAGTCCAG TGCATCAGTT TCATCGCGTA CAAACCACCA AGCTTCACCG GTGCTTAA
BniC2_B01g032930.1      ATCGGATTCG ACAACAACCG TCAAGTCCAG TGCATCAGTT TCATCGCGTA CAAACCACCA AGCTTCACCG GTGCTTAA
Bjuv_B04_VARUNA_g244    ATCGGATTCG ACAACAACCG TCAAGTCCAG TGCATCAGTT TCATCGCGTA CAAACCACCA AGCTTCACCG GTGCTTAA
Bjut_O001832            ATCGGATTCG ACAACAACCG TCAAGTCCAG TGCATCAGTT TCATCGCGTA CAAACCACCA AGCTTCACCG GTGCTTAA
BraPCA_A04p10780.1      ATCGGATTCG ACAACAACCG TCAAGTCCAG TGCATCAGTT TCATCGCCTA CAAGCCACCA AGCTTCACCG GTGCTTAA
BraCCB_A04p10950.1      ATCGGATTCG ACAACAACCG TCAAGTCCAG TGCATCAGTT TCATCGCCTA CAAGCCACCA AGCTTCACCG GTGCTTAA
BraZ1_A04t16794         ATCGGATTCG ACAACAACCG TCAAGTCCAG TGCATCAGTT TCATCGCCTA CAAGCCACCA AGCTTCACCG GTGCTTAA
BraTUE_A04p11960.1      ATCGGATTCG ACAACAACCG TCAAGTCCAA TGCATCAGTT TCATCGCCTA CAAGCCACCA AGCTTCACCG GTGCTTAA
Bjut_O011421            ATCGGATTCG ACAACAACCG TCAAGTCCAG TGCATCAGTT TCATCGCGTA CAAACCACCA AGCTTCACCG GTGCTTAA
Bjuv_A04_VARUNA_g114    ATCGGATTCG ACAACAACCG TCAAGTCCAG TGCATCAGTT TCATCGCCTA CAAGCCACCA AGCTTCACCG GTGCTTAA
BniN100_B01g029420.2    ATCGGATTCG ACAACAACCG TCAAGTCCAG TGCATCAGTT TCATCGCGTA CAAACCACCA AGCTTCACCG GTGCTTAA
BniC2_B01g032900.1      ATCGGATTCG ACAACAACCG TCAAGTCCAG TGCATCAGTT TCATCGCGTA CAAACCACCA AGCTTCACCG GTGCTTAA
Bjut_O4471_new1         ATCGGATTCG ACAACAACCG TCAAGTCCAG TGCATCAGTT TCATCGCGTA CAAACCACCA AGCTTCACCG GTGCTTAA
Bjuv_B04_VARUNA_g244    ATCGGATTCG ACAACAACCG TCAAGTCCAG TGCATCAGTT TCATCGCGTA CAAACCACCA AGCTTCACCG GTGCTTAA
BniC2_B01g032690.1      ATCGGATTCG ACAACAACCG TCAAGTCCAG TGCATCAGTT TCATCGCCTA CAAGCCACCA AGCTTCACCG GTGCTTAA
BniN100_B01g029210.2    ATCGGATTCG ACAACAACCG TCAAGTCCAG TGCATCAGTT TCATCGCCTA CAAGCCACCA AGCTTCACCG GTGCTTAA
BniC2_B01g032670.1      ATCGGATTCG ACAACAACCG TCAAGTCCAG TGCATCAGTT TCATCGCCTA CAAGCCACCA AGCTTCACCG GTGCTTAA
Bjuv_B04_VARUNA_g243    ATCGGATTCG ACAACAACCG TCAAGTCCAG TGCATCAGTT TCATCGCCTA CAAGCCACCA AGCTTCACCG GTGCTTAA
Bjut_O012439            ATCGGATTCG ACAACAACCG TCAAGTCCAG TGCATCAGTT TCATCGCCTA CAAGCCACCA AGCTTCACCG GTGCTTAA
```

(F) Clade IB BBCC

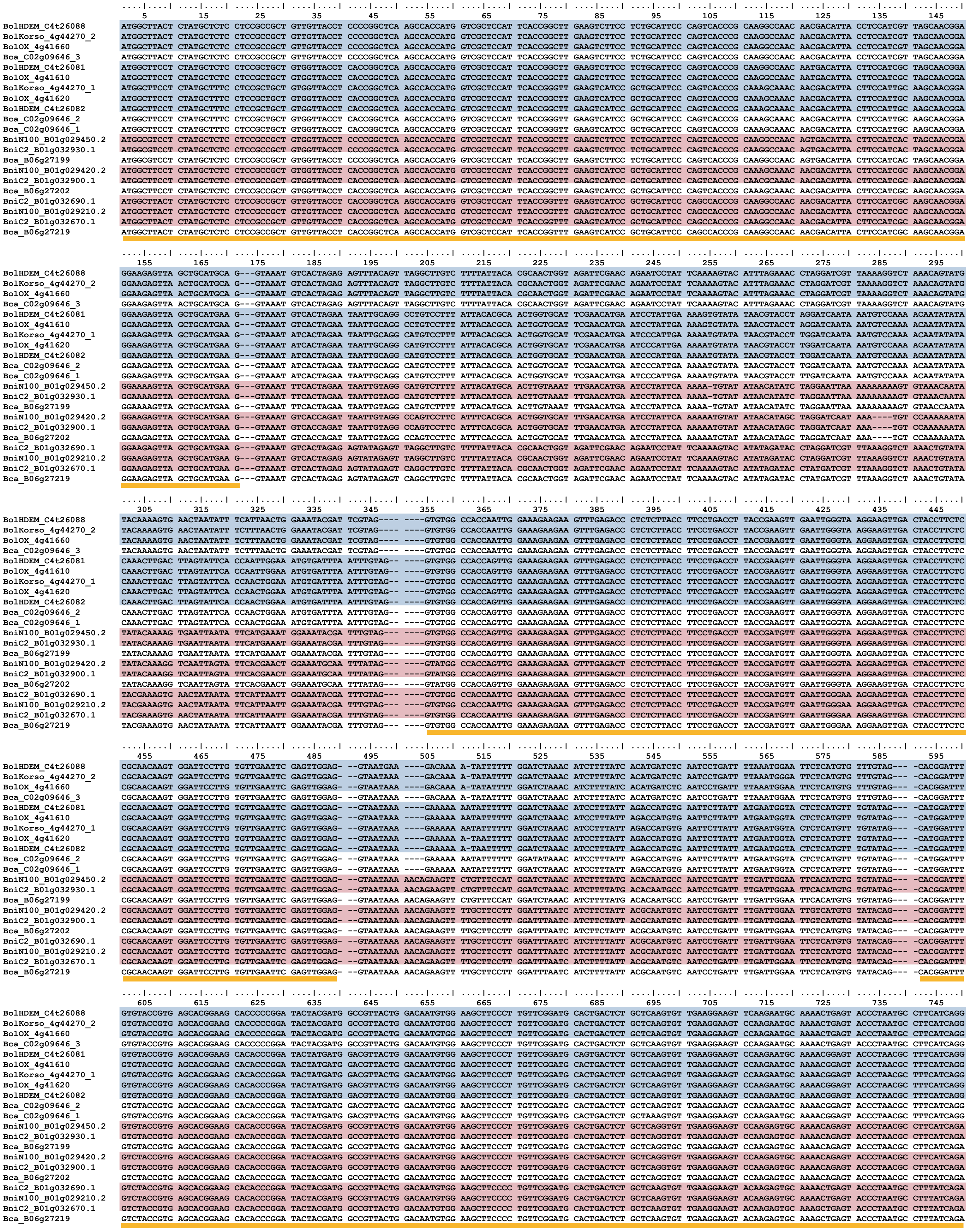

```
                         ....|....| ....|....| ....|....| ....|....| ....|....| ....|....| ....|....| ....|....| .
                              755        765        775        785        795        805        815        825
BolHDEM_C4t26088         ATCATTGGAT TCGACAACAA CCGTCAAGTC CAGTGCATCA GTTTCATCGC TTACAAGCCA CCAAGCTTCA CCGGTGCTTA A
BolKorso_4g44270_2       ATCATCGGAT TCGACAACAA CCGTCAAGTC CAGTGCATCA GTTTCATCGC GTACAAGCCA CCAAGCTTCA CCGGTGCTTA A
BolOX_4g41660            ATCATCGGAT TCGACAACAA CCGTCAAGTC CAGTGCATCA GTTTCATCGC GTACAAGCCA CCAAGCTTCA CCGGTGCTTA A
Bca_C02g09646_3          ATCATCGGAT TCGACAACAA CCGTCAAGTC CAGTGCATCA GTTTCATCGC GTACAAGCCA CCAAGCTTCA CCGGTGCTTA A
BolHDEM_C4t26081         ATCATCGGAT TTGACAACAA CCGTCAGGTT CAGTGCATCA GTTTCATCGC CTACAAACCA CCAAGCTTCA CCGGTGCTTA A
BolOX_4g41610            ATCATCGGAT TTGACAACAA CCGTCAGGTT CAGTGCATCA GTTTCATCGC CTACAAACCA CCAAGCTTCA CCGGTGCTTA A
BolKorso_4g44270_1       ATCATCGGAT TTGACAACAA CCGTCAGGTT CAGTGCATCA GTTTCATCGC CTACAAACCA CCAAGCTTCA CCGGTGCTTA A
BolOX_4g41620            ATCATCGGAT TTGACAACAA CCGTCAGGTT CAGTGCATCA GTTTCATCGC CTACAAACCA CCAAGCTTCA CCGGTGCTTA A
BolHDEM_C4t26082         ATCATCGGAT TTGACAACAA CCGTCAGGTT CAGTGCATCA GTTTCATCGC CTACAAACCA CCAAGCTTCA CCGGTGCTTA A
Bca_C02g09646_2          ATCATCGGAT TTGACAACAA CCGTCAGGTT CAGTGCATCA GTTTCATCGC CTACAAACCA CCAAGCTTCA CCGGTGCTTA A
Bca_C02g09646_1          ATCATCGGAT TTGACAACAA CCGTCAGGTT CAGTGCATCA GTTTCATCGC CTACAAACCA CCAAGCTTCA CCGGTGCTTA A
BniN100_B01g029450.2     ATCATCGGAT TCGACAACAA CCGTCAAGTC CAGTGCATCA GTTTCATCGC GTACAAACCA CCAAGCTTCA CCGGTGCTTA A
BniC2_B01g032930.1       ATCATCGGAT TCGACAACAA CCGTCAAGTC CAGTGCATCA GTTTCATCGC GTACAAACCA CCAAGCTTCA CCGGTGCTTA A
Bca_B06g27199            ATCATCGGAT TCGACAACAA CCGTCAAGTC CAGTGCATCA GTTTCATCGC GTACAAACCA CCAAGCTTCA CCGGTGCTTA A
BniN100_B01g029420.2     ATCATCGGAT TCGACAACAA CCGTCAAGTC CAGTGCATCA GTTTCATCGC GTACAAACCA CCAAGCTTCA CCGGTGCTTA A
BniC2_B01g032900.1       ATCATCGGAT TCGACAACAA CCGTCAAGTC CAGTGCATCA GTTTCATCGC GTACAAACCA CCAAGCTTCA CCGGTGCTTA A
Bca_B06g27202            ATCATCGGAT TCGACAACAA CCGTCAAGTC CAGTGCATCA GTTTCATCGC GTACAAACCA CCAAGCTTCA CCGGTGCTTA A
BniC2_B01g032690.1       ATCATCGGAT TCGACAACAA CCGTCAAGTC CAGTGCATCA GTTTCATCGC CTACAAGCCA CCAAGCTTCA CCGGTGCTTA A
BniN100_B01g029210.2     ATCATCGGAT TCGACAACAA CCGTCAAGTC CAGTGCATCA GTTTCATCGC CTACAAGCCA CCAAGCTTCA CCGGTGCTTA A
BniC2_B01g032670.1       ATCATCGGAT TCGACAACAA CCGTCAAGTC CAGTGCATCA GTTTCATCGC CTACAAGCCA CCAAGCTTCA CCGGTGCTTA A
Bca_B06g27219            ATCATCGGAT TCGACAACAA CCGTCAAGTC CAGTGCATCA GTTTCATCGC CTACAAGCCA CCAAGCTTCA CCGGTGCTTA A
```

(G) Clade II AABB

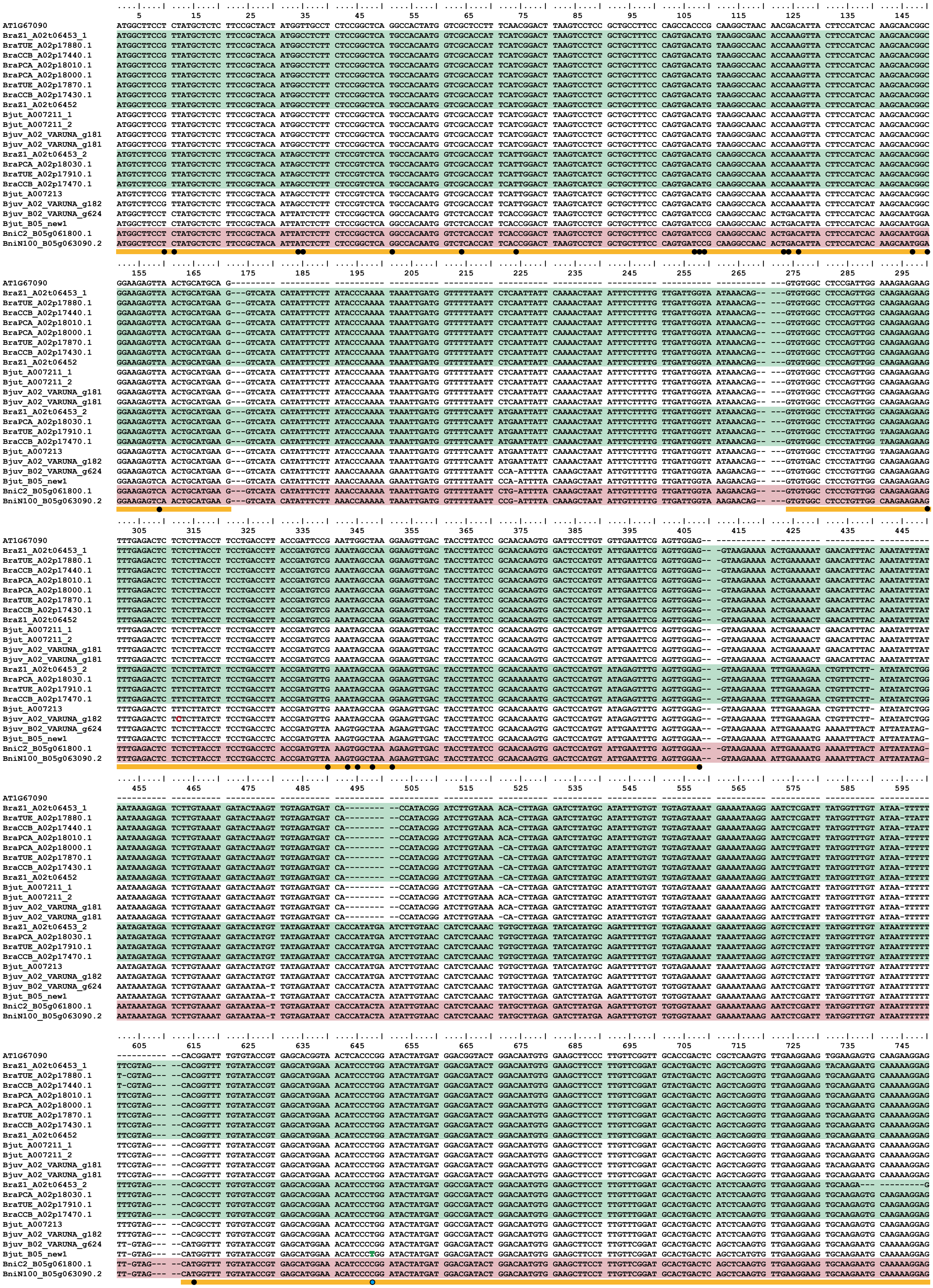

```
                        ....|....| ....|....| ....|....| ....|....| ....|....| ....|....| ....|....| ....|....| ....|....| ....|....| ..
                              755        765        775        785        795        805        815        825        835        845
AT1G67090               TACCCCAATG CCTTCATTAG GATCATCGGA TTCGACAACA CCCGTCAAGT CCAGTGCATC AGTTTCATTG CCTACAAGCC ACCAAGCTTC ACCGGTTAA- --
BraZ1_A02t06453_1       TACCCCAACG CCTTCATTAG GATCATCGGA TTCGACAACA ATCGTCAAGC CCAGTGCATC AGTTTCATCG CCTACAAGCC ACCAAGCTTC ACTAATGCTT AA
BraTUE_A02p17880.1      TACCCCAACG CCTTCATTAG GATCATCGGA TTCGACAACA ATCGTCAAGC CCAGTGCATC AGTTTCATCG CCTACAAGCC ACCAAGCTTC ACTAATGCTT AA
BraCCB_A02p17440.1      TACCCCAACG CCTTCATTAG GATCATCGGA TTCGACAACA ATCGTCAAGC CCAGTGCATC AGTTTCATCG CCTACAAGCC ACCAAGCTTC ACTAATGCTT AA
BraPCA_A02p18010.1      TACCCCAACG CCTTCATTAG GATCATCGGA TTCGACAACA ATCGTCAAGC CCAGTGCATC AGTTTCATCG CCTACAAGCC ACCAAGCTTC ACTAATGCTT AA
BraPCA_A02p18000.1      TACCCCAACG CCTTCATTAG GATCATCGGA TTCGACAACA ATCGTCAAGC CCAGTGCATC AGTTTCATCG CCTACAAGCC ACCAAGCTTC ACTGATGCTT AA
BraTUE_A02p17870.1      TACCCCAACG CCTTCATTAG GATCATCGGA TTCGACAACA ATCGTCAAGC CCAGTGCATC AGTTTCATCG CCTACAAGCC ACCAAGCTTC ACTGATGCTT AA
BraCCB_A02p17430.1      TACCCCAACG CCTTCATTAG GATCATCGGA TTCGACAACA ATCGTCAAGC CCAGTGCATC AGTTTCATCG CCTACAAGCC ACCAAGCTTC ACTGATGCTT AA
BraZ1_A02t06452         TACCCCAACG CCTTCATTAG GATCATCGGA TTCGACAACA ATCGTCAAGC CCAGTGCATC AGTTTCATCG CCTACAAGCC ACCAAGCTTC ACTGATGCTT AA
Bjut_A007211_1          TACCCCAACG CCTTCATTAG GATCATCGGA TTCGACAACA ATCGTCAAGC CCAGTGCATC AGTTTCATCG CCTACAAGCC ACCAAGCTTC ACTAATGCTT AA
Bjut_A007211_2          TACCCCAACG CCTTCATTAG GATCATCGGA TTCGACAACA ATCGTCAAGC CCAGTGCATC AGTTTCATCG CCTACAAGCC ACCAAGCTTC ACTAATGCTT AA
Bjuv_A02_VARUNA_g181    TACCCCAACG CCTTCATTAG GATCATCGGA TTCGACAACA ATCGTCAAGC CCAGTGCATC AGTTTCATCG CCTACAAGCC ACCAAGCTTC ACTAATGCTT AA
Bjuv_A02_VARUNA_g181    TACCCCAACG CCTTCATTAG GATCATCGGA TTCGACAACA ATCGTCAAGC CCAGTGCATC AGTTTCATCG CCTACAAGCC ACCAAGCTTC ACTGATGCTT AA
BraZ1_A02t06453_2       TACCCCAACG CCTTCATTAG GATCATCGGA TTCGACAACA ACCGTCAAGC CCAGTGCATC AGTTTCATCG CCTATAAGCC ACTAAGCTTC GGCGATGCTT AA
BraPCA_A02p18030.1      TACCCCAACG CCTTCATTAG GATCATCGGA TTCGACAACA ACCGTCAAGC CCAGTGCATC AGTTTCATCG CCTATAAGCC ACTAAGCTTC GGCGATGCTT AA
BraTUE_A02p17910.1      TACCCCAACG CCTTCATTAG GATCATCGGA TTCGACAACA ACCGTCAAGC CCAGTGCATC AGTTTCATCG CCTATAAGCC ACTAAGCTTC GGCGATGCTT AA
BraCCB_A02p17470.1      TACCCCAACG CCTTCATTAG GATCATCGGA TTCGACAACA ACCGTCAAGC CCAGTGCATC AGTTTCATCG CCTATAAGCC ACTAAGCTTC GGCGATGCTT AA
Bjut_A007213            TACCCCAACG CCTTCATTAG GATCATCGGA TTCGACAACA ACCGTCAAGC CCAGTGCATC AGTTTCATCG CCTATAAGCC ACTAAGCTTC GGCGATGCTT AA
Bjuv_A02_VARUNA_g182    TACCCCAACG CCTTCATTAG GATCATCGGA TTCGACAACA ACCGTCAAGC CCAGTGCATC AGTTTCATCG CCTATAAGCC ACTAAGCTTC GGCGATGCTT AA
Bjuv_B02_VARUNA_g624    TACCCCAACG CCTTCATTAG GATCATTGGA TTCGACAACA ACCGTCAAGC GCAGTGCATC AGTTTCATCG CCTACAAGCC ACCAAGCATC ACTGGTGCTT AA
Bjut_B05_new1           TACCCCAACG CCTTCATTAG GATCATTGGA TTCGACAACA ACCGTCAAGC GCAGTGCATC AGTTTCATCG CCTACAAGCC ACCAAGCATC ACTGGTGCTT AA
BniC2_B05g061800.1      TACCCCAACG CCTTCATTAG GATCATTGGA TTCGACAACA ACCGTCAAGC GCAGTGCATC AGTTTCATCG CCTACAAGCC ACCAAGCATC ACTGGTGCTT AA
BniN100_B05g063090.2    TACCCCAACG CCTTCATTAG GATCATTGGA TTCGACAACA ACCGTCAAGC GCAGTGCATC AGTTTCATCG CCTACAAGCC ACCAAGCATC ACTGGTGCTT AA
```

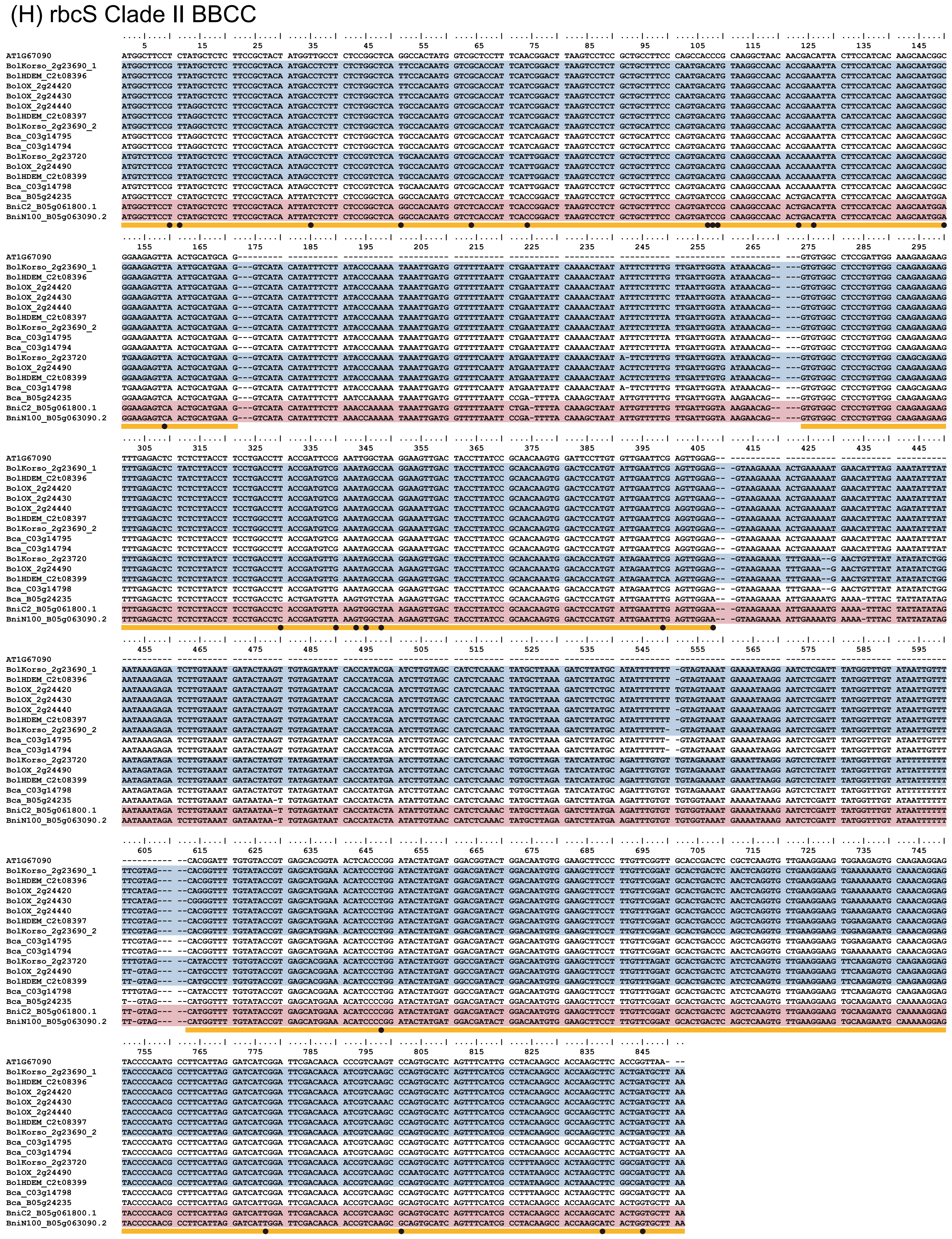

Supplementary Fig S6. Alignment of *rbcS* genes in studied genomes/subgenomes. Yellow lines represent the exons. The shade in green indicates *B. rapa* (AA), red indicates *B. nigra* (BB), and blue indicates *B. oleracea* (CC). The black dot indicates genome-specific site, blue dot indicates synonymous inter-genomic conversion, red dot indicates non-synonymous inter-genomic conversion, and grey dot indicates autapomorphy.
