# Supplementary material for: Cytonuclear Interactions and Subgenome Dominance Shape the Evolution of Organelle-Targeted Genes in the Brassica Triangle of U
Source: Mol Biol Evol. 2024 Feb 23;41(3):msae043. doi: 10.1093/molbev/msae043 (PMC10919925; doi:10.1093/molbev/msae043)
Supplement: msae043_Supplementary_Data [file msae043_supplementary_data.zip › Supplementary Figure S9.pdf]

(A) MPP-α

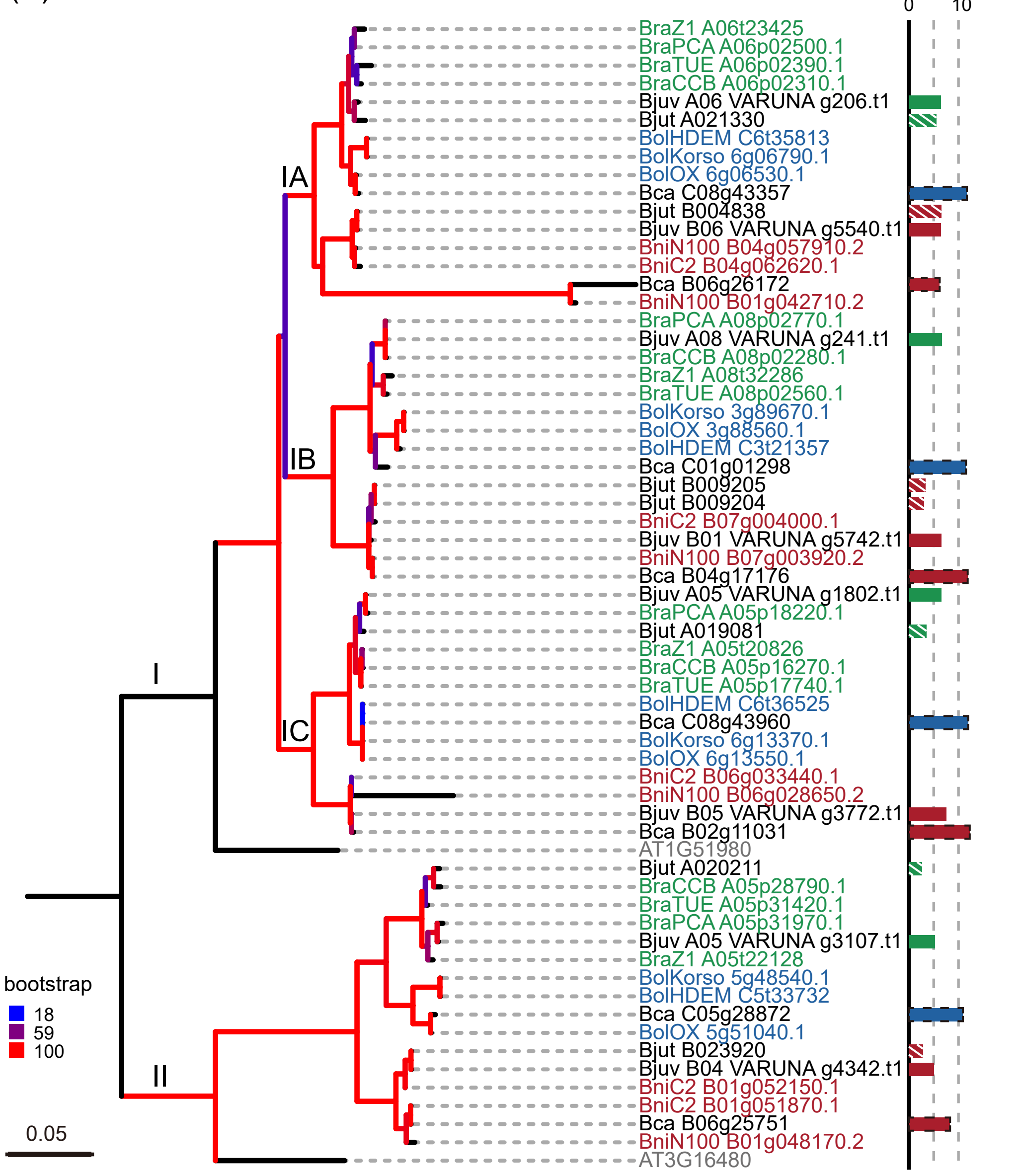
log₂(FPKM)
0
10
IA
IB
IC
I
II
bootstrap
18
59
100
0.05
BraZ1 A06t23425
BraPCA A06p02500.1
BraTUE A06p02390.1
BraCCB A06p02310.1
Bjuv A06 VARUNA g206.t1
Bjut A021330
BolHDEM C6t35813
BolKorso 6g06790.1
BolOX 6g06530.1
Bca C08g43357
Bjut B004838
Bjuv B06 VARUNA g5540.t1
BniN100 B04g057910.2
BniC2 B04g062620.1
Bca B06g26172
BniN100 B01g042710.2
BraPCA A08p02770.1
Bjuv A08 VARUNA g241.t1
BraCCB A08p02280.1
BraZ1 A08t32286
BraTUE A08p02560.1
BolKorso 3g89670.1
BolOX 3g88560.1
BolHDEM C3t21357
Bca C01g01298
Bjut B009205
Bjut B009204
BniC2 B07g004000.1
Bjuv B01 VARUNA g5742.t1
BniN100 B07g003920.2
Bca B04g17176
Bjuv A05 VARUNA g1802.t1
BraPCA A05p18220.1
Bjut A019081
BraZ1 A05t20826
BraCCB A05p16270.1
BraTUE A05p17740.1
BolHDEM C6t36525
Bca C08g43960
BolKorso 6g13370.1
BolOX 6g13550.1
BniC2 B06g033440.1
BniN100 B06g028650.2
Bjuv B05 VARUNA g3772.t1
Bca B02g11031
AT1G51980
Bjut A020211
BraCCB A05p28790.1
BraTUE A05p31420.1
BraPCA A05p31970.1
Bjuv A05 VARUNA g3107.t1
BraZ1 A05t22128
BolKorso 5g48540.1
BolHDEM C5t33732
Bca C05g28872
BolOX 5g51040.1
Bjut B023920
Bjuv B04 VARUNA g4342.t1
BniC2 B01g052150.1
BniC2 B01g051870.1
Bca B06g25751
BniN100 B01g048170.2
AT3G16480

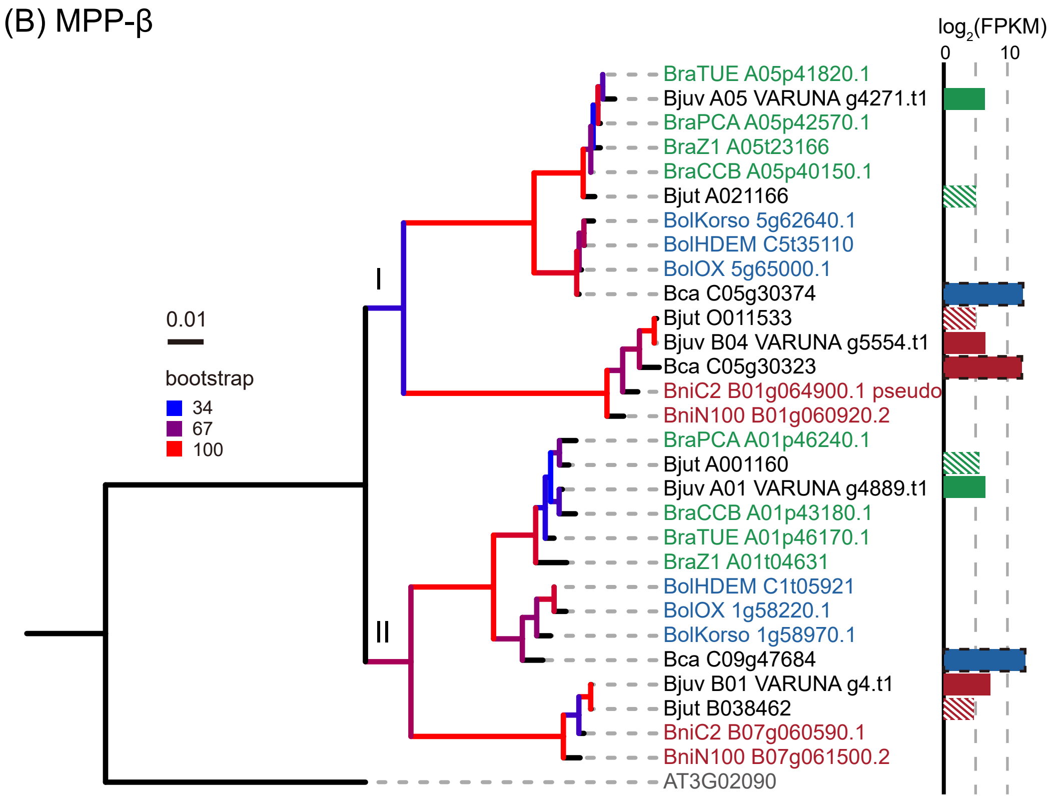

(B) MPP-β
log2(FPKM)
0
10
0.01
bootstrap
34
67
100
I
II
BraTUE A05p41820.1
Bjuv A05 VARUNA g4271.t1
BraPCA A05p42570.1
BraZ1 A05t23166
BraCCB A05p40150.1
Bjut A021166
BolKorso 5g62640.1
BolHDEM C5t35110
BolOX 5g65000.1
Bca C05g30374
Bjut O011533
Bjuv B04 VARUNA g5554.t1
Bca C05g30323
BniC2 B01g064900.1 pseudo
BniN100 B01g060920.2
BraPCA A01p46240.1
Bjut A001160
Bjuv A01 VARUNA g4889.t1
BraCCB A01p43180.1
BraTUE A01p46170.1
BraZ1 A01t04631
BolHDEM C1t05921
BolOX 1g58220.1
BolKorso 1g58970.1
Bca C09g47684
Bjuv B01 VARUNA g4.t1
Bjut B038462
BniC2 B07g060590.1
BniN100 B07g061500.2
AT3G02090

(C) QCR7

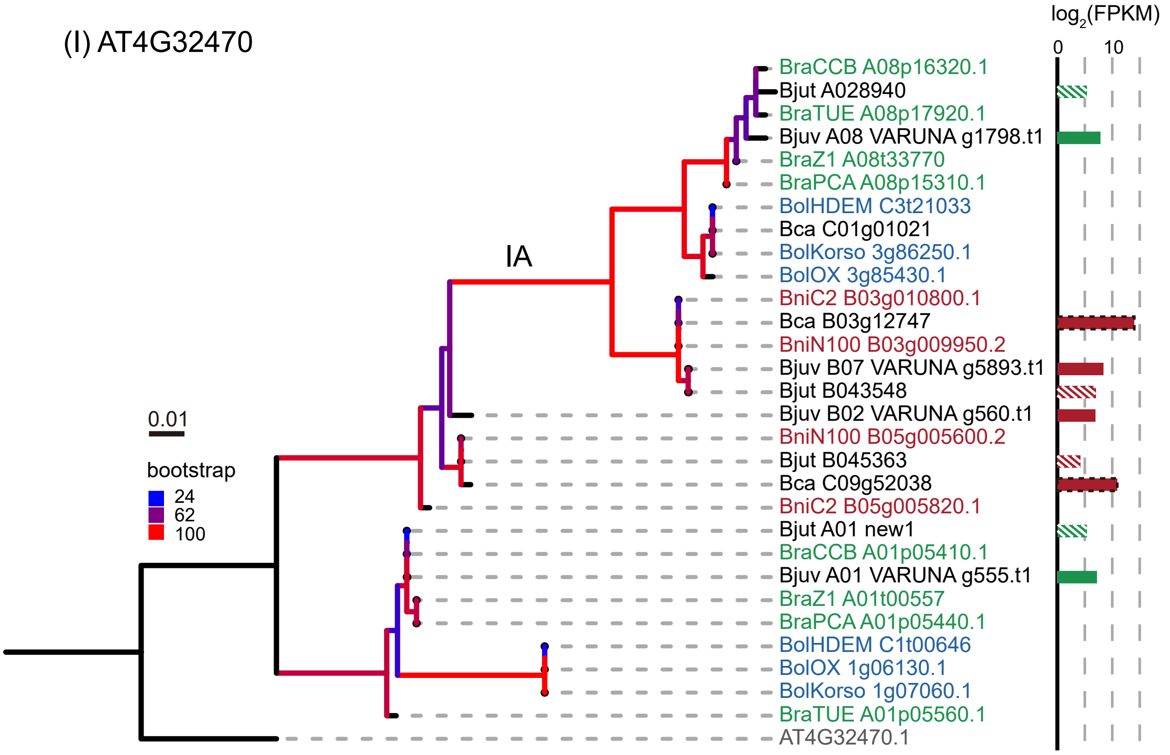

(I) AT4G32470
log2(FPKM)
0
10
IA
0.01
bootstrap
24
62
100
BraCCB A08p16320.1
Bjut A028940
BraTUE A08p17920.1
Bjuv A08 VARUNA g1798.t1
BraZ1 A08t33770
BraPCA A08p15310.1
BolHDEM C3t21033
Bca C01g01021
BolKorso 3g86250.1
BolOX 3g85430.1
BniC2 B03g010800.1
Bca B03g12747
BniN100 B03g009950.2
Bjuv B07 VARUNA g5893.t1
Bjut B043548
Bjuv B02 VARUNA g560.t1
BniN100 B05g005600.2
Bjut B045363
Bca C09g52038
BniC2 B05g005820.1
Bjut A01 new1
BraCCB A01p05410.1
Bjuv A01 VARUNA g555.t1
BraZ1 A01t00557
BraPCA A01p05440.1
BolHDEM C1t00646
BolOX 1g06130.1
BolKorso 1g07060.1
BraTUE A01p05560.1
AT4G32470.1

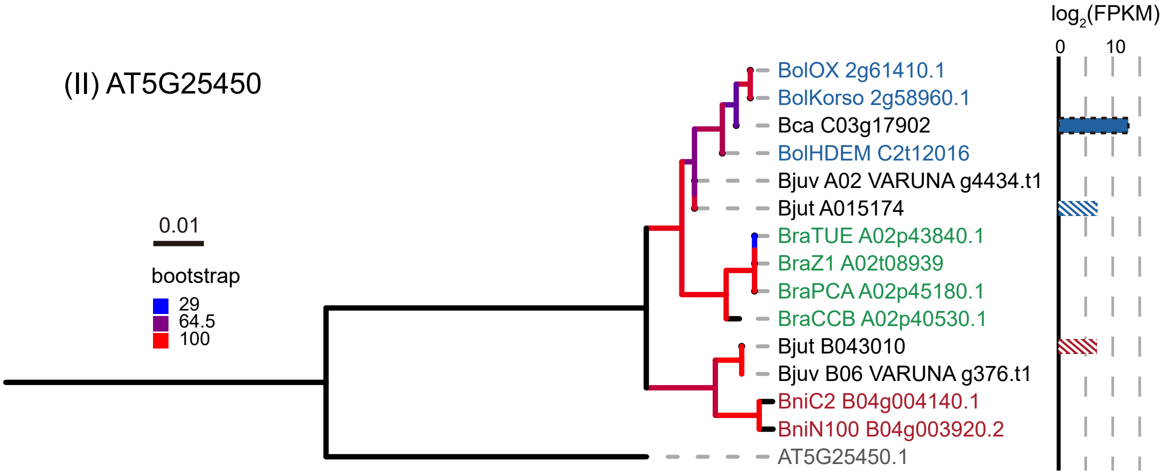

(II) AT5G25450
log2(FPKM)
0
10
0.01
bootstrap
29
64.5
100
BolOX 2g61410.1
BolKorso 2g58960.1
Bca C03g17902
BolHDEM C2t12016
Bjuv A02 VARUNA g4434.t1
Bjut A015174
BraTUE A02p43840.1
BraZ1 A02t08939
BraPCA A02p45180.1
BraCCB A02p40530.1
Bjut B043010
Bjuv B06 VARUNA g376.t1
BniC2 B04g004140.1
BniN100 B04g003920.2
AT5G25450.1

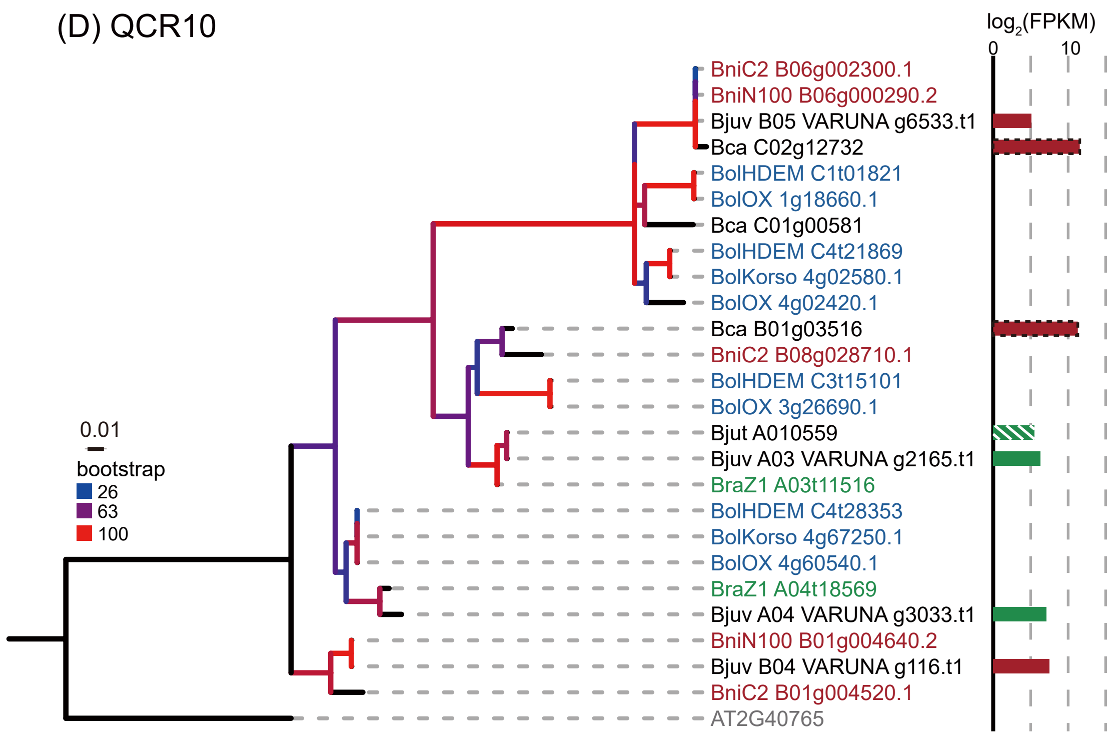
(D) QCR10
log2(FPKM)
0
10
0.01
bootstrap
26
63
100
BniC2 B06g002300.1
BniN100 B06g000290.2
Bjuv B05 VARUNA g6533.t1
Bca C02g12732
BolHDEM C1t01821
BolOX 1g18660.1
Bca C01g00581
BolHDEM C4t21869
BolKorso 4g02580.1
BolOX 4g02420.1
Bca B01g03516
BniC2 B08g028710.1
BolHDEM C3t15101
BolOX 3g26690.1
Bjut A010559
Bjuv A03 VARUNA g2165.t1
BraZ1 A03t11516
BolHDEM C4t28353
BolKorso 4g67250.1
BolOX 4g60540.1
BraZ1 A04t18569
Bjuv A04 VARUNA g3033.t1
BniN100 B01g004640.2
Bjuv B04 VARUNA g116.t1
BniC2 B01g004520.1
AT2G40765

(E) QCR9

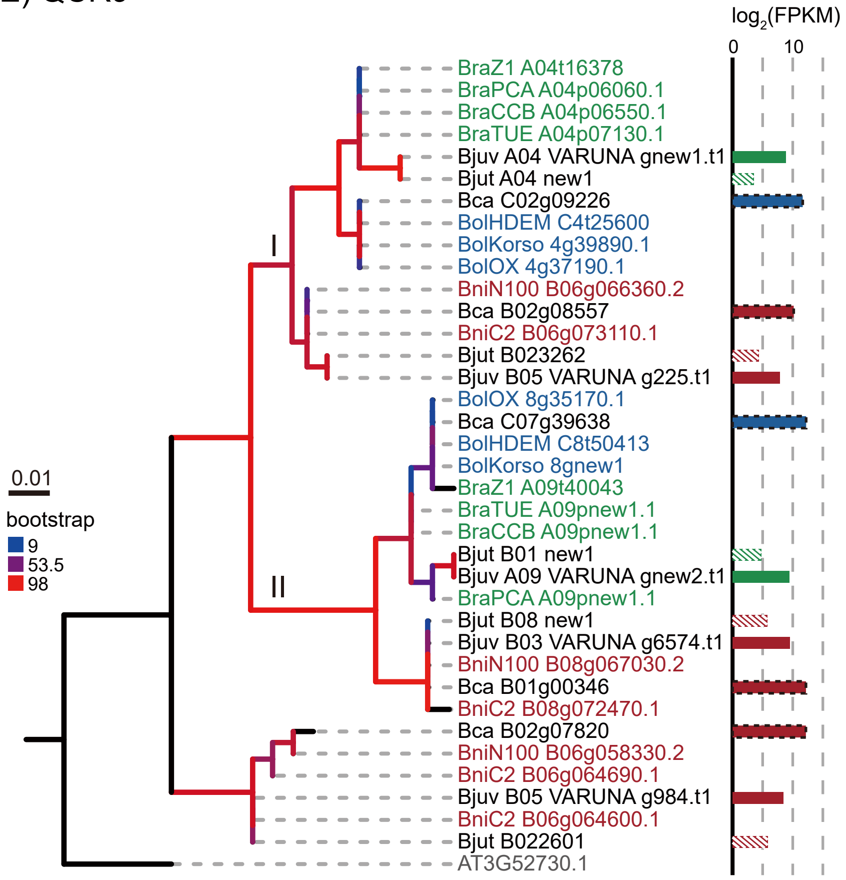
log2(FPKM)
0
10
BraZ1 A04t16378
BraPCA A04p06060.1
BraCCB A04p06550.1
BraTUE A04p07130.1
Bjuv A04 VARUNA gnew1.t1
Bjut A04 new1
Bca C02g09226
BolHDEM C4t25600
BolKorso 4g39890.1
BolOX 4g37190.1
BniN100 B06g066360.2
Bca B02g08557
BniC2 B06g073110.1
Bjut B023262
Bjuv B05 VARUNA g225.t1
BolOX 8g35170.1
Bca C07g39638
BolHDEM C8t50413
BolKorso 8gnew1
BraZ1 A09t40043
BraTUE A09pnew1.1
BraCCB A09pnew1.1
Bjut B01 new1
Bjuv A09 VARUNA gnew2.t1
BraPCA A09pnew1.1
Bjut B08 new1
Bjuv B03 VARUNA g6574.t1
BniN100 B08g067030.2
Bca B01g00346
BniC2 B08g072470.1
Bca B02g07820
BniN100 B06g058330.2
BniC2 B06g064690.1
Bjuv B05 VARUNA g984.t1
BniC2 B06g064600.1
Bjut B022601
AT3G52730.1
I
II
0.01
bootstrap
9
53.5
98

(F) QCR8

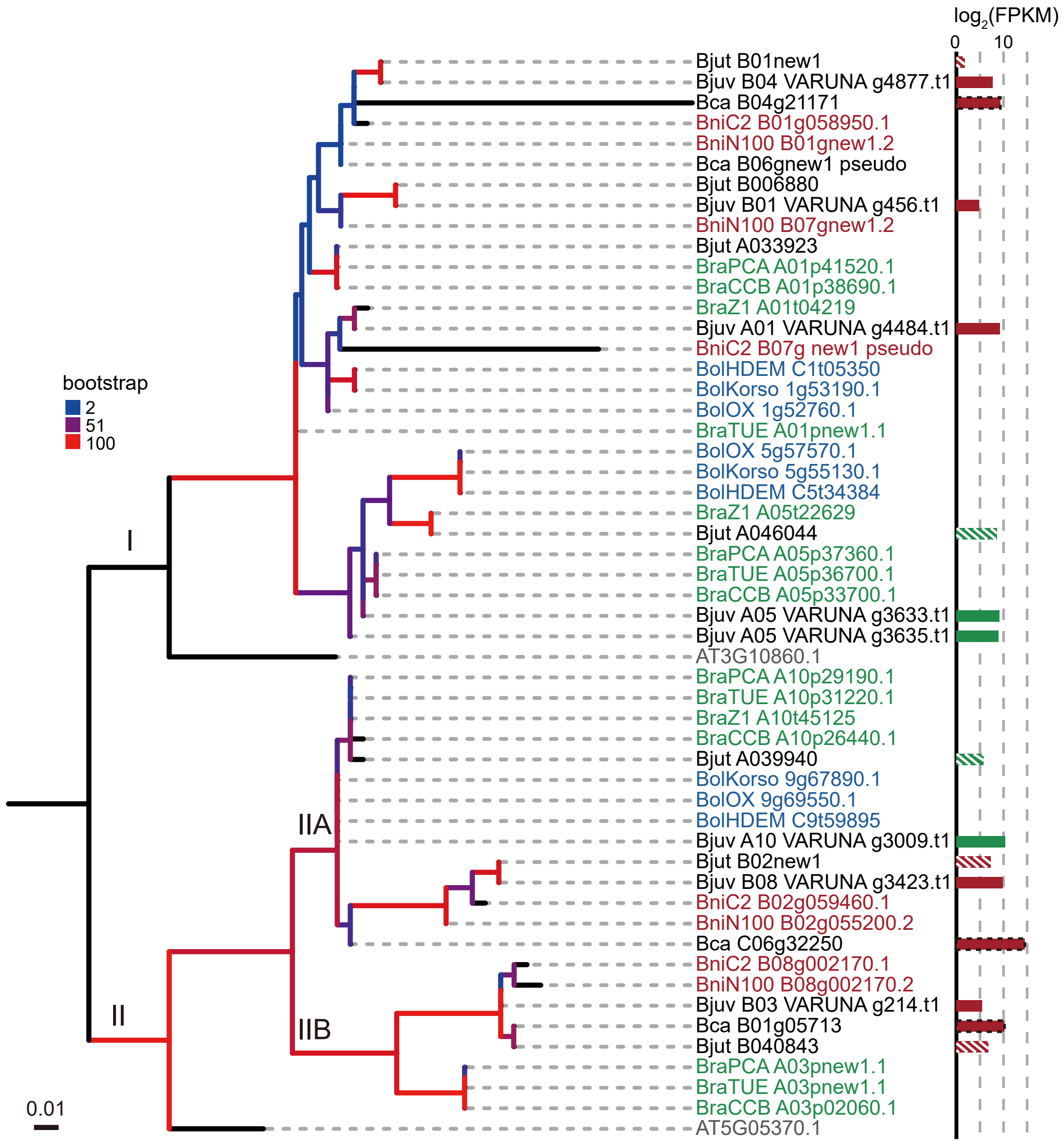

log2(FPKM)
0
10
bootstrap
2
51
100
I
II
IIA
IIB
0.01
Bjut B01new1
Bjuv B04 VARUNA g4877.t1
Bca B04g21171
BniC2 B01g058950.1
BniN100 B01gnew1.2
Bca B06gnew1 pseudo
Bjut B006880
Bjuv B01 VARUNA g456.t1
BniN100 B07gnew1.2
Bjut A033923
BraPCA A01p41520.1
BraCCB A01p38690.1
BraZ1 A01t04219
Bjuv A01 VARUNA g4484.t1
BniC2 B07g new1 pseudo
BolHDEM C1t05350
BolKorso 1g53190.1
BolOX 1g52760.1
BraTUE A01pnew1.1
BolOX 5g57570.1
BolKorso 5g55130.1
BolHDEM C5t34384
BraZ1 A05t22629
Bjut A046044
BraPCA A05p37360.1
BraTUE A05p36700.1
BraCCB A05p33700.1
Bjuv A05 VARUNA g3633.t1
Bjuv A05 VARUNA g3635.t1
AT3G10860.1
BraPCA A10p29190.1
BraTUE A10p31220.1
BraZ1 A10t45125
BraCCB A10p26440.1
Bjut A039940
BolKorso 9g67890.1
BolOX 9g69550.1
BolHDEM C9t59895
Bjuv A10 VARUNA g3009.t1
Bjut B02new1
Bjuv B08 VARUNA g3423.t1
BniC2 B02g059460.1
BniN100 B02g055200.2
Bca C06g32250
BniC2 B08g002170.1
BniN100 B08g002170.2
Bjuv B03 VARUNA g214.t1
Bca B01g05713
Bjut B040843
BraPCA A03pnew1.1
BraTUE A03pnew1.1
BraCCB A03p02060.1
AT5G05370.1

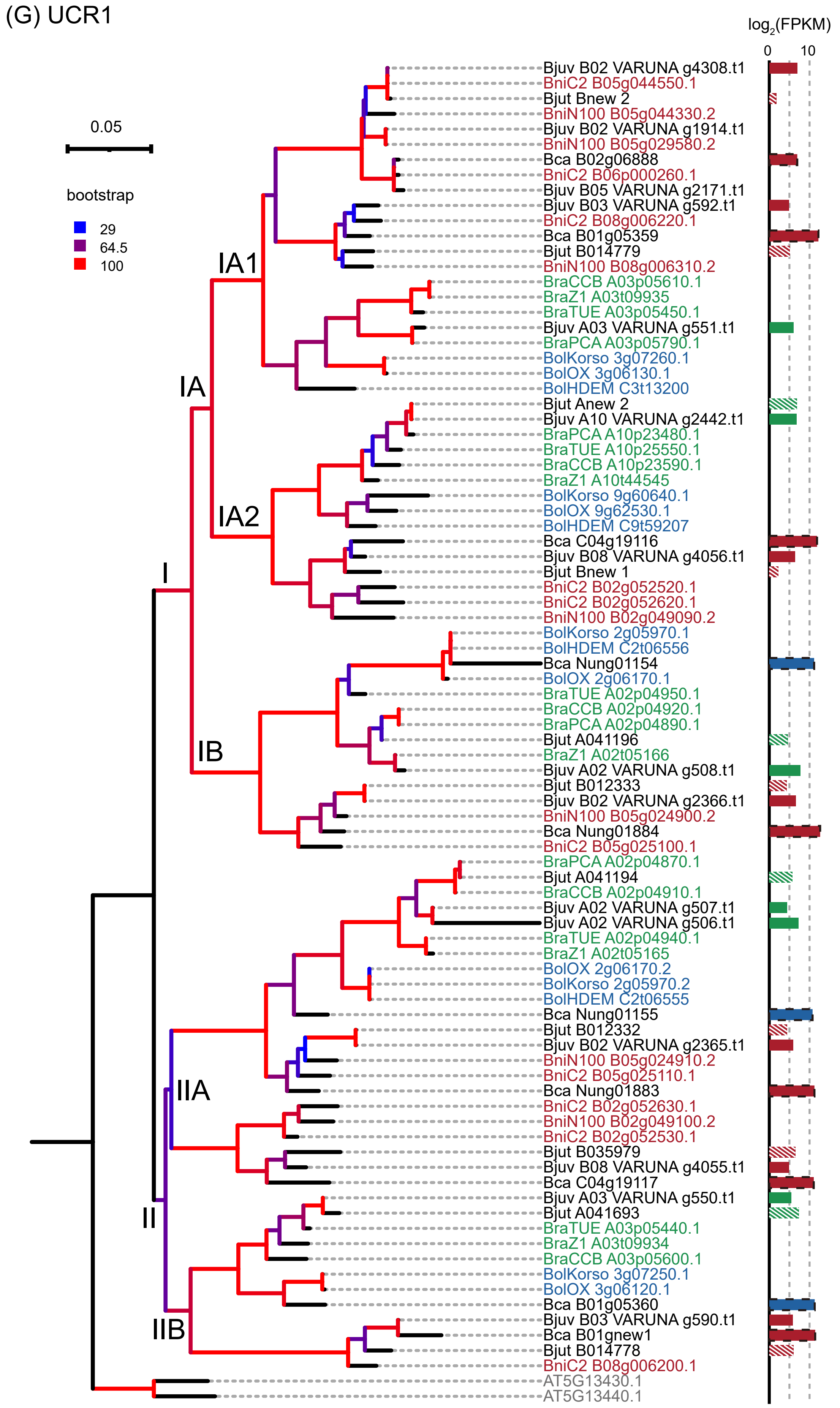
(G) UCR1
log2(FPKM)
0
10
0.05
bootstrap
29
64.5
100
IA1
IA
IA2
I
IB
IIA
II
IIB
Bjuv B02 VARUNA g4308.t1
BniC2 B05g044550.1
Bjut Bnew 2
BniN100 B05g044330.2
Bjuv B02 VARUNA g1914.t1
BniN100 B05g029580.2
Bca B02g06888
BniC2 B06p000260.1
Bjuv B05 VARUNA g2171.t1
Bjuv B03 VARUNA g592.t1
BniC2 B08g006220.1
Bca B01g05359
Bjut B014779
BniN100 B08g006310.2
BraCCB A03p05610.1
BraZ1 A03t09935
BraTUE A03p05450.1
Bjuv A03 VARUNA g551.t1
BraPCA A03p05790.1
BolKorso 3g07260.1
BolOX 3g06130.1
BolHDEM C3t13200
Bjut Anew 2
Bjuv A10 VARUNA g2442.t1
BraPCA A10p23480.1
BraTUE A10p25550.1
BraCCB A10p23590.1
BraZ1 A10t44545
BolKorso 9g60640.1
BolOX 9g62530.1
BolHDEM C9t59207
Bca C04g19116
Bjuv B08 VARUNA g4056.t1
Bjut Bnew 1
BniC2 B02g052520.1
BniC2 B02g052620.1
BniN100 B02g049090.2
BolKorso 2g05970.1
BolHDEM C2t06556
Bca Nung01154
BolOX 2g06170.1
BraTUE A02p04950.1
BraCCB A02p04920.1
BraPCA A02p04890.1
Bjut A041196
BraZ1 A02t05166
Bjuv A02 VARUNA g508.t1
Bjut B012333
Bjuv B02 VARUNA g2366.t1
BniN100 B05g024900.2
Bca Nung01884
BniC2 B05g025100.1
BraPCA A02p04870.1
Bjut A041194
BraCCB A02p04910.1
Bjuv A02 VARUNA g507.t1
Bjuv A02 VARUNA g506.t1
BraTUE A02p04940.1
BraZ1 A02t05165
BolOX 2g06170.2
BolKorso 2g05970.2
BolHDEM C2t06555
Bca Nung01155
Bjut B012332
Bjuv B02 VARUNA g2365.t1
BniN100 B05g024910.2
BniC2 B05g025110.1
Bca Nung01883
BniC2 B02g052630.1
BniN100 B02g049100.2
BniC2 B02g052530.1
Bjut B035979
Bjuv B08 VARUNA g4055.t1
Bca C04g19117
Bjuv A03 VARUNA g550.t1
Bjut A041693
BraTUE A03p05440.1
BraZ1 A03t09934
BraCCB A03p05600.1
BolKorso 3g07250.1
BolOX 3g06120.1
Bca B01g05360
Bjuv B03 VARUNA g590.t1
Bca B01gnew1
Bjut B014778
BniC2 B08g006200.1
AT5G13430.1
AT5G13440.1

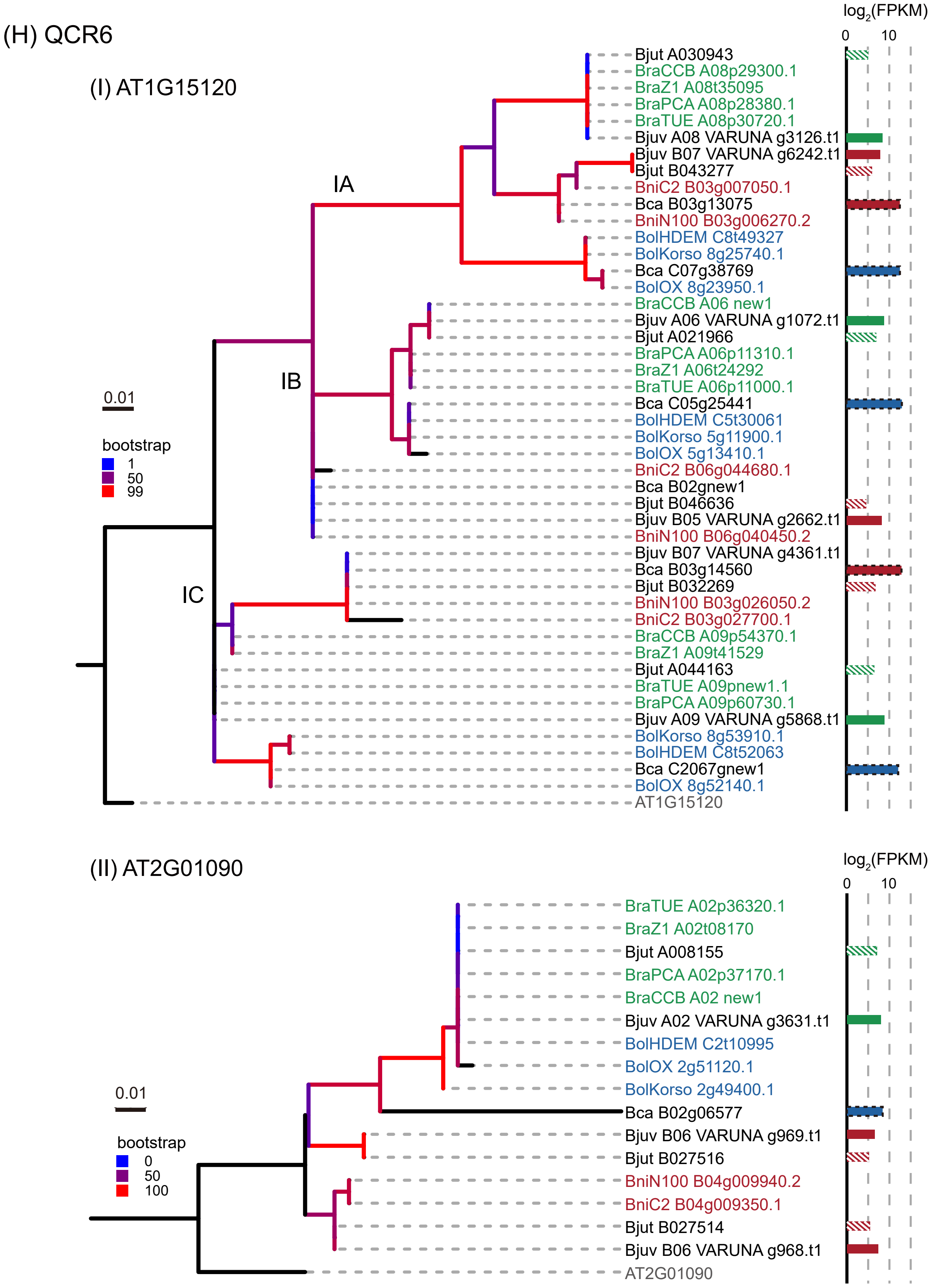
(H) QCR6
(I) AT1G15120
log2(FPKM)
0
10
IA
IB
IC
0.01
bootstrap
1
50
99
Bjut A030943
BraCCB A08p29300.1
BraZ1 A08t35095
BraPCA A08p28380.1
BraTUE A08p30720.1
Bjuv A08 VARUNA g3126.t1
Bjuv B07 VARUNA g6242.t1
Bjut B043277
BniC2 B03g007050.1
Bca B03g13075
BniN100 B03g006270.2
BolHDEM C8t49327
BolKorso 8g25740.1
Bca C07g38769
BolOX 8g23950.1
BraCCB A06 new1
Bjuv A06 VARUNA g1072.t1
Bjut A021966
BraPCA A06p11310.1
BraZ1 A06t24292
BraTUE A06p11000.1
Bca C05g25441
BolHDEM C5t30061
BolKorso 5g11900.1
BolOX 5g13410.1
BniC2 B06g044680.1
Bca B02gnew1
Bjut B046636
Bjuv B05 VARUNA g2662.t1
BniN100 B06g040450.2
Bjuv B07 VARUNA g4361.t1
Bca B03g14560
Bjut B032269
BniN100 B03g026050.2
BniC2 B03g027700.1
BraCCB A09p54370.1
BraZ1 A09t41529
Bjut A044163
BraTUE A09pnew1.1
BraPCA A09p60730.1
Bjuv A09 VARUNA g5868.t1
BolKorso 8g53910.1
BolHDEM C8t52063
Bca C2067gnew1
BolOX 8g52140.1
AT1G15120
(II) AT2G01090
log2(FPKM)
0
10
0.01
bootstrap
0
50
100
BraTUE A02p36320.1
BraZ1 A02t08170
Bjut A008155
BraPCA A02p37170.1
BraCCB A02 new1
Bjuv A02 VARUNA g3631.t1
BolHDEM C2t10995
BolOX 2g51120.1
BolKorso 2g49400.1
Bca B02g06577
Bjuv B06 VARUNA g969.t1
Bjut B027516
BniN100 B04g009940.2
BniC2 B04g009350.1
Bjut B027514
Bjuv B06 VARUNA g968.t1
AT2G01090

(I) CYC1

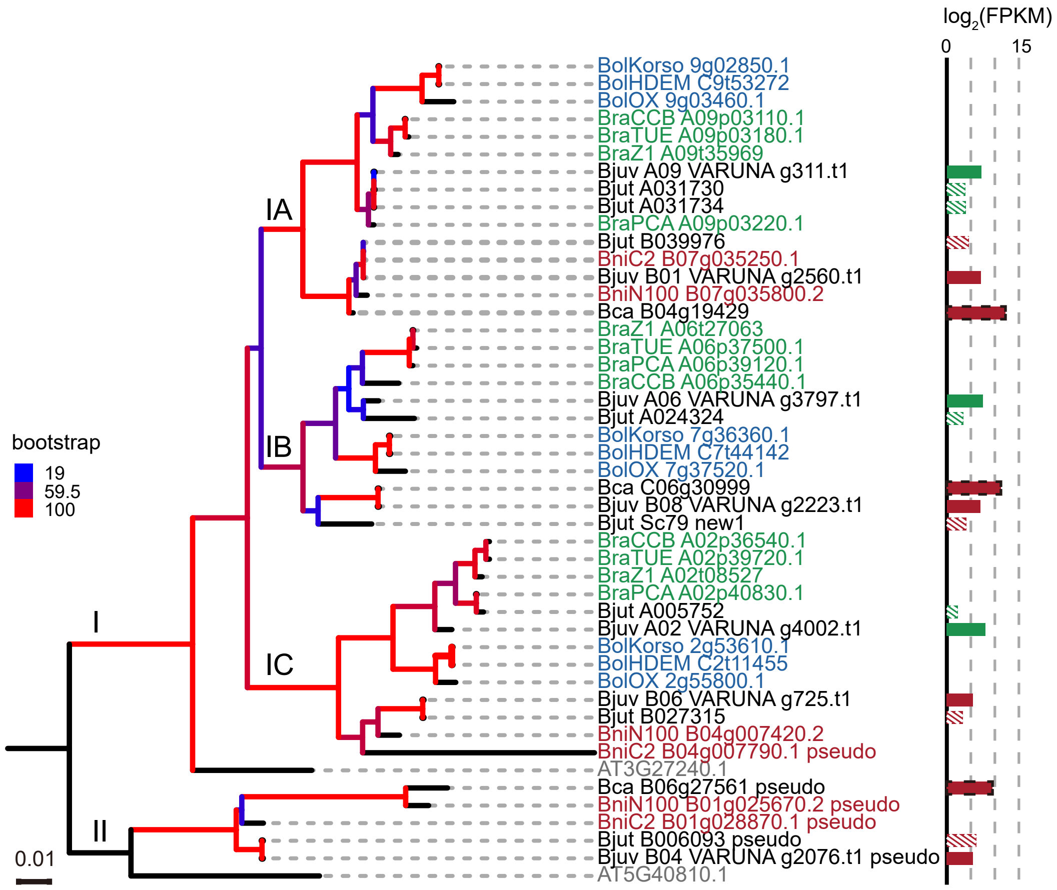

Supplementary Fig S9. Maximum likelihood phylogeny of nuclear gene families encoded the mitochondrial complex III in studied genomes/subgenomes.
