# Supplementary material for: Cytonuclear Interactions and Subgenome Dominance Shape the Evolution of Organelle-Targeted Genes in the Brassica Triangle of U
Source: Mol Biol Evol. 2024 Feb 23;41(3):msae043. doi: 10.1093/molbev/msae043 (PMC10919925; doi:10.1093/molbev/msae043)
Supplement: msae043_Supplementary_Data [file msae043_supplementary_data.zip › Supplementary Figure S10.pdf]

(A) Clade IA AABB

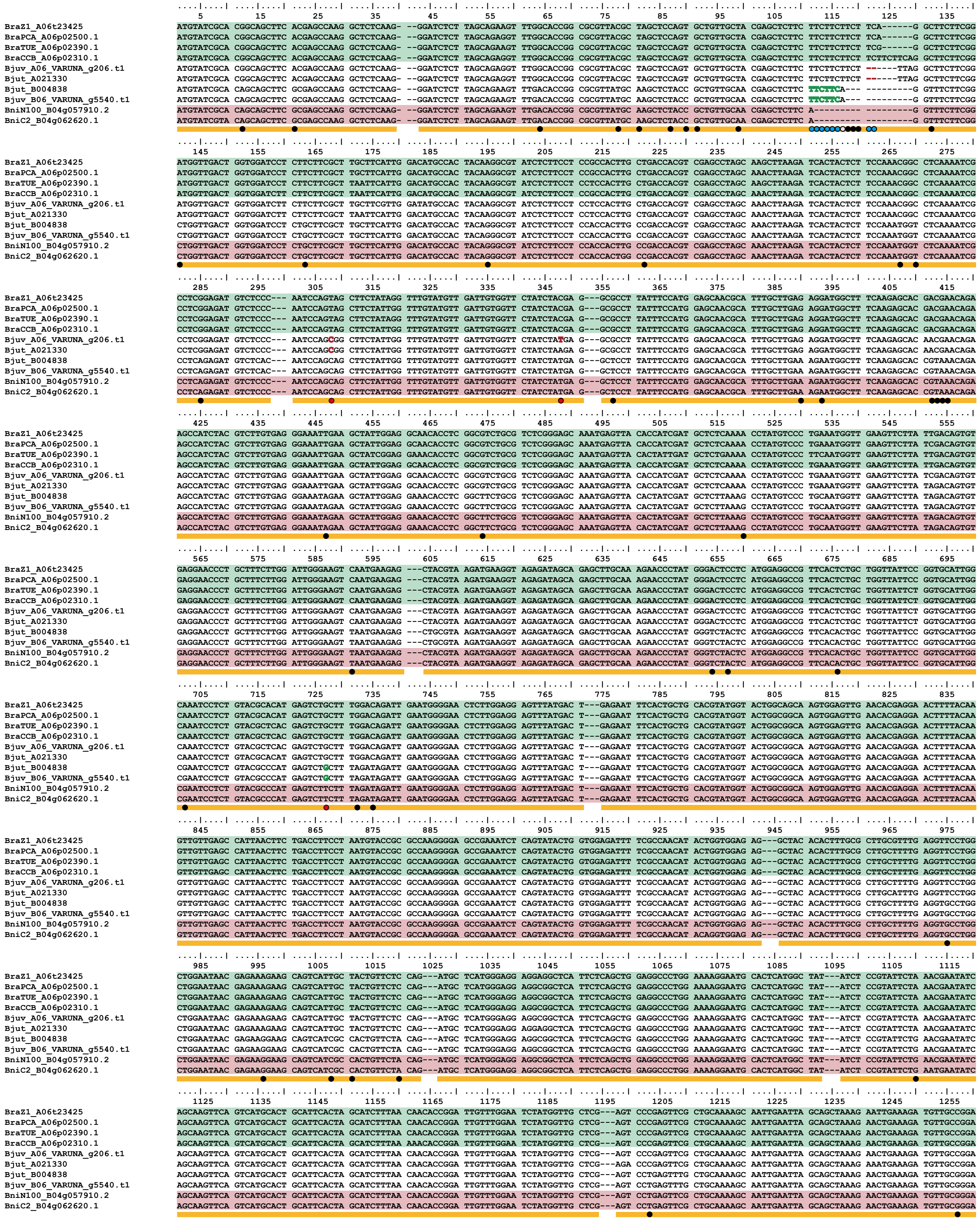

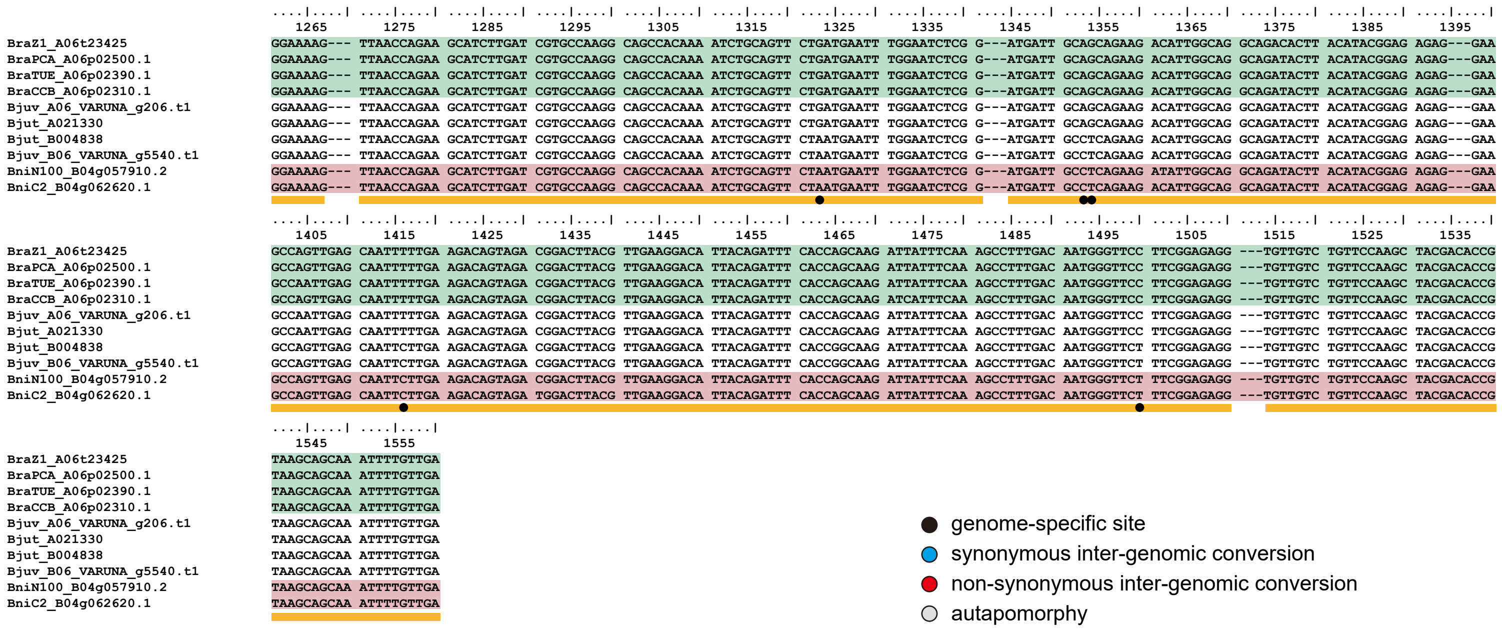
1265 1275 1285 1295 1305 1315 1325 1335 1345 1355 1365 1375 1385 1395
BraZ1_A06t23425 GGAAAAG--- TTAACCAGAA GCATCTTGAT CGTGCCAAGG CAGCCACAAA ATCTGCAGTT CTGATGAATT TGGAATCTCG G---ATGATT GCAGCAGAAG ACATTGGCAG GCAGACACTT ACATACGGAG AGAG---GAA
BraPCA_A06p02500.1 GGAAAAG--- TTAACCAGAA GCATCTTGAT CGTGCCAAGG CAGCCACAAA ATCTGCAGTT CTGATGAATT TGGAATCTCG G---ATGATT GCAGCAGAAG ACATTGGCAG GCAGATACTT ACATACGGAG AGAG---GAA
BraTUE_A06p02390.1 GGAAAAG--- TTAACCAGAA GCATCTTGAT CGTGCCAAGG CAGCCACAAA ATCTGCAGTT CTGATGAATT TGGAATCTCG G---ATGATT GCAGCAGAAG ACATTGGCAG GCAGATACTT ACATACGGAG AGAG---GAA
BraCCB_A06p02310.1 GGAAAAG--- TTAACCAGAA GCATCTTGAT CGTGCCAAGG CAGCCACAAA ATCTGCAGTT CTGATGAATT TGGAATCTCG G---ATGATT GCAGCAGAAG ACATTGGCAG GCAGATACTT ACATACGGAG AGAG---GAA
Bjuv_A06_VARUNA_g206.t1 GGAAAAG--- TTAACCAGAA GCATCTTGAT CGTGCCAAGG CAGCCACAAA ATCTGCAGTT CTGATGAATT TGGAATCTCG G---ATGATT GCAGCAGAAG ACATTGGCAG GCAGATACTT ACATACGGAG AGAG---GAA
Bjut_A021330 GGAAAAG--- TTAACCAGAA GCATCTTGAT CGTGCCAAGG CAGCCACAAA ATCTGCAGTT CTGATGAATT TGGAATCTCG G---ATGATT GCAGCAGAAG ACATTGGCAG GCAGATACTT ACATACGGAG AGAG---GAA
Bjut_B004838 GGAAAAG--- TTAACCAGAA GCATCTTGAT CGTGCCAAGG CAGCCACAAA ATCTGCAGTT CTAATGAATT TGGAATCTCG G---ATGATT GCCTCAGAAG ACATTGGCAG GCAGATACTT ACATACGGAG AGAG---GAA
Bjuv_B06_VARUNA_g5540.t1 GGAAAAG--- TTAACCAGAA GCATCTTGAT CGTGCCAAGG CAGCCACAAA ATCTGCAGTT CTAATGAATT TGGAATCTCG G---ATGATT GCCTCAGAAG ACATTGGCAG GCAGATACTT ACATACGGAG AGAG---GAA
BniN100_B04g057910.2 GGAAAAG--- TTAACCAGAA GCATCTTGAT CGTGCCAAGG CAGCCACAAA ATCTGCAGTT CTAATGAATT TGGAATCTCG G---ATGATT GCCTCAGAAG ATATTGGCAG GCAGATACTT ACATACGGAG AGAG---GAA
BniC2_B04g062620.1 GGAAAAG--- TTAACCAGAA GCATCTTGAT CGTGCCAAGG CAGCCACAAA ATCTGCAGTT CTAATGAATT TGGAATCTCG G---ATGATT GCCTCAGAAG ACATTGGCAG GCAGATACTT ACATACGGAG AGAG---GAA
1405 1415 1425 1435 1445 1455 1465 1475 1485 1495 1505 1515 1525 1535
BraZ1_A06t23425 GCCAGTTGAG CAATTTTTGA AGACAGTAGA CGGACTTACG TTGAAGGACA TTACAGATTT CACCAGCAAG ATTATTTCAA AGCCTTTGAC AATGGGTTCC TTCGGAGAGG ---TGTTGTC TGTTCCAAGC TACGACACCG
BraPCA_A06p02500.1 GCCAGTTGAG CAATTTTTGA AGACAGTAGA CGGACTTACG TTGAAGGACA TTACAGATTT CACCAGCAAG ATTATTTCAA AGCCTTTGAC AATGGGTTCC TTCGGAGAGG ---TGTTGTC TGTTCCAAGC TACGACACCG
BraTUE_A06p02390.1 GCCAATTGAG CAATTTTTGA AGACAGTAGA CGGACTTACG TTGAAGGACA TTACAGATTT CACCAGCAAG ATTATTTCAA AGCCTTTGAC AATGGGTTCC TTCGGAGAGG ---TGTTGTC TGTTCCAAGC TACGACACCG
BraCCB_A06p02310.1 GCCAGTTGAG CAATTTTTGA AGACAGTAGA CGGACTTACG TTGAAGGACA TTACAGATTT CACCAGCAAG ATCATTTCAA AGCCTTTGAC AATGGGTTCC TTCGGAGAGG ---TGTTGTC TGTTCCAAGC TACGACACCG
Bjuv_A06_VARUNA_g206.t1 GCCAATTGAG CAATTTTTGA AGACAGTAGA CGGACTTACG TTGAAGGACA TTACAGATTT CACCAGCAAG ATTATTTCAA AGCCTTTGAC AATGGGTTCC TTCGGAGAGG ---TGTTGTC TGTTCCAAGC TACGACACCG
Bjut_A021330 GCCAATTGAG CAATTTTTGA AGACAGTAGA CGGACTTACG TTGAAGGACA TTACAGATTT CACCAGCAAG ATTATTTCAA AGCCTTTGAC AATGGGTTCC TTCGGAGAGG ---TGTTGTC TGTTCCAAGC TACGACACCG
Bjut_B004838 GCCAGTTGAG CAATTCTTGA AGACAGTAGA CGGACTTACG TTGAAGGACA TTACAGATTT CACCGGCAAG ATTATTTCAA AGCCTTTGAC AATGGGTTCT TTCGGAGAGG ---TGTTGTC TGTTCCAAGC TACGACACCG
Bjuv_B06_VARUNA_g5540.t1 GCCAGTTGAG CAATTCTTGA AGACAGTAGA CGGACTTACG TTGAAGGACA TTACAGATTT CACCGGCAAG ATTATTTCAA AGCCTTTGAC AATGGGTTCT TTCGGAGAGG ---TGTTGTC TGTTCCAAGC TACGACACCG
BniN100_B04g057910.2 GCCAGTTGAG CAATTCTTGA AGACAGTAGA CGGACTTACG TTGAAGGACA TTACAGATTT CACCAGCAAG ATTATTTCAA AGCCTTTGAC AATGGGTTCT TTCGGAGAGG ---TGTTGTC TGTTCCAAGC TACGACACCG
BniC2_B04g062620.1 GCCAGTTGAG CAATTCTTGA AGACAGTAGA TGGACTTACG TTGAAGGACA TTACAGATTT CACCAGCAAG ATTATTTCAA AGCCTTTGAC AATGGGTTCT TTCGGAGAGG ---TGTTGTC TGTTCCAAGC TACGACACCG
1545 1555
BraZ1_A06t23425 TAAGCAGCAA ATTTTGTTGA
BraPCA_A06p02500.1 TAAGCAGCAA ATTTTGTTGA
BraTUE_A06p02390.1 TAAGCAGCAA ATTTTGTTGA
BraCCB_A06p02310.1 TAAGCAGCAA ATTTTGTTGA
Bjuv_A06_VARUNA_g206.t1 TAAGCAGCAA ATTTTGTTGA
Bjut_A021330 TAAGCAGCAA ATTTTGTTGA
Bjut_B004838 TAAGCAGCAA ATTTTGTTGA
Bjuv_B06_VARUNA_g5540.t1 TAAGCAGCAA ATTTTGTTGA
BniN100_B04g057910.2 TAAGCAGCAA ATTTTGTTGA
BniC2_B04g062620.1 TAAGCAGCAA ATTTTGTTGA
genome-specific site
synonymous inter-genomic conversion
non-synonymous inter-genomic conversion
autapomorphy

(B) Clade IA BBCC

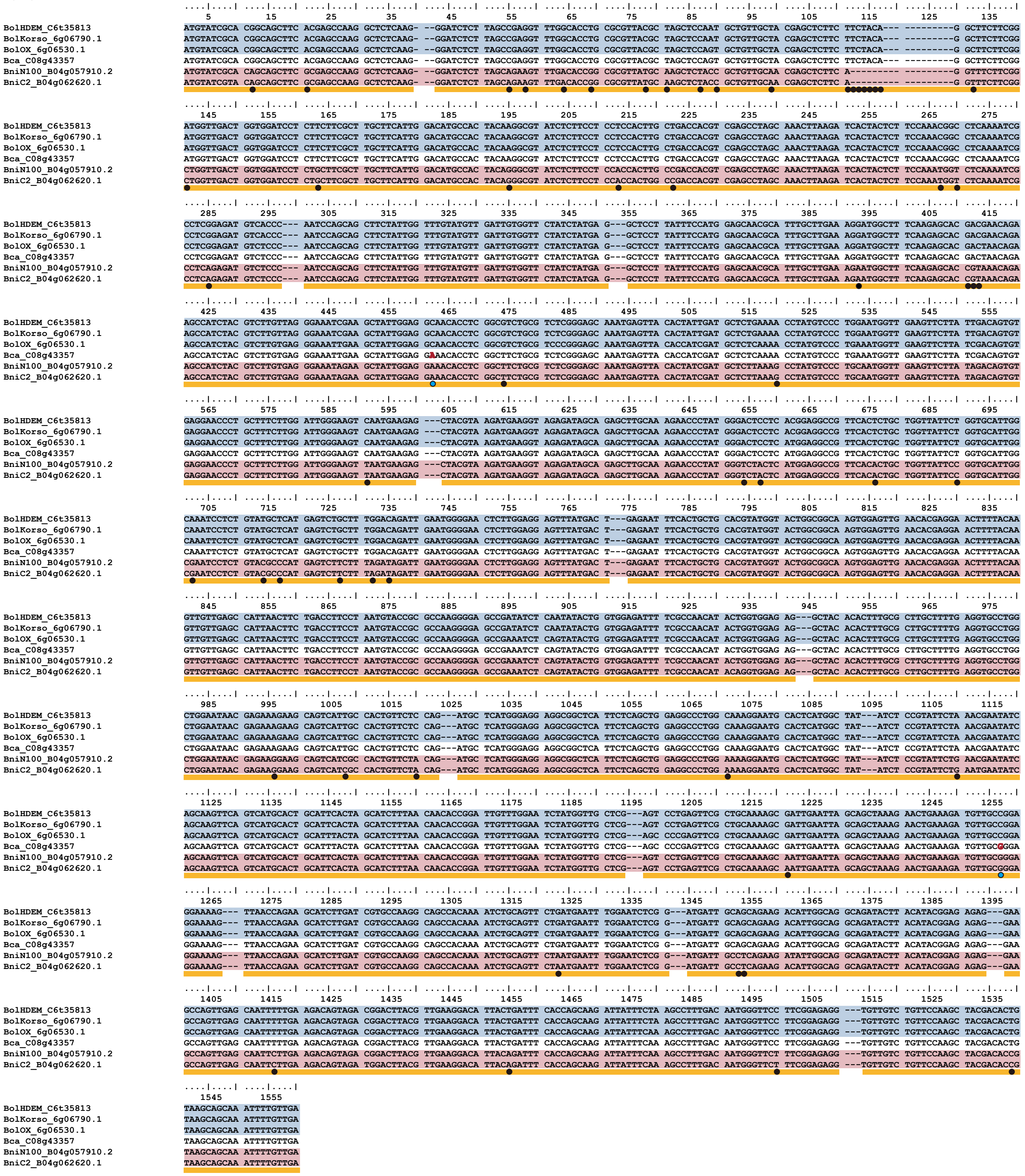

(C) Clade IB AABB

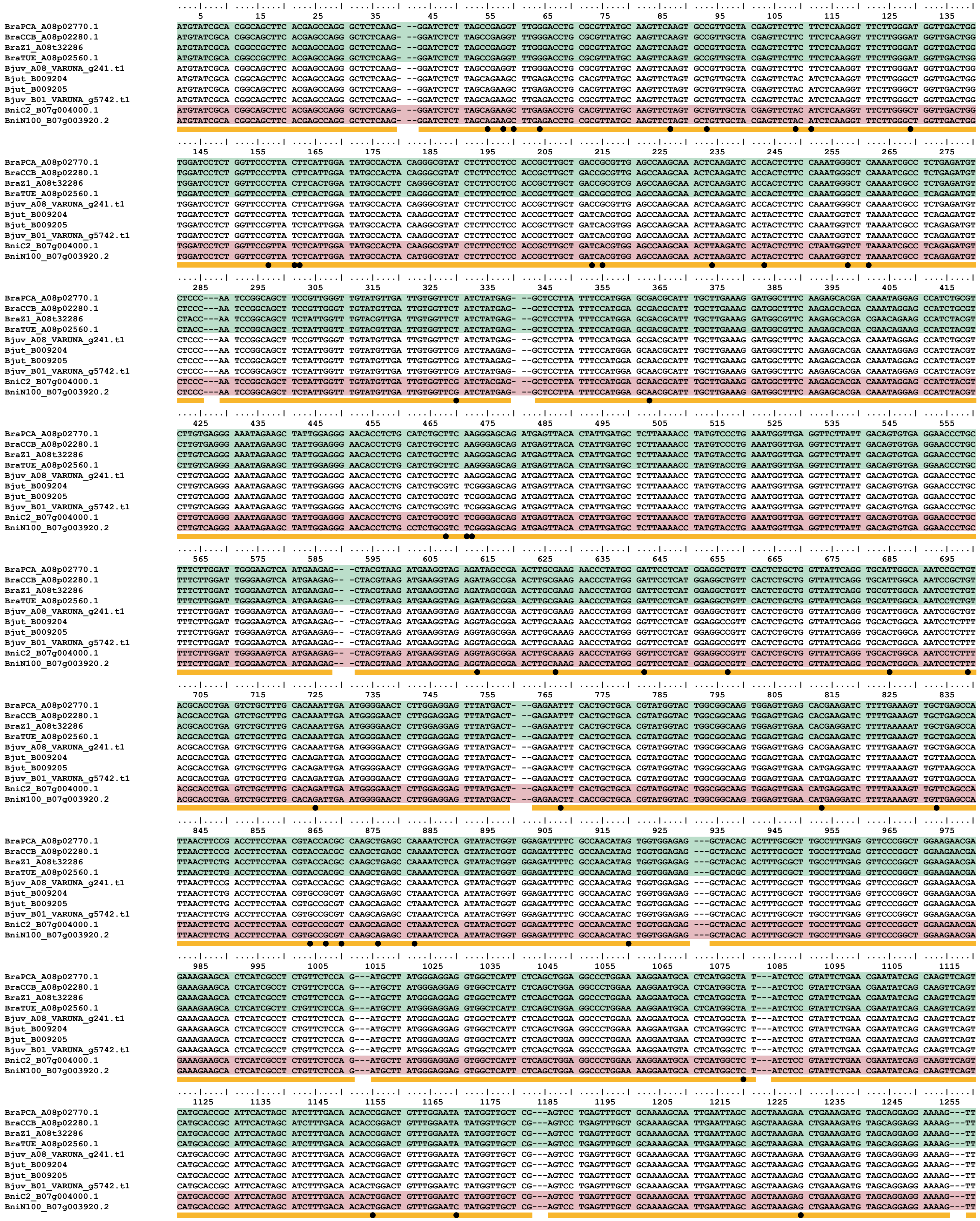

```
                             ....|....| ....|....| ....|....| ....|....| ....|....| ....|....| ....|....| ....|....| ....|....| ....|....| ....|....| ....|....| ....|....| ....|....|
                                1265       1275       1285       1295       1305       1315       1325       1335       1345       1355       1365       1375       1385       1395
BraPCA_A08p02770.1           AACCAGAAGC ATCTAGATCG TGCCAAGGCA GCCACGAAAT CTGCAGTTCT GATGAATTTG GAATCTCGG- --ATGATTGC AGCAGAAGAC ATTGGCAGGC AGATACTTAC ATACGGAGAG AG---GAAAC CAGTTGAGCA
BraCCB_A08p02280.1           AACCAGAAGC ATCTAGATCG TGCCAAGACA GCCACGAAAT CTGCAGTTCT GATGAATTTG GAATCTCGG- --ATGATTGC AGCAGAAGAC ATTGGCAGGC AGATACTTAC ATACGGAGAG AG---GAAAC CAGTTGAGCA
BraZ1_A08t32286              AACCAGAAGC ATCTAGATCG TGCCAAGGCA GCCACGAAAT CTGCAGTTCT GATGAATTTG GAATCTCGG- --ATGATTGC AGCAGAAGAC ATTGGCAGGC AGATACTTAC ATACGGAGAG AG---GAAAC CAGTTGAGCA
BraTUE_A08p02560.1           AACCAGAAGC ATCTAGATCG TGCCAAGACA GCCACGAAAT CTGCAGTTCT GATGAATTTG GAATCTCGG- --ATGATTGC AGCAGAAGAC ATTGGCAGGC AGATACTTAC ATACGGAGAG AG---GAAAC CAGTTGAGCA
Bjuv_A08_VARUNA_g241.t1      AACCAGAAGC ATCTAGATCG TGCCAAGGCA GCCACGAAAT CTGCAGTTCT GATGAATTTG GAATCTCGG- --ATGATTGC AGCAGAAGAC ATTGGCAGGC AGATACTTAC ATACGGAGAG AG---GAAAC CAGTTGAGCA
Bjut_B009204                 AACCAGAAGC ATCTTGATCG TGCCAAGGCA GCCACAAAAT CTGCAGTTCT GATGAATTTG GAATCTCGG- ---------- ---------- ---------- ---------- ---------- ---------- ----------
Bjut_B009205                 AACCAGAAGC ATCTTGATCG TGCCAAGGCA GCCACAAAAT CTGCAGTTCT GATGAATTTG GAATCTCGG- ---------- ---------- ---------- ---------- ---------- ---------- ----------
Bjuv_B01_VARUNA_g5742.t1     AACCAGAAGC ATCTTGATCG TGCCAAGGCA GCCACAAAAT CTGCAGTTCT GATGAATTTG GAATCTCGG- --ATGATTGC AGCAGAAGAC ATTGGCAGGC AGATACTTAC ATACGGAGAG AG---GAAAC CACTTGAGCA
BniC2_B07g004000.1           AACCAGAAGC ATCTTGATCG TGCCAAGGCA GCCACAAAAT CTGCAGTTCT GATGAATTTG GAATCTCGG- --ATGATTGC AGCAGAAGAC ATTGGCAGGC AGATACTTAC ATACGGAGAG AG---GAAAC CAGTTGAGCA
BniN100_B07g003920.2         AACCAGAAGC ATCTTGATCG TGCCAAGGCA GCCACAAAAT CTGCAGTTCT GATGAATTTG GAATCTCGG- --ATGATTGC AGCAGAAGAC ATTGGCAGGC AGATACTTAC ATACGGAGAG AG---GAAAC CAGTTGAGCA

                             ....|....| ....|....| ....|....| ....|....| ....|....| ....|....| ....|....| ....|....| ....|....| ....|....| ....|....| ....|....| ....|....| ....|....|
                                1405       1415       1425       1435       1445       1455       1465       1475       1485       1495       1505       1515       1525       1535
BraPCA_A08p02770.1           GTTCTTGAAG GCAGTAGACG AACTTACGTT GAAAGACATT ACAGATTTCA CCAGCAAGAT AATTTCAAAG CCTTTGACAA TGGGTTCCTT CGGAGATG-- -TGTTGTCTG TTCCGAGCTA CGACACCGTA AGCAGTAAGT
BraCCB_A08p02280.1           GTTCTTGAAG GCAGTAGACG AACTTACGTT GAAAGACATT ACAGATTTCA CCAGCAAGAT AATTTCAAAG CCTTTGACAA TGGGTTCCTT CGGAGATG-- -TGTTGTCTG TTCCGAGCTA CGACACCGTA AGCAGTAAGT
BraZ1_A08t32286              GTTCTTGAAG GCAGTAGACG AACTTACGTT GAAAGACATT ACAGATTTCA CCAGCAAGAT AATTTCAAAG CCTTTGACAA TGGGTTCCTT CGGAGATG-- -TGTTGTCTG TTCCGAGCTA CGACACCGTA CGCAGTAAGT
BraTUE_A08p02560.1           GTTCTTGAAG GCAGTAGACG AACTTACGTT GAAAGACATT ACAGATTTCA CCAGCAAGAT AATTTCAAAG CCTTTGACAA TGGGTTCCTT TGGAGATG-- -TGTTGTCTG TTCCGAGCTA CGACACCGTA AGCAGTAAGT
Bjuv_A08_VARUNA_g241.t1      GTTCTTGAAG GCAGTAGACG AACTTACGTT GAAAGACATT ACAGATTTCA CCAGCAAGAT AATTTCAAAG CCTTTGACAA TGGGTTCCTT CGGAGATG-- -TGTTGTCTG TTCCGAGCTA CGACACCGTA AGCAGTAAGT
Bjut_B009204                 ---------- ---------- ---------- ---------- ---------- ---------- ---------- ---------- ---------- ---------- ---------- ---------- ---------- ----------
Bjut_B009205                 ---------- ---------- ---------- ---------- ---------- ---------- ---------- ---------- ---------- ---------- ---------- ---------- ---------- ----------
Bjuv_B01_VARUNA_g5742.t1     GTTCTTGAAG GCAGTAGACG AACTTACGTT GAAGGACATT ACCGATTTCA CCAGCAAGAT AATTTCAAAG CCTTTAACAA TGGGTTCCTT TGGAGATG-- -TGTTGTCTG TTCCAAGCTA TGACACCGTC AGCAGCAAGT
BniC2_B07g004000.1           GTTCTTGAAG GCAGTAGACG AACTTACGTT GAAGGACATT ACCGATTTCA CCAGCAAGAT AATTTCAAAG CCTTTAACAA TGGGTTCCTT TGGAGATG-- -TGTTGTCTG TTCCAAGCTA TGACACCGTC AGCAGCAAGT
BniN100_B07g003920.2         GTTCTTGAAG GCAGTAGACG AACTTACGTT GAAGGACATT ACCGATTTCA CCAGCAAGAT AATTTCAAAG CCTTTAACAA TGGGTTCCTT TGGAGATG-- -TGTTGTCTG TTCCAAGCTA TGACACCGTC AGCAGCAAGT

                             ....|...
                                1545
BraPCA_A08p02770.1           TTTGTTGA
BraCCB_A08p02280.1           TTTGTTGA
BraZ1_A08t32286              TTTGTTGA
BraTUE_A08p02560.1           TTTGTTGA
Bjuv_A08_VARUNA_g241.t1      TTTGTTGA
Bjut_B009204                 --------
Bjut_B009205                 --------
Bjuv_B01_VARUNA_g5742.t1     TTTGTTGA
BniC2_B07g004000.1           TTTGTTGA
BniN100_B07g003920.2         TTTGTTGA
```

(D) Clade IB BBCC

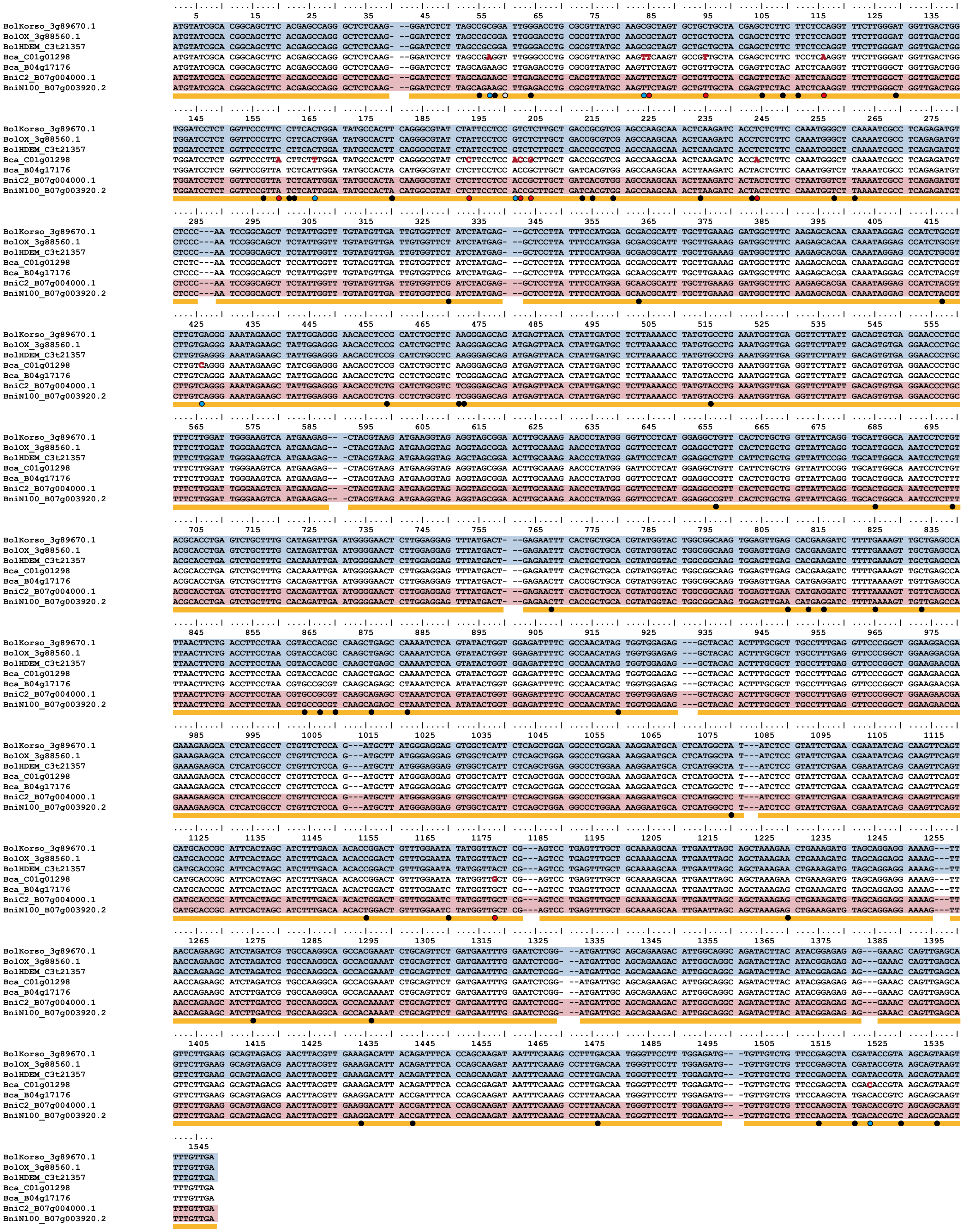

(E) Clade IC AABB

```
                            5         15         25         35         45         55         65         75         85         95        105        115        125        135
BraCCB_A05p16270.1      ATGTATCGCA CGGCAGCTTC ACGAGCCAGG GCTCTCAAG- --GGATCTCT TAGCCGGAGT TTGAGACCGG CGCGTTATGC AAGTTCAAGT GCTGTTGCGA CTAGTTCTTC TTCTTCTTCT TCTCCAGGTT TCTTCAGCTG
BraTUE_A05p17740.1      ATGTATCGCA CGGCAGCTTC ACGAGCCAGG GCTCTCAAG- --GGATCTCT TAGCCGGAGT TTGAGACCGG CGCGTTATGC AAGTTCAAGT GCTGTTGCGA CTAGTTCTTC TTCTTCTTCT TCTCCAGGTT TCTTCAGCTG
BraZ1_A05t20826         ATGTATCGCA CGGCAGCTTC ACGAGCCAGG GCTCTCAAG- --GGATCTCT TAGCCGGAGT TTGAGACCGG CGCGTTATGC AAGTTCAAGT GCTGTTGCGA CTAGTTCTTC TTCTTCTTCT TCTCCAGGTT TCTTCAGCTG
BraPCA_A05p18220.1      ATGTATCGCA CGGCAGCTTC ACGAGCCAGG GCTCTCAAG- --GGATCTCT TAGCCGGAGT TTGAGACCGG CGCGTTATGC AAGTTCAAGT GCCGTTGCGA CTAGTTCTTC TTCTTCTTCT ---CCAGGTT TCTTCAGCTG
Bjut_A019081            ATGTATCGCA CGGCAGCTTC ACGAGCCAGG GCTCTCAAG- --GGATCTCT TAGCCGGAGT TTGAGACCGG CGCGTTATGC AAGTTCAAGT GCCGTTGCGA CTAGTTCTTC TTCTTCTTCT ---CCAGGTT TCTTCAGCTG
Bjuv_A05_VARUNA_g1802.t1 ATGTATCGCA CGGCAGCTTC ACGAGCCAGG GCTCTCAAG- --GGATCTCT TAGCCGGAGT TTGAGACCGG CGCGTTATGC AAGTTCAAGT GCCGTTGCGA CTAGTTCTTC TTCTTCTTCT ---CCAGGTT TCTTCAGCTG
Bjuv_B05_VARUNA_g3772.t1 ATGTATCGCA CGGCAGCTTC GCGAGCCAGG GCTCTCAAG- --GGATCCCT GAGCCGGAGT TTGAGACCGG CGCGTTATGC AAGTTCTAGT GCTGTTGGTA CTAGTTCGTC TTCTTCTTCT TCTCCAGGTT TCTTCAGCTG
BniC2_B06g033440.1      ATGTATCGCA CGGCAGCTTC GCGAGCCAGG GCTCTCAAG- --GGATCCCT GAGCCGGAGT TTGAGACCGG CGCGTTATGC AAGTTCTAGT GCTGTTGGTA CTAGTTCGTC TTCTTCTTCT TCTCCAGGTT TCTTCAGCTG
BniN100_B06g028650.2    ATGTATCGCA CGGCAGCTTC GCGAGCCAGG GCTCTCAAG- --GGATCCCT GAGCCGGAGT TTGAGACCGG CGCGTTATGC AAGTTCTAGT GCTGTTGGTA CTAGTTCGTC TTCTTCTTCT TCTCCAGGTT TCTTCAGCTG

                          145        155        165        175        185        195        205        215        225        235        245        255        265        275
BraCCB_A05p16270.1      GTTGACTGGG GGATCATCTT CTTCGCTTAA TTCATTGGAC ATGCCACTAC AGGGTGTGTC TCTTCCTGCA CCACTTGCTG ACCTTGTTGA GCCAAGCAAA CTTAAGATCA CTACTCTCCC AAATGGTCTC AAAATCGCCT
BraTUE_A05p17740.1      GTTGACTGGG GGATCATCTT CTTCGCTTAA TTCATTGGAC ATGCCACTAC AGGGTGTGTC TCTTCCTCCA CCACTTGCTG ACCTTGTTGA GCCAAGCAAA CTTAAGATCA CTACTCTCCC AAATGGTCTC AAAATCGCCT
BraZ1_A05t20826         GTTGACTGGG GGATCATCTT CTTCGCTTAA TTCATTGGAC ATGCCACTAC AGGGTGTGTC TCTTCCTCCA CCACTTGCTG ACCTTGTTGA GCCAAGCAAA CTTAAGATCA CTACTCTCCC AAATGGTCTC AAAATCGCCT
BraPCA_A05p18220.1      GTTGACTGGT GGATCATCTT CTTCGCTTAA TTCATTGGAC ATGCCACTAC AGGGTGTGTC TCTTCCTCCA CCACTTGCTG ACCTTGTTGA GCCAAGCAAA CTTAAGATCA CTACTCTCCC AAATGGTCTC AAAATCGCCT
Bjut_A019081            GTTGACTGGT GGATCATCTT CTTCGCTTAA TTCATTGGAC ATGCCACTAC AGGGTGTGTC TCTTCCTCCA CCACTTGCTG ACCTTGTTGA GCCAAGCAAA CTTAAGATCA CTACTCTCCC AAATGGTCTC AAAATCGCCT
Bjuv_A05_VARUNA_g1802.t1 GTTGACTGGT GGATCATCTT CTTCGCTTAA TTCATTGGAC ATGCCACTAC AGGGTGTGTC TCTTCCTCCA CCACTTGCTG ACCTTGTTGA GCCAAGCAAA CTTAAGATCA CTACTCTCCC AAATGGTCTC AAAATCGCCT
Bjuv_B05_VARUNA_g3772.t1 GTTGACTGGT GGATCCTCTG CTTCGCTTGC TTCATTGGAC ATGCCACTAC AGGGTGTATC TCTTCCTCCG CCACTTGCTG ACCACGTTGA GCCAAGCAAA CTTAAGATCA CTACTCTTTC TAATGGTCTC AAAATCGCTT
BniC2_B06g033440.1      GTTGACTGGT GGATCCTCTG CTTCGCTTGC TTCATTGGAC ATGCCACTAC AGGGTGTATC TCTTCCTCCG CCACTTGCTG ACCACGTTGA GCCAAGCAAA CTTAAGATCA CTACTCTTTC TAATGGTCTC AAAATCGCCT
BniN100_B06g028650.2    GTTGACTGGT GGATCCTCTG CTTCGCTTGC TTCATTGGAC ATGCCACCTA TGAAGCACGG GTACGTCTGT TTAGGTGCCG TACCGGTACC GGAAACGTTT CGGGGACGGA TACTTCACGG GGACATCACT GGTACGGCTT

                          285        295        305        315        325        335        345        355        365        375        385        395        405        415
BraCCB_A05p16270.1      CAGAGATGTC TCCC------ AATCCGGCAG CTTCCATTGG TTTGTATGTT GATTGTGGCT CTATCTACGA A---GCTCCT TATTTCCATG GAGCAACGCA TTTGCTTGAG AGGATGGCTT TCAAGAGCAC GAGAAACAGG
BraTUE_A05p17740.1      CAGAGATGTC TCCC------ AATCCGGCAG CTTCCATTGG TTTGTATGTT GATTGTGGCT CTATCTACGA A---GCTCCT TATTTCCATG GAGCAACGCA TTTGCTTGAG AGGATGGCTT TCAAGAGCAC GAGAAACAGG
BraZ1_A05t20826         CAGAGATGTC TCCC------ AATCCGGCAG CTTCCATTGG TTTGTATGTT GATTGTGGCT CTATCTACGA A---GCTCCT TATTTCCATG GAGCAACGCA TTTGCTTGAG AGGATGGCTT TCAAGAGCAC GAGAAACAGG
BraPCA_A05p18220.1      CCGAGATGTC TCCC------ AATCCAGCAG CTTCCATTGG TTTGTATGTT GATTGTGGTT CTATCTACGA A---GCTCCT TATTTCCATG GAGCAACGCA TTTGCTTGAG AGGATGGCTT TCAAGAGCAC GAGAAACAGG
Bjut_A019081            CCGAGATGTC TCCC------ AATCCGGCAG CTTCCATTGG TTTGTATGTT GATTGTGGCT CTATCTACGA A---GCTCCT TATTTCCATG GAGCAACGCA TTTGCTTGAG AGGATGGCTT TCAAGAGCAC GAGAAACAGG
Bjuv_A05_VARUNA_g1802.t1 CCGAGATGTC TCCC------ AATCCGGCAG CTTCCATTGG TTTGTATGTT GATTGTGGCT CTATCTACGA A---GCTCCT TATTTCCATG GAGCAACGCA TTTGCTTGAG AGGATGGCTT TCAAGAGCAC GAGAAACAGG
Bjuv_B05_VARUNA_g3772.t1 CAGAGATGTC TCCC------ AATCCGGCAG CTTCCATTGG TTTGTATGTT GATTGTGGTT CTATCTATGA G---GCTCCT TATTTCCATG GAGCAACGCA TTTGCTTGAG AGGATGGCTT TCAAGAGCAC GAGAAACAGA
BniC2_B06g033440.1      CAGAGATGTC TCCC------ AATCCGGCAG CTTCCATTGG TTTGTATGTT GATTGTGGTT CTATCTATGA G---GCTCCT TATTTCCATG GAGCAACGCA TTTGCTTGAG AGGATGGCTT TCAAGAGCAC GAGAAACAGA
BniN100_B06g028650.2    GGGGACGGTT AGAGAAC--- AATCCGGCAG CTTCCATTGG TTTGTATGTT GATTGTGGTT CTATCTATGA G---GCTCCT TATTTCCATG GAGCAACGCA TTTGCTTGAG AGGATGGCTT TCAAGAGCAC GAGAAACAGA

                          425        435        445        455        465        475        485        495        505        515        525        535        545        555
BraCCB_A05p16270.1      AGCCATCTAC GTCTCGTGAG GGAGATTGAG GCTATTGGAG GCAACACCTC CGCATCTGCG TCTCGGGAGC AGATGAGTTA CACTATTGAT GCTCTTAAGA CCTATGTCCC TGAAATGGTT GAAGTTCTTG TTGACAGTGT
BraTUE_A05p17740.1      AGCCATCTAC GTCTCGTGAG GGAGATTGAG GCTATTGGAG GCAACACCTC CGCATCTGCG TCTCGGGAGC AGATGAGTTA CACTATTGAT GCTCTTAAGA CCTATGTCCC TGAAATGGTT GAAGTTCTTA TTGACAGTGT
BraZ1_A05t20826         AGCCATCTAC GTCTCGTGAG GGAGATTGAG GCTATTGGAG GCAACACCTC CGCATCTGCG TCTCGGGAGC AGATGAGTTA CACTATTGAT GCTCTTAAGA CCTATGTCCC TGAAATGGTT GAAGTTCTTG TTGACAGTGT
BraPCA_A05p18220.1      AGCCATCTAC GTCTCGTGAG GGAGATTGAG GCTATTGGAG GCAACACCTC CGCATCTGCG TCTCGGGAGC AGATGAGTTA CACTATTGAT GCTCTTAAGA CCTATGTCCC TGAAATGGTT GAAGTTCTTA TTGACAGTGT
Bjut_A019081            AGCCATCTAC GTCTCGTGAG GGAGATTGAG GCTATTGGAG GCAACACCTC CGCATCTGCG TCTCGGGAGC AGATGAGTTA CACTATTGAT GCTCTTAAGA CCTATGTCCC TGAAATGGTT GAAGTTCTTA TTGACAGTGT
Bjuv_A05_VARUNA_g1802.t1 AGCCATCTAC GTCTCGTGAG GGAGATTGAG GCTATTGGAG GCAACACCTC CGCATCTGCG TCTCGGGAGC AGATGAGTTA CACTATTGAT GCTCTTAAGA CCTATGTCCC TGAAATGGTT GAAGTTCTTA TTGACAGTGT
Bjuv_B05_VARUNA_g3772.t1 AGCCACCTAC GTCTTGTGAG GGAGATAGAA GCTATCGGAG GCAACACCTC CGCATCTGCG TCTCGGGAGC AGATGAGTTA CACTATTGAT GCTCTTAAAA CCTATGTCCC TGAAATGGTT GAAGTTCTTA TTGACAGTGT
BniC2_B06g033440.1      AGCCACCTAC GTCTTGTGAG GGAGATAGAA GCTATCGGAG GCAACACCTC CGCATCTGCG TCTCGGGAGC AGATGAGTTA CACTATTGAT GCTCTTAAAA CCTATGTCCC TGAAATGGTT GAAGTTCTTA TTGACAGTGT
BniN100_B06g028650.2    AGCCACCTAC GTCTTGTGAG GGAGATAGAA GCTATCGGAG GCAACACCTC CGCATCTGCG TCTCGGGAGC AGATGAGTTA CACTATTGAT GCTCTTAAAA CCTATGTCCC TGAAATGGTT GAAGTTCTTA TTGACAGTGT

                          565        575        585        595        605        615        625        635        645        655        665        675        685        695
BraCCB_A05p16270.1      GAGGAACCCT GCTTTCTTGG ATTGGGAAGT CAACGAAGAG ---CTACGTA AGATGAAGCT AGAGATAGCG GAACTTGCAA ATAACCCTAT GGGCCTTCTC ATGGAGGCCG TTCACTCTGC TGGTTATTCC GGTGCATTGG
BraTUE_A05p17740.1      GAGGAACCCT GCTTTCTTGG ATTGGGAAGT CAACGAAGAG ---CTACGTA AGATGAAGCT AGAGATAGCG GAACTTGCAA ATAACCCTAT GGGCCTTCTC ATGGAGGCCG TTCACTCTGC TGGTTATTCC GGTGCATTGG
BraZ1_A05t20826         GAGGAACCCT GCTTTCTTGG ATTGGGAAGT CAACGAAGAG ---CTACGTA AGATGAAGCT AGAGATAGCG GAACTTGCAA ATAACCCTAT GGGCCTTCTC ATGGAGGCCG TTCACTCTGC TGGTTATTCC GGTGCATTGG
BraPCA_A05p18220.1      GAGGAACCCT GCTTTCTTGG ATTGGGAAGT CAACGAAGAG ---CTACGTA AGATGAAGCT AGAGATAGCG GAACTTGCAA AGAACCCTAT GGGCCTTCTC ATGGAGGCCG TTCACTCTGC TGGTTATTCC GGTGCATTGG
Bjut_A019081            GAGGAACCCT GCTTTCTTGG ATTGGGAAGT CAACGAAGAG ---CTACGTA AGATGAAGCT AGAGATAGCG GAACTTGCAA AGAACCCTAT GGGCCTTCTC ATGGAGGCCG TTCACTCTGC TGGTTATTCC GGTGCATTGG
Bjuv_A05_VARUNA_g1802.t1 GAGGAACCCT GCTTTCTTGG ATTGGGAAGT CAACGAAGAG ---CTACGTA AGATGAAGCT AGAGATAGCG GAACTTGCAA AGAACCCTAT GGGCCTTCTC ATGGAGGCCG TTCACTCTGC TGGTTATTCC GGTGCATTGG
Bjuv_B05_VARUNA_g3772.t1 GAGGAACCCT GCTTTCTTGG ATTGGGAAGT CAATGAAGAG ---TTACGTA AGATGAAGCT AGAGATAGCG GAACTTGCAA AGAACCCTAT GGGACTTCTC ATGGAGGCTG TTCATTCTGC TGGTTATTCT GGTGCATTGG
BniC2_B06g033440.1      GAGGAACCCT GCTTTCTTGG ATTGGGAAGT CAATGAAGAG ---TTACGTA AGATGAAGCT AGAGATAGCG GAACTTGCAA AGAACCCTAT GGGACTTCTC ATGGAGGCTG TTCATTCTGC TGGTTATTCT GGTGCATTGG
BniN100_B06g028650.2    GAGGAACCCT GCTTTCTTGG ATTGGGAAGT CAATGAAGAG ---TTACGTA AGATGAAGCT AGAGATAGCG GAACTTGCAA AGAACCCTAT GGGACTTCTC ATGGAGGCTG TTCATTCTGC TGGTTATTCT GGTGCATTGG

                          705        715        725        735        745        755        765        775        785        795        805        815        825        835
BraCCB_A05p16270.1      CGAATCCTCT CTACGCACCT GAGTCTGCTT TGGACAGATT GAATGGGGAA CTCTTGGAGG AGTTTATGGC T---GAGAAT TTCACTGCTG CACGTATGGT GCTGGCGGCA AGTGGAGTTG AACACGAGGA ACTTTAAAAA
BraTUE_A05p17740.1      CGAATCCTCT CTACGCACCT GAGTCTGCTT TGGACAGATT GAATGGGGAA CTCTTGGAGG AGTTTATGGC T---GAGAAT TTCACTGCTG CACGTATGGT GCTGGCGGCA AGTGGAGTTG AACACGAGGA ACTTTTAAAA
BraZ1_A05t20826         CGAATCCTCT CTACGCACCT GAGTCTGCTT TGGACAGATT GAATGGGGAA CTCTTGGAGG AGTTTATGGC T---GAGAAT TTCACTGCTG CACGTATGGT GCTGGCGGCA AGTGGAGTTG AACACGAGGA ACTTTTAAAA
BraPCA_A05p18220.1      CGAATCCTCT CTACGCACCT GAGTCTGCTT TGGACAGATT GAATGGGGAA CTCTTGGAGG AGTTTATGGC T---GAGAAT TTCACTGCTG CACGTATGGT GCTGGCGGCA AGTGGAGTTG AACACGAGGA ACTTCTAAAA
Bjut_A019081            CGAATCCTCT CTACGCACCT GAGTCTGCTT TGGACAGATT GAATGGGGAA CTCTTGGAGG AGTTTATGGC T---GAGAAT TTCACTGCTG CACGTATGGT GCTGGCGGCA AGTGGAGTTG AACACGAGGA ACTTTTAAAA
Bjuv_A05_VARUNA_g1802.t1 CGAATCCTCT CTACGCACCT GAGTCTGCTT TGGACAGATT GAATGGGGAA CTCTTGGAGG AGTTTATGGC T---GAGAAT TTCACTGCTG CACGTATGGT GCTGGCGGCA AGTGGAGTTG AACACGAGGA ACTTTTAAAA
Bjuv_B05_VARUNA_g3772.t1 CAAATCCTCT TTACGCACCT GAGTCTGCGT TGGACAGATT GAACGGAGAA CTCTTGGAGG AGTTTATGAC C---GAGAAT TTCACTGCTG CGCGTATGGT GCTGGCGGCA AGTGGAGTTG AACACGAGGA ACTTCTAAAA
BniC2_B06g033440.1      CAAATCCTCT TTACGCACCT GAGTCTGCGT TGGACAGATT GAACGGAGAA CTCTTGGAGG AGTTTATGAC C---GAGAAT TTCACTGCTG CGCGTATGGT GCTGGCGGCA AGTGGAGTTG AACACGAGGA ACTTCTAAAA
BniN100_B06g028650.2    CAAATCCTCT TTACGCACCT GAGTCTGCGT TGGACAGATT GAACGGAGAA CTCTTGGAGG AGTTTATGAC C---GAGAAT TTCACTGCTG CGCGTATGGT GCTGGCGGCA AGTGGAGTTG AACACGAGGA ACTTCTAAAA

                          845        855        865        875        885        895        905        915        925        935        945        955        965        975
BraCCB_A05p16270.1      GTTGTTGAGC CATTGACTTC TGACCTTCCT AATGTAACGC GCCAGGCGGA ACCAAAATCT CAGTATACTG GTGGAGATTT CCGCCAACAT ACTGGTGGAG AG---GCTAC GCACTTTGCT CTCGCCTTTG AGGTTCCTGG
BraTUE_A05p17740.1      GTTGTTGAGC CATTGACTTC TGACCTTCCT AATGTAACGC GCCAGGCGGA ACCAAAATCT CAGTATACTG GTGGAGATTT CCGCCAACAT ACTGGTGGAG AG---GCTAC GCACTTTGCT CTCGCCTTTG AGGTTCCTGG
BraZ1_A05t20826         GTTGTTGAGC CATTGACTTC TGACCTTCCT AATGTAACGC GCCAGGCGGA ACCAAAATCT CAGTATACTG GTGGAGATTT CCGCCAACAT ACTGGTGGAG AG---GCTAC GCACTTTGCT CTCGCCTTTG AGGTTCCTGG
BraPCA_A05p18220.1      GTTGTTGAGC CGTTGACTTC AGACCTTCCT AATGTACCGC GCCAGGCGGA ACCAAAATCT CAGTATACTG GTGGAGATTT CCGCCAACAT ACTGGTGGAG AG---GCTAC GCACTTTGCT CTCGCCTTTG AGGTTCCTGG
Bjut_A019081            GTTGTTGAGC CATTGACTTC TGACCTTCCT AATGTACCGC GCCAGGCGGA ACCGAAATCT CAGTATACTG GTGGAGATTT CCGCCAACAT ACTGGTGGAG AG---GCTAC GCACTTTGCT CTCGCCTTTG AGGTTCCTGG
Bjuv_A05_VARUNA_g1802.t1 GTTGTTGAGC CGTTGACTTC AGACCTTCCT AATGTACCGC GCCAGGCGGA ACCAAAATCT CAGTATACTG GTGGAGATTT CCGCCAACAT ACTGGTGGAG AG---GCTAC GCACTTTGCT CTCGCCTTTG AGGTTCCTGG
Bjuv_B05_VARUNA_g3772.t1 GTTGTTGAGC CATTAACTTC TGACCTTCCT AATGTACCGC GCCAGGCGGA ACCGAAATCT CAGTATACTG GTGGAGATTT CGCCCAACAT ACTGGTGGAG AG---GCTAC ACACTTTGCT CTTGCCTTTG AGGTTCCTGG
BniC2_B06g033440.1      GTTGTTGAGC CATTAACTTC TGACCTTCCT AATGTACCGC GACAGGTGGA ACCGAAATCT CAGTATACTG GTGGAGATTT CCGCCAACAT ACTGGTGGAG AG---GCTAC ACACTTTGCT CTTGCCTTTG AGGTTCCTGG
BniN100_B06g028650.2    GTTGTTGAGC CATTAACTTC TGACCTTCCT AATGTACCGC GACAGGTGGA ACCGAAATCT CAGTATACTG GTGGAGATTT CCGCCAACAT ACTGGTGGAG AG---GCTAC ACACTTTGCT CTTGCCTTTG AGGTTCCTGG

                          985        995       1005       1015       1025       1035       1045       1055       1065       1075       1085       1095       1105       1115
BraCCB_A05p16270.1      CTGGAATAAC GAGAAAGAAG CAGTCATCGC CACTGTTCTC CAG---ATGC TTATGGGAGG AGGTGGCTCG TTCTCTGCTG GAGGCCCTGG AAAAGGAATG CACTCATGGC TGT---ATCT CCGTATTCTT AACGAATACC
BraTUE_A05p17740.1      CTGGAATAAC GAGAAAGAAG CAGTCATCGC CACTGTTCTC CAG---ATGC TTATGGGAGG AGGTGGCTCG TTCTCTGCTG GAGGCCCTGG AAAAGGAATG CACTCATGGC TGT---ATCT CCGTATTCTT AACGAATACC
BraZ1_A05t20826         CTGGAATAAC GAGAAAGAAG CAGTCATCGC CACTGTTCTC CAG---ATGC TTATGGGAGG AGGTGGCTCG TTCTCTGCTG GAGGCCCTGG AAAAGGAATG CACTCATGGC TGT---ATCT CCGTATTCTT AACGAATACC
BraPCA_A05p18220.1      CTGGAATAAC GAGAAAGAAG CAGTCATCGC CACTGTTCTC CAG---ATGC TTATGGGAGG AGGTGGCTCG TTCTCTGCTG GAGGCCCTGG AAAAGGAATG CACTCATGGC TGT---ATCT CCGTATTCTT AACGAATACC
Bjut_A019081            CTGGAATAAC GAGAAAGAAG CAGTCATCGC CACTGTTCTC CAG---ATGC TTATGGGAGG AGGTGGCTCG TTCTCTGCTG GAGGCCCTGG AAAAGGAATG CACTCATGGC TAT---ATCT CCGTATTCTT AACGAATACC
Bjuv_A05_VARUNA_g1802.t1 CTGGAATAAC GAGAAAGAAG CAGTCATCGC CACTGTTCTC CAG---ATGC TTATGGGAGG AGGTGGCTCG TTCTCTGCTG GAGGCCCTGG AAAAGGAATG CACTCATGGC TGT---ATCT CCGTATTCTT AACGAATACC
Bjuv_B05_VARUNA_g3772.t1 CTGGAATAAC GAGAAAGAAG CAGTCATCGC CACTGTTCTC CAG---ATGC TATTGGGAGG AGGTGGCTCG TTCTCTGCTG GAGGCCCTGG AAAAGGAATG CACTCATGGC TCT---ATCT CCGTATTCTA AACGAATATC
BniC2_B06g033440.1      CTGGAATAAC GAGAAAGAAG CAGTCATCGC CACTGTTCTC CAG---ATGC TATTGGGAGG AGGTGGCTCG TTCTCTGCTG GAGGCCCTGG AAAAGGAATG CACTCATGGC TCT---ATCT CCGTATTCTA AACGAATATC
BniN100_B06g028650.2    CTGGAATAAC GAGAAAGAAG CAGTCATCGC CACTGTTCTC CAG---ATGC TATTGGGAGG AGGTGGCTCG TTCTCTGCTG GAGGCCCTGG AAAAGGAATG CACTCATGGC TCT---ATCT CCGTATTCTA AACGAATATC

                         1125       1135       1145       1155       1165       1175       1185       1195       1205       1215       1225       1235       1245       1255
BraCCB_A05p16270.1      AGCAAGTGCA GTCATGCACT GCTTTCAGTA GCATCTTTAA CAACACCGGG TTGTTTGGAA TCTATGGTTG CTCG---AGT CCCGAGTTCG CTGCAAAAGC AATTGAATTA GCAGCTAAAG AAATGAAAGA TGTTGCCGGA
BraTUE_A05p17740.1      AGCAAGTGCA GTCATGCACT GCTTTCAGTA GCATCTTTAA CAACACCGGG TTGTTTGGAA TCTATGGTTG CTCG---AGT CCCGAGTTCG CTGCAAAAGC AATTGAATTA GCAGCTAAAG AAATGAAAGA TGTTGCCGGA
BraZ1_A05t20826         AGCAAGTGCA GTCATGCACT GCTTTCAGTA GCATCTTTAA CAACACCGGG TTGTTTGGAA TCTATGGTTG CTCG---AGT CCCGAGTTCG CTGCAAAAGC AATTGAATTA GCAGCTAAAG AAATGAAAGA TGTTGCCGGA
BraPCA_A05p18220.1      AGCAAGTGCA GTCATGCACT GCTTTCAGTA GCATCTTTAA CAACACCGGG TTGTTTGGAA TCTATGGTTG CTCG---AGT CCCGAGTTCG CTGCAAAAGC AATTGAATTA GCAGCTAAAG AAATGAAAGA TGTTGCCGGA
Bjut_A019081            AGCAAGTGCA GTCATGCACT GCTTTCAGTA GCATCTTTAA CAACACCGGG TTGTTTGGAA TCTATGGTTG CTCG---AGT CCCGAGTTCG CTGCAAAAGC AATTGAATTA GCAGCTAAAG AAATGAAAGA TGTTGCCGGA
Bjuv_A05_VARUNA_g1802.t1 AGCAAGTGCA GTCATGCACT GCTTTCAGTA GCATCTTTAA CAACACCGGG TTGTTTGGAA TCTATGGTTG CTCG---AGT CCCGAGTTCG CTGCAAAAGC AATTGAATTA GCAGCTAAAG AAATGAAAGA TGTTGCCGGA
Bjuv_B05_VARUNA_g3772.t1 AGCAAGTTCA GTCATGCACT GCATTCAGTA GCATCTTTAA CAACACCGGG TTGTTTGGAA TCTATGGTTG CTCG---AGT CCCGAGTTCG CTGCAAAAGC AATTGAATTA GCAGCTAAAG AACTTAAAGA TGTTGCCGGA
BniC2_B06g033440.1      AGCAAGTTCA GTCATGCACT GCATTCAGTA GCATCTTTAA CAACACCGGA TTGTTTGGAA TCTATGGTTG CTCG---AGT CCCGAGTTCG CTGCAAAAGC AATTGAATTA GCAGCTAAAG AACTTAAAGA TGTTGCCGGA
BniN100_B06g028650.2    AGCAAGTTCA GTCATGCACT GCATTCAGTA GCATCTTTAA CAACACCGGA TTGTTTGGAA TCTATGGTTG CTCG---AGT CCCGAGTTCG CTGCAAAAGC AATTGAATTA GCAGCTAAAG AACTTAAAGA TGTTGCCGGA
```

```
                             ....|....| ....|....| ....|....| ....|....| ....|....| ....|....| ....|....| ....|....| ....|....| ....|....| ....|....| ....|....| ....|....| ....|....|
                                   1265       1275       1285       1295       1305       1315       1325       1335       1345       1355       1365       1375       1385       1395
BraCCB_A05p16270.1           GGAAAAG--- TTAATCAGAA GCATCTTGAT CGCGCCAAGG CAGCCACAAA ATCTGCAGTT CTGATGAATT TGGAATCACG G---ATGATT GCAGCAGAAG ACATTGGCAG GCAGATTCTT ACATACGGAG AGAG---GAA
BraTUE_A05p17740.1           GGAAAAG--- TTAATCAGAA GCATCTTGAT CGCGCCAAGG CAGCCACAAA ATCTGCAGTT CTGATGAATT TGGAATCACG G---ATGATT GCAGCAGAAG ACATTGGCAG GCAGATTCTT ACATACGGAG AGAG---GAA
BraZ1_A05t20826              GGAAAAG--- TTAATCAGAA GCATCTTGAT CGCGCCAAGG CAGCCACAAA ATCTGCAGTT CTGATGAATT TGGAATCACG G---ATGATT GCAGCAGAAG ACATTGGCAG GCAGATTCTT ACATACGGAG AGAG---GAA
BraPCA_A05p18220.1           GGAAAAG--- TTAATCAGAA GCATCTTGAT CGCGCCAAGG CAGCCACAAA ATCTGCAGTT CTGATGAATT TGGAATCACG G---ATGATT GCAGCAGAAG ACATTGGCAG GCAGATTCTT ACATACGGAG AGAG---GAA
Bjut_A019081                 GGAAAAG--- TTAATCAGAA GCATCTTGAT CGCGCTAAGG CAGCCACAAA ATCTGCAGTT CTGATGAATT TGGAATCACG G---ATGATT GCAGCAGAAG ACATTGGCAG GCAGATTCTT ACATACGGAG AGAG---GAA
Bjuv_A05_VARUNA_g1802.t1     GGAAAAG--- TTAATCAGAA GCATCTTGAT CGCGCCAAGG CAGCCACAAA ATCTGCAGTT CTGATGAATT TGGAATCACG G---ATGATT GCAGCAGAAG ACATTGGCAG GCAGATTCTT ACATACGGAG AGAG---GAA
Bjuv_B05_VARUNA_g3772.t1     GGAAAAG--- TTAATCAGAA GCATCTTGAC CGCGCCAAGG CAGCCACAAA ATCTGCAGTT CTGATGAATT TGGAATCACG G---ATGATT GCCGCAGAAG ACATTGGCAG GCAGATTCTT ACATACGGAG AGAG---GAA
BniC2_B06g033440.1           GGAAAAG--- TTAATCAGAA GCATCTTGAC CGCGCCAAGG CAGCCACAAA ATCTGCAGTT CTGATGAATT TGGAATCACG G---ATGATT GCCGCAGAAG ACATTGGCAG GCAGATTCTT ACATACGGAA AGAG---GAA
BniN100_B06g028650.2         GGAAAAG--- TTAATCAGAA GCATCTTGAC CGCGCCAAGG CAGCCACAAA ATCTGCAGTT CTGATGAATT TGGAATCACG G---ATGATT GCCGCAGAAG ACATTGGCAG GCAGATTCTT ACATACGGAA AGAG---GAA

                             ....|....| ....|....| ....|....| ....|....| ....|....| ....|....| ....|....| ....|....| ....|....| ....|....| ....|....| ....|....| ....|....| ....|....|
                                   1405       1415       1425       1435       1445       1455       1465       1475       1485       1495       1505       1515       1525       1535
BraCCB_A05p16270.1           ACCAGTTGAG CAATTCCTGA AGACAGTTGA TGGACTAACC TTGAAGGACA TTACAGACTT CACCAGCAAG ATTATCTCAA AGCCTTTGAC AATGGGTTCC TTCGGAGATG ---TGTTGTC TGTTCCTAGC TACGACACCA
BraTUE_A05p17740.1           ACCAGTTGAG CAATTCCTGA AGACAGTTGA TGGACTAACC TTGAAGGACA TTACAGACTT CACCAGCAAG ATTATCTCAA AGCCTTTGAC AATGGGTTCC TTCGGAGATG ---TGTTGTC TGTTCCTAGC TACGACACCA
BraZ1_A05t20826              ACCAGTTGAG CAATTCCTGA AGACAGTTGA TGGACTAACC TTGAAGGACA TTACAGACTT CACCAGCAAG ATTATCTCAA AGCCTTTGAC AATGGGTTCC TTCGGAGATG ---TGTTGTC TGTTCCTAGC TACGACACCA
BraPCA_A05p18220.1           ACCAGTTGAG CAATTCCTGA AGACAGTTGA TGGACTAACC TTGAAGGACA TTACAGATTT CACCAGCAAG ATTATCTCAA AGCCTTTGAC AATGGGTTCC TTCGGAGATG ---TGTTGTC TGTTCCTAGC TACGACACCG
Bjut_A019081                 ACCAGTTGAG CAATTCCTGA AGACAGTTGA TGGACTAACC TTGAAGGACA TTACAGACTT CACCAGCAAG ATTATCTCAA AGCCTTTGAC AATGGGTTCC TTCGGAGATG ---TGTTGTC TGTTCCTAGC TACGACACCA
Bjuv_A05_VARUNA_g1802.t1     ACCAGTTGAG CAATTCCTGA AGACAGTTGA TGGACTAACC TTGAAGGACA TTACAGATTT CACCAGCAAG ATTATCTCAA AGCCTTTGAC AATGGGTTCC TTCGGAGATG ---TGTTGTC TGTTCCTAGC TACGACACCG
Bjuv_B05_VARUNA_g3772.t1     ACCAGTTGAG CAATTCCTGA AGACAGTAGA TGGACTAACC TTGAAGGACA TTACAGATTT CACCAGCAAG ATTATCTCAA AGCCTTTGAC AATGGCTTCC TTCGGAGATG ---TGTTGTC TGTTCCTAGC TACGACACCG
BniC2_B06g033440.1           ACCAGTTGAG CAATTCCTGA AGACAGTAGA TGGACTAACC TTGAAGGACA TTACAGATTT CACCAGCAAG ATTATCTCAA AGCCTTTGAC AATGGCTTCC TTCGGAGATG ---TGTTGTC TGTTCCTAGC TACGACACCG
BniN100_B06g028650.2         ACCAGTTGAG CAATTCCTGA AGACAGTAGA TGGACTAACC TTGAAGGACA TTACAGATTT CACCAGCAAG ATTATCTCAA AGCCTTTGAC AATGGCTTCC TTCGGAGATG ---TGTTGTC TGTTCCTAGC TACGACACCG

                             ....|....| ....|....|
                                   1545       1555
BraCCB_A05p16270.1           TAAGCAGCAA GTTTTCTTGA
BraTUE_A05p17740.1           TAAGCAGCAA GTTTTCTTGA
BraZ1_A05t20826              TAAGCAGCAA GTTTTCTTGA
BraPCA_A05p18220.1           TAAGCAACAA GTTTTCTTGA
Bjut_A019081                 TAAGCAGCAA GTTTTCTTGA
Bjuv_A05_VARUNA_g1802.t1     TAAGCAACAA GTTTTCTTGA
Bjuv_B05_VARUNA_g3772.t1     TAAGCAGCAA GTTTTCTTGA
BniC2_B06g033440.1           TAAGCAGCAA GTTTTCTTGA
BniN100_B06g028650.2         TAAGCAGCAA GTTTTCTTGA
```

(F) Clade IC BBCC

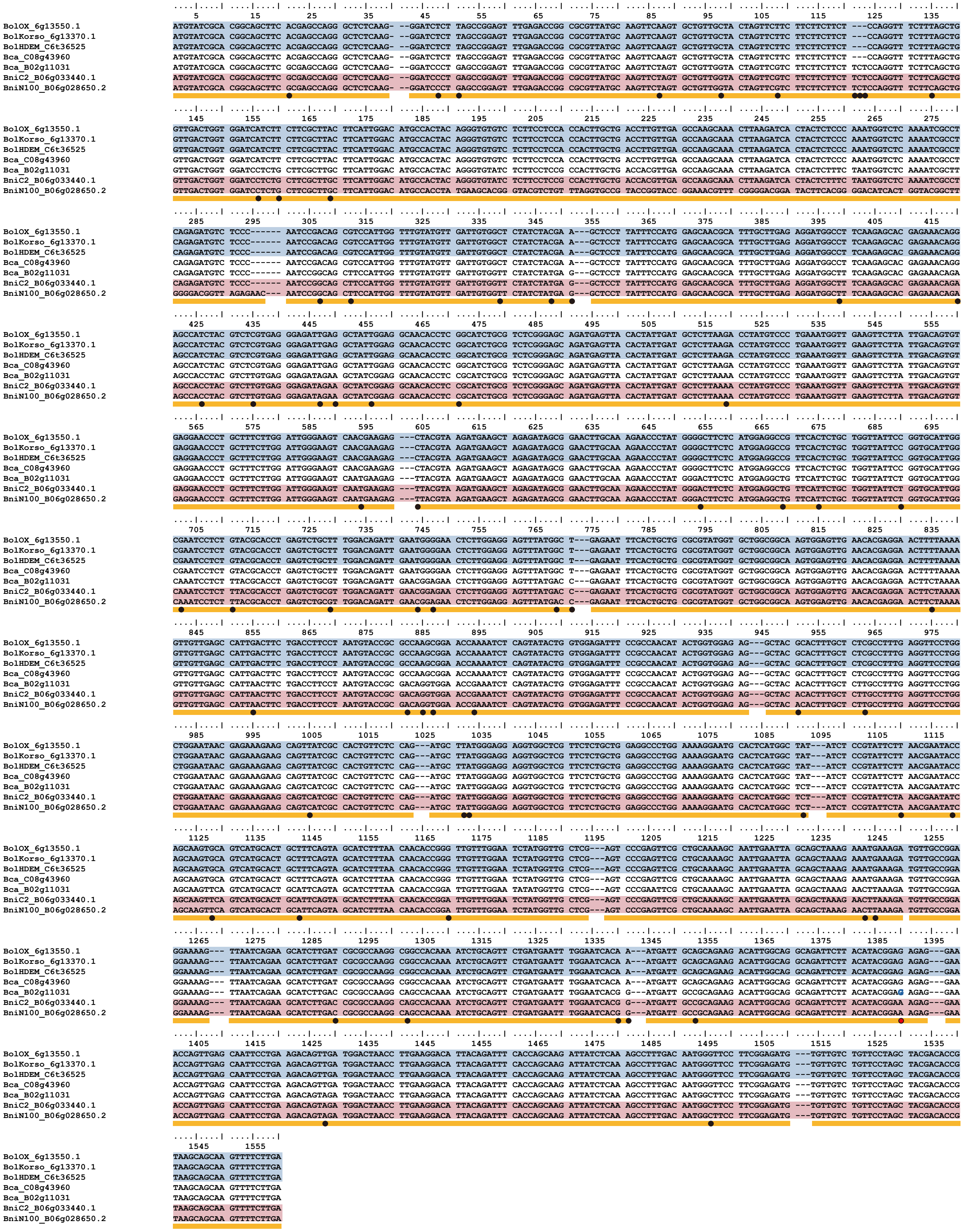

(G) Clade II AABB

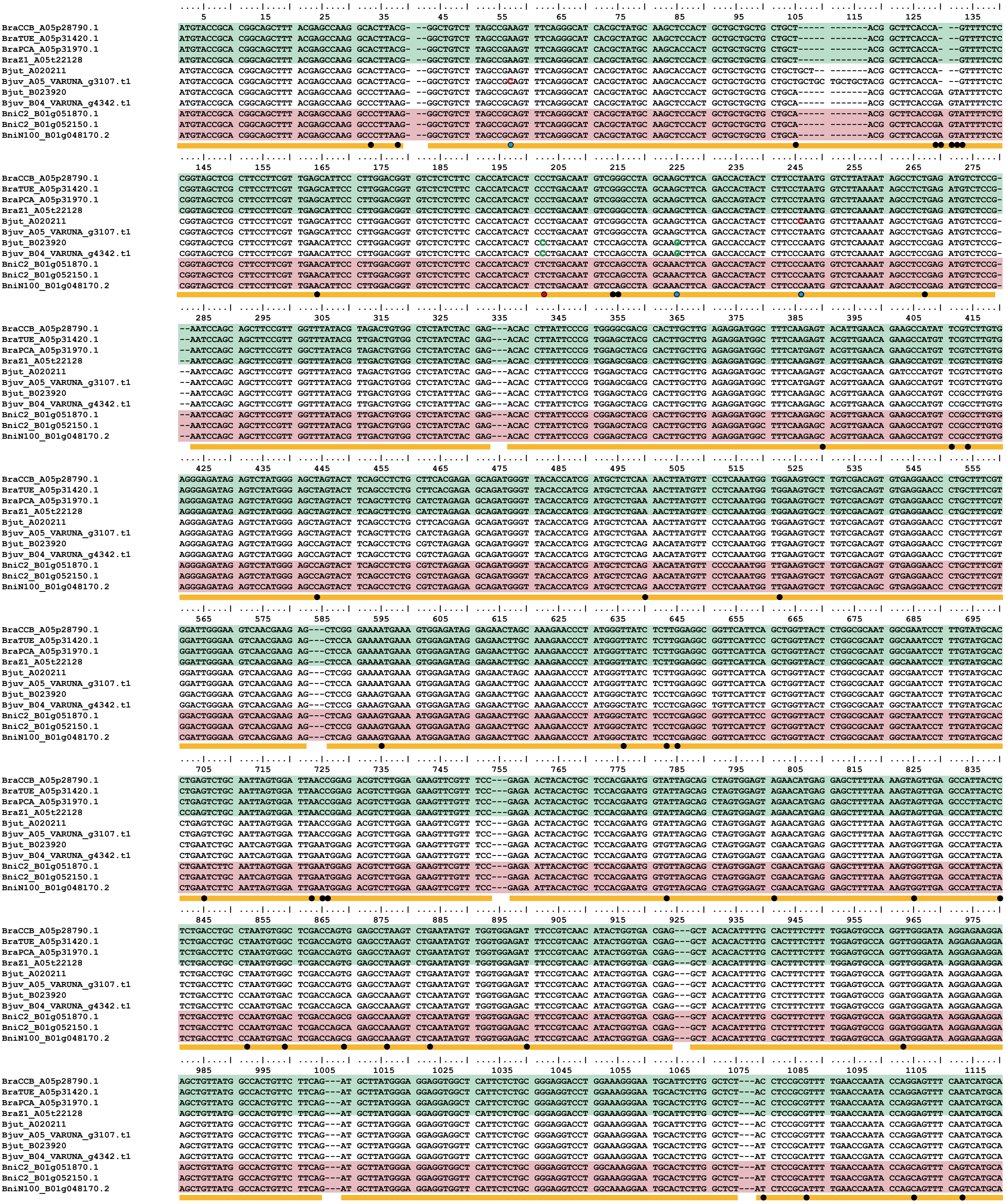

```
                              ....|....| ....|....| ....|....| ....|....| ....|....| ....|....| ....|....| ....|....| ....|....| ....|....| ....|....| ....|....| ....|....| ....|....|
                                   1125       1135       1145       1155       1165       1175       1185       1195       1205       1215       1225       1235       1245       1255
BraCCB_A05p28790.1            CTGCCTTCAC TAGCATCTTT AACAACTCTG GATTGTTTGG AATCTATGGC TGCACG---A GTCCTGAGTA TGCTGCAAAG GGAATTGAAT TAGTAGCTGA TGAAATGAAA GCTGTCGCTG AAGGAAAAG- --TTAACCAG
BraTUE_A05p31420.1            CTGCCTTCAC TAGCATCTTT AACAACTCTG GATTGTTTGG AATCTATGGC TGCACG---A GTCCTGAGTA TGCTGCAAAG GGAATTGAAT TAGTAGCTGA TGAAATGAAA GCTGTCGCTG AAGGAAAAG- --TTAACCAG
BraPCA_A05p31970.1            CTGCCTTCAC TAGCATCTTT AACAACTCTG GATTGTTTGG AATCTATGGC TGCACG---A GTCCTGAGTA TGCTGCAAAG GGAATTGAAT TAGTAGCTGA TGAAATGAAA GCTGTCGCTG AAGGAAAAG- --TTAACCAG
BraZ1_A05t22128               CTGCCTTCAC TAGCATCTTT AACAACTCTG GATTGTTTGG AATCTATGGC TGCACG---A GTCCTGAGTA TGCTGCAAAG GGAATTGAAT TAGTAGCTGA TGAAATGAAA GCTGTCGCTG AAGGAAAAG- --TTAACCAG
Bjut_A020211                  CTGCCTTCAC TAGCATCTTT AACAACTCTG GATTGTTTGG AATCTATGGC TGCACG---A GTCCTGAGTA TGCTGCAAAG GGAATTGAAT TAGTAGCTGA TGAAATGAAA GCTGTCGCTG AAGGAAAAG- --TTAACCAG
Bjuv_A05_VARUNA_g3107.t1      CTGCCTTCAC TAGCATCTTT AACAACTCTG GATTGTTTGG AATCTATGGC TGCACG---A GTCCTGAGTA TGCTGCAAAG GGAATTGAAT TAGTAGCTGA TGAAATGAAA GCTGTCGCTG AAGGAAAAG- --TTAACCAG
Bjut_B023920                  CTGCATTCAC TAGCATCTTT AACAACGCTG GGTTGTTTGG AATCTATGGT TGCACG---A GTCCTGAGTA TGCTGCAAAG GGAATTGAAT TAGTAGCTGA TGAAATGAAA GCTGTCGCTG AAGGAAAAG- --TTAACCAG
Bjuv_B04_VARUNA_g4342.t1      CTGCATTCAC TAGCATCTTT AACAACGCTG GGTTGTTTGG AATCTATGGT TGCACG---A GTCCTGAGTA TGCTGCAAAG GGAATTGAAT TAGTAGCTGA TGAAATGAAA GCTGTCGCTG AAGGAAAAG- --TTAACCAG
BniC2_B01g051870.1            CTGCATTCAC TAGCATCTTT AACAACGCTG GATTGTTTGG AATCTATGGT TGCACG---A GTCCTGAGTA TGCTGCAAAG GGAATTGAAT TAGTAGCTGA TGAAATGAAA GCTGTCGCTG AAGGAAAAG- --TTAACCAG
BniC2_B01g052150.1            CTGCATTCAC TAGCATCTTT AACAACGCTG GGTTGTTTGG AATCTATGGT TGCACG---A GTCCTGAGTA TGCTGCAAAG GGAATTGAAT TAGTAGCTGA TGAAATGAAA GCTGTCGCTG AAGGAAAAG- --TTAACCAG
BniN100_B01g048170.2          CTGCATTCAC TAGCATCTTT AACAACGCTG GGTTGTTTGG AATCTATGGT TGCACG---A GTCCTGAGTA TGCTGCAAAG GGAATTGAAT TAGTAGCTGA TGAAATGAAA GCTGTCGCTG AAGGCAAAG- --TTAACCAG

                              ....|....| ....|....| ....|....| ....|....| ....|....| ....|....| ....|....| ....|....| ....|....| ....|....| ....|....| ....|....| ....|....| ....|....|
                                   1265       1275       1285       1295       1305       1315       1325       1335       1345       1355       1365       1375       1385       1395
BraCCB_A05p28790.1            AAACATCTTG ATCGAGCCAA GGCTGCTACT AAATCTGCAA TTCTAATGAA TCTTGAATCT AGG---ATGA TTGTAGCAGA AGACATTGGT AGACAGATAC TTACATACGG GGAGAG---G AAGCCAGTGG ATGCATTCTT
BraTUE_A05p31420.1            AAACATCTTG ATCGAGCTAA GGCTGCTACT AAATCTGCAA TTCTAATGAA TCTTGAATCT AGG---ATGA TTGTAGCAGA AGACATTGGT AGACAGATAC TTACATACGG GGAGAG---G AAGCCAGTGG ATGCATTCTT
BraPCA_A05p31970.1            AAACATCTTG ATCGAGCCAA GGCTGCTACT AAATCTGCAA TTCTAATGAA TCTTGAATCT AGG---ATGA TTGTAGCAGA AGACATTGGT AGACAGATAC TTACATACGG GGAGAG---G AAGCCAGTGG ATGCATTCTT
BraZ1_A05t22128               AAACATCTTG ATCGAGCTAA GGCTGCTACT AAATCTGCAA TTCTAATGAA TCTTGAATCT AGG---ATGA TTGTAGCAGA AGACATTGGT AGACAGATAC TTACATACGG GGAGAG---G AAGCCAGTGG ATGCATTCTT
Bjut_A020211                  AAACATCTTG ATCGAGCTAA GGCTGCTACT AAATCTGCAA TTCTAATGAA TCTTGAATCT AGG---ATGA TTGTAGCAGA AGACATTGGT AGACAGATAC TTACATACGG GGAGAG---G AAGCCAGTGG ATGCATTCTT
Bjuv_A05_VARUNA_g3107.t1      AAACATCTTG ATCGAGCCAA GGCTGCTACT AAATCTGCAA TTCTAATGAA TCTTGAATCT AGG---ATGA TTGTAGCAGA AGACATTGGT AGACAGATAC TTACATACGG GGAGAG---G AAGCCAGTGG ATGCATTCTT
Bjut_B023920                  AAACATCTTG ATCGAGCCAA GGCAGCAACT AAATCTGCAA TTCTGATGAA TCTTGAATCT AGG---ATGA TTGTAGCAGA AGACATCGGT AGACAGATAC TTACATACGG AGAAAG---G AAGCCAGTGG ATGCATTCTT
Bjuv_B04_VARUNA_g4342.t1      AAACATCTTG ATCGAGCCAA GGCAGCAACT AAATCTGCAA TTCTGATGAA TCTTGAATCT AGG---ATGA TTGTAGCAGA AGACATCGGT AGACAGATAC TTACATACGG AGAAAG---G AAGCCAGTGG ATGCATTCTT
BniC2_B01g051870.1            AAACATCTTG ATCGAGCCAA GGCAGCAACT AAATCTGCAA TTCTGATGAA TCTTGAATCT AGG---ATGA TTGTAGCAGA AGACATCGGT AGACAGATAC TTACATACGG AGAAAG---G AAGCCAGTGG ATGCATTCTT
BniC2_B01g052150.1            AAACATCTTG ATCGAGCCAA GGCAGCAACT AAATCTGCAA TTCTGATGAA TCTTGAATCT AGG---ATGA TTGTAGCAGA AGACATCGGT AGACAGATAC TTACATACGG AGAAAG---G AAGCCAGTGG ATGCATTCTT
BniN100_B01g048170.2          AAACATCTTG ATCGAGCCAA GGCAGCAACT AAATCTGCAA TTCTGATGAA TCTTGAATCT AGG---ATGA TTGTAGCAGA AGACATCGGT AGACAGATAC TTACATACGG AGAAAG---G AAGCCAGTGG ATGCATTCTT

                              ....|....| ....|....| ....|....| ....|....| ....|....| ....|....| ....|....| ....|....| ....|....| ....|....| ....|....| ....|....| ....|....| ....|....|
                                   1405       1415       1425       1435       1445       1455       1465       1475       1485       1495       1505       1515       1525       1535
BraCCB_A05p28790.1            AAAGACAGTG GACCAACTCA CCTTAAAGGA CATTGCAGAT TTCACCAGCA AGATTATTGC AAAACCTTTG ACGATGGGAT CTTTAGGAGC AG---TGTTG AATGTTCCAA GCTACGACTC CGTAAGCAGT AGATTTTCTT
BraTUE_A05p31420.1            AAAGACAGTG GACCAACTCA CCTTAAAGGA CATTGCAGAT TTCACCAGCA AGATTATTGC AAAACCTTTG ACGATGGGAT CTTTAGGAGC AG---TGTTG AATGTTCCAA GCTACGACTC CGTAAGCAGT AGATTTTCTT
BraPCA_A05p31970.1            AAAGACAGTG GACCAACTCA CCTTAAAGGA CATTGCAGAT TTCACCAGCA AGATTATTGC AAAACCTTTG ACGATGGGAT CTTTAGGAGC AG---TGTTG AATGTTCCAA GCTACGACTC CGTAAGCAGT AGATTTTCTT
BraZ1_A05t22128               AAAGACAGTG GACCAACTCA CCTTAAAGGA CATTGCAGAT TTCACCAGCA AGATTATTGC AAAACCTTTG ACGATGGGAT CTTTAGGAGC AG---TGTTG AATGTTCCAA GCTACGACTC CGTAAGCAGT AGATTTTCTT
Bjut_A020211                  AAAGACAGTG GACCAACTCA CCTTAAAGGA CATTGCAGAT TTCACCAGCA AGATTATTGC AAAACCTTTG ACGATGGGAT CTTTAGGAGC AG---TGTTG AATGTTCCAA GCTACGACTC CGTAAGCAGT AGATTTTCTT
Bjuv_A05_VARUNA_g3107.t1      AAAGACAGTG GACCAACTCA CCTTAAAGGA CATTGCAGAT TTCACCAGCA AGATTATTGC AAAACCTTTG ACGATGGGAT CTTTAGGAGC AG---TGTTG AATGTTCCAA GCTACGACTC CGTAAGCAGT AGATTTTCTT
Bjut_B023920                  GAAGACAGTT GACCAACTCA CCTTAAAGGA CATAGCAGAT TTCACCAGCA AGATTATTGC AAAGCCTTTG ACCATGGCAT CTTTAGGAGC AG---TGTTG AATGTTCCAA GCTACGACTC CGTAAGCAGT AGATTTTCTT
Bjuv_B04_VARUNA_g4342.t1      GAAGACAGTT GACCAACTCA CCTTAAAGGA CATAGCAGAT TTCACCAGCA AGATTATTGC AAAGCCTTTG ACCATGGCAT CTTTAGGAGC AG---TGTTG AATGTTCCAA GCTACGACTC CGTAAGCAGT AGATTTTCTT
BniC2_B01g051870.1            GAAGACAGTT GACCAACTCA CCTTAAAGGA CATAGCAGAT TTCACCAGCA AGATTATTGC AAAGCCTTTG ACCATGGCAT CTTTAGGAGC AG---TGTTG AATGTTCCAA GCTACGACTC CATAAGCAGT AGATTTTCTT
BniC2_B01g052150.1            GAAGACAGTT GACCAACTCA CCTTAAAGGA CATAGCAGAT TTCACCAGCA AGATTATTGC AAAGCCTTTG ACCATGGCAT CTTTAGGAGC AG---TGTTG AATGTTCCAA GCTACGACTC CGTAAGCAGT AGATTTTCTT
BniN100_B01g048170.2          GAAGACAGTT GACCAACTCA CCTTAAAGGA CATAGCAGAT TTCACCAGCA AGATTATTGC AAAGCCTTTG ACCATGGCAT CTTTAGGAGC AG---TGTTG AATGTTCCAA CCTACGACTC CGTAAGCAGT AGATTTTCTT

                              ..
BraCCB_A05p28790.1            GA
BraTUE_A05p31420.1            GA
BraPCA_A05p31970.1            GA
BraZ1_A05t22128               GA
Bjut_A020211                  GA
Bjuv_A05_VARUNA_g3107.t1      GA
Bjut_B023920                  GA
Bjuv_B04_VARUNA_g4342.t1      GA
BniC2_B01g051870.1            GA
BniC2_B01g052150.1            GA
BniN100_B01g048170.2          GA
```

(H) Clade II BBCC

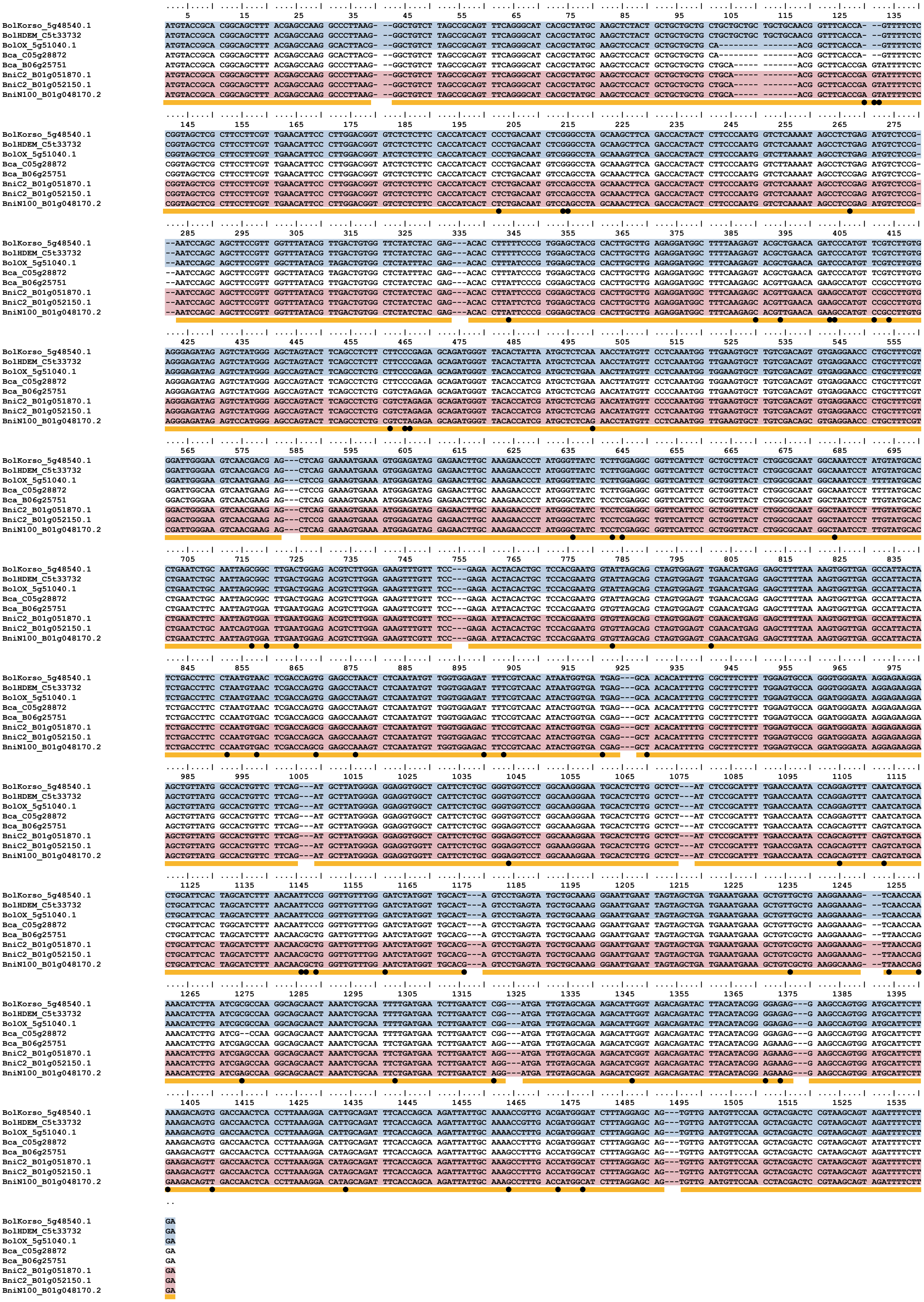

Supplementary Fig S10. Alignment of coding region of genes encoded MPP-α subunit of the mitochondrial complex III in studied genomes/subgenomes. The shade in green indicates *B. rapa* (AA), red indicates *B. nigra* (BB), and blue indicates *B. oleracea* (CC). The black dot indicates genome-specific site, blue dot indicates synonymous inter-genomic conversion, red dot indicates non-synonymous inter-genomic conversion, and grey dot indicates autapomorphy.
