# Supplementary material for: Cytonuclear Interactions and Subgenome Dominance Shape the Evolution of Organelle-Targeted Genes in the Brassica Triangle of U
Source: Mol Biol Evol. 2024 Feb 23;41(3):msae043. doi: 10.1093/molbev/msae043 (PMC10919925; doi:10.1093/molbev/msae043)
Supplement: msae043_Supplementary_Data [file msae043_supplementary_data.zip › Supplementary Figure S11.pdf]

(A) Clade I AABB

```
                         5         15         25         35         45         55         65         75         85         95        105        115        125        135
BraTUE_A05p41820.1       ATGGCGATGA AGAACCTATT GACCTCCGCC CTCCGATCTC AGAGGCGTCT CGCCCTGAAC CGAGCCGCAC GAGCTTTGTC CTCCGTTTCA GCTCTCGATT CGGCTTCTCT GACGTCTCAC TCCCCCTCCC CCACGCCGCC
BraPCA_A05p42570.1       ATGGCGATGA AGAACCTATT GACCTCCGCC CTCCGATCTC AGAGGCGTCT CGCCCTGAAC CGAGCCGCAC GAGCTTTGTC CTCCGTTTCA GCTCTCGATT CGGCTTCTCT GACGTCTCAC TCCCCCTCCC CCACGCCGCC
BraCCB_A05p40150.1       ATGGCGATGA AGAACCTATT GACCTCCGCC CTCCGATCTC AGAGGCGTCT CGCCCTGAAC CGAGCCGCAC GAGCTTTGTC CTCCGTTTCA GCTCTCGATT CGGCTTCTCT GACGTCTCAC TCCCCCTCCC CCACGCCGCC
BraZ1_A05t23166          ATGGCGATGA AGAACCTATT GACCTCCGCC CTCCGATCTC AGAGGCGTCT CGCCCTGAAC CGAGCCGCAC GAGCTTTGTC CTCCGTTTCA GCTCTCGACT CGGCTTCTCT GACGTCTCAC TCCCCCTCCC CCACGCCGCC
Bjuv_A05_VARUNA_g4271.t1 ATGGCGATGA AGAACCTATT GACCTCCGCC CTCCGATCTC AGAGGCGTCT CGCCCTTAAC CGAGCCGCAC GAGCTTTGTC CTCCGTTTCA GCTCTCGACT CGGCTTCTCT GACGTCTCAC TCCCCCTCCC CCACGCCGCC
Bjut_A021166             ATGGCGATGA AGAACCTATT GACCTCCGCC CTCCGATCTC AGAGGCGTCT CGCCCTGAAC CGAGCCGCAC GAGCTTTGTC CTCCGTTTCA GCTCTCGATT CGGCTTCTCT GACGTCTCAC TCCCCCTCCC CCACGCCGCC
Bjut_0011533             ATGGCGATGA AGAATCTATT GACCGCAGCT CTCCGATCTC AGAGGCGTCT GGCCCTGAAT CGAGCCGTAA GAGCTTCCTC CTCCCTTTCA GCG------- -----TCTCT GGCGTCTCCC TCCCCTACGA CCACGCCGCC
Bjuv_B04_VARUNA_g5554.t1 ATGGCGATGA AGAATCTATT GACCGCAGCT CTCCGATCTC AGAGGCGTCT GGCCCTGAAT CGAGCCGTAA GAGCTTCCTC CTCCCTTTCA GCG------- -----TCTCT GGCGTCTCCC TCCCCTACGA CCACGCCGCC
BniC2_B01g064900.1_pseudo ATGGCGATGA AGAATCTATT GACCGCAGCT CTCCGATCTC AGAGGCGTCT GGCCCTGAAT CGAGCCGTAC GAGCTTCCTC CTCCCTTTCA GCG------- -----TCTCT GGCGTCTCCC TCCCCTACGA CCACGCCGCC
BniN100_B01g060920.2     ATGGCGATGA AGAATCTATT GACCGCAGCT CTCCGATCTC AGAGGCGTCT GGCCCTGAAT CGAGCCGTAC GAGCTTCCTC CTCCCTTTCA GCG------- -----TCTCT GGCGTCTCCC TCCCCTACGA CCACGCCGCC

                       145        155        165        175        185        195        205        215        225        235        245        255        265        275
BraTUE_A05p41820.1       GATTCTCATG CCCTACGACC ACTCCGCCGA GATCGTCAAA GAGAAGCTCA AGAGGCTAGA GAACCCAGAT CAGAGATTCC TCAAATACGC ATCTCCGCAT CCAATCCTCG CCTCACACAA CCACATCTTG TCATCCCCCG
BraPCA_A05p42570.1       GTTTCTCATG CCCTACGACC ACTCCGCCGA GATCGTCAAA GAGAAGCTCA AGAGGCTAGA GAACCCAGAT CAGAGATTCC TCAAATACGC ATCTCCGCAT CCAATCCTCG CCTCACACAA CCACATCTTG TCATCCCCCG
BraCCB_A05p40150.1       GATTCTCATG CCCTACGACC ACTCCGCCGA GATCGTCAAA GAGAAGCTCA AGAGGCTGGA GAACCCAGAT CAGAGATTCC TCAAATACGC ATCTCCGCAT CCAATCCTCG CCTCACACAA CCACATCTTG TCATCCCCCG
BraZ1_A05t23166          GATTCTCATG CCCTACGACC ACTCCGCCGA GATCGTCAAA GAGAAGCTCA AGAGGCTAGA GAACCCAGAT CAGAGATTCC TCAAATACGC ATCTCCGCAT CCAATCCTCG CCTCACACAA CCACATCTTG TCATCCCCCG
Bjuv_A05_VARUNA_g4271.t1 GTTTCTCATG CCCTACGACC ACTCCGCCGA GATCGTCAAA GAGAAGCTCA AGAGGCTGGA GAATCCAGAT CAGAGATTCC TCAAATACGC ATCTCCGCAT CCAATCCTCG CCTCACACAA CCACATCTTG TCATCCCCCG
Bjut_A021166             GATTCTCATG CCCTACGACC ACTCCGCCGA GATCGTCAAA GAGAAGCTCA AGAGGCTGGA GAACCCAGAT CAGAGATTCC TCAAATACGC ATCTCCGCAT CCAATCCTCG CCTCCCACAA CCACATCTTA TCAGCTCCCG
Bjut_0011533             GATCGTCATG CCCTACGACC ACGCCGCCGA GATCACGAAA GAGAAGCTCA AGAGGCTGGA GAACCCAGAT CAGCGATTCC TCAAATACGC ATCTCCTCAT CCGGTCCTCG CCTCCCACAA CCACATCCTC TCAGCCCCGG
Bjuv_B04_VARUNA_g5554.t1 GATCGTCATG CCCTACGACC ACGCCGCCGA GATCACGAAA GAGAAGCTCA AGAGGCTGGA GAACCCAGAT CAGCGATTCC TCAAATACGC ATCTCCTCAT CCGGTCCTCG CCTCCCACAA CCACATCCTC TCAGCCCCGG
BniC2_B01g064900.1_pseudo GATCGTCATG CCCTACGACC ACGCCGCCGA GATCACGAAA GAGAAGCTCA AGAGGCTGGA GAACCCAGAT CAGCGATTCC TCAAATACGC ATCTCCTCAT CCGGTCCTCG CCTCCCACAA CCACATCCTC TCAGCCCCGG
BniN100_B01g060920.2     GATCGTCATG CCCTACGACC ACGCCGCCGA GATCACGAAA GAGAAGCTCA AGAGGCTGGA GAACCCAGAT CAGCGATTCC TCAAATACGC ATCTCCTCAT CCGGTCCTCG CCTCCCACAA CCACATCCTC TCAGCCCCGG

                       285        295        305        315        325        335        345        355        365        375        385        395        405        415
BraTUE_A05p41820.1       AGACGCGCGT CACCACTTTA CCCAACGGCC TCCGAGTCGC CACCGAATCG AATCTCTCCG CGAAGACCGC AACCGTCGGG GTATGGATCG ACGCTGGATC GAGGTTCGAG TCGGACGAGA CGAACGGGAC GGCTCATTTT
BraPCA_A05p42570.1       AGACGCGCGT CACCACTTTA CCCAACGGCC TCCGAGTCGC CACCGAATCG AATCTCTCCG CGAAGACCGC AACCGTCGGG GTATGGATCG ACGCTGGATC GAGGTTCGAG TCGGACGAGA CGAACGGGAC GGCTCATTTT
BraCCB_A05p40150.1       AGACGCGCGT CACCACTTTA CCCAACGGCC TCCGAGTCGC CACCGAATCG AATCTCTCCG CGAAGACCGC AACCGTCGGG GTATGGATCG ACGCTGGATC GAGGTTCGAG TCGGACGAGA CGAACGGGAC GGCTCATTTT
BraZ1_A05t23166          AGACGCGCGT CACCACTTTA CCCAACGGCC TCCGAGTCGC CACCGAATCG AATCTCTCCG CGAAGACCGC AACCGTCGGG GTATGGATCG ACGCTGGATC GAGGTTCGAG TCGGACGAGA CGAACGGGAC GGCTCATTTT
Bjuv_A05_VARUNA_g4271.t1 AGACGCGCGT CACCACTTTA CCCAACGGCC TCCGAGTCGC CACCGAATCG AATCTCTCCG CGAAGACCGC AACCGTCGGG GTATGGATCG ACGCTGGATC GAGGTTCGAG TCGGACGAGA CGAACGGGAC GGCTCATTTT
Bjut_A021166             AGACGCGCGT CACCACTTTA CCCAACGGCC TCCGAGTCGC CACCGAATCG AATCTCTCCG CGAAGACCGC AACCGTCGGG GTATGGATCG ACGCTGGATC GAGGTTCGAG TCGGACGAGA CGAACGGGAC GGCTCATTTT
Bjut_0011533             AGACTCGCGT CACCACCCTG CCCAACGGTC TGCGAGTCGC CACCGAATCC AATCTCTCGG CCAAGACGGC CACCGTCGGA GTCTGGATCG ACGCTGGATC CAGGTTCGAA TCCGACGAGA CGAACGGGAC GGCTCATTTC
Bjuv_B04_VARUNA_g5554.t1 AGACTCGCGT CACCACCCTG CCCAACGGTC TGCGAGTCGC CACCGAATCC AATCTCTCGG CCAAGACGGC CACCGTCGGA GTCTGGATCG ACGCTGGATC CAGGTTCGAA TCCGACGAGA CGAACGGGAC GGCTCATTTC
BniC2_B01g064900.1_pseudo AGACCCGCGT CACCACCCTG CCCAACGGTC TGCGAGTCGC CACCGAATCC AATCTCTCCG CGAAGACGGC CACCGTTGGA GTCTGGATCG ACGCTGGATC CAGGTTCGAA TCTGACGAGA CGAACGGGAC GGCTCATTTC
BniN100_B01g060920.2     AGACCCGCGT CACCACCCTG CCCAACGGTC TGCGAGTCGC CACCGAATCC AATCTCTCCG CGAAGACGGC CACCGTTGGA GTCTGGATCG ACGCTGGATC CAGGTTCGAA TCTGACGAGA CGAACGGGAC GGCTCATTTC

                       425        435        445        455        465        475        485        495        505        515        525        535        545        555
BraTUE_A05p41820.1       CTGGAGCATA TGATATTCAA AGGCACGGAG AGGCGTACGG TGAGGGCGTT GGAGGAGGAG ATCGAGGACA TTGGTGGTCA TTTGAATGCG TATACGTCGA GGGAGCAGAC TACTTACTAT GCCAAGGTGA TGGATTCGGA
BraPCA_A05p42570.1       CTGGAGCATA TGATATTCAA AGGCACGGAG AGGCGTACGG TGAGGGCGTT GGAGGAGGAG ATCGAGGACA TTGGTGGTCA TTTGAATGCG TATACGTCGA GGGAGCAGAC TACTTACTAT GCCAAGGTGA TGGATTCGGA
BraCCB_A05p40150.1       CTGGAGCATA TGATATTCAA AGGCACGGAG AGGCGTACGG TGAGGGCGTT GGAGGAGGAG ATCGAGGACA TTGGTGGTCA TTTGAATGCG TATACGTCGA GGGAGCAGAC TACTTACTAT GCCAAGGTGA TGGATTCGGA
BraZ1_A05t23166          CTGGAGCATA TGATATTCAA AGGCACGGAG AGGCGTACGG TGAGGGCGTT GGAGGAGGAG ATCGAGGACA TTGGTGGTCA TTTGAATGCG TATACGTCGA GGGAGCAGAC CACTTACTAT GCCAAGGTGA TGGATTCGGA
Bjuv_A05_VARUNA_g4271.t1 CTGGAGCATA TGATATTCAA AGGCACGGAG AGGCGTACGG TGAGGGCGTT GGAGGAGGAG ATCGAGGACA TTGGTGGTCA TTTGAATGCG TATACGTCGA GGGAGCAGAC TACTTACTAT GCCAAGGTGA TGGATTCGGA
Bjut_A021166             CTGGAGCATA TGATATTCAA AGGCACGGAG AGGCGTACGG TGAGGGCGTT GGAGGAGGAG ATCGAGGACA TTGGTGGTCA TTTGAATGCG TATACGTCGA GGGAGCAGAC CACTTACTAT GCCAAGGTGA TGGATTCGGA
Bjut_0011533             TTGGAGCATA TGATATTCAA AGGCACGGAG AGGCGTACGG TGAGAGCGTT GGAGGAAGAG ATCGAGGACA TTGGTGGTCA TTTGAATGCG TATACGTCGA GGGAGCAGAC CACTTACTAT GCAAAGGTGA TGGAGGCCGA
Bjuv_B04_VARUNA_g5554.t1 TTGGAGCATA TGATATTCAA AGGCACGGAG AGGCGTACGG TGAGAGCGTT GGAGGAAGAG ATCGAGGACA TTGGTGGTCA TTTGAATGCG TATACGTCGA GGGAGCAGAC CACTTACTAT GCAAAGGTGA TGGAGGCCGA
BniC2_B01g064900.1_pseudo TTGGAGCATA TGATATTCAA AGGCACGGAG AGGCGTACGG TGAGAGCGTT GGAGGAAGAG ATCGAGGACA TTGGTGGTCA TTTGAATGCG TATACGTCGA GGGAGCAGAC CACTTACTAT GCCAAGGTGA TGGAGTCCGA
BniN100_B01g060920.2     TTGGAGCATA TGATATTCAA AGGCACGGAG AGGCGTACGG TGAGAGCGTT GGAGGAAGAG ATCGAGGACA TTGGTGGTCA TCTCAATGCG TATACGTCGA GGGAGCAGAC CACTTACTAT GCCAAGGTGA TGGAGTCCGA

                       565        575        585        595        605        615        625        635        645        655        665        675        685        695
BraTUE_A05p41820.1       TGTGAACCAG GCGTTGGATG TGTTGGCTGA TATCTTGCAG AACTCTAAGT TCGAGGAGCA GAGGATTAAC CGGGAGCGTG ATGTCATCCT CAGGGAAATG CAAGAG---G TGGAGGGACA GACCGATGAA GTTGTTCTTG
BraPCA_A05p42570.1       TGTGAACCAG GCGTTGGATG TGTTGGCTGA TATCTTGCAG AACTCTAAGT TCGAGGAGCA GAGGATTAAC CGGGAGCGTG ATGTCATCCT CAGGGAAATG CAAGAG---G TGGAGGGACA GACCGATGAA GTTGTTCTTG
BraCCB_A05p40150.1       TGTGAACCAG GCGTTGGATG TGTTGGCTGA TATCTTGCAG AACTCTAAGT TCGAGGAGCA GAGGATTAAC CGGGAGCGTG ATGTCATCCT CAGGGAAATG CAAGAG---G TGGAGGGACA AACTGATGAA GTTGTTCTTG
BraZ1_A05t23166          TGTGAACCAG GCGTTGGATG TGTTGGCTGA TATCTTGCAG AACTCTAAGT TCGAGGAGCA GAGGATTAAC CGGGAGCGTG ATGTCATCCT CAGGGAAATG CAAGAG---G TGGAGGGACA AACTGATGAA GTTGTGCTTG
Bjuv_A05_VARUNA_g4271.t1 TGTGAACCAG GCGTTGGATG TGTTGGCTGA TATCTTGCAG AACTCTAAGT TCGAGGAGCA GAGGATTAAC CGGGAGCGTG ATGTCATCCT CAGGGAAATG CAAGAG---G TGGAGGGACA GACCGATGAA GTTGTTCTTG
Bjut_A021166             TGTGAACCAG GCGTTGGATG TGTTGGCTGA TATCTTGCAG AACTCTAAGT TCGAGGAGCA GAGGATTAAC CGGGAGCGTG ATGTCATCCT CAGGGAAATG CAAGAG---G TGGAGGGACA AACTGATGAA GTTGTGCTTG
Bjut_0011533             TGTGAACCAG GCTTTGGATG TCTTGGCTGA TATCTTGCAG AACTCCAAGT TCGAGGAGCA GAGGATTAAC CGTGAGCGTG ATGTCATCCT CAGGGAAATG CAAGAG---G TGGAGGGACA AACCGATGAA GTTGTTCTTG
Bjuv_B04_VARUNA_g5554.t1 TGTGAACCAG GCTTTGGATG TCTTGGCTGA TATCTTGCAG AACTCCAAGT TCGAGGAGCA GAGGATTAAC CGTGAGCGTG ATGTCATCCT CAGGGAAATG CAAGAG---G TGGAGGGACA AACCGATGAA GTTGTTCTTG
BniC2_B01g064900.1_pseudo TGTGAACCAG GCTTTGGATG TCTTGGCTGA TATCTTGCAG AACTCCAAGT TCGAGGAGCA GAGGATTAAC CGTGAGCGTG ATGTCATCCT CAGGGAAATG CAAGAG---G TGGAGGGACA AACCGATGAA GTTGTTCTTG
BniN100_B01g060920.2     TGTGAACCAG GCTTTGGATG TCTTGGCTGA TATCTTGCAG AACTCCAAGT TCGAGGAGCA GAGGATTAAC CGTGAGCGTG ATGTCATCCT CAGGGAAATG CAAGAG---G TGGAGGGACA AACCGATGAA GTTGTTCTTG

                       705        715        725        735        745        755        765        775        785        795        805        815        825        835
BraTUE_A05p41820.1       ACCATCTACA TGCCACTGCT TTCCAATACA CACCTCTTGG AAGAACTATT CTTGGACCTG CTCAGAATAT CAAGTCTATC ACCAGAAAGG ATCTTCAGAA CTACATCAAG ACTCACTACA CAGCTTCCAG GATG------
BraPCA_A05p42570.1       ACCATCTACA TGCCACTGCT TTCCAATACA CACCTCTTGG AAGAACTATT CTTGGACCTG CTCAGAATAT CAAGTCTATC ACCAGAAATG ATCTTCAGAA CTACATCAAG ACTCACTACA CAGCTTCCAG GATG------
BraCCB_A05p40150.1       ACCATCTACA TGCCACTGCT TTCCAATACA CACCTCTTGG AAGAACTATT CTTGGACCTG CTCAGAATAT CAAGTCTATC ACCAGAAATG ATCTTCAGAA CTACATCAAG ACTCACTACA CAGCTTCCAG GATG------
BraZ1_A05t23166          ACCATCTACA TGCCACTGCT TTCCAATACA CACCTCTTGG AAGAACTATT CTTGGACCTG CTCAGAATAT CAAGTCTATC ACCAGAAATG ATCTTCAGAA CTACATCAAG ACTCACTACA CAGCTTCCAG GATG------
Bjuv_A05_VARUNA_g4271.t1 ACCATCTACA TGCCACTGCT TTCCAATACA CACCTCTTGG AAGAACTATT CTTGGACCTG CTCAGAATAT CAAGTCTATC ACCAGAAAGG ATCTTCAGAA CTACATCAAG ACTCACTACA CAGCTTCCAG GATG------
Bjut_A021166             ACCATCTACA TGCCACTGCT TTCCAATACA CACCTCTTGG AAGAACTATT CTTGGACCTG CTCAGAATAT CAAGTCTATC ACCAGAAATG ATCTTCAGAA CTACATCAAG ACTCACTACA CAGCTTCCAG GATG------
Bjut_0011533             ACCATTTACA TGCCACTGCA TTCCAGTACA CACCTCTTGG AAGAACTATT CTTGGACCTG CTCAGAATAT CAAGTCCATC ACCAGAAATG ATCTTCAGAA TTACATTAAG ACTCACTACA CTGCCTCCAG GATG------
Bjuv_B04_VARUNA_g5554.t1 ACCATTTACA TGCCACTGCA TTCCAGTACA CACCTCTTGG AAGAACTATT CTTGGACCTG CTCAGAATAT CAAGTCCATC ACCAGAAATG ATCTTCAGAA TTACATTAAG ACTCACTACA CTGCCTCCAG GATG------
BniC2_B01g064900.1_pseudo ACCATTTACA TGCCACTGCA TTCCAGTACA CACCTCTTGG AAGAACTATT CTTGGACCTG CTCAGAATAT CAAGTCTATC ACCAGAAATG ATCTTCAGAA TTACATTAAG ACTCACTACA CTGCCTTCCA GGATG-----
BniN100_B01g060920.2     ACCATTTACA TGCCACTGCA TTCCAGTACA CACCTCTTGG AAGAACTATT CTTGGACCTG CTCAGAATAT CAAGTCTATC ACCAGAAATG ATCTTCAGAA CTACATCAAG ACTCACTACA CAGCTTCCAG GATG------

                       845        855        865        875        885        895        905        915        925        935        945        955        965        975
BraTUE_A05p41820.1       GTGATTGCTG CGGCAGGAGC TGTCAAGCAT GAGGAAGTTG TTGAGCAAGT GAAGAAGCTA TTTAACAAGT TGTCATCTGA TCCGACTACT ACTACTCAAC TAGTTGCCAA AGAACCTGCT AGTTTTACTG GCTCTGAG--
BraPCA_A05p42570.1       GTGATTGCTG CGGCAGGAGC TGTCAAGCAT GAGGAAGTTG TTGAGCAAGT GAAGAAGCTA TTTAACAAGT TGTCATCTGA TCCGACTACT ACTACTCAAC TAGTTGCCAA AGAACCTGCT AGTTTTACTG GCTCTGAG--
BraCCB_A05p40150.1       GTGATTGCTG CGGCAGGAGC TGTCAAGCAT GAGGAAGTTG TTGAGCAAGT GAAGAAGCTA TTTAACAAGT TGTCATCTGA TCCGACTACT ACTACTCAAC TAGTTGCCAA AGAACCTGCT AGTTTTACTG GCTCTGAG--
BraZ1_A05t23166          GTGATTGCTG CGGCAGGAGC TGTCAAGCAT GAGGAAGTTG TTGAGCAAGT GAAGAAGCTA TTTAACAAGT TGTCATCTGA TCCGACTACT ACTACTCAAC TAGTTGCCAA AGAACCTGCT AGTTTTACTG GCTCTGAG--
Bjuv_A05_VARUNA_g4271.t1 GTGATTGCTG CGGCAGGAGC TGTCAAGCAT GAGGAAGTTG TTGAGCAAGT GAAGAAGCTA TTTAACAAGT TGTCATCTGA TCCGACTACT ACTACTCAAC TAGTTGCCAA AGAACCTGCT AGTTTTACTG GCTCTGAG--
Bjut_A021166             GTGATTGCTG CGGCAGGAGC TGTCAAGCAT GAGGAAGTTG TTGAGCAAGT GAAGAAGCTA TTTAACAAGT TGTCATCTGA TCCGACTACT ACTACTCAAC TAGTTGCCAA AGAACCTGCT AGTTTTACTG GCTCTGAG--
Bjut_0011533             GTGATTGCTG CGGCAGGAGC TGTGAAGCAT GAGGAAGTAG TTGAGCAAGT GAAGAAGCTA TTTACCAAGT TGTCATCTGA CTCAACTTCT ACTACTCAAC TAGTTGCCAA GGAACCTGCC AGTTTTACTG GTTCTGAG--
Bjuv_B04_VARUNA_g5554.t1 GTGATTGCTG CGGCAGGAGC TGTGAAGCAT GAGGAAGTAG TTGAGCAAGT GAAGAAGCTA TTTACCAAGT TGTCATCTGA CTCAACTTCT ACTACTCAAC TAGTTGCCAA GGAACCTGCC AGTTTTACTG GTTCTGAG--
BniC2_B01g064900.1_pseudo GTGATCGCTG CGGCAGGAGC TGTGAAGCAT GAGGAAGTAG TTGAGCAAGT GAAGAAGCTA TTTACCAAGT TGTCATCTGA CTCAACTTCT ACTACTCAAC TAGTTGCCAA GGAACCTGCC AGTTTTACTG GTTCTGAG--
BniN100_B01g060920.2     GTGATCGCTG CGGCAGGAGC TGTGAAGCAT GAGGAAGTAG TTGAGCAAGT GAAGAAGCTA TTTACCAAGT TGTCATCTGA CTCAACTTCT ACTACTCAAC TAGTTGCCAA AGAACCTGCC AGTTTTACTG GTTCTGAG--

                       985        995       1005       1015       1025       1035       1045       1055       1065       1075       1085       1095       1105       1115
BraTUE_A05p41820.1       -GTTCGAATG ATTGATGACG ATCTACCCCT TGCACAATTT GCTGTGGCCT TCGAAGGAGC TTCTTGGACA GATCCAGATT CCGTTGCTCT TATGGTTATG CAAACCATGT TGGGTTCTTG GAACAAAAAC GTTGGTGGTG
BraPCA_A05p42570.1       -GTTCGAATG ATTGATGACG ATCTACCCCT TGCACAATTT GCTGTGGCCT TCGAAGGAGC TTCTTGGACA GATCCAGATT CCGTTGCTCT TATGGTTATG CAAACCATGT TGGGTTCTTG GAACAAAAAC GTTGGTGGTG
BraCCB_A05p40150.1       -GTTCGAATG ATTGATGACG ATCTACCCCT TGCACAATTT GCTGTGGCCT TCGAAGGAGC TTCTTGGACA GATCCAGATT CCGTTGCTCT TATGGTTATG CAAACCATGT TGGGTTCTTG GAACAAAAAC GTTGGTGGTG
BraZ1_A05t23166          -GTTCGAATG ATTGATGACG ATCTACCCCT TGCACAATTT GCTGTGGCCT TCGAAGGAGC TTCTTGGACA GATCCAGATT CCGTTGCTCT TATGGTTATG CAAACCATGT TGGGTTCTTG GAACAAAAAC GTTGGTGGTG
Bjuv_A05_VARUNA_g4271.t1 -GTTCGAATG ATTGATGACG ATCTACCCCT TGCACAATTT GCTGTGGCCT TCGAAGGAGC TTCTTGGACA GATCCAGATT CCGTTGCTCT TATGGTTATG CAAACCATGT TGGGTTCTTG GAACAAAAAC GTTGGTGGTG
Bjut_A021166             -GTTCGAATG ATTGATGACG ATCTACCCCT TGCACAATTT GCTGTGGCCT TCGAAGGAGC TTCTTGGACA GATCCAGATT CCGTTGCTCT TATGGTTATG CAAACCATGT TGGGTTCTTG GAACAAAAAC GTTGGTGGTG
Bjut_0011533             -GTTCGAATG ATCGATGATG ATCTACCCCT TGCACAATTT GCTGTTGCCT TTGAAGGAGC ATCTTGGACA GATCCAGATT CCGTTGCTCT TATGGTTATG CAAACCATGT TGGGTTCATG GAACAAAAAT GTTGGTGGTG
Bjuv_B04_VARUNA_g5554.t1 -GTTCGAATG ATCGATGATG ATCTACCCCT TGCACAATTT GCTGTTGCCT TTGAAGGAGC ATCTTGGACA GATCCAGATT CCGTTGCTCT TATGGTTATG CAAACCATGT TGGGTTCATG GAACAAAAAT GTTGGTGGTG
BniC2_B01g064900.1_pseudo -GTTCGAATG ATCGATGATG ATCTACCC-T TGCACAATTT GCTGTTGCCT TTGAAGGAGC ATCTTGGACA GATCCAGATT CCGTTGCTCT TATGGTTATG CAAACCATGT TGGGTTCATG GAACAAAAAT GTTGGTGGTG
BniN100_B01g060920.2     -GTTCGAATG ATCGATGATG ATCTACCCCT TGCACAATTT GCTGTTGCAT TTGAAGGAGC ATCTTGGACA GATCCAGATT CCGTTGCTCT TATGGTTATG CAAACCATGT TGGGTTCATG GAACAAAAAT GTTGGTGGTG

                      1125       1135       1145       1155       1165       1175       1185       1195       1205       1215       1225       1235       1245       1255
BraTUE_A05p41820.1       GCAAGCACAT GGG---GTCT GCCCTGACCC AGAGGGTTGC CGTTAATGAA ATAGCGGAAA GCATAATGGC ATTCAACACC AACTACAAGG ATACTGGACT TTTCGGCGTG TACGCAGTTG CTAAG---GC TGATTGCTTA
BraPCA_A05p42570.1       GCAAGCACAT GGG---GTCT GCCCTGACCC AGAGGGTTGC CGTTAATGAA ATAGCGGAAA GCATAATGGC ATTCAACACC AACTACAAGG ATACTGGACT TTTCGGCGTG TACGCAGTTG CTAAG---GC TGATTGCTTA
BraCCB_A05p40150.1       GCAAGCACAT GGG---GTCT GCCCTGACCC AGAGGGTTGC CGTTAATGAA ATAGCGGAAA GCATAATGGC ATTCAACACC AACTACAAGG ATACTGGACT TTTCGGCGTG TACGCAGTTG CTAAG---GC TGATTGCTTA
BraZ1_A05t23166          GCAAGCACAT GGG---GTCT GCCCTGACCC AGAGGGTTGC CGTTAATGAA ATAGCGGAAA GCATAATGGC ATTCAACACC AACTACAAGG ATACTGGACT TTTCGGCGTG TACGCAGTTG CTAAG---GC TGATTGCTTA
Bjuv_A05_VARUNA_g4271.t1 GCAAGCACAT GGG---GTCT GCCCTGACCC AGAGGGTTGC CGTTAATGAA ATAGCGGAAA GCATAATGGC ATTCAACACC AACTACAAGG ATACTGGACT TTTCGGCGTG TACGCAGTTG CTAAG---GC TGATTGCTTA
Bjut_A021166             GCAAGCACAT GGG---GTCT GCCCTGACCC AGAGGGTTGC CGTTAATGAA ATAGCGGAAA GCATAATGGC ATTCAACACC AACTACAAGG ATACTGGACT TTTCGGCGTG TACGCAGTTG CTAAG---GC TGATTGCTTA
Bjut_0011533             GCAAACACAT GGG---GTCT GACCTGACCC AGAGGGTTGC CATTAATGAA ATAGCGGAAA GCATAATGGC ATTCAACACC AACTACAAGG ATACCGGACT GTTCGGCGTG TACGCTGTTG CTAAG---GC CGATTGCTTA
Bjuv_B04_VARUNA_g5554.t1 GCAAACACAT GGG---GTCT GACCTGACCC AGAGGGTTGC CATTAATGAA ATAGCGGAAA GCATAATGGC ATTCAACACC AACTACAAGG ATACCGGACT GTTCGGCGTG TACGCTGTTG CTAAG---GC CGATTGCTTA
BniC2_B01g064900.1_pseudo GCAAACACAT GGG---GTCT GACCTGACCC AGAGGGTTGC CATTAATGAA ATAGCGGAAA GCATAATGGC ATTCAACACC AACTACAAGG ATACTGGACT TTTCGGCGTG TACGCAGTTG CTAAG---GC CGATTGCTTA
BniN100_B01g060920.2     GCAAACACAT GGG---GTCT GACTTGACCC AGAGGGTTGC CATTAATGAA ATAGCGGAAA GCATAATGGC ATTCAACACC AACTACAAGG ATACTGGACT TTTCGGCGTG TACGCAGTTG CTAAG---GC CGATTGCTTA
```

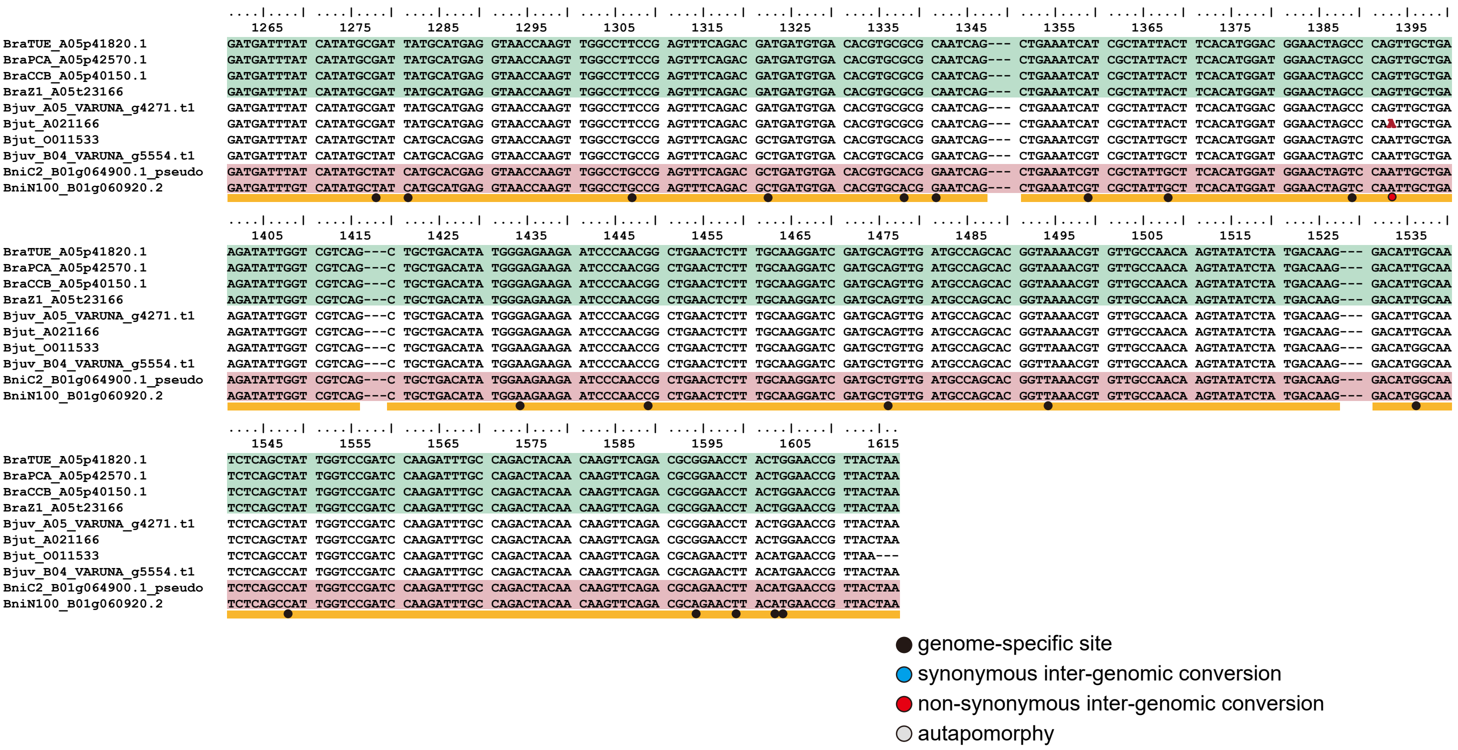

1265 1275 1285 1295 1305 1315 1325 1335 1345 1355 1365 1375 1385 1395
BraTUE_A05p41820.1 GATGATTTAT CATATGCGAT TATGCATGAG GTAACCAAGT TGGCCTTCCG AGTTTCAGAC GATGATGTGA CACGTGCGCG CAATCAG--- CTGAAATCAT CGCTATTACT TCACATGGAC GGAACTAGCC CAGTTGCTGA
BraPCA_A05p42570.1 GATGATTTAT CATATGCGAT TATGCATGAG GTAACCAAGT TGGCCTTCCG AGTTTCAGAC GATGATGTGA CACGTGCGCG CAATCAG--- CTGAAATCAT CGCTATTACT TCACATGGAT GGAACTAGCC CAGTTGCTGA
BraCCB_A05p40150.1 GATGATTTAT CATATGCGAT TATGCATGAG GTAACCAAGT TGGCCTTCCG AGTTTCAGAC GATGATGTGA CACGTGCGCG CAATCAG--- CTGAAATCAT CGCTATTACT TCACATGGAT GGAACTAGCC CAGTTGCTGA
BraZ1_A05t23166 GATGATTTAT CATATGCGAT TATGCATGAG GTAACCAAGT TGGCCTTCCG AGTTTCAGAC GATGATGTGA CACGTGCGCG CAATCAG--- CTGAAATCAT CGCTATTACT TCACATGGAT GGAACTAGCC CAGTTGCTGA
Bjuv_A05_VARUNA_g4271.t1 GATGATTTAT CATATGCGAT TATGCATGAG GTAACCAAGT TGGCCTTCCG AGTTTCAGAC GATGATGTGA CACGTGCGCG CAATCAG--- CTGAAATCAT CGCTATTACT TCACATGGAC GGAACTAGCC CAGTTGCTGA
Bjut_A021166 GATGATTTAT CATATGCGAT TATGCATGAG GTAACCAAGT TGGCCTTCCG AGTTTCAGAC GATGATGTGA CACGTGCGCG CAATCAG--- CTGAAATCAT CGCTATTACT TCACATGGAT GGAACTAGCC CAATTGCTGA
Bjut_O011533 GATGATTTAT CATATGCTAT CATGCACGAG GTAACCAAGT TGGCCTGCCG AGTTTCAGAC GCTGATGTGA CACGTGCACG GAATCAG--- CTGAAATCGT CGCTATTGCT TCACATGGAT GGAACTAGTC CAATTGCTGA
Bjuv_B04_VARUNA_g5554.t1 GATGATTTAT CATATGCTAT CATGCACGAG GTAACCAAGT TGGCCTGCCG AGTTTCAGAC GCTGATGTGA CACGTGCACG GAATCAG--- CTGAAATCGT CGCTATTGCT TCACATGGAT GGAACTAGTC CAATTGCTGA
BniC2_B01g064900.1_pseudo GATGATTTAT CATATGCTAT CATGCACGAG GTAACCAAGT TGGCCTGCCG AGTTTCAGAC GCTGATGTGA CACGTGCACG GAATCAG--- CTGAAATCGT CGCTATTGCT TCACATGGAT GGAACTAGTC CAATTGCTGA
BniN100_B01g060920.2 GATGATTTGT CATATGCTAT CATGCATGAG GTAACCAAGT TGGCCTGCCG AGTTTCAGAC GCTGATGTGA CACGTGCACG GAATCAG--- CTGAAATCGT CGCTATTGCT TCACATGGAT GGAACTAGTC CAATTGCTGA
1405 1415 1425 1435 1445 1455 1465 1475 1485 1495 1505 1515 1525 1535
BraTUE_A05p41820.1 AGATATTGGT CGTCAG---C TGCTGACATA TGGGAGAAGA ATCCCAACGG CTGAACTCTT TGCAAGGATC GATGCAGTTG ATGCCAGCAC GGTAAAACGT GTTGCCAACA AGTATATCTA TGACAAG--- GACATTGCAA
BraPCA_A05p42570.1 AGATATTGGT CGTCAG---C TGCTGACATA TGGGAGAAGA ATCCCAACGG CTGAACTCTT TGCAAGGATC GATGCAGTTG ATGCCAGCAC GGTAAAACGT GTTGCCAACA AGTATATCTA TGACAAG--- GACATTGCAA
BraCCB_A05p40150.1 AGATATTGGT CGTCAG---C TGCTGACATA TGGGAGAAGA ATCCCAACGG CTGAACTCTT TGCAAGGATC GATGCAGTTG ATGCCAGCAC GGTAAAACGT GTTGCCAACA AGTATATCTA TGACAAG--- GACATTGCAA
BraZ1_A05t23166 AGATATTGGT CGTCAG---C TGCTGACATA TGGGAGAAGA ATCCCAACGG CTGAACTCTT TGCAAGGATC GATGCAGTTG ATGCCAGCAC GGTAAAACGT GTTGCCAACA AGTATATCTA TGACAAG--- GACATTGCAA
Bjuv_A05_VARUNA_g4271.t1 AGATATTGGT CGTCAG---C TGCTGACATA TGGGAGAAGA ATCCCAACGG CTGAACTCTT TGCAAGGATC GATGCAGTTG ATGCCAGCAC GGTAAAACGT GTTGCCAACA AGTATATCTA TGACAAG--- GACATTGCAA
Bjut_A021166 AGATATTGGT CGTCAG---C TGCTGACATA TGGGAGAAGA ATCCCAACGG CTGAACTCTT TGCAAGGATC GATGCAGTTG ATGCCAGCAC GGTAAAACGT GTTGCCAACA AGTATATCTA TGACAAG--- GACATTGCAA
Bjut_O011533 AGATATTGGT CGTCAG---C TGCTGACATA TGGAAGAAGA ATCCCAACCG CTGAACTCTT TGCAAGGATC GATGCTGTTG ATGCCAGCAC GGTTAAACGT GTTGCCAACA AGTATATCTA TGACAAG--- GACATGGCAA
Bjuv_B04_VARUNA_g5554.t1 AGATATTGGT CGTCAG---C TGCTGACATA TGGAAGAAGA ATCCCAACCG CTGAACTCTT TGCAAGGATC GATGCTGTTG ATGCCAGCAC GGTTAAACGT GTTGCCAACA AGTATATCTA TGACAAG--- GACATGGCAA
BniC2_B01g064900.1_pseudo AGATATTGGT CGTCAG---C TGCTGACATA TGGAAGAAGA ATCCCAACCG CTGAACTCTT TGCAAGGATC GATGCTGTTG ATGCCAGCAC GGTTAAACGT GTTGCCAACA AGTATATCTA TGACAAG--- GACATGGCAA
BniN100_B01g060920.2 AGATATTGGT CGTCAG---C TGCTGACATA TGGAAGAAGA ATCCCAACCG CTGAACTCTT TGCAAGGATC GATGCTGTTG ATGCCAGCAC GGTTAAACGT GTTGCCAACA AGTATATCTA TGACAAG--- GACATGGCAA
1545 1555 1565 1575 1585 1595 1605 1615
BraTUE_A05p41820.1 TCTCAGCTAT TGGTCCGATC CAAGATTTGC CAGACTACAA CAAGTTCAGA CGCGGAACCT ACTGGAACCG TTACTAA
BraPCA_A05p42570.1 TCTCAGCTAT TGGTCCGATC CAAGATTTGC CAGACTACAA CAAGTTCAGA CGCGGAACCT ACTGGAACCG TTACTAA
BraCCB_A05p40150.1 TCTCAGCTAT TGGTCCGATC CAAGATTTGC CAGACTACAA CAAGTTCAGA CGCGGAACCT ACTGGAACCG TTACTAA
BraZ1_A05t23166 TCTCAGCTAT TGGTCCGATC CAAGATTTGC CAGACTACAA CAAGTTCAGA CGCGGAACCT ACTGGAACCG TTACTAA
Bjuv_A05_VARUNA_g4271.t1 TCTCAGCTAT TGGTCCGATC CAAGATTTGC CAGACTACAA CAAGTTCAGA CGCGGAACCT ACTGGAACCG TTACTAA
Bjut_A021166 TCTCAGCTAT TGGTCCGATC CAAGATTTGC CAGACTACAA CAAGTTCAGA CGCGGAACCT ACTGGAACCG TTACTAA
Bjut_O011533 TCTCAGCCAT TGGTCCGATC CAAGATTTGC CAGACTACAA CAAGTTCAGA CGCAGAACTT ACATGAACCG TTAA---
Bjuv_B04_VARUNA_g5554.t1 TCTCAGCCAT TGGTCCGATC CAAGATTTGC CAGACTACAA CAAGTTCAGA CGCAGAACTT ACATGAACCG TTACTAA
BniC2_B01g064900.1_pseudo TCTCAGCCAT TGGTCCGATC CAAGATTTGC CAGACTACAA CAAGTTCAGA CGCAGAACTT ACATGAACCG TTACTAA
BniN100_B01g060920.2 TCTCAGCCAT TGGTCCGATC CAAGATTTGC CAGACTACAA CAAGTTCAGA CGCAGAACTT ACATGAACCG TTACTAA
genome-specific site
synonymous inter-genomic conversion
non-synonymous inter-genomic conversion
autapomorphy

(B) Clade I BBCC

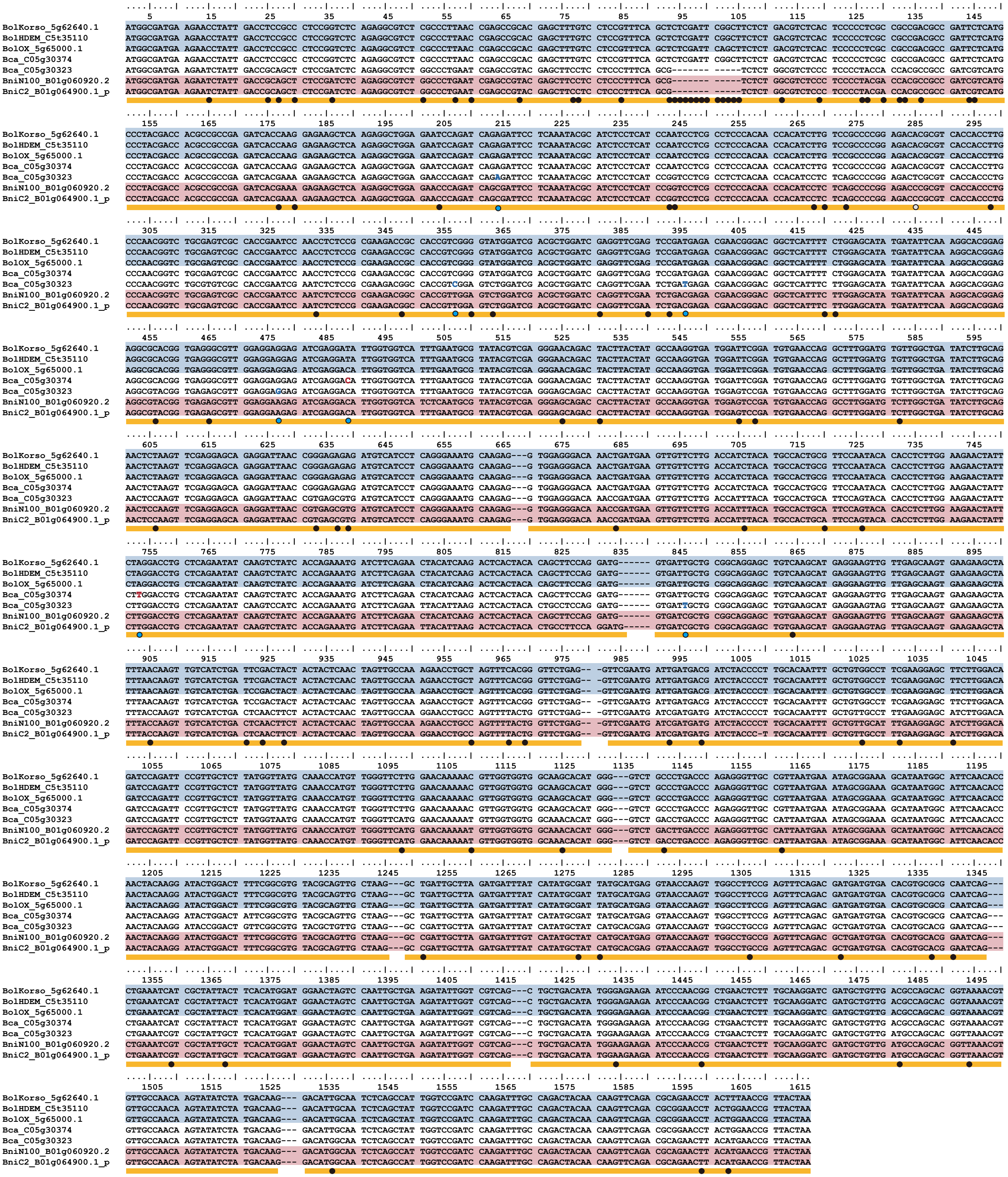

(C) Clade II AABB

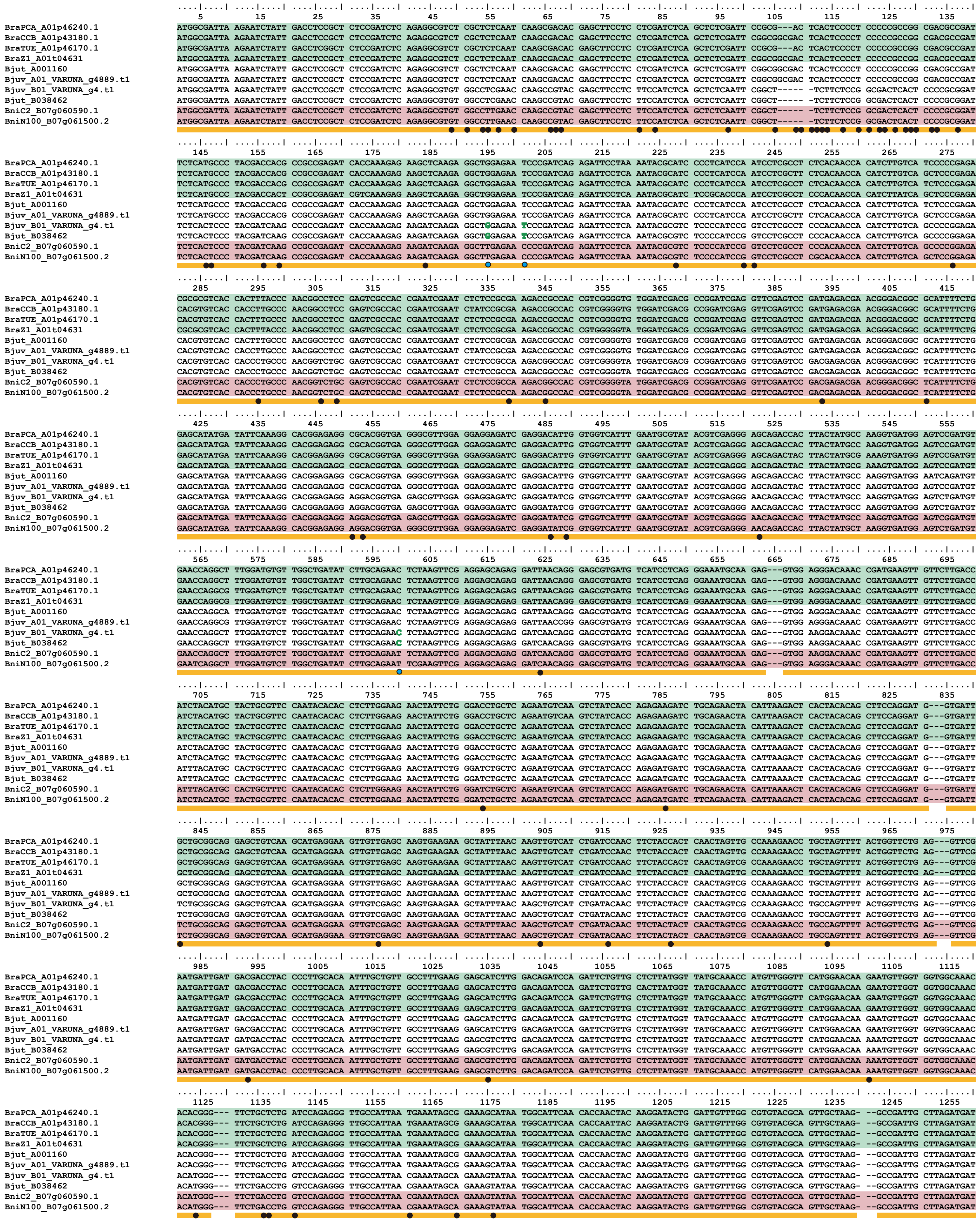

```
                              ....|....| ....|....| ....|....| ....|....| ....|....| ....|....| ....|....| ....|....| ....|....| ....|....| ....|....| ....|....| ....|....| ....|....|
                                  1265       1275       1285       1295       1305       1315       1325       1335       1345       1355       1365       1375       1385       1395
BraPCA_A01p46240.1            TTATCATATG CCATTATGCA TGAGGTAACG AAGTTGGCCT TCCGAGTTTC AGACGATGAT GTGACCCGTG CACGGAATCA G---CTCAAA TCGTCGCTTT TGCTTCACAT GGATGGAACT AGCCCAATTG CTGAAGATAT
BraCCB_A01p43180.1            TTATCATATG CCATTATGCA TGAGGTAACG AAGTTGGCCT TCCGAGTTTC AGACGATGAT GTGACCCGTG CACGGAATCA G---CTCAAA TCGTCGCTTT TGCTTCACAT GGATGGAACT AGCCCAATTG CTGAAGATAT
BraTUE_A01p46170.1            TTATCATATG CCATTATGCA TGAGGTAACG AAGTTGGCCT TCCGAGTTTC AGACGATGAT GTGACCCGTG CACGGAATCA G---CTCAAA TCGTCGCTTT TGCTTCACAT GGATGGAACT AGCCCAATTG CTGAAGATAT
BraZ1_A01t04631               TTATCATATG CAATTATGCA TGAGGTAACG AAGCTGGCCT TCCGAGTTTC AGACGATGAT GTGACCCGTG CACGGAATCA G---CTCAAA TCGTCGCTTT TGCTTCACAT GGATGGAACT AGCCCAATTG CTGAAGATAT
Bjut_A001160                  TTATCATATG CCATTATGCA TGAGGTAACG AAGTTGGCCT TCCGAGTTTC AGACGATGAT GTGACCCGTG CACGGAATCA G---CTCAAA TCGTCGCTTT TGCTTCACAT GGATGGAACT AGCCCAATTG CTGAAGATAT
Bjuv_A01_VARUNA_g4889.t1      TTATCATATG CCATTATGCA TGAGGTAACG AAGTTGGCCT TCCGAGTTTC AGACGATGAT GTGACCCGTG CACGGAATCA G---CTCAAA TCGTCGCTTT TGCTTCACAT GGATGGAACT AGCCCAATTG CTGAAGATAT
Bjuv_B01_VARUNA_g4.t1         TTATCATATG CCATTATGCA TGAGGTAACC AAGTTGGCCT TCCGAGTTTC AGACGCTGAT GTGACACGTG CACGGAATCA G---CTCAAA TCGTCGCTTT TGCTTCACAT GGATGGAACT AGCCCAATTG CTGAAGATAT
Bjut_B038462                  TTATCATATG CCATTATGCA TGAGGTAACC AAGTTGGCCT TCCGAGTTTC AGACGCTGAT GTGACACGTG CACGGAATCA G---CTCAAA TCGTCGCTTT TGCTTCACAT GGATGGAACT AGCCCAATTG CTGAAGATAT
BniC2_B07g060590.1            TTATCATATG CCATTATGCA TGAGGTAACC AAGTTGGCCT TCCGAGTTTC AGACGCTGAT GTGACACGTG CACGGAATCA G---CTCAAA TCGTCGCTTT TGCTTCACAT GGATGGAACT AGCCCAATTG CTGAAGATAT
BniN100_B07g061500.2          TTATCATATG CCATTATGCA TGAGGTAACC AAGTTGGCCT TCCGAGTTTC AGACGCTGAT GTGACACGTG CACGGAATCA G---CTCAAA TCGTCGCTTT TGCTTCACAT GGATGGAACT AGCCCAATTG CTGAAGATAT

                              ....|....| ....|....| ....|....| ....|....| ....|....| ....|....| ....|....| ....|....| ....|....| ....|....| ....|....| ....|....| ....|....| ....|....|
                                  1405       1415       1425       1435       1445       1455       1465       1475       1485       1495       1505       1515       1525       1535
BraPCA_A01p46240.1            TGGTCGTCAG ---CTGCTGA CCTATGGGAG AAGAATCCCA ACCGCTGAAC TATTTGCTAG GATCGATGCT GTTGATGCCA GCACCGTTAA ACGTGTGGCC AACAAGTATG TCTATGACAA G---GACATT GCAATCTCAG
BraCCB_A01p43180.1            TGGTCGTCAG ---CTGCTGA CCTATGGGAG AAGAATCCCA ACCGCTGAAC TATTTGCTAG GATCGATGCT GTTGATGCCA GCACCGTTAA ACGTGTGGCC AACAAGTTTG TCTATGACAA G---GACATT GCAATCTCAG
BraTUE_A01p46170.1            TGGTCGTCAG ---CTGCTGA CCTATGGGAG AAGAATCCCA ACCGCTGAAC TATTTGCTAG GATCGATGCT GTTGATGCCA GCACCGTTAA ACGTGTGGCC AACAAGTTTG TCTATGACAA G---GACATT GCAATCTCAG
BraZ1_A01t04631               TGGTCGTCAG ---CTGCTGA CCTATGGGAG AAGAATCCCA ACCGCTGAAC TATTTGCTAG GATCGATGCT GTTGATGCCA GCACCGTTAA ACGTGTGGCC AACAAGTATG TCTATGACAA G---GACATT GCAATCTCAG
Bjut_A001160                  TGGTCGTCAG ---CTGCTGA CCTATGGGAG AAGAATCCCA ACCGCTGAAC TATTTGCTAG GATCGATGCT GTTGATGCCA GCACCGTTAA ACGTGTGGCC AACAAGTATG TCTATGACAA G---GACATT GCAATCTCAG
Bjuv_A01_VARUNA_g4889.t1      TGGTCGTCAG ---CTGCTGA CCTATGGGAG AAGAATCCCA ACCGCTGAAC TATTTGCTAG GATCGATGCT GTTGATGCCA GCACCGTTAA ACGTGTGGCC AACAAGTATG TCTATGACAA G---GACATT GCAATCTCAG
Bjuv_B01_VARUNA_g4.t1         TGGTCGTCAG ---CTGCTGA CCTATGGGAG AAGAATCCCA ACCGCTGAAC TATTTGCTAG GATCGATGCT GTTGATGCCA GCACAGTTAA ACGTGTTGCC AACAAGTATG TCTATGACAA G---GACATT GCAATCTCAG
Bjut_B038462                  TGGTCGTCAG ---CTGCTGA CCTATGGGAG AAGAATCCCA ACCGCTGAAC TATTTGCTAG GATCGATGCT GTTGATGCCA GCACAGTTAA ACGTGTTGCC AACAAGTATG TCTATGACAA G---GACATT GCAATCTCAG
BniC2_B07g060590.1            TGGTCGTCAG ---CTGCTGA CCTATGGGAG AAGAATCCCA ACCGCTGAAC TATTTGCTAG GATCGATGCT GTTGATGCCA GCACAGTTAA ACGTGTTGCC AACAAGTATG TCTATGACAA G---GACATT GCAATCTCAG
BniN100_B07g061500.2          TGGTCGTCAG ---CTGCTGA CCTATGGGAG AAGAATCCCA ACCGCTGAAC TATTTGCTAG GATCGATGCT GTTGATGCCA GCACAGTTAA ACGTGTTGCC AACAAGTATG TCTATGACAA G---GACATT GCAATCTCAG

                              ....|....| ....|....| ....|....| ....|....| ....|....| ....|....| ....|....| .
                                  1545       1555       1565       1575       1585       1595       1605
BraPCA_A01p46240.1            CCATTGGTCC GATCCAAGAT TTGCCAGACT ACAACAAGTT CAGACGCAGA ACCTACTTTA ACCGTTACTA A
BraCCB_A01p43180.1            CCATTGGTCC GATCCAAGAT TTGCCAGACT ACAACAAGTT CAGACGCAGA ACCTACTTTA ACCGTTACTA A
BraTUE_A01p46170.1            CCATTGGTCC GATCCAAGAT TTGCCAGACT ACAACAAGTT CAGACGCAGA ACCTACTTTA ACCGTTACTA A
BraZ1_A01t04631               CCATTGGTCC GATCCAAGAT TTGCCAGACT ACAACAAGTT CAGACGCAGA ACCTACTTTA ACCGTTACTA A
Bjut_A001160                  CCATTGGTCC GATCCAAGAT TTGCCAGACT ACAACAAGTT CAGACGCAGA ACCTACTTTA ACCGTTAA-- -
Bjuv_A01_VARUNA_g4889.t1      CCATTGGTCC GATCCAAGAT TTGCCAGACT ACAACAAGTT CAGACGCAGA ACCTACTTTA ACCGTTACTA A
Bjuv_B01_VARUNA_g4.t1         CCATTGGTCC GATTCAAGAT TTGCCAGACT ACAACAAGTT CAGACGCAGA ACCTACTTCA ACCGTTACTA A
Bjut_B038462                  CCATTGGTCC GATTCAAGAT TTGCCAGACT ACAACAAGTT CAGACGCAGA ACCTACTTCA ACCGTTACTA A
BniC2_B07g060590.1            CCATTGGTCC GATTCAAGAT TTGCCAGACT ACAACAAGTT CAGACGCAGA ACTTACTTCA ACCGTTACTA A
BniN100_B07g061500.2          CCATTGGTCC GATTCAAGAT TTGCCAGACT ACAACAAGTT CAGACGCAGA ACTTACTTCA ACCGTTACTA A
```

(D) Clade II BBCC

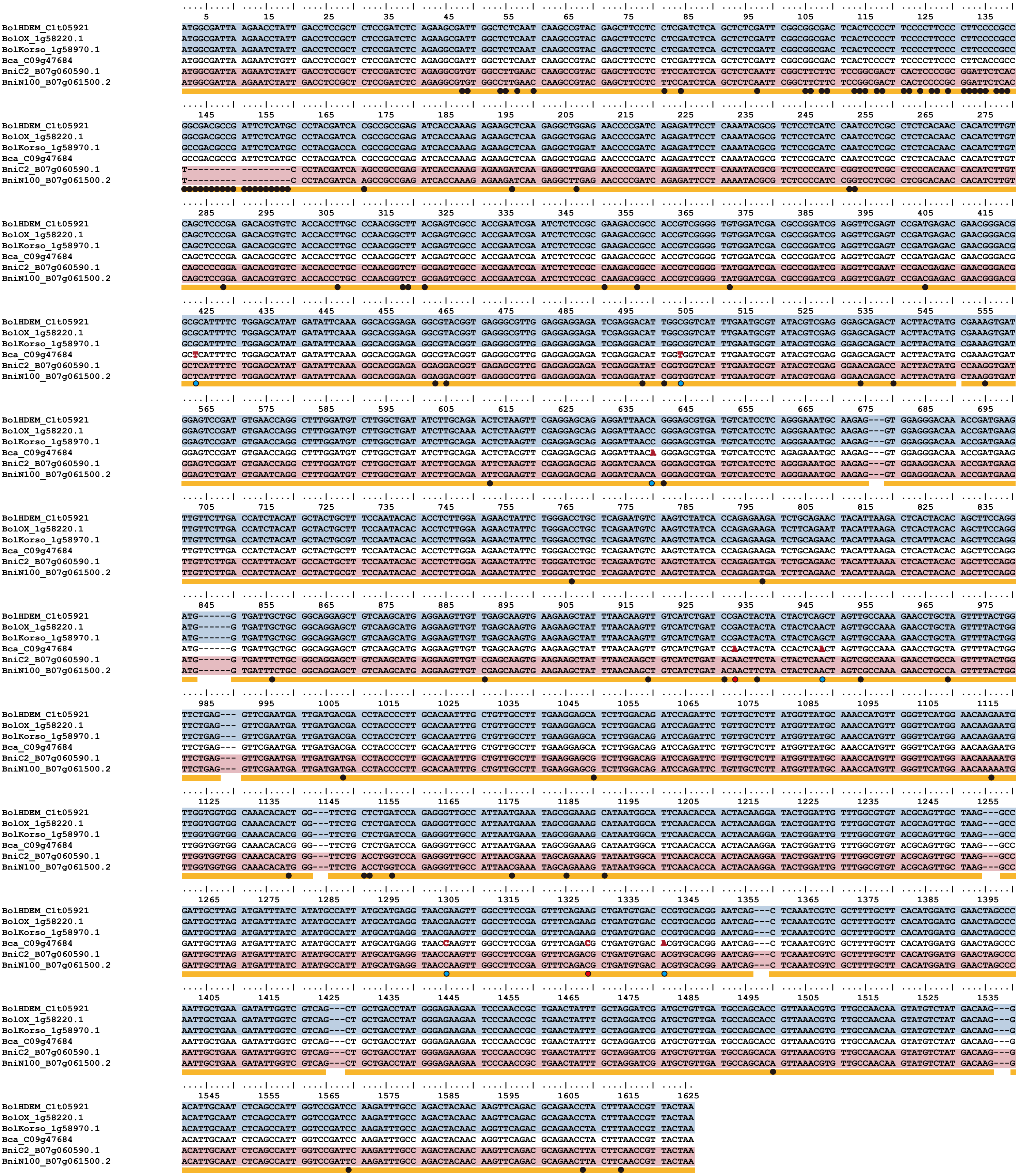

Supplementary Fig S11. Alignment of coding region of genes encoded MPP-β subunit of the mitochondrial complex III in studied genomes/subgenomes. The shade in green indicates *B. rapa* (AA), red indicates *B. nigra* (BB), and blue indicates *B. oleracea* (CC). The black dot indicates genome-specific site, blue dot indicates synonymous inter-genomic conversion, red dot indicates non-synonymous inter-genomic conversion, and grey dot indicates autapomorphy.
