# Supplementary material for: Cytonuclear Interactions and Subgenome Dominance Shape the Evolution of Organelle-Targeted Genes in the Brassica Triangle of U
Source: Mol Biol Evol. 2024 Feb 23;41(3):msae043. doi: 10.1093/molbev/msae043 (PMC10919925; doi:10.1093/molbev/msae043)
Supplement: msae043_Supplementary_Data [file msae043_supplementary_data.zip › Supplementary Figure S12.pdf]

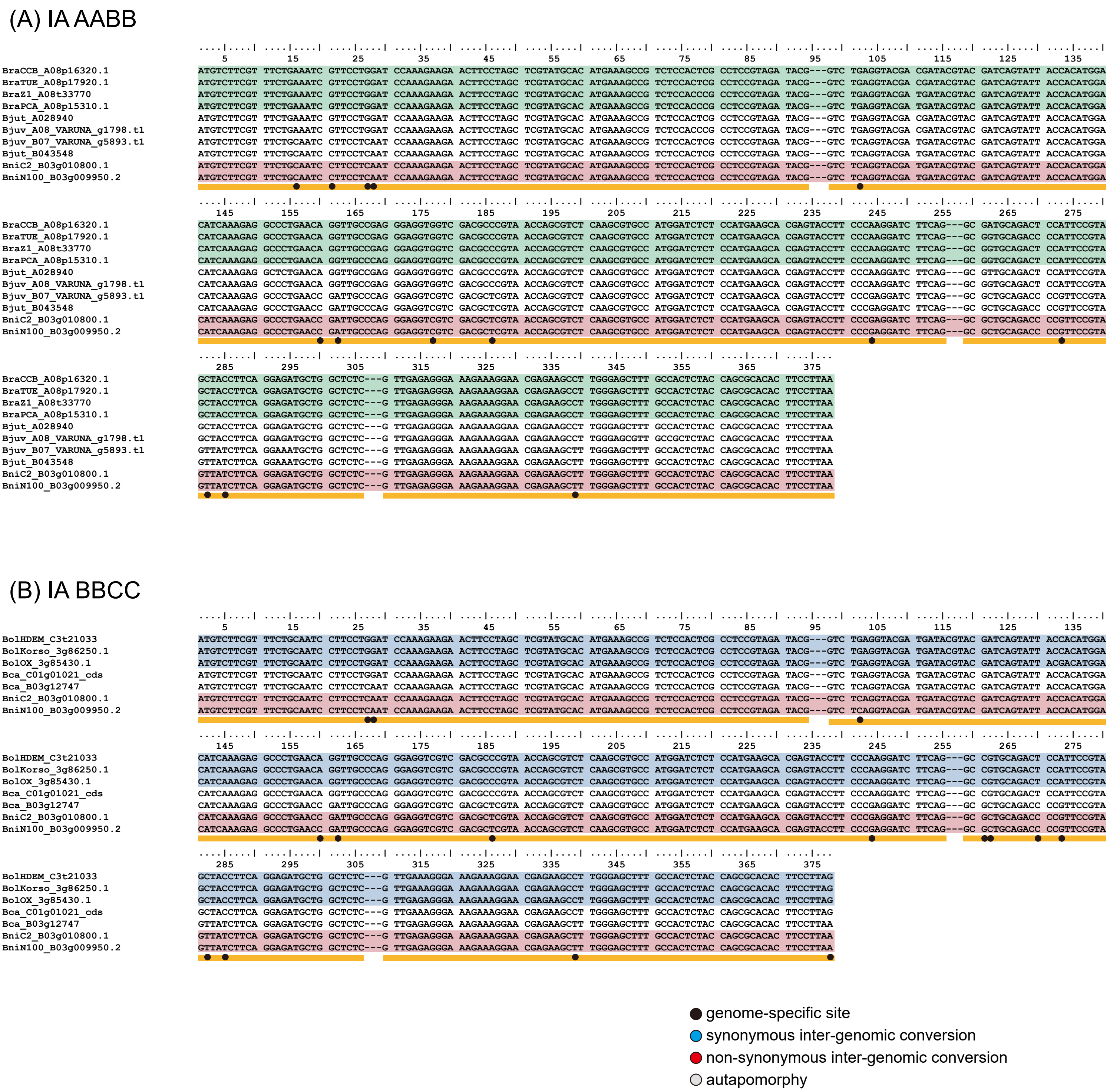

(A) IA AABB
BraCCB_A08p16320.1
BraTUE_A08p17920.1
BraZ1_A08t33770
BraPCA_A08p15310.1
Bjut_A028940
Bjuv_A08_VARUNA_g1798.t1
Bjuv_B07_VARUNA_g5893.t1
Bjut_B043548
BniC2_B03g010800.1
BniN100_B03g009950.2
(B) IA BBCC
BolHDEM_C3t21033
BolKorso_3g86250.1
BolOX_3g85430.1
Bca_C01g01021_cds
Bca_B03g12747
BniC2_B03g010800.1
BniN100_B03g009950.2
genome-specific site
synonymous inter-genomic conversion
non-synonymous inter-genomic conversion
autapomorphy

## (C) II AABB

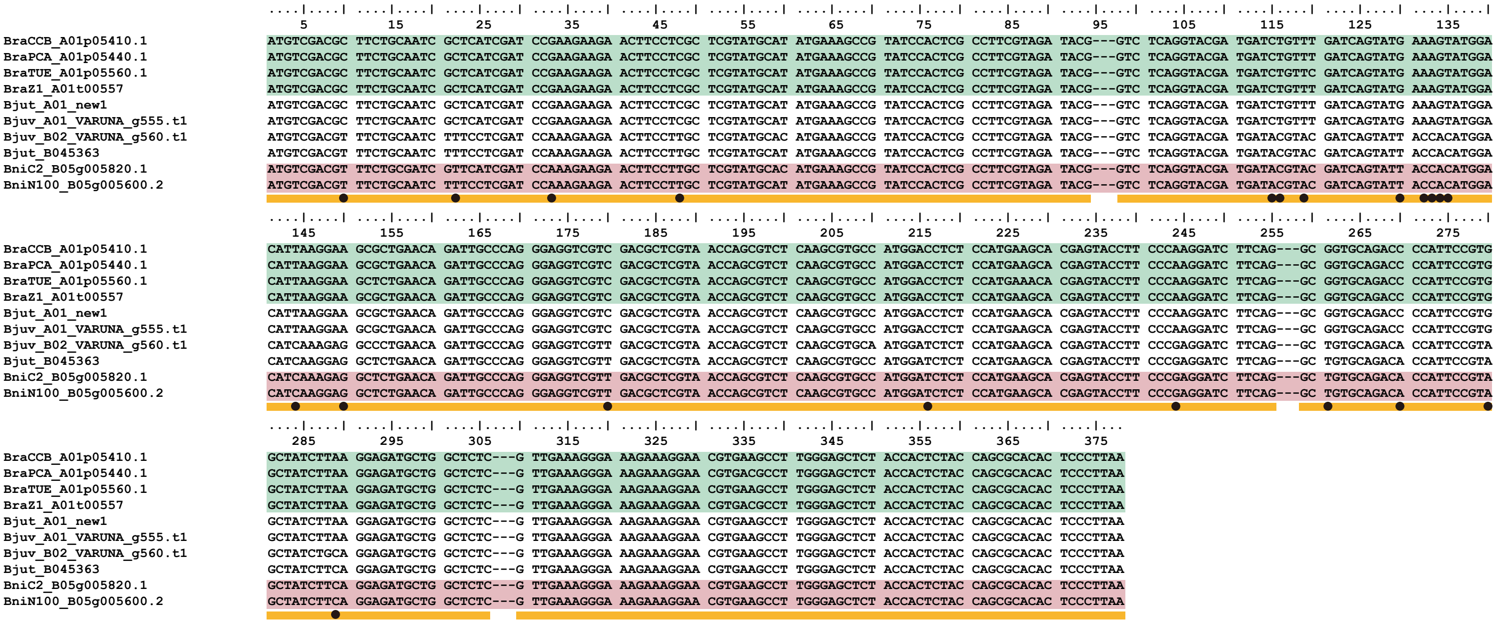


## (D) II BBCC

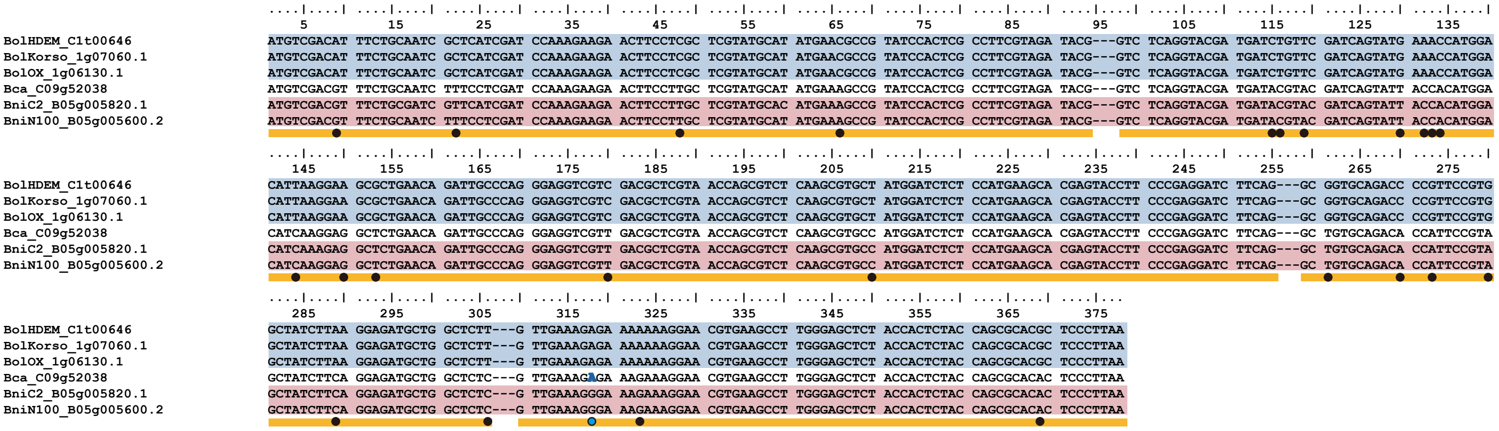


**Supplementary Fig S12. Alignment of coding region of genes encoded QCR7 subunit of the mitochondrial complex III in studied genomes/subgenomes.** The shade in green indicates *B. rapa* (AA), red indicates *B. nigra* (BB), and blue indicates *B. oleracea* (CC). The black dot indicates genome-specific site, blue dot indicates synonymous inter-genomic conversion, red dot indicates non-synonymous inter-genomic conversion, and grey dot indicates autapomorphy.
