# Supplementary material for: Cytonuclear Interactions and Subgenome Dominance Shape the Evolution of Organelle-Targeted Genes in the Brassica Triangle of U
Source: Mol Biol Evol. 2024 Feb 23;41(3):msae043. doi: 10.1093/molbev/msae043 (PMC10919925; doi:10.1093/molbev/msae043)
Supplement: msae043_Supplementary_Data [file msae043_supplementary_data.zip › Supplementary Figure S14.pdf]

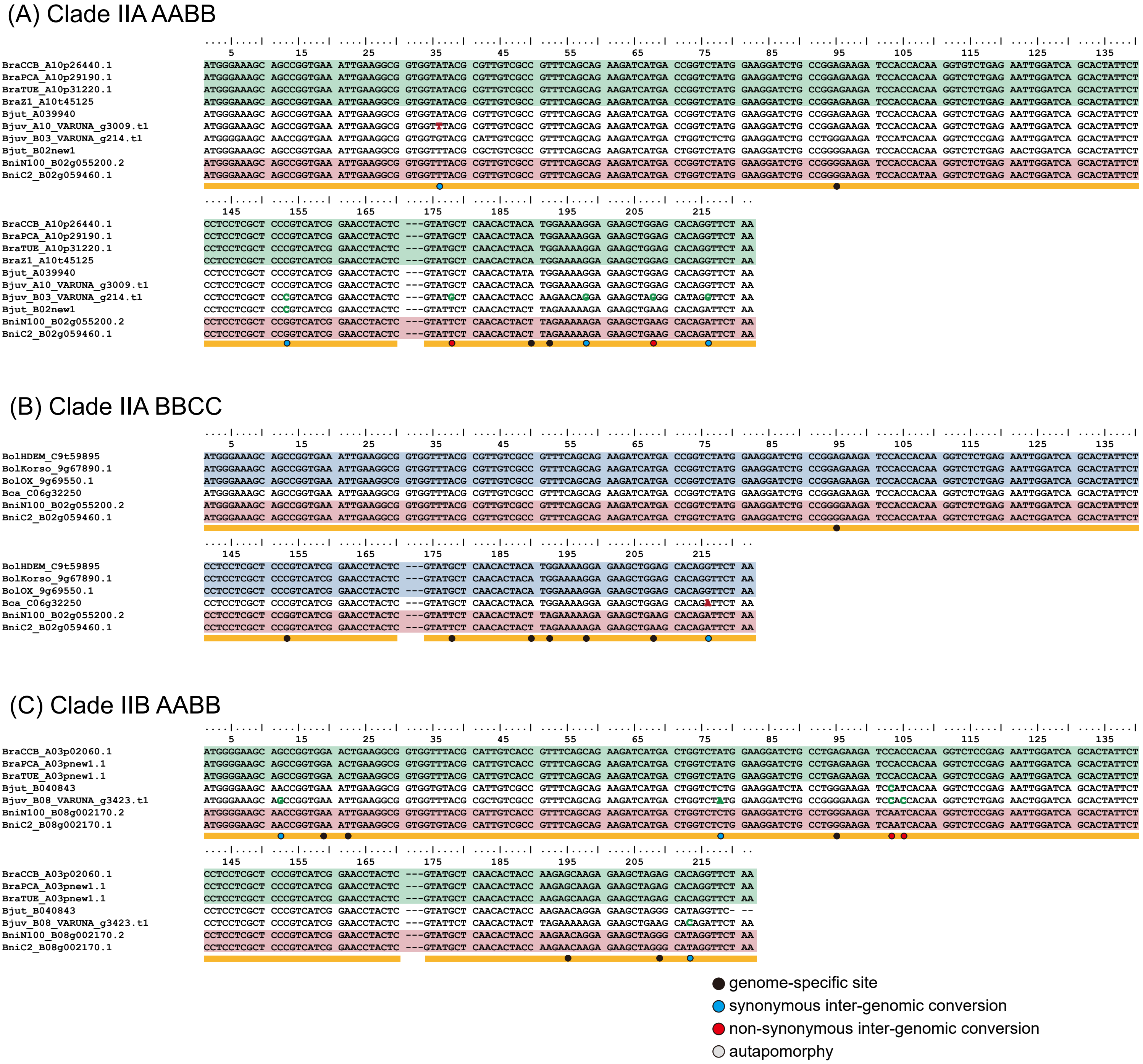

Supplementary Fig S14. Alignment of coding region of genes encoded QCR8 subunit of the mitochondrial complex III in studied genomes/subgenomes. The shade in green indicates *B. rapa* (AA), red indicates *B. nigra* (BB), and blue indicates *B. oleracea* (CC). The black dot indicates genome-specific site, blue dot indicates synonymous inter-genomic conversion, red dot indicates non-synonymous inter-genomic conversion, and grey dot indicates autapomorphy.
