# Supplementary material for: Cytonuclear Interactions and Subgenome Dominance Shape the Evolution of Organelle-Targeted Genes in the Brassica Triangle of U
Source: Mol Biol Evol. 2024 Feb 23;41(3):msae043. doi: 10.1093/molbev/msae043 (PMC10919925; doi:10.1093/molbev/msae043)
Supplement: msae043_Supplementary_Data [file msae043_supplementary_data.zip › Supplementary Figure S15.pdf]

(A) Clade IA1 AABB

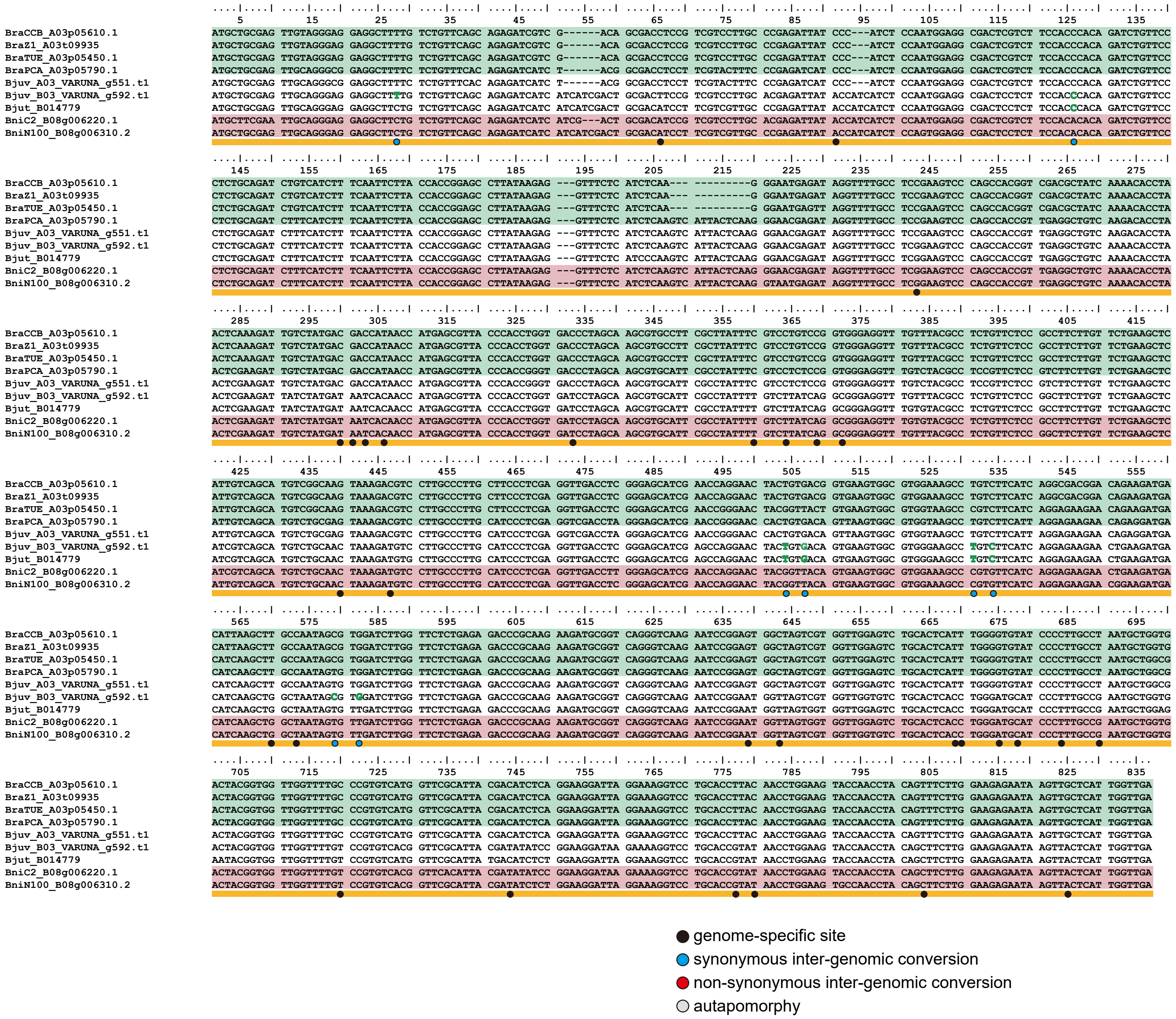

(B) Clade IA1 BBCC

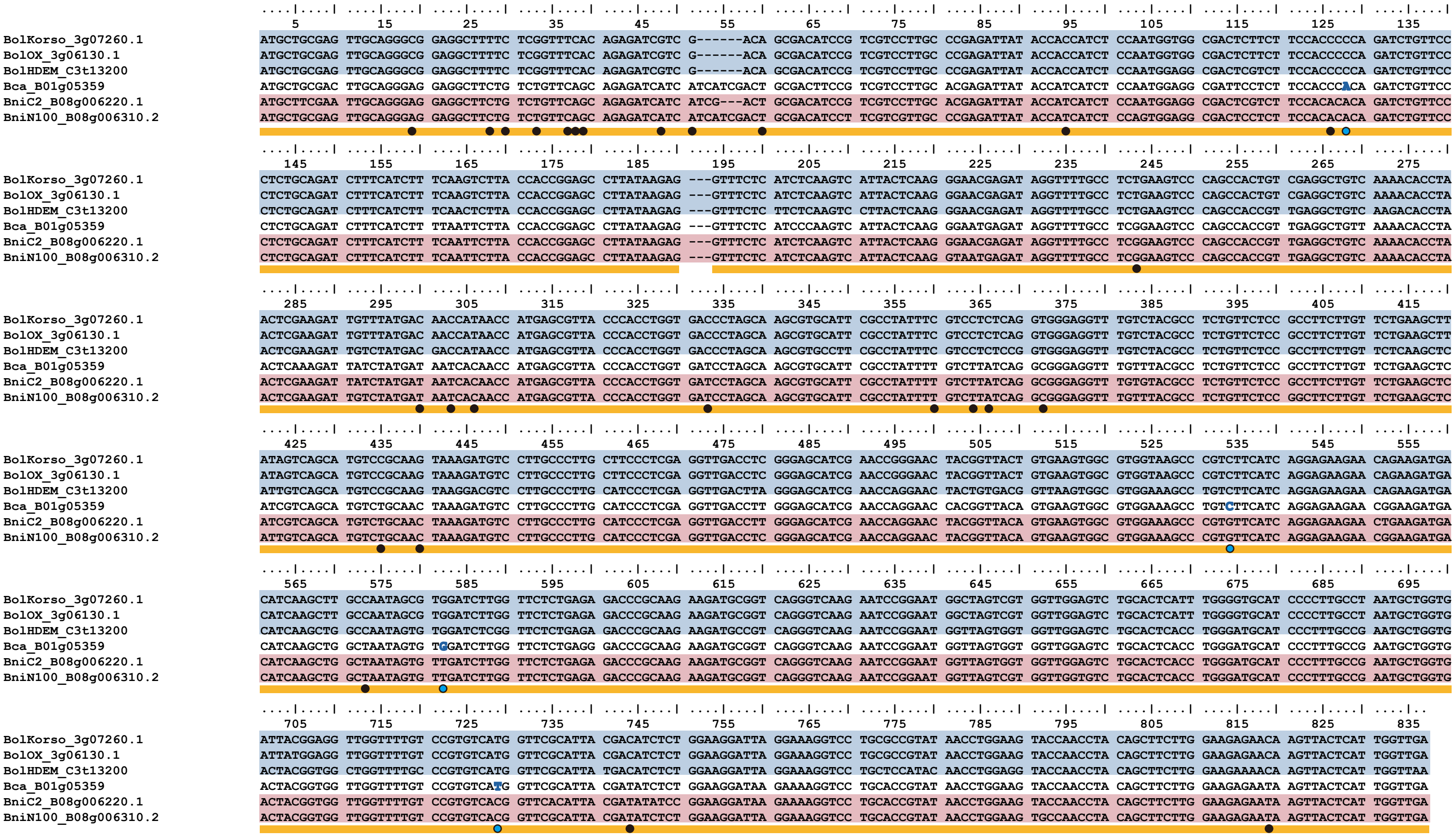

(C) Clade 2A AABB

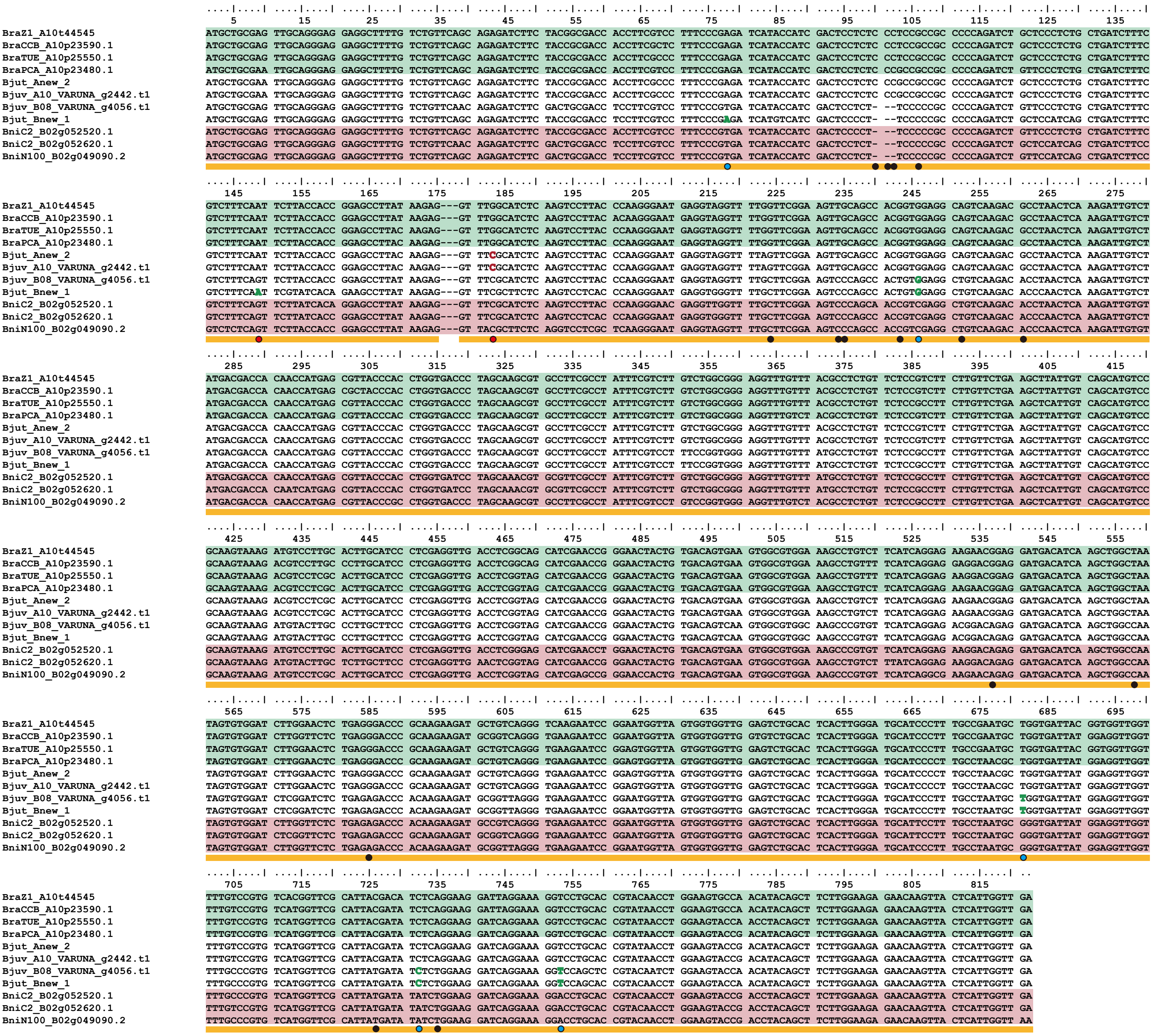

(D) Clade IA2 BBCC

```
                        5          15         25         35         45         55         65         75         85         95         105        115        125        135
BolKorso_9g60640.1      ATGCTGCGAA TTGCAGGGAG GAGGCTATTG TCTGTTCAGC AGAGATCTTG TACCGCGACC ACCTTCGTCC TTTCCCGAGA TCATACCATC GACTCCTCTC CCGCCGCCCC CAGATCTGCT CCCTCTGCTG ATCTTTCGTC
BolOX_9g62530.1         ATGCTGCGAA TTGCAGGGAG GAGGCTATTG TCTGTTCAGC AGAGATCTTG TACCGCGACC ACCTTCGTCC TTTCCCGAGA TCATACCATC GACTCCTCTC CCGCCGCCCC CAGATCTGCT CCCTCTGCTG ATCTTTCGTC
BolHDEM_C9t59207        ATGCTGCGAA TTGCAGGGAG GAGGCTATTG TCTGTTCAGC AGAGATCTTG TACCGCGACC ACCTTCGTCC TTTCCCGAGA TCATACCATC GACTCCTCTC CCGCCGCCCC CAGATCTGCT CCCTCTGCTG ATCTTTCGTC
Bca_C04g19116           ATGCTGCGAG TTGCAGGGAG GAGGCTTTTG TCTGTTCAGC AGAGATCTTC TACCGCGACC ACCTTCGTCC TTTCCCGTGA TCATACCATC GACTCCCCTT CCCCCGCCCC CAGATCTGTT CCCTCTGCTG ATCTTTCGTC
BniC2_B02g052520.1      ATGCTGCGAG TTGCAGGGAG GAGGCTTTTG TCTGTTCAGC AGAGATCTTC TACCGCGACC ACCTTCGTCC TTTCCCGTGA TCATACCATC GACTCCCCTT CCCCCGCCCC CAGATCTGTT CCCTCTGCTG ATCTTTCGTC
BniC2_B02g052620.1      ATGCTGCGAG TTGCAGGGAG GAGGCTTTTG TCTGTTCAAC AGAGATCTTC GACTGCGACC TCCTTCGTCC TTTCCCGTGA TCATACCATC GACTCCTCTT CCCCCGCCCC CAGATCTGCT CCATCAGCTG ATCTTCCGTC
BniN100_B02g049090.2    ATGCTGCGAG TTGCAGGGAG GAGGCTTTTG TCTGTTCAGC AGAGATCTTC GACTGCGACC TCCTTCGTCC TTTCCCGTGA TCATACCATC GACTCCTCTT CCCCCGCCCC CAGATCTGTT CCATCAGCTG ATCTTCCGTC

                        145        155        165        175        185        195        205        215        225        235        245        255        265        275
BolKorso_9g60640.1      GTTCAATTCT TACCACCGGA GCCTTATAAG AG---GTTTA GCTTCTCAAG TCCTTACCCA AGGGAATGAG GTAGGTTTTG CTTCGGAAGT TCCAGCCACG GTGGAGGCAG TCAAGACACC TAACTCAAAG ATTGTCTATG
BolOX_9g62530.1         GTTCAATTCT TACCACCGGA GCCTTATAAG AG---GTTTC GCTTCTCAAG TCCTTACCCA AGGGAATGAG GTAGGTTTTG CTTCGGAAGT TCCAGCCACG GTGGAGGCAG TCAAGACACC TAACTCAAAG ATTGTCTATG
BolHDEM_C9t59207        TTTCAATTCT TACCACCGGA GCCTTATAAG AG---GTTTA GCTTCTCAAG TCCTTACCCA AGGGAATGAG GTAGGTTTTG CTTCGGAAGT TCCAGCCACG GTGGAGGCAG TCAAGACACC TAACTCAAAG ATTGTCTATG
Bca_C04g19116           TTTCAGTTCT TACCACCGGA GCCTTATAAG AG---GTTTC GCTTCTCAAG TCCTCACCCA AGGGAATGAG GTGGGTTTTG CTTCGGAAGT TCCAGCCACC GTCGAGGCTG TCAAGACACC CAACTCAAAG ATTGTCTATG
BniC2_B02g052520.1      TTTCAGTTCT TATCACAGGA GCCTTATAAG AG---GTTTC GCATCTCAAG TCCTTACCCA AGGGAACGAG GTTGGTTTTG CTTCGGAAGT CCCAGCAACC GTCGAGGCTG TCAAGACACC TAACTCAAAG ATTGTGTATG
BniC2_B02g052620.1      TTTCAGTTCT TATCACCGGA GCCTTATAAG AG---GTTTC GCATCTCAAG TCCTCACCCA AGGGAATGAG GTGGGTTTTG CTTCGGAAGT CCCAGCCACC GTCGAGGCTG TCAAGACACC CAACTCAAAG ATTGTCTATG
BniN100_B02g049090.2    TCTCAGTTCT TACCACCGGA GCCTTATAAG AG---GTTAC GCTTCTCAGG TCCTCGCTCA AGGGAATGAG GTAGGTTTTG CTTCGGAAGT CCCAGCCACC GTCGAGGCTG TCAAGACACC CAACTCAAAG ATTGTGTATG

                        285        295        305        315        325        335        345        355        365        375        385        395        405        415
BolKorso_9g60640.1      ACGACCACAA CCATGAGCGC TACCCACCTG GTGACCCTAG CAAGCGTGCC TTCGCCTATT TCGTCTTGTC TGGCGGGAGG TTTGTTTACG CCTCTGTTCT CCGCCTTCTT GTTCTGAAGC TTATTGTCAG CATGTCCGCA
BolOX_9g62530.1         ACGACCACAA CCATGAGCGT TACCCACCTG GTGACCCTAG CAAGCGTGCC TTCGCCTATT TCGTCCTCTC TGGTGGGAGG TTTGTCTACG CCTCTGTTCT ACGCCTTCTT GTTCTGAAGC TCATTGTCAG CATGTCCGCA
BolHDEM_C9t59207        ACGACCACAA CCATGAGCGC TACCCACCTG GTGACCCTAG CAAGCGTGCC TTCGCCTATT TCGTCTTGTC TGGCGGGAGG TTTGTTTACG CCTCTGTTCT CCGTCTTCTT GTTCTGAAGC TCATTGTCAG CATGTCCGCA
Bca_C04g19116           ACGACCACAA TCATGAGCGT TACCCACCTG GTGACCCTAG CAAGCGTGCC TTCGCCTATT TCGTCCTTTC CGGTGGGAGG TTTGTTTATG CCTCTGTTCT CCGCCTTCTT GTTCTGAAGC TTATTGTCAG CATGTCCGCA
BniC2_B02g052520.1      ACGACCACAA CCATGAGCGT TACCCACCTG GTGATCCTAG CAAACGTGCG TTCGCCTATT TCGTCTTGTC TGGCGGGAGG TTTGTTTATG CCTCTGTTCT CCGCCTTCTT GTTCTGAAGC TCATTGTCAG CATGTCCGCA
BniC2_B02g052620.1      ACGACCACAA TCATGAGCGT TACCCACCTG GTGATCCTAG CAAACGTGCG TTCGCCTATT TCGTCTTGTC TGGCGGGAGG TTTGTTTATG CCTCTGTTCT CCGCCTTCTT GTTCTGAAGC TCATTGTCAG CATGTCCGCA
BniN100_B02g049090.2    ACGACCACAA CCATGAGCGT TACCCGCCTG GTGACCCTAG CAAGCGTGCC TTCGCCTATT TCGTCCTGTC CGGTGGGAGG TTTGTCTACG CCTCTGTTCT CCGCCTTCTT GTTCTGAAGC TCATTGTCAG CATGTCCGCA

                        425        435        445        455        465        475        485        495        505        515        525        535        545        555
BolKorso_9g60640.1      AGTAAAGATG TCCTTGCCCT TGCATCCCTC GAGGTTGACC TCGGTAGCAT CGAACCAGGA ACGACCGTTA CAGTGAAGTG GCGTGGAAAG CCCGTGTTTA TCAGGCGAAG AACCGAAGAT GACATCAAGC TGGCCAATAG
BolOX_9g62530.1         AGTAAAGATG TCCTTGCACT TGCATCCCTC GAGGTTGACC TAGGGAGCAT CGAACCGGGA ACTACTGTGA CAGTGAAGTG GCGTGGAAAG CCTGTCTTCA TCAGGAGAAG GACAGAAGAC GACATCAAGC TGGCCAATAG
BolHDEM_C9t59207        ACTAAAGATG TCCTTGCACT TGCATCCCTC GAGGTTGACC TAGGGAGCAT CGAACCGGGA ACTACTGTGA CAGTGAAGTG GCGTGGAAAG CCTGTCTTCA TCAGGAGAAG GACAGAAGAC GACATCAAGC TGGCCAATAG
Bca_C04g19116           AGTAAAGATG TACTTGCCCT TGCTTCCCTC GAGGTTGACC TCGGTAGCAT CGAACCGGGA ACTACTGTGA CAGTCAAGTG GCGTGGCAAG CCCGTGTTCA TCAGGAGACG GACAGAGGAT GACATCAAGC TGGCCAATAG
BniC2_B02g052520.1      AGTAAAGATG TCCTTGCACT TGCATCCCTC GAGGTTGACC TCGGGAGCAT CGAACCTGGA ACTACTGTGA CAGTGAAGTG GCGTGGAAAG CCCGTGTTCA TCAGGAGAAG GACAGAGGAT GACATCAAGC TGGCCAATAG
BniC2_B02g052620.1      AGTAAAGATG TACTTGCTCT TGCTTCCCTC GAGGTTGAAC TCGGTAGCAT CGAACCGGGA ACTACTGTGA CAGTGAAGTG GCGTGGAAAG CCTGTCTTTA TCAGGAGAAG GACAGAGGAT GACATCAAGC TGGCCAATAG
BniN100_B02g049090.2    AGTAAAGATG TCCTCGCACT TGCATCCCTC GAGGTTGACC TCGGTAGCAT CGAGCCGGGA ACCACTGTGA CAGTGAAGTG GCGTGGAAAG CCCGTGTTCA TCAGGCGAAG AACAGAGGAT GACATCAAGC TGGCCAATAG

                        565        575        585        595        605        615        625        635        645        655        665        675        685        695
BolKorso_9g60640.1      TGTGGATCTT GGTTCTCTGA GAGACCCTCA AGAAGATGCT GTCAGGGTCA AGAATCCGGA ATGGTTAGTG GTGGTTGGTG TCTGCACTCA CTTGGGATGC ATCCCCTTGC CTAACGCTGG TGATTATGGA GGTTGGTTCT
BolOX_9g62530.1         CGTGGATCTT GGATCTCTGA GGGATCCACA AGAAGATGCT GTCAGGGTGA AGAATCCGGA ATGGCTAGTG GTGGTTGGAG TCTGCACTCA CTTGGGATGC ATCCCTTTAC CTAACGCTGG TGATTATGGA GGTTGGTTCT
BolHDEM_C9t59207        CGTGGATCTT GGAACTCTGA GAGACCCGCA AGAAGATGCT GTCAGGGTCA AGAATCCGGA ATGGCTAGTG GTGGTTGGAG TCTGCACTCA CTTGGGATGC ATCCCTTTAC CTAACGCTGG TGATTATGGA GGTTGGTTTT
Bca_C04g19116           TGTGGATCTC GGATCTCTGA GAGACCCACA AGAAGATGCG GTCAGGGTGA AGAATCCGGA ATGGTTAGTG GTGGTTGGAG TCTGCACTCA CTTGGGATGC ATCCCTTTGC CTAATGCTGG TGATTATGGA GGTTGGTTCT
BniC2_B02g052520.1      TGTGGATCTT GGTTCTCTGA GAGACCCACA AGAAGATGCC GTCAGGGTGA AGAATCCGGA ATGGTTAGTG GTGGTTGGAG TCTGCACTCA CTTGGGATGC ATTCCTTTGC CTAATGCGGG TGATTATGGA GGTTGGTTTT
BniC2_B02g052620.1      TGTGGATCTC GGTTCTCTGA GAGACCCGCA AGAAGATGCG GTCAGGGTGA AGAATCCGGA ATGGTTAGTG GTGGTTGGAG TCTGCACTCA CTTGGGATGC ATTCCTTTGC CTAATGCGGG TGATTATGGA GGTTGGTTTT
BniN100_B02g049090.2    TGTGGATCTT GGTTCTCTGA GAGACCCACA AGAAGATGCG GTTAGGGTGA AGAATCCGGA ATGGTTAGTG GTGGTTGGAG TCTGCACTCA CTTGGGATGC ATCCCTTTGC CTAATGCGGG TGATTATGGA GGTTGGTTTT

                        705        715        725        735        745        755        765        775        785        795        805        815
BolKorso_9g60640.1      GCCCCTGTCA CGGATCACAT TACGATATCT CTGGAAGGAT CAGGAAAGGT CCTGCACCGT ACAACCTGGA AGTGCCAACA TACAGCTTCT TGGAAGAGAA CAAGTTACTA ATTGGTTGA
BolOX_9g62530.1         GCCCGTGTCA CGGATCACAT TACGATATCT CTGGAAGGAT CAGGAAAGGT CCTGCACCGT ACAACCTGGA AGTGCCAACA TACAGCTTCT TGGAAGAGAA CAAGTTACTA ATTGGTTGA
BolHDEM_C9t59207        GCCCTTGTCA TGGTTCACAT TATGATATCT CTGGAAGGAT TAGGAAAGGT CCTGCACCAT ACAACTTGGA AGTACCAACC TACAGCTTCT TGGAAGAGAA TAAGTTACTC ATTGGTTGA
Bca_C04g19116           GCCCCTGCCA CGGATCACAT TATGATATCT CTGGAAGGAT CAGGAAAGGT CCAGCTCCGT ACAATCTGGA AGTACCAACA TACAGCTTCT TGGAAGAGAA CAAGTTACTC ATTGGTTGA
BniC2_B02g052520.1      GCCCGTGTCA TGGTTCGCAT TATGATATAT CTGGAAGGAT CAGGAAAGGA CCTGCACCGT ACAACCTGGA AGTACCGACC TACAGCTTCT TGGAAGAGAA CAAGTTACTC ATTGGTTGA
BniC2_B02g052620.1      GTCCGTGTCA TGGTTCGCAT TATGATATAT CTGGAAGGAT CAGGAAAGGA CCTGCACCGT ACAACCTGGA AGTACCGACC TACAGCTTCT TGGAAGAGAA CAAGTTACTC ATTGGTTGA
BniN100_B02g049090.2    GCCCGTGTCA TGGTTCGCAT TATGATATAT CTGGAAGGAT CAGGAAAGGA CCTGCACCGT ACAACCTGGA AGTACCGACC TACAGCTTCT TGGAAGAGAA CAAGTTACTC ATTGGTTAA
```

(E) Clade IB AABB

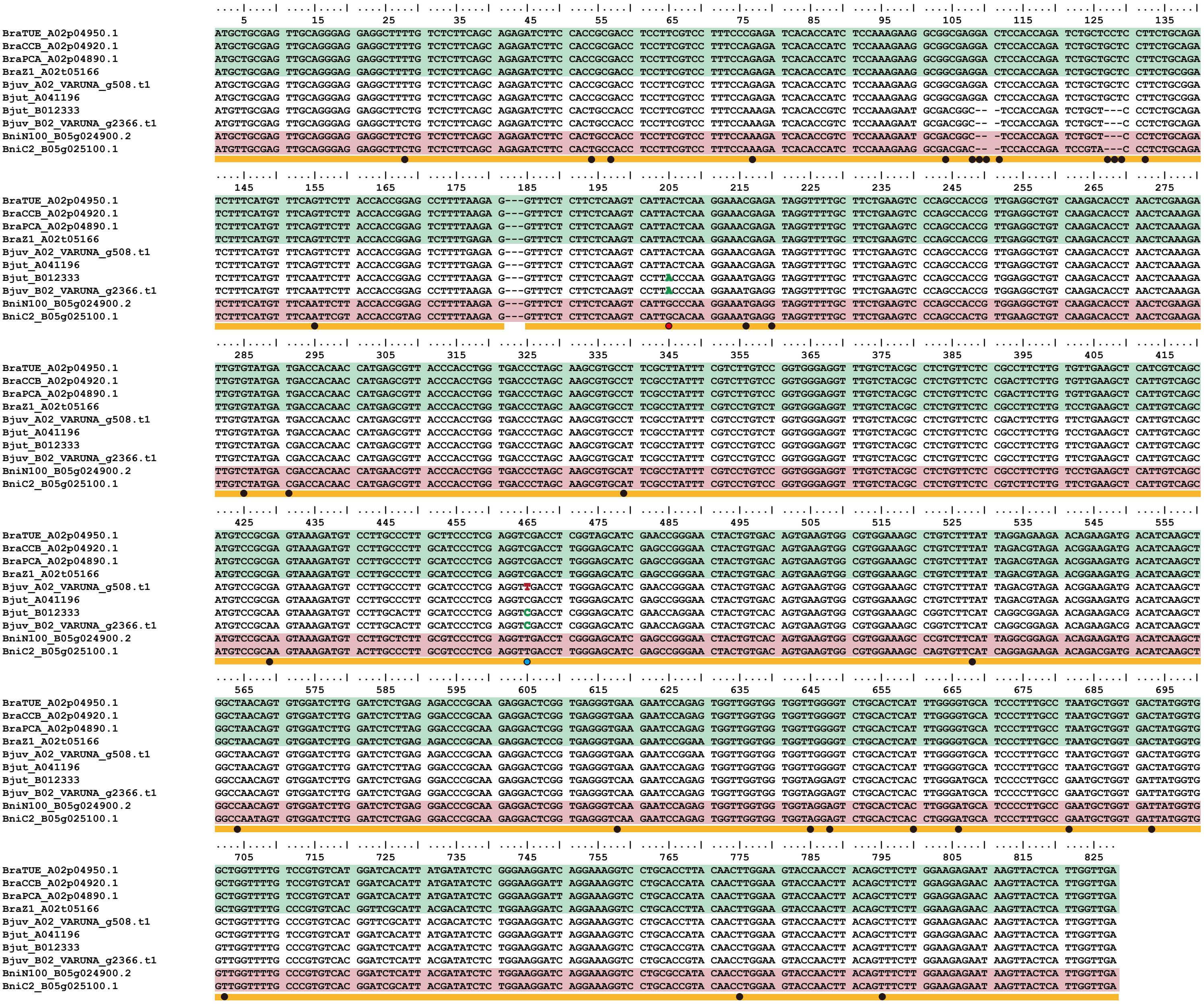

(F) Clade IB BBCC

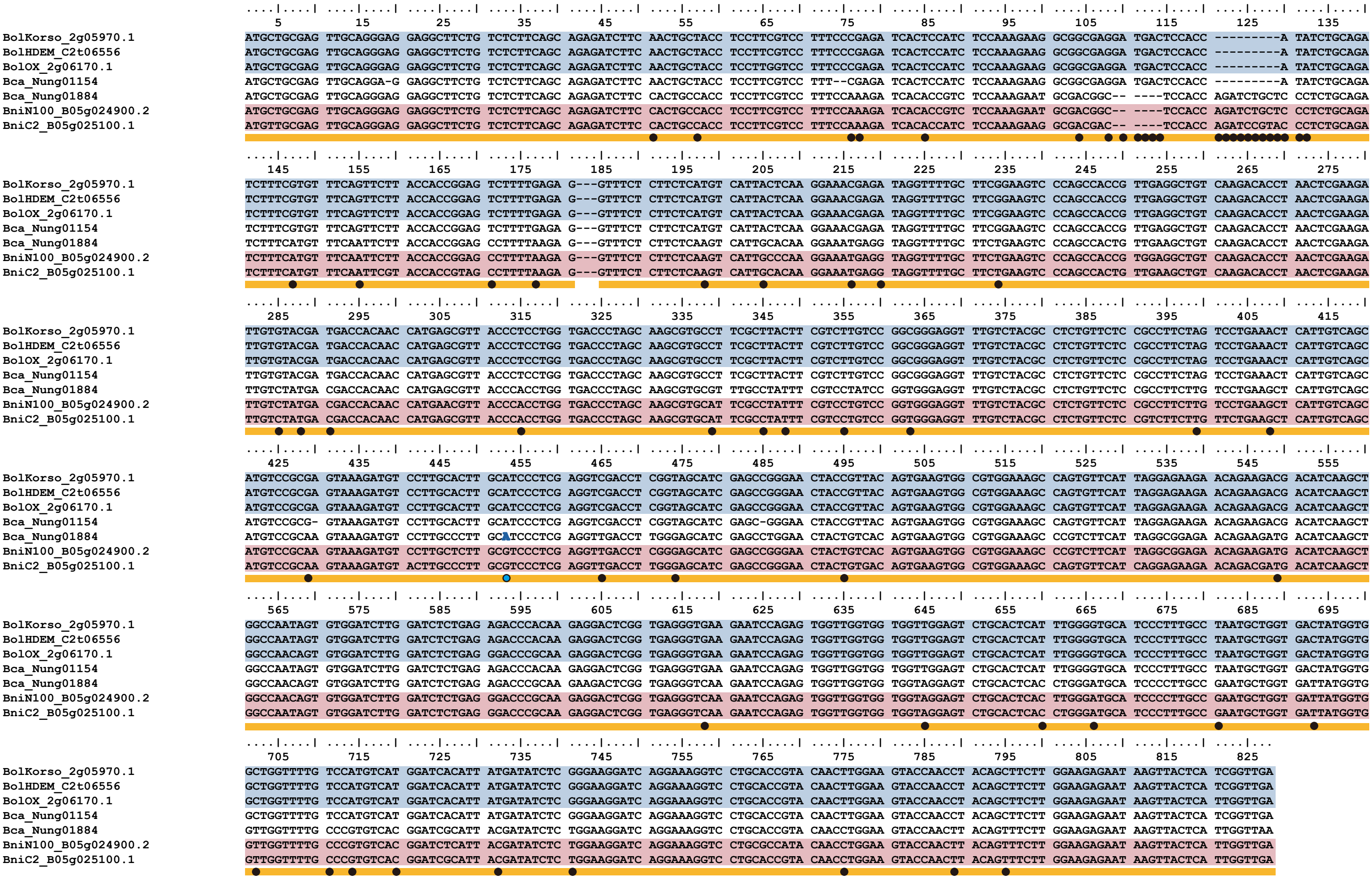

(G) Clade IIA AABB

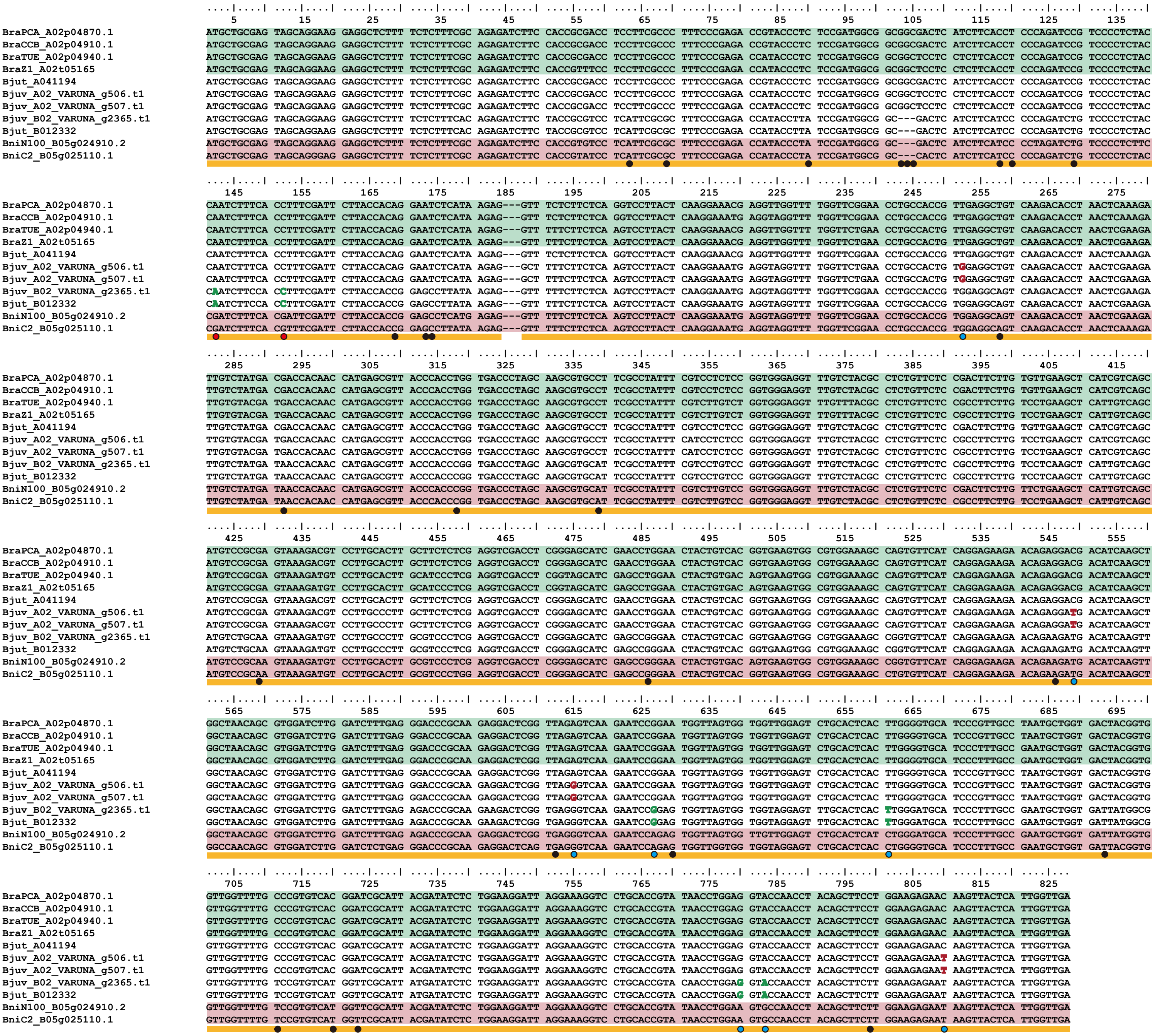

(H) Clade IIA BBCC

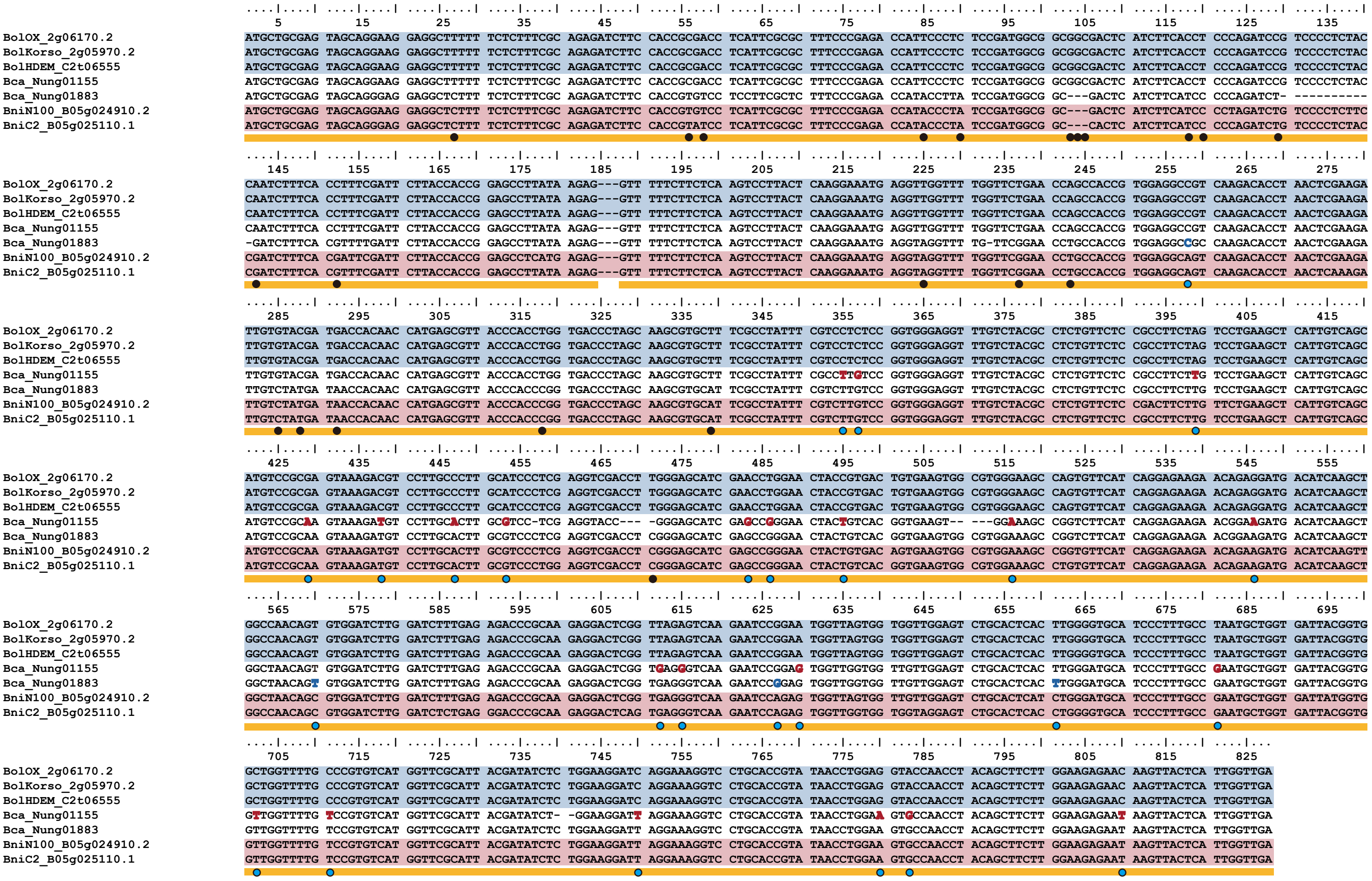

(I) Clade IIB AABB

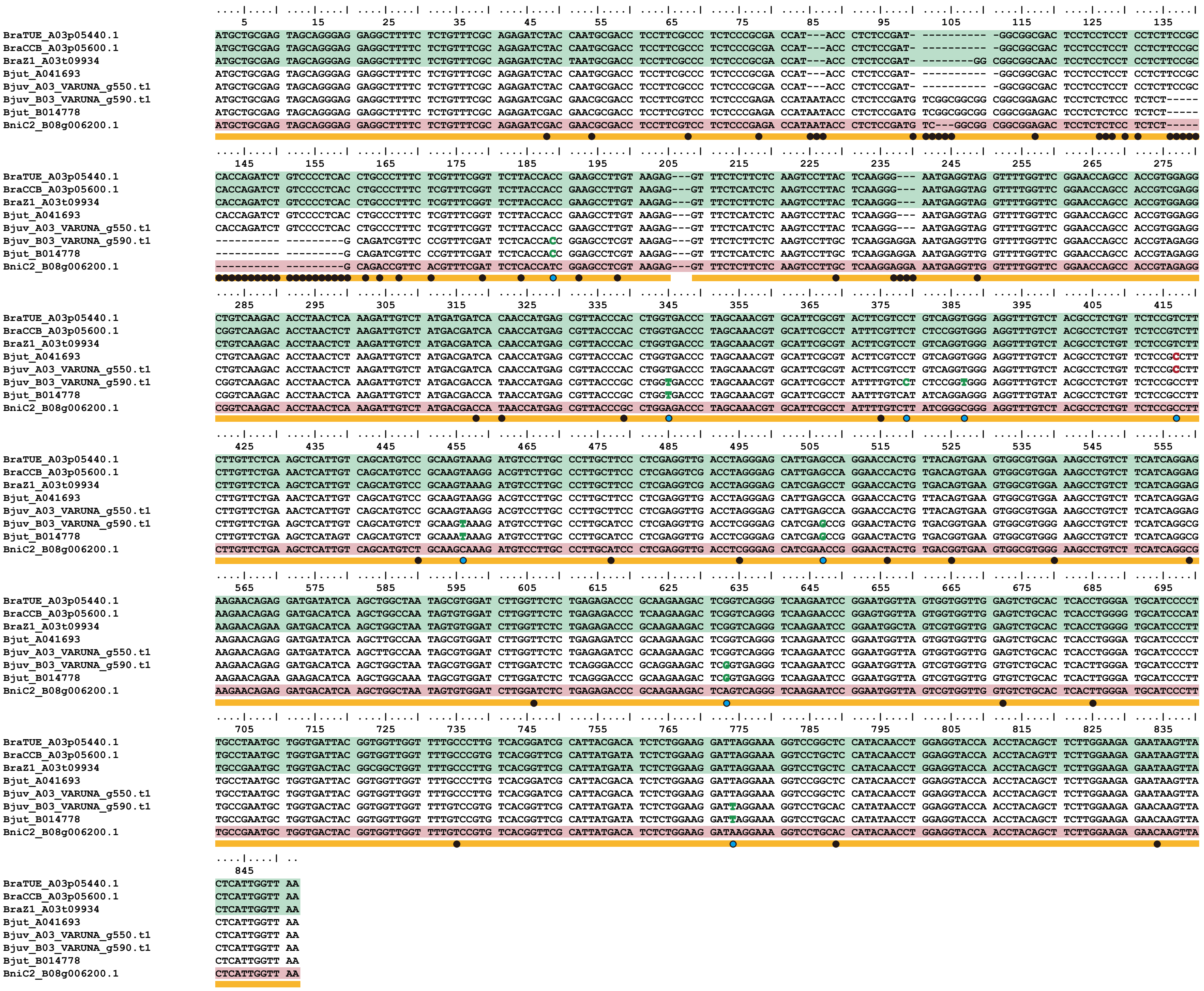

(J) Clade IIB BBCC

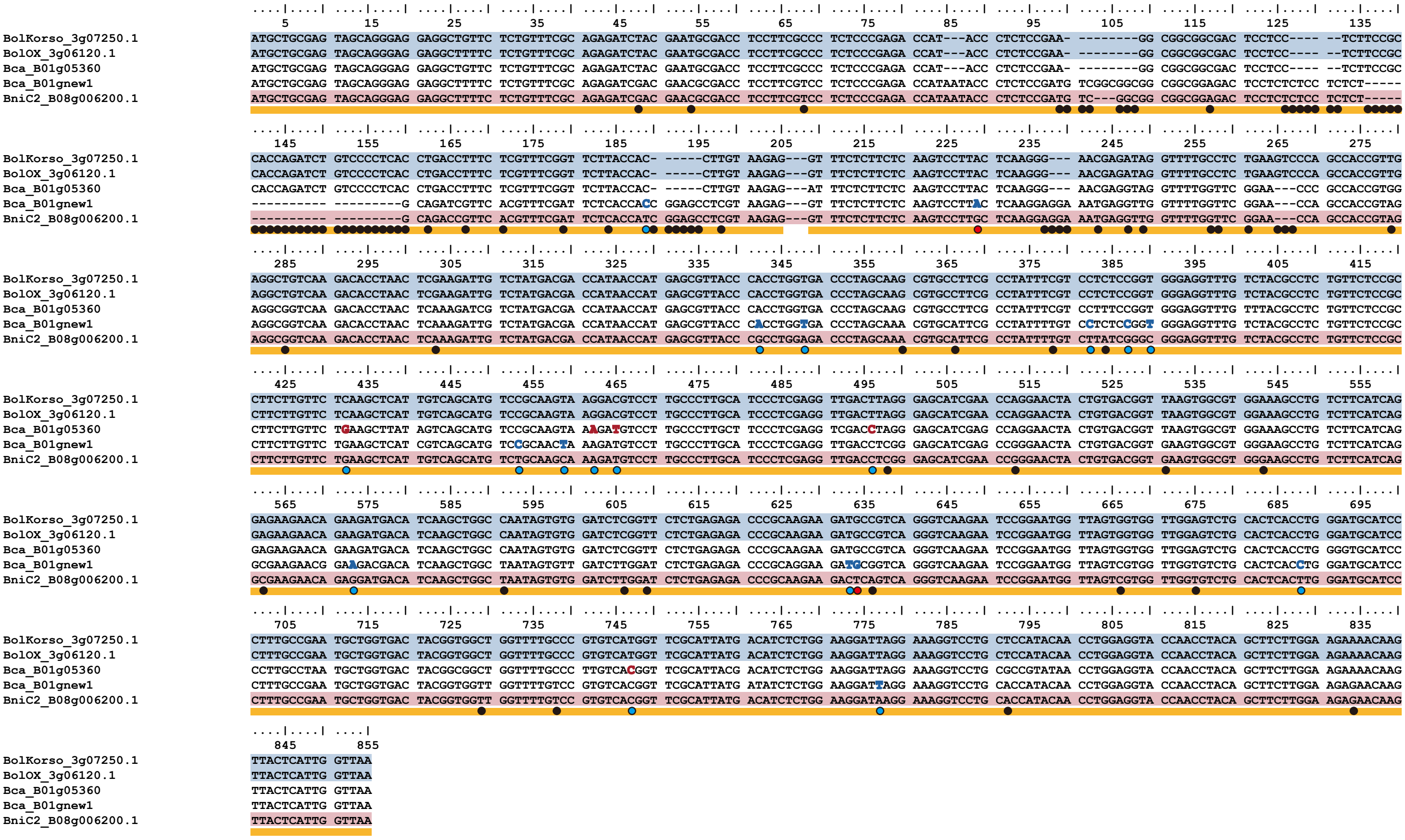

Supplementary Fig S15. Alignment of coding region of genes encoded UCR1 subunit of the mitochondrial complex III in studied genomes/subgenomes. The shade in green indicates *B. rapa* (AA), red indicates *B. nigra* (BB), and blue indicates *B. oleracea* (CC). The black dot indicates genome-specific site, blue dot indicates synonymous inter-genomic conversion, red dot indicates non-synonymous inter-genomic conversion, and grey dot indicates autapomorphy.
