# Supplementary material for: Cytonuclear Interactions and Subgenome Dominance Shape the Evolution of Organelle-Targeted Genes in the Brassica Triangle of U
Source: Mol Biol Evol. 2024 Feb 23;41(3):msae043. doi: 10.1093/molbev/msae043 (PMC10919925; doi:10.1093/molbev/msae043)
Supplement: msae043_Supplementary_Data [file msae043_supplementary_data.zip › Supplementary Figure S16.pdf]

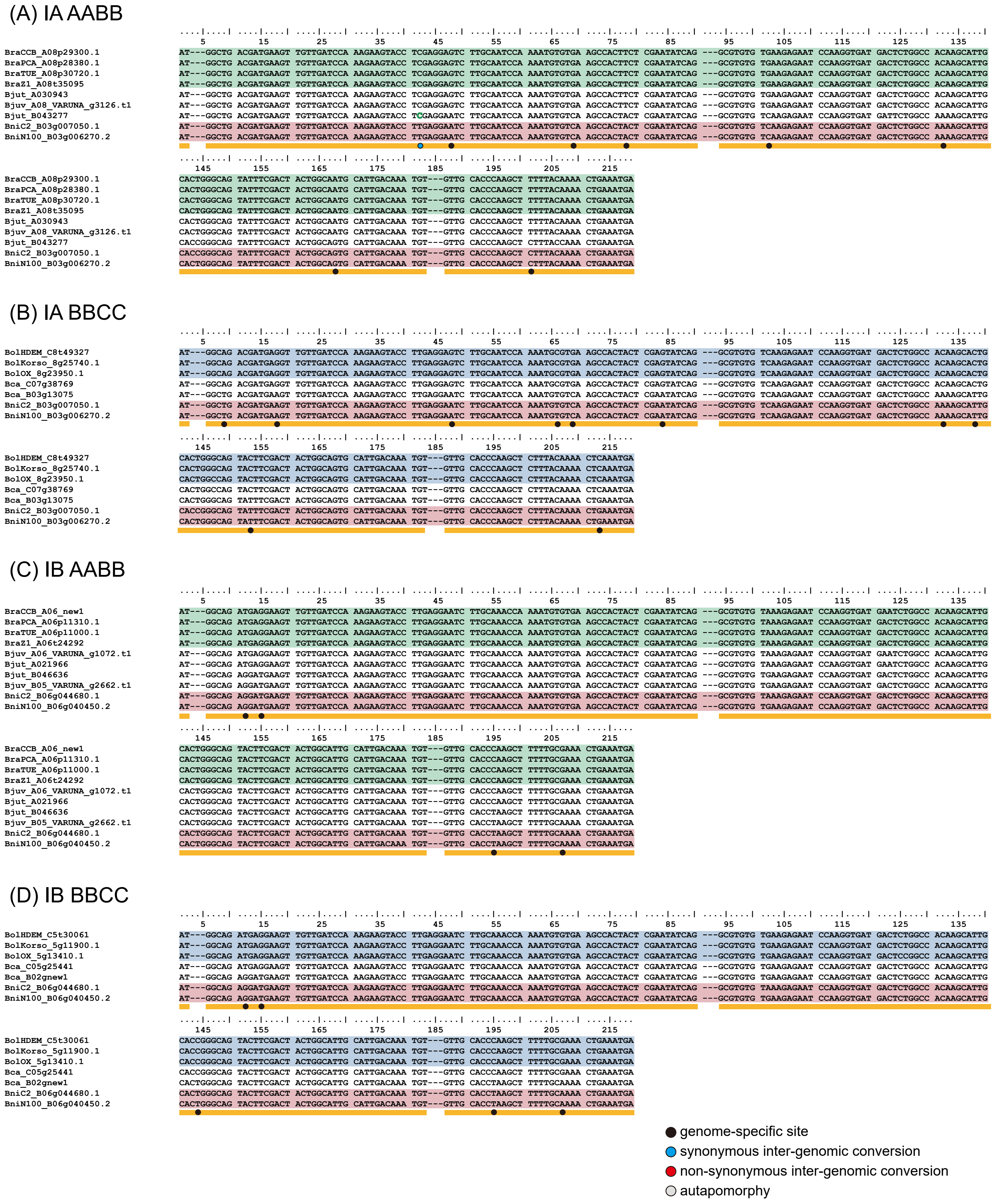
(A) IA AABB
BraCCB_A08p29300.1
BraPCA_A08p28380.1
BraTUE_A08p30720.1
BraZ1_A08t35095
Bjut_A030943
Bjuv_A08_VARUNA_g3126.t1
Bjut_B043277
BniC2_B03g007050.1
BniN100_B03g006270.2
(B) IA BBCC
BolHDEM_C8t49327
BolKorso_8g25740.1
BolOX_8g23950.1
Bca_C07g38769
Bca_B03g13075
BniC2_B03g007050.1
BniN100_B03g006270.2
(C) IB AABB
BraCCB_A06_new1
BraPCA_A06p11310.1
BraTUE_A06p11000.1
BraZ1_A06t24292
Bjuv_A06_VARUNA_g1072.t1
Bjut_A021966
Bjut_B046636
Bjuv_B05_VARUNA_g2662.t1
BniC2_B06g044680.1
BniN100_B06g040450.2
(D) IB BBCC
BolHDEM_C5t30061
BolKorso_5g11900.1
BolOX_5g13410.1
Bca_C05g25441
Bca_B02gnew1
BniC2_B06g044680.1
BniN100_B06g040450.2
genome-specific site
synonymous inter-genomic conversion
non-synonymous inter-genomic conversion
autapomorphy

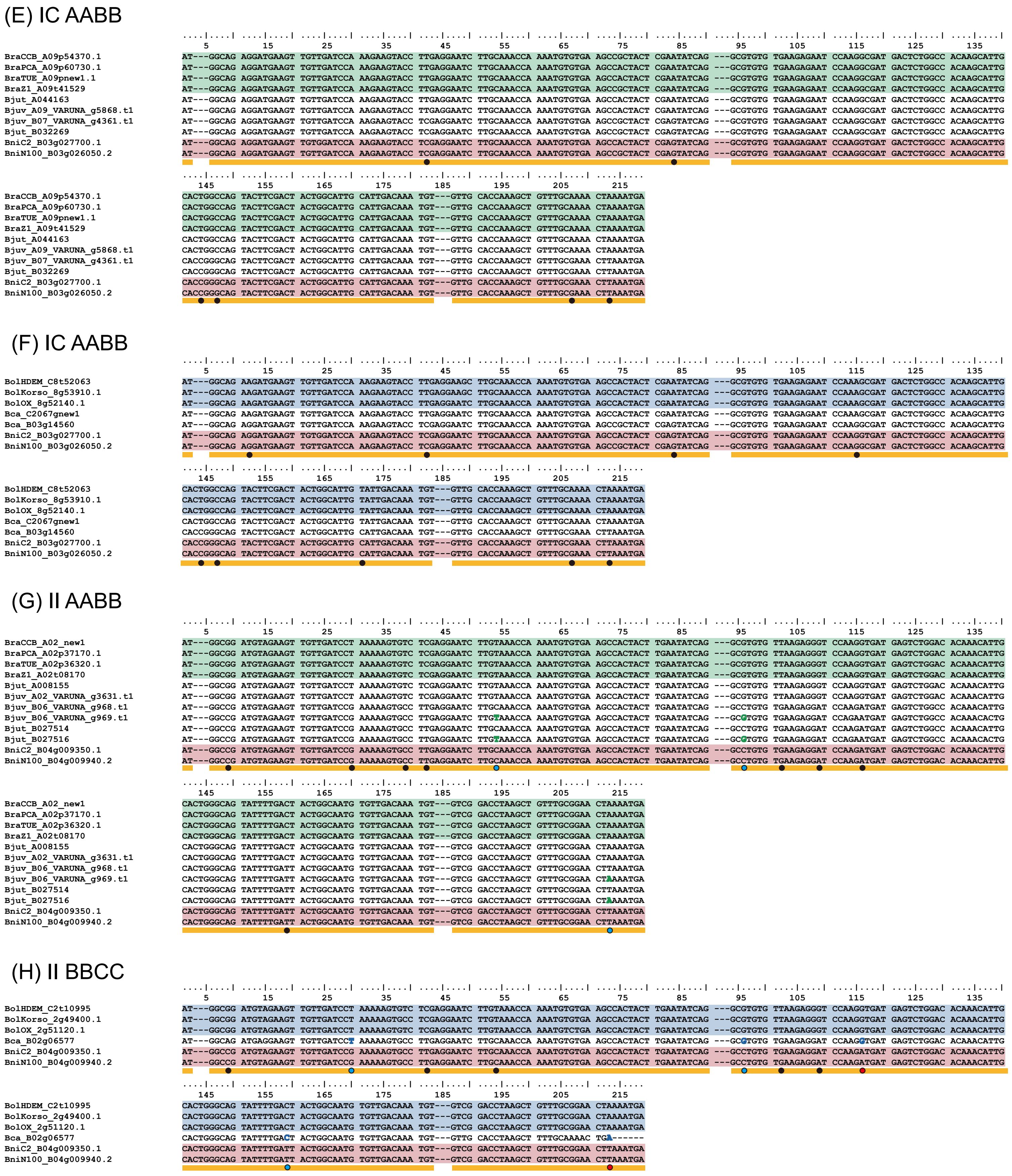

Supplementary Fig S16. Alignment of coding region of genes encoded QCR6 subunit of the mitochondrial complex III in studied genomes/subgenomes. The shade in green indicates *B. rapa* (AA), red indicates *B. nigra* (BB), and blue indicates *B. oleracea* (CC). The black dot indicates genome-specific site, blue dot indicates synonymous inter-genomic conversion, red dot indicates non-synonymous inter-genomic conversion, and grey dot indicates autapomorphy.
