# Supplementary material for: Cytonuclear Interactions and Subgenome Dominance Shape the Evolution of Organelle-Targeted Genes in the Brassica Triangle of U
Source: Mol Biol Evol. 2024 Feb 23;41(3):msae043. doi: 10.1093/molbev/msae043 (PMC10919925; doi:10.1093/molbev/msae043)
Supplement: msae043_Supplementary_Data [file msae043_supplementary_data.zip › Supplementary Figure S19.pdf]

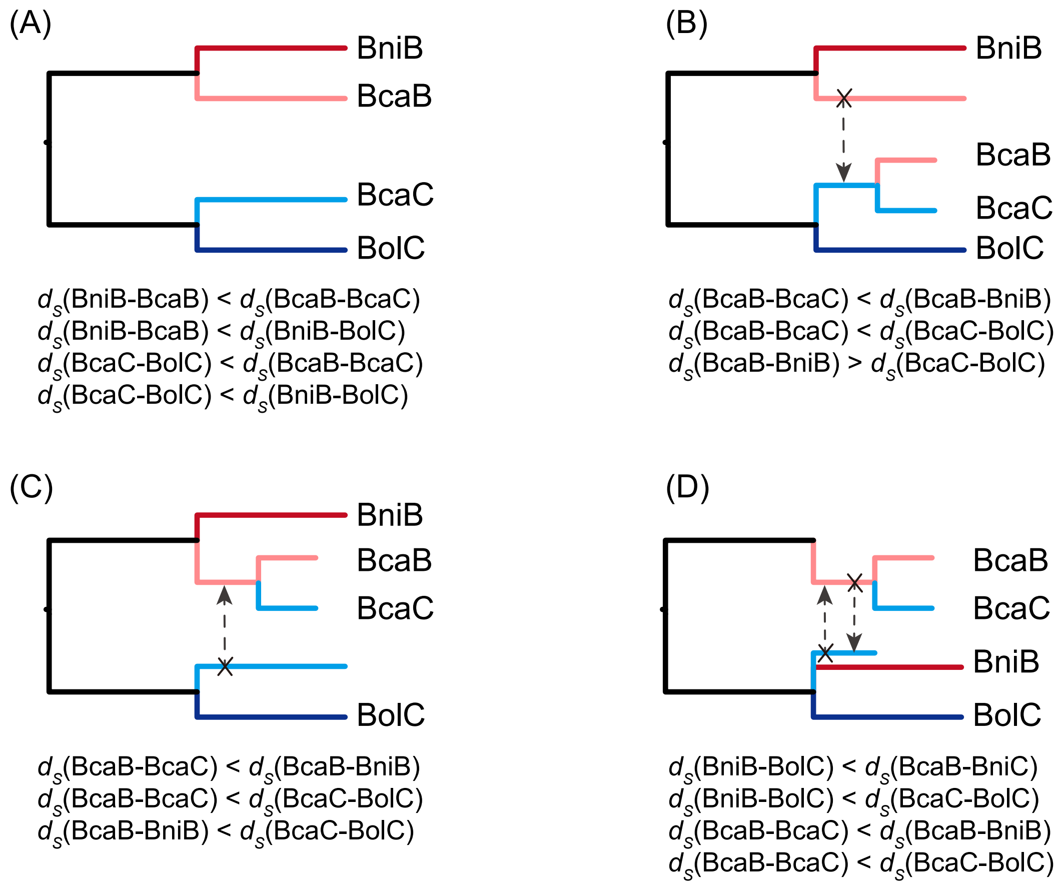

Supplementary Fig S19. Schematic diagram and criteria for detecting gene conversion in single-copy orthologous groups in *B. carinata* (BBCC). (A). The expected phylogeny if no conversion occurs. (B). The sequence of BcaB was replaced by BcaC. (C). The sequence of BcaC was replaced by BcaB. (D). Both of the above conversions occurred.
