# Supplementary material for: Cytonuclear Interactions and Subgenome Dominance Shape the Evolution of Organelle-Targeted Genes in the Brassica Triangle of U
Source: Mol Biol Evol. 2024 Feb 23;41(3):msae043. doi: 10.1093/molbev/msae043 (PMC10919925; doi:10.1093/molbev/msae043)
Supplement: msae043_Supplementary_Data [file msae043_supplementary_data.zip › Supplementary Tables.pdf]

Supplementary Table S1. Gene classification according to CyMIRA in *B. juncea* (AABB) and its diploid parents.

**Functional category**	**Ara**	**AA_PCA**	**AA_CCB**	**AA_TUE**	**AA_Z1**	**BB_N100**	**BB_C2**	**AABB_Tumida_AA**	**AABB_Tumida_BB**	**AABB_Varuna_AA**	**AABB_Varuna_BB**
Other	14344	19521	19763	19130	19637	21243	23375	16404	15504	19423	20309
Dual	435	948	969	893	1007	1056	1134	726	659	940	955
Plastid-targeted (pt_tar)	2408	3788	3881	3788	3820	3991	4323	3225	3012	3638	3757
Plastid-targeted interacting (pt_int)	272	361	363	353	363	367	425	315	270	359	356
Plastid enzyme complexes (pt_com)	119	202	197	193	199	196	229	169	131	194	187
Mitochondria-targeted (mt_tar)	1231	1709	1768	1789	1865	1965	2197	1540	1371	1707	1772
Mitochondria-targeted interacting (mt_int)	464	559	572	575	572	611	716	503	468	560	605
Mitochondria enzyme complexes (mt_com)	146	231	226	229	244	255	286	183	189	232	242

Supplementary Table S2. Gene classification according to CyMIRA in *B. carinata* (BBCC) and its diploid parents.

**Functional category**	**Ara**	**BB_C2**	**BB_N100**	**CC_HDEM**	**CC_Korso**	**CC_OX**	**BBCC_BB**	**BBCC_CC**
Other	14867	22512	20445	20232	19784	19627	14704	18942
Dual	436	896	826	824	827	816	599	745
Plastid-targeted (pt_tar)	2418	3889	3598	3560	3535	3561	2630	3306
Plastid-targeted interacting (pt_int)	273	405	351	364	350	353	254	347
Plastid enzyme complexes (pt_com)	119	209	187	189	177	179	142	195
Mitochondria-targeted (mt_tar)	1246	1883	1704	1746	1645	1656	1219	1576
Mitochondria-targeted interacting (mt_int)	477	681	608	595	565	572	430	586
Mitochondria enzyme complexes (mt_com)	147	261	254	233	227	233	185	229

Supplementary Table S3. Chi-square test for significant difference in retention preference between non-targeted genes and organelle targeted genes in each functional category.

	**AABB_tumida**	**AABB_varuna**	**BBCC**
Dual-targeted (Dual)	0.4791	0.5563	0.5486
Plastid-targeted without interacting (pt_tar)	0.6776	0.6329	0.3951
Plastid-interacted without complexes (pt_int)	0.2600	0.5063	0.5070
Plastid enzyme complexes (pt_com)	0.1010	0.4600	0.6021
Mitochondria-targeted without interacting (mt_tar)	0.1277	0.8518	0.9436
Mitochondria-interacted without complexes (mt_int)	0.8351	0.6033	0.4002
Mitochondria enzyme complexes (mt_com)	0.4247	1.0000	0.7254

Numbers represent *P*-values in Chi-square test.

Supplementary Table S4. Single-copy orthologous groups classification according to CyMIRA in all allotetraploids.

Species	Functional Category	No. of OGs	d_S	d_N	d_N/d_S	ω_{PAT}		ω_{MAT}		**Log2($\omega_{PAT}/\omega_{MAT}$)**	
						tumida	varuna	tumida	varuna	tumida	varuna
AABB	Other	3504	0.7046	0.1381	0.1960	0.2597	0.2695	0.2297	0.2461	0.1769	0.1308
	Dual-targeted	99	0.7663	0.1054	0.1375	0.1360	0.1406	0.1927	0.1679	-0.5033	-0.2563
	Plastid-targeted without interacting	612	0.7041	0.1165	0.1655	0.2149	0.2429	0.2214	0.2215	-0.0426	0.1332
	Plastid-interacted without complexes	55	0.8547	0.1472	0.1722	0.2059	0.2368	0.1534	0.2530	0.4244	-0.0953
	Plastid enzyme complexes	32	0.7804	0.1120	0.1435	0.1709	0.0918	0.0849	0.1494	1.0090	-0.7029
	Mitochondria-targeted without interacting	202	0.7183	0.1139	0.1586	0.3183	0.2835	0.2101	0.2190	0.5992	0.3721
	Mitochondria-interacted without complexes	97	0.8783	0.1740	0.1981	0.4197	0.3615	0.2235	0.2120	0.9094	0.7697
	Mitochondria enzyme complexes	42	0.9035	0.1395	0.1544	0.2273	0.2273	0.1406	0.2273	0.6932	0.0000
BBCC	Other	3911	0.6283	0.1203	0.1915	0.3065		0.2456		0.3192	
	Dual-targeted	141	0.6377	0.0966	0.1515	0.2425		0.1607		0.5935	
	Plastid-targeted without interacting	659	0.6138	0.1006	0.1639	0.2854		0.2195		0.3788	
	Plastid-interacted without complexes	49	0.7093	0.1312	0.1850	0.4063		0.2680		0.6001	
	Plastid enzyme complexes	35	0.6538	0.0844	0.1291	0.0483		0.2845		-2.5576	
	Mitochondria-targeted without interacting	214	0.6205	0.0995	0.1604	0.3131		0.2354		0.4112	
	Mitochondria-interacted without complexes	85	0.7769	0.1507	0.1940	0.3055		0.2518		0.2790	
	Mitochondria enzyme complexes	47	0.7600	0.1034	0.1361	0.2072		0.1110		0.8995	

Supplementary Table S5. Wilcox test for significant difference in ω (d_N/d_S) between non-targeted genes and organelle targeted genes in each functional category.

	**AABB_tumida**	**AABB_varuna**	**BBCC**
Dual-targeted (Dual)	0.2600	0.8800	0.4100
Plastid-targeted without interacting (pt_tar)	0.6300	0.0390	0.7400
Plastid-interacted without complexes (pt_int)	0.7200	0.8100	0.3700
Plastid enzyme complexes (pt_com)	0.3200	0.6400	0.0640
Mitochondria-targeted without interacting (mt_tar)	0.5600	0.7200	0.1900
Mitochondria-interacted without complexes (mt_int)	0.1100	0.7000	0.6800
Mitochondria enzyme complexes (mt_com)	0.6100	0.3700	0.1900

Numbers represent *P*-values in Wilcox test.

Supplementary Table S6. Functional classification of genes that undergo conversion events in *B. juncea* var *tumida* (AABB_tumida).

#OG	Type	AGI Identifier	CyMIRA targeting	CyMIRA Interaction	CyMIRA Interaction Category	CyMIRA Interaction Subcategory
OG0000629.0	A(M)-to-B(P)	AT3G17510	Other	N/A	N/A	N/A
OG0001005.0	A(M)-to-B(P)	AT1G71140	Other	N/A	N/A	N/A
OG0001861.0	A(M)-to-B(P)	AT5G62280	Other	N/A	N/A	N/A
OG0002500.0	A(M)-to-B(P)	AT4G17550	Other	N/A	N/A	N/A
OG0004394.0	A(M)-to-B(P)	AT1G30420	Other	N/A	N/A	N/A
OG0004747.0	A(M)-to-B(P)	AT5G54440	Other	N/A	N/A	N/A
OG0005615.0	A(M)-to-B(P)	AT3G21520	Other	N/A	N/A	N/A
OG0006888.0	A(M)-to-B(P)	AT3G13360	Other	N/A	N/A	N/A
OG0007431.0	A(M)-to-B(P)	AT2G46020	Other	N/A	N/A	N/A
OG0008588.0	A(M)-to-B(P)	AT1G64000	Other	N/A	N/A	N/A
OG0010100.0	A(M)-to-B(P)	AT5G64170	Other	N/A	N/A	N/A
OG0010593.0	A(M)-to-B(P)	AT5G43560	Other	N/A	N/A	N/A
OG0012376.0	A(M)-to-B(P)	AT2G38460	Other	N/A	N/A	N/A
OG0013069.0	A(M)-to-B(P)	AT1G49900	Other	N/A	N/A	N/A
OG0013509.0	A(M)-to-B(P)	AT5G11650	Other	N/A	N/A	N/A
OG0013598.0	A(M)-to-B(P)	AT5G45120	Other	N/A	N/A	N/A
OG0014207.0	A(M)-to-B(P)	AT2G26420	Other	N/A	N/A	N/A
OG0016023.0	A(M)-to-B(P)	AT1G79620	Other	N/A	N/A	N/A
OG0017307.0	A(M)-to-B(P)	AT5G43820	Mitochondria	Mitochondria	PPR	N/A
OG0001608.0	A(M)-to-B(P)	AT5G64040	Plastid	Plastid	Photosynthesis	PSI
OG0014515.0	A(M)-to-B(P)	AT4G14690	Plastid	No	N/A	N/A
OG0015042.0	A(M)-to-B(P)	AT4G32810	Plastid	No	N/A	N/A
OG0016001.0	A(M)-to-B(P)	AT1G61850	Plastid	No	N/A	N/A
OG0016615.0	A(M)-to-B(P)	AT3G28460	Plastid	No	N/A	N/A
OG0000782.0	B(P)-to-A(M)	AT1G66150	Other	N/A	N/A	N/A

OG0001083.0	B(P)-to-A(M)	AT3G03080	Other	N/A	N/A	N/A
OG0001518.0	B(P)-to-A(M)	AT1G60930	Other	N/A	N/A	N/A
OG0001857.0	B(P)-to-A(M)	AT5G11070	Other	N/A	N/A	N/A
OG0004035.0	B(P)-to-A(M)	AT5G64000	Other	N/A	N/A	N/A
OG0004132.0	B(P)-to-A(M)	AT2G41860	Other	N/A	N/A	N/A
OG0004239.1	B(P)-to-A(M)	AT5G45620	Other	N/A	N/A	N/A
OG0004387.0	B(P)-to-A(M)	AT1G71010	Other	N/A	N/A	N/A
OG0005504.0	B(P)-to-A(M)	AT3G17790	Other	N/A	N/A	N/A
OG0005684.0	B(P)-to-A(M)	AT3G17850	Other	N/A	N/A	N/A
OG0006610.0	B(P)-to-A(M)	AT1G10740	Other	N/A	N/A	N/A
OG0006795.0	B(P)-to-A(M)	AT3G23290	Other	N/A	N/A	N/A
OG0006907.0	B(P)-to-A(M)	AT3G59020	Other	N/A	N/A	N/A
OG0011554.0	B(P)-to-A(M)	AT2G26890	Other	N/A	N/A	N/A
OG0011769.0	B(P)-to-A(M)	AT1G20920	Other	N/A	N/A	N/A
OG0012093.0	B(P)-to-A(M)	AT5G66100	Other	N/A	N/A	N/A
OG0013108.0	B(P)-to-A(M)	AT1G59510	Other	N/A	N/A	N/A
OG0013616.0	B(P)-to-A(M)	AT5G65740	Other	N/A	N/A	N/A
OG0014269.0	B(P)-to-A(M)	AT2G26600	Other	N/A	N/A	N/A
OG0014668.0	B(P)-to-A(M)	AT4G26790	Other	N/A	N/A	N/A
OG0014725.0	B(P)-to-A(M)	AT4G15800	Other	N/A	N/A	N/A
OG0015367.0	B(P)-to-A(M)	AT1G04390	Other	N/A	N/A	N/A
OG0016196.0	B(P)-to-A(M)	AT3G15080	Other	N/A	N/A	N/A
OG0016419.0	B(P)-to-A(M)	AT3G20740	Other	N/A	N/A	N/A
OG0016675.0	B(P)-to-A(M)	AT3G56000	Other	N/A	N/A	N/A
OG0016764.0	B(P)-to-A(M)	AT5G60450	Other	N/A	N/A	N/A
OG0016990.0	B(P)-to-A(M)	AT5G01700	Other	N/A	N/A	N/A
OG0016995.0	B(P)-to-A(M)	AT5G11040	Other	N/A	N/A	N/A
OG0017197.0	B(P)-to-A(M)	AT5G07500	Other	N/A	N/A	N/A

OG0017300.0	B(P)-to-A(M)	AT5G38630	Other	N/A	N/A	N/A
OG0017442.0	B(P)-to-A(M)	AT5G42440	Other	N/A	N/A	N/A
OG0001158.0	B(P)-to-A(M)	AT3G27280	Dual	No	N/A	N/A
OG0001809.0	B(P)-to-A(M)	AT3G58610	Dual	No	N/A	N/A
OG0010033.0	B(P)-to-A(M)	AT5G23890	Dual	No	N/A	N/A
OG0015843.0	B(P)-to-A(M)	AT1G06710	Mitochondria	Mitochondria	PPR	N/A
OG0017136.0	B(P)-to-A(M)	AT5G16860	Mitochondria	Mitochondria	PPR	N/A
OG0016891.0	B(P)-to-A(M)	AT5G63420	Plastid	Plastid	Transcription and Transcript Maturation	Transcript End Processing
OG0011839.0	B(P)-to-A(M)	AT1G15700	Plastid	No	N/A	N/A
OG0014391.0	B(P)-to-A(M)	AT2G26610	Plastid	No	N/A	N/A
OG0014812.0	B(P)-to-A(M)	AT4G17540	Plastid	No	N/A	N/A
OG0014857.0	B(P)-to-A(M)	AT4G34190	Plastid	No	N/A	N/A
OG0015020.0	B(P)-to-A(M)	AT4G18740	Plastid	No	N/A	N/A

A(M)-to-B(P) means the sequence of B was replaced by A, while B(P)-to-A(M) means the sequence of A was replaced by B.

Supplementary Table S7. Functional classification of genes that undergo conversion events in *B. juncea* var *varuna* (AABB_varuna).

#OG	Type	AGI Identifier	CyMIRA targeting	CyMIRA Interaction	CyMIRA Interaction Category	CyMIRA Interaction Subcategory
OG0005870.0	A(M)-to-B(P)	AT5G57655	Other	N/A	N/A	N/A
OG0007986.0	A(M)-to-B(P)	AT3G57290	Other	N/A	N/A	N/A
OG0004638.1	A(M)-to-B(P)	AT5G09770	Mitochondria	Mitochondria	Mitoribosome	Small Subunit
OG0008805.0	A(M)-to-B(P)	AT5G16660	Plastid	No	N/A	N/A
OG0001158.0	B(P)-to-A(M)	AT3G27280	Dual	No	N/A	N/A
OG0005172.0	B(P)-to-A(M)	AT1G71865	Plastid	No	N/A	N/A

A(M)-to-B(P) means the sequence of B was replaced by A, while B(P)-to-A(M) means the sequence of A was replaced by B.

Supplementary Table S8. Functional classification of genes that undergo gene conversion events in *B. carinata* (BBCC).

#OG	Type	AGI Identifier	CyMIRA targeting	CyMIRA Interaction	CyMIRA Interaction Category	CyMIRA Interaction Subcategory
OG0000985.1	B(M)-to-C(P)	AT1G50360	Other	N/A	N/A	N/A
OG0001751.0	B(M)-to-C(P)	AT3G17440	Other	N/A	N/A	N/A
OG0002079.0	B(M)-to-C(P)	AT1G62920	Other	N/A	N/A	N/A
OG0002365.0	B(M)-to-C(P)	AT4G25550	Other	N/A	N/A	N/A
OG0002794.0	B(M)-to-C(P)	AT4G34850	Other	N/A	N/A	N/A
OG0003288.0	B(M)-to-C(P)	AT1G73730	Other	N/A	N/A	N/A
OG0003626.1	B(M)-to-C(P)	AT2G39518	Other	N/A	N/A	N/A
OG0004417.0	B(M)-to-C(P)	AT4G28600	Other	N/A	N/A	N/A
OG0004455.0	B(M)-to-C(P)	AT5G10370	Other	N/A	N/A	N/A
OG0004718.0	B(M)-to-C(P)	AT1G69330	Other	N/A	N/A	N/A
OG0006027.0	B(M)-to-C(P)	AT3G23290	Other	N/A	N/A	N/A
OG0006028.0	B(M)-to-C(P)	AT3G16130	Other	N/A	N/A	N/A
OG0006121.0	B(M)-to-C(P)	AT3G53150	Other	N/A	N/A	N/A
OG0006183.0	B(M)-to-C(P)	AT3G15430	Other	N/A	N/A	N/A
OG0006299.0	B(M)-to-C(P)	AT5G64360	Other	N/A	N/A	N/A
OG0007077.0	B(M)-to-C(P)	AT4G11380	Other	N/A	N/A	N/A
OG0007336.0	B(M)-to-C(P)	AT1G63090	Other	N/A	N/A	N/A
OG0007808.0	B(M)-to-C(P)	AT5G67580	Other	N/A	N/A	N/A
OG0008288.0	B(M)-to-C(P)	AT2G22510	Other	N/A	N/A	N/A
OG0008455.0	B(M)-to-C(P)	AT4G16630	Other	N/A	N/A	N/A
OG0008572.0	B(M)-to-C(P)	AT1G21480	Other	N/A	N/A	N/A
OG0008654.0	B(M)-to-C(P)	AT1G04660	Other	N/A	N/A	N/A
OG0008809.0	B(M)-to-C(P)	AT1G11090	Other	N/A	N/A	N/A
OG0008913.0	B(M)-to-C(P)	AT3G19290	Other	N/A	N/A	N/A
OG0010194.0	B(M)-to-C(P)	AT3G23175	Other	N/A	N/A	N/A

OG0010261.0	B(M)-to-C(P)	AT5G25800	Other	N/A	N/A	N/A
OG0010282.0	B(M)-to-C(P)	AT5G01230	Other	N/A	N/A	N/A
OG0012175.0	B(M)-to-C(P)	AT2G45520	Other	N/A	N/A	N/A
OG0012333.0	B(M)-to-C(P)	AT4G29260	Other	N/A	N/A	N/A
OG0012538.0	B(M)-to-C(P)	AT1G02890	Other	N/A	N/A	N/A
OG0013536.0	B(M)-to-C(P)	AT2G21250	Other	N/A	N/A	N/A
OG0013570.0	B(M)-to-C(P)	AT2G30380	Other	N/A	N/A	N/A
OG0006885.0	B(M)-to-C(P)	AT2G40090	Mitochondria	No	N/A	N/A
OG0008764.0	B(M)-to-C(P)	AT1G61570	Mitochondria	No	N/A	N/A
OG0009276.0	B(M)-to-C(P)	AT5G58970	Mitochondria	No	N/A	N/A
OG0010051.0	B(M)-to-C(P)	AT1G07615	Mitochondria	No	N/A	N/A
OG0012937.0	B(M)-to-C(P)	AT5G24165	Mitochondria	No	N/A	N/A
OG0016888.0	B(M)-to-C(P)	AT1G19240	Mitochondria	No	N/A	N/A
OG0007584.0	B(M)-to-C(P)	AT3G57050	Plastid	No	N/A	N/A
OG0000298.0	C(P)-to-B(M)	AT2G03760	Other	N/A	N/A	N/A
OG0000735.1	C(P)-to-B(M)	AT1G79570	Other	N/A	N/A	N/A
OG0000867.0	C(P)-to-B(M)	AT2G33240	Other	N/A	N/A	N/A
OG0001091.0	C(P)-to-B(M)	AT5G43690	Other	N/A	N/A	N/A
OG0002903.0	C(P)-to-B(M)	AT3G19760	Other	N/A	N/A	N/A
OG0003240.0	C(P)-to-B(M)	AT3G19450	Other	N/A	N/A	N/A
OG0003298.0	C(P)-to-B(M)	AT1G75030	Other	N/A	N/A	N/A
OG0005053.0	C(P)-to-B(M)	AT5G25830	Other	N/A	N/A	N/A
OG0005603.0	C(P)-to-B(M)	AT4G14650	Other	N/A	N/A	N/A
OG0005865.0	C(P)-to-B(M)	AT1G47380	Other	N/A	N/A	N/A
OG0005866.0	C(P)-to-B(M)	AT1G70420	Other	N/A	N/A	N/A
OG0006019.0	C(P)-to-B(M)	AT3G55240	Other	N/A	N/A	N/A
OG0006395.0	C(P)-to-B(M)	AT5G25090	Other	N/A	N/A	N/A
OG0007204.0	C(P)-to-B(M)	AT1G51360	Other	N/A	N/A	N/A

OG0007253.0	C(P)-to-B(M)	AT1G62320	Other	N/A	N/A	N/A
OG0007261.0	C(P)-to-B(M)	AT1G29120	Other	N/A	N/A	N/A
OG0007719.0	C(P)-to-B(M)	AT3G52100	Other	N/A	N/A	N/A
OG0007723.0	C(P)-to-B(M)	AT5G46240	Other	N/A	N/A	N/A
OG0007913.0	C(P)-to-B(M)	AT5G52210	Other	N/A	N/A	N/A
OG0008201.0	C(P)-to-B(M)	AT2G31865	Other	N/A	N/A	N/A
OG0008420.0	C(P)-to-B(M)	AT4G36930	Other	N/A	N/A	N/A
OG0008675.0	C(P)-to-B(M)	AT1G71410	Other	N/A	N/A	N/A
OG0008706.0	C(P)-to-B(M)	AT1G12360	Other	N/A	N/A	N/A
OG0008891.0	C(P)-to-B(M)	AT5G26720	Other	N/A	N/A	N/A
OG0008916.0	C(P)-to-B(M)	AT3G19850	Other	N/A	N/A	N/A
OG0009221.0	C(P)-to-B(M)	AT5G23575	Other	N/A	N/A	N/A
OG0009526.0	C(P)-to-B(M)	AT2G35650	Other	N/A	N/A	N/A
OG0009915.0	C(P)-to-B(M)	AT1G26945	Other	N/A	N/A	N/A
OG0010158.0	C(P)-to-B(M)	AT3G07860	Other	N/A	N/A	N/A
OG0010225.0	C(P)-to-B(M)	AT3G15450	Other	N/A	N/A	N/A
OG0010487.0	C(P)-to-B(M)	AT5G45400	Other	N/A	N/A	N/A
OG0010879.0	C(P)-to-B(M)	AT2G23140	Other	N/A	N/A	N/A
OG0011433.0	C(P)-to-B(M)	AT3G47190	Other	N/A	N/A	N/A
OG0011622.0	C(P)-to-B(M)	AT5G11280	Other	N/A	N/A	N/A
OG0015961.0	C(P)-to-B(M)	AT2G32770	Other	N/A	N/A	N/A
OG0017959.0	C(P)-to-B(M)	AT1G55180	Other	N/A	N/A	N/A
OG0018372.0	C(P)-to-B(M)	AT3G62970	Other	N/A	N/A	N/A
OG0019312.0	C(P)-to-B(M)	AT5G42120	Other	N/A	N/A	N/A
OG0006846.0	C(P)-to-B(M)	AT2G25840	Dual	Dual	tRNA Aminoacylation	
OG0018497.0	C(P)-to-B(M)	AT3G08000	Dual	No	N/A	N/A
OG0019176.0	C(P)-to-B(M)	AT5G50340	Dual	Dual	DNA-RRR	
OG0004003.0	C(P)-to-B(M)	AT3G27930	Mitochondria	No	N/A	N/A

OG0012811.0	C(P)-to-B(M)	AT5G27290	Plastid	No	N/A	N/A
OG0013710.0	C(P)-to-B(M)	AT2G43940	Plastid	No	N/A	N/A
OG0019017.0	C(P)-to-B(M)	AT5G10460	Plastid	No	N/A	N/A

B(M)-to-C(P) means the sequence of C was replaced by B, while C(P)-to-B(M) means the sequence of B was replaced by C.

Supplementary Table S9. Chi-square test for significant difference in expression between non-targeted genes and organelle targeted genes in each functional category.

	**AABB_tumida**	**AABB_varuna**	**BBCC**
Dual-targeted (Dual)	0.6763	0.9565	0.4807
Plastid-targeted without interacting (pt_tar)	0.1248	0.5163	0.3494
Plastid-interacted without complexes (pt_int)	1.0000	0.2789	0.2863
Plastid enzyme complexes (pt_com)	0.0131	1.0000	0.1902
Mitochondria-targeted without interacting (mt_tar)	0.4729	0.8846	0.5990
Mitochondria-interacted without complexes (mt_int)	0.5878	0.9328	0.5595
Mitochondria enzyme complexes (mt_com)	0.0861	0.0998	0.8983

Numbers represent *P*-values in Chi-square test.

Supplementary Table S10. Numbers of nuclear genes encoded RuBisCo in studied genomes/subgenomes.

Species	Subgenome Type	Clade IA-1	Clade IA-2	Clade IB	Clade II	Total
Ara	NA		3		1	4
AA_Z1	A	1	1	3	3	8
AA_CCB	A	1	1	3	3	8
AA_PCA	A	1	1	3	3	8
AA_TUE	A	1	1	3	3	8
BB_N100	B	1	2	3	1	7
BB_C2	B	1	2	4	1	8
CC_HDEM	C	1	1	3	3	8
CC_Korso	C	1	1	2	3	7
CC_OX	C	1	2	3	4	10
AABB_tumida	A	2	1	3	3	9
AABB_tumida	B	1	2	3	1	7
AABB_varuna	A	1	1	3	3	8
AABB_varuna	B	1	2	3	1	7
BBCC	B	1	2	3	1	7
BBCC	C	1	1	3	3	8

Supplementary Table S11. Numbers of nuclear genes encoded mitochondrial complex III in studied genomes/subgenomes.

Species	Subgenome Type	*At1g51980* *At3g16480*	*At3g02090*	*At4g32470* *At5g25450*	*At2g40765*	*At3g52730*	*At3g10860* *At5g05370*	*At5g13430* *At5g13440*	*At1g15120* *At2g01090*	*At5g40810* *At3g27240*
**Subunit name**	**NA**	**MPP-α**	**MPP-β**	**QCR7**	**QCR10**	**QCR9**	**QCR8**	**UCR1**	**QCR6**	**CYC1**
Ara	NA	2	1	2	1	1	2	2	2	2
AA_Z1	A	4	2	3	2	2	3	5	4	3
AA_CCB	A	4	2	3	0	2	4	5	4	3
AA_PCA	A	4	2	3	0	2	4	4	4	3
AA_TUE	A	4	2	3	0	2	4	5	4	3
BB_N100	B	5	2	3	2	3	4	7	4	2
BB_C2	B	6	2	3	3	4	4	10	4	3
CC_HDEM	C	4	2	3	4	2	3	4	4	3
CC_Korso	C	4	2	3	2	2	3	5	4	3
CC_OX	C	4	2	3	4	2	3	5	4	3
AABB_tumida	A	3	2	3	1	1	3	4	4	4
AABB_tumida	B	4	2	3	0	4	4	7	5	4
AABB_varuna	A	4	2	3	2	2	4	6	4	3
AABB_varuna	B	4	2	3	2	3	4	9	5	4
BBCC	B	4	1	1	1	3	3	8	4	3
BBCC	C	4	2	3	2	2	2	2	3	1

Supplementary Table S12. Variants and conversions in genes encoded the mitochondrial complex III in all allotetraploids and its diploid parents.

gene	subunit	clade	A-B genome-specific SNPs	Bjut (AABB)				Bjuv (AABB)				C-B genome-specific SNPs	Bca (BBCC)			
				A (M)-to-B (P)		B (P)-to-A (M)		A (M)-to-B (P)		B (P)-to-A (M)			B (M)-to-C (P)		C (P)-to-B (M)	
				S	N	S	N	S	N	S	N		S	N	S	N
cob			2	NA	NA	NA	NA	NA	NA	NA	NA	2	NA	NA	NA	NA
At1g51980_At3g16480	MPP-α	IA	62	6	1	2	1	6	1	2	2	59	2	0	0	0
		IB	52	0	0	0	0	0	0	0	0	69	6	9	0	0
		IC	56	0	0	1	0	0	1	0	0	67	0	0	0	1
		II	59	1	1	1	0	1	1	1	0	57	0	0	0	0
		sum	229	7	2	4	1	7	3	3	2	252	8	9	0	1
At3g02090	MPP-β	I	112	2	0	2	2	2	0	1	0	110	2	0	5	0
		II	82	4	0	0	0	4	0	0	0	105	6	2	0	0
		sum	194	6	0	2	2	6	0	1	0	215	8	2	5	0
At4g32470_At5g25450	QCR7	IA	14	0	0	0	0	0	0	0	0	15	0	0	0	0
		II	21	0	0	0	0	0	0	0	0	25	0	0	1	0
		sum	35	0	0	0	0	0	0	0	0	40	0	0	1	0
At2g40765	QCR10		NA	NA	NA	NA	NA	NA	NA	NA	NA	NA	NA	NA	NA	NA
At3g52730	QCR9	I	4	0	0	0	0	0	0	0	0	4	0	0	0	0
		II	4	0	0	0	0	0	0	0	0	5	0	0	0	0
		sum	8	0	0	0	0	0	0	0	0	9	0	0	0	0
At3g10860_At5g05370	QCR8	I	NA	NA	NA	NA	NA	NA	NA	NA	NA	NA	NA	NA	NA	NA
		IIA	9	1	0	0	0	3	2	1	0	8	1	0	0	0
		IIB	10	0	1	0	0	3	2	0	0	NA	NA	NA	NA	NA
		sum	19	1	1	0	0	6	4	1	0	8	1	0	2	0
At5g13430_At5g13440	UCR1	IA1	38	5	0	0	0	8	0	0	0	30	0	0	4	0

		IA2	22	5	1	0	1	4	0	0	1	18	0	0	6	0
		IB	33	1	1	0	0	1	1	1	0	65	0	0	1	0
		IIA	36	4	2	0	0	4	2	3	0	48	22	0	4	0
		IIB	74	6	0	1	0	8	0	1	0	98	5	0	12	2
		sum	203	21	4	1	1	25	3	5	1	259	27	0	27	2
At1g15120_At2g01090	QCR6	IA	8	1	0	0	0	0	0	0	0	10	0	0	0	0
		IB	4	0	0	0	0	0	0	0	0	5	0	0	0	0
		IC	6	0	0	0	0	0	0	0	0	9	0	0	0	0
		II	11	3	0	0	0	3	0	0	0	10	0	0	3	1
		sum	29	4	0	0	0	3	0	0	0	34	0	0	3	1
At3g27240_At5g40810	CYC1	IA	23	0	0	0	0	0	0	0	0	28	0	0	2	0
		IB	NA	NA	NA	NA	NA	NA	NA	NA	NA	NA	NA	NA	NA	NA
		IC	22	1	0	0	0	1	0	1	0	NA	NA	NA	NA	NA
		II	NA	NA	NA	NA	NA	NA	NA	NA	NA	NA	NA	NA	NA	NA
		sum	45	1	0	0	0	1	0	1	0	28	0	0	2	0
**Total**			**764**	**40**	**7**	**7**	**4**	**48**	**10**	**11**	**3**	**847**	**44**	**11**	**38**	**4**

Supplementary Table S13 Data information of *Brassica* and *Arabidopsis* used in this study.

Species	Genome Type	Accession	Code	Accession number	Reference
Arabidopsis	-		Ara	TAIR10	Cheng et al. (2017)
Brassica rapa	AA	Z1	AA_Z1	PRJNA730930	Cai et al. (2021)
		CCB	AA_CCB	PRJNA730930	Cai et al. (2021)
		PCA	AA_PCA	PRJNA730930	Cai et al. (2021)
		TUE	AA_TUE	PRJNA730930	Cai et al. (2021)
Brassica nigra	BB	N100	BB_N100	PRJNA516907	Perumal et al. (2020)
		C2	BB_C2	PRJNA516907	Perumal et al. (2020)
Brassica oleracea	CC	HDEM	CC_HDEM	PRJEB26621	Belser et al. (2018)
		Korso	CC_Korso	PRJNA546441	Guo et al. (2021)
		OX-heart	CC_OX	PRJNA548819	Guo et al. (2021)
Brassica juncea	AABB	tumida	AABB_Tumida	PRJNA285130	Yang et al. (2016)
		varuna	AABB_Varuna	PRJNA550308	Paritosh et al. (2020)
Brassica napus	AACC	ZS11	AACC_ZS11	PRJNA394926	Sun et al. (2017)
		No2127	AACC_No2127	PRJNA546246	Song et al. (2020)
		Darmor-bzh	AACC_Darmor-bzh	PRJEB39416 and PRJEB39508	Rousseau-Gueutin et al. (2020)
Brassica carinata	BBCC	zd-1	BBCC	CRA002151, CRA002152, CRA002162, and CRA002177	Song et al. (2021)
